# Acceptor substrate determines donor specificity of an aromatic prenyltransferase: expanding the biocatalytic potential of NphB

**DOI:** 10.1007/s00253-020-10529-8

**Published:** 2020-03-18

**Authors:** Bryce P. Johnson, Erin M. Scull, Dustin A. Dimas, Tejaswi Bavineni, Chandrasekhar Bandari, Andrea L. Batchev, Eric D. Gardner, Susan L. Nimmo, Shanteri Singh

**Affiliations:** Department of Chemistry and Biochemistry, Stephenson Life Sciences Research Center, University of Oklahoma, 101 Stephenson Parkway, Norman, OK 73019 USA

**Keywords:** Biocatalysis, Diversification, Alkyl-donor, *N*-prenylation, Sulfabenzamide, Enzyme promiscuity

## Abstract

**Abstract:**

Aromatic prenyltransferases are known for their extensive promiscuity toward aromatic acceptor substrates and their ability to form various carbon-carbon and carbon-heteroatom bonds. Of particular interest among the prenyltransferases is NphB, whose ability to geranylate cannabinoid precursors has been utilized in several in vivo and in vitro systems. It has therefore been established that prenyltransferases can be utilized as biocatalysts for the generation of useful compounds. However, recent observations of non-native alkyl-donor promiscuity among prenyltransferases indicate the role of NphB in biocatalysis could be expanded beyond geranylation reactions. Therefore, the goal of this study was to elucidate the donor promiscuity of NphB using different acceptor substrates. Herein, we report distinct donor profiles between NphB-catalyzed reactions involving the known substrate 1,6-dihydroxynaphthalene and an FDA-approved drug molecule sulfabenzamide. Furthermore, we report the first instance of regiospecific, NphB-catalyzed *N*-alkylation of sulfabenzamide using a library of non-native alkyl-donors, indicating the biocatalytic potential of NphB as a late-stage diversification tool.

**Key Points:**

*• NphB can utilize the antibacterial drug sulfabenzamide as an acceptor.*

*• The donor profile of NphB changes dramatically with the choice of acceptor.*

*• NphB performs a previously unknown regiospecific N-alkylation on sulfabenzamide.*

*• Prenyltransferases like NphB can be utilized as drug-alkylating biocatalysts.*

**Electronic supplementary material:**

The online version of this article (10.1007/s00253-020-10529-8) contains supplementary material, which is available to authorized users.

## Introduction

Aromatic prenyltransferases (PTs) comprise a large class of enzymes catalyzing the transfer of a prenyl group from a prenyl-pyrophosphate donor onto an aromatic acceptor (Awakawa and Abe 2019; Saleh et al. 2009). This reaction scheme, delivers a high level of chemical diversity among the products of PT reactions arising from a combination of factors (Kumano et al. 2008; Kuzuyama et al. 2005; Shindo et al. 2011; Tanner 2015; Tello et al. 2008). For example, the accepted prenyl groups can vary in size (5-carbon dimethyallyl, 10-carbon geranyl, 15-carbon farnesyl, 20-carbon geranylgeranyl) between PTs, while the attachment can occur from either C1′ or C3′ of the prenyl donors onto various positions of the aromatic acceptors, resulting in normal or reverse prenylation, respectively (Giessen and Marahiel 2015; Winkelblech et al. 2015a). Furthermore, PTs have been shown to catalyze the formation of C–C (Araya-Cloutier et al. 2017; Bandari et al. 2019; Elshahawi et al. 2017; Fan et al. 2015b; Haagen et al. 2007; Winkelblech et al. 2015b), C–O (Bandari et al. 2017; Haagen et al. 2007; Kumano et al. 2008; Pockrandt et al. 2012; Rudolf et al. 2013; Shindo et al. 2011; Wunsch et al. 2015), C–N (Bonitz et al. 2013; Dalponte et al. 2018; Mahmoodi and Tanner 2013; Qian et al. 2012; Yin et al. 2007; Zou et al. 2011), and C–S bonds (Rudolf and Poulter 2013). Studies thus far reveal PTs to be highly flexible toward aromatic acceptors (Fan et al. 2015a; Kumano et al. 2008; Schuller et al. 2012; Shindo et al. 2011; Tanner 2015) as well as non-native alkyl donors (Bandari et al. 2019; Bandari et al. 2017; Liebhold and Li 2013; Liebhold et al. 2012; Liebhold et al. 2013; Yu et al. 2015). These promiscuous activities have been attributed to the spacious active site within the common PT fold, the ABBA motif, composed of 10 antiparallel β-strands forming a β-barrel and surrounded by α-helices alternating in an αββα pattern (Kuzuyama et al. 2005; Saleh et al. 2009).

**Fig. 1:**
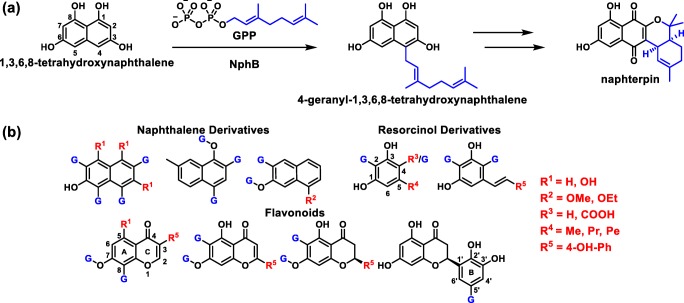
Reactions of NphB. **a** Recently proposed role of NphB in naphterpin biosynthesis (Murray et al. 2018). **b** Known substrates of NphB. Each known site of geranylation is highlighted with a blue “G”

The motif was originally elucidated from the crystal structure of NphB, a member of the Mg^2+^-dependent CloQ/NovQ family of PTs (Kuzuyama et al. 2005). This particular PT is involved in the biosynthesis of naphterpin in *Streptomyces* sp. CL190 (Fig. 1a), where it has been recently proposed to transfer a geranyl group onto the C4 position of the putative substrate 1,3,6,8-tetrahydroxynaphthalene (Murray et al. 2018). Beyond this natural activity, though, NphB is known to exhibit a high degree of promiscuity toward phenolic acceptors, including flavonoids, isoflavonoids, plant polyketides, and various naphthols (Fig. 1b) (Kumano et al. 2008; Kuzuyama et al. 2005; Shindo et al. 2011; Xiao et al. 2009). Furthermore, most of these reactions resulted in several geranylated products differing in their regiochemistry. For the dihydroxynaphthalenes (DHNs), normal geranylation occurred at positions ortho to one of the hydroxy groups, though one instance of substitution para to a hydroxy group was observed for 1,6-DHN (Kumano et al. 2008; Kuzuyama et al. 2005). Alkylated naphthol derivatives showed similar patterns of normal ortho and para substitution, though normal *O*-geranylation was also observed on the single hydroxy group (Shindo et al. 2011). Most of the flavonoids were geranylated at C6 of the A ring (ortho to both hydroxy groups) and on the C7-hydroxy oxygen (Kumano et al. 2008; Kuzuyama et al. 2005), though a chrysin analog generated by Shindo et al. (2011) with 2,3-dihydroxylation of the C ring was geranylated para to the 2′-hydroxy moiety on this ring. Finally, the resorcinol derivatives underwent *C*-geranylation at C2 or C4, ortho to both or one hydroxy group respectively (Kumano et al. 2008; Qian et al. 2019; Valliere et al. 2019). As for non-GPP donors, NphB has been shown to utilize a terminal azido-substituted GPP derivative with 1,6-DHN, installing it ortho to the C6 hydroxy group, as well as farnesyl pyrophosphate (FPP) with 1,6-DHN and unreported regiospecificity (Kuzuyama et al. 2005; Xiao et al. 2009).

The established promiscuity of NphB, particularly with resorcinol derivatives, has focused attention on the enzyme in recent years for its biocatalytic potential. As a result, a handful of biocatalytic platforms have been developed using NphB for its natural geranylating activity. In 2017, the enzyme was recombinantly expressed in the yeast *Komagataella phaffii* with tetrahydrocannabinolic acid synthase from *Cannabis sativa*. The resulting lysate was shown to produce Δ^9^-tetrahydrocannabinolic acid from GPP and olivetolic acid (Zirpel et al. 2017), demonstrating the viability of NphB as a biocatalyst in the production of valuable cannabinoids. This initial utility was further expanded in 2019 when NphB was implemented in a multi-enzyme, “synthetic biochemistry” platform for the generation of various cannabinoids (Valliere et al. 2019). The optimized system, which included an NphB variant (G286SY288A) engineered to produce cannabigerolic acid (CBGA) almost exclusively from olivetolic acid, produced 1.25 g/L of this cannabinoid over 24 h. Furthermore, an in vivo system was published in the same year that produced geranylated phenolic acids, including CBGA, in *Escherichia coli* using an engineered NphB variant (G286S) (Qian et al. 2019). This study also established the NphB variant’s ability to utilize farnesyl pyrophosphate (FPP) to a greater extent than the wild-type enzyme in the production of farnesylated phenolic acids. Overall, these studies have recontextualized NphB as a prominent biocatalyst whose prenylating activity is useful in the production of pharmacologically relevant compounds.

Nevertheless, the role of NphB presented in these works is limited mostly to its natural geranylating activity. The enzyme’s utility as a biocatalyst could likely be expanded considering the wider promiscuities of PTs with respect to their alkyl-donors. Recent studies, including those with the tyrosine-*O*-PT SirD and the tryptophan *C*4-PT FgaPT2, have revealed the ability of PTs to transfer a wide array of non-native alkyl-groups onto both native and non-native aromatic acceptors (Bandari et al. 2019; Bandari et al. 2017; Liebhold and Li 2013; Liebhold et al. 2012; Liebhold et al. 2013; Winkelblech et al. 2015b; Yu et al. 2015). These types of late-stage functionalization reactions on aromatic rings are usually considered difficult to achieve with traditional synthetic chemistry (Hong 2014). Therefore, a biocatalyst capable of performing such reactions with predictable regiospecificity would be incredibly useful from a methodology perspective. Furthermore, facile access to these types of reactions opens a vast chemical space for pharmacologically relevant compounds, allowing one to conduct extensive structure-activity relationship (SAR) studies. Within this context, the precedence of NphB in biocatalytic systems provides a preemptive solution to the optimization of a new platform for late-stage modification. However, this platform can only be developed if NphB demonstrates activity with a range of non-native alkyl donors, of which only one (azido-GPP) has been confirmed (Xiao et al. 2009). Thus, the goal of this study was to elucidate NphB’s ability to transfer a multitude of non-native alkyl groups from synthetic alkyl-pyrophosphates (alkyl-PPs) onto diverse and potentially useful aromatic acceptors. To this end, a panel of structurally disctint, aromatic drug molecules were screened for activity with NphB using two natural donors, DMAPP and GPP. From this initial screen, a single hit was identified: the antibacterial compound sulfabenzamide with DMAPP, a surprising result considering NphB’s natural geranylating activity. To further investigate this donor promiscuity, a library of 66 unique alkyl-PP analogs was generated and tested using two acceptors, the known substrate 1,6-DHN and the newly discovered substrate sulfabenzamide. The subsequent activity assays demonstrated that NphB can transfer an array of non-native alkyl groups onto both acceptors, but the donor specificity of the enzyme depends heavily on the specific acceptor substrate. In addition, structural characterization of diverse sulfabenzamide derivatives revealed a previously unreported activity of NphB to perform regiospecific and chemoselective *N*-alkylation. Thus, the present study establishes alkyl-diversification as a legitimate biocatalytic pursuit for NphB while also illuminating the general considerations needed to utilize it.

## Materials and methods

### General reagents and materials

Unless otherwise stated, all chemicals and reagents were purchased from Sigma-Aldrich (St. Louis, MO, USA), Acros (New Jersey, USA), Alfa Aesar (Ward Hill, MA, USA), or TCI (Portland, OR, USA) and were reagent grade or better.

### Synthesis, purification, and characterization of alkyl-PP analogs

Procedures for the synthesis of alkyl-pyrophosphate analogs **1**, **2**, **4**–**7**, **9**, **11**–**13**, **17**, **18**, **20**, **24**–**26**, **37**, **45**–**57** have been reported previously (Bandari et al. 2019; Bandari et al. 2017). All other analogs (**3**, **8**, **10**, **14**–**16**, **19**, **21**–**23**, **27**–**36**, **38**–**44**, **58**–**66**) were synthesized using 1 equiv. of corresponding alcohol and an excess of bis(triethylammonium) phosphate (TEAP) solution. This solution (25.0–100.0 mmol) was formed by mixing 36 mL reagent A (94 mL acetonitrile and 25 mL H_3_PO_4_) and 60 mL reagent B (110 mL triethylamine and 100 mL acetonitrile). Each alcohol was first dissolved in 20 mL trichloroacetonitrile (25 equiv.) in a round-bottom flask and stirred at room temperature for a total of 25 min, with 20 mL of TEAP solution being added to initiate the reaction and subsequently at both the 5- and 10-min time points. The products were purified by flash column chromatography in 7:2:1 isopropanol/NH_4_OH/H_2_O, yielding white amorphous solids. The identity of each analog was confirmed by nuclear magnetic resonance (NMR) spectroscopy and either high-resolution mass spectrometry (HRMS) or liquid chromatography mass spectrometry (LCMS) (Supplementary Material). NMR spectra were obtained on a Varian VNMRS 500 MHz instruments at the NMR core facility of the Department of Chemistry and Biochemistry of the University of Oklahoma using 99.9% D_2_O or DMSO-D_6_ with 0.05% v/v TMS. ^1^H, ^13^C, and ^31^P chemical shifts were referenced to internal standards or solvent resonances. All NMR spectra were recorded at ambient temperature and processed using Mnova (Mestrelab Research, L.C., Santiago de Compostela, Spain). HRMS and LCMS data were obtained on an Agilent 6545-QTOF W/1290 HPLC mass spectrometer at the mass spectrometry core facility of the Department of Chemistry and Biochemistry of the University of Oklahoma.

### Overexpression and purification of NphB

A synthetic gene of wild-type NphB (GenBank: BAE00106.1) was purchased from Genscript Biotech (Piscataway, NJ) with codons optimized (see Supplementary Material Fig. S1a) for expression in *E. coli*. This gene was inserted into the pET-28a to obtain a N-terminal His_6_-tagged construct (full amino acid sequence in Supplementary Material Fig. S1b), and the resulting recombinant plasmid was transformed into *E. coli* Rosetta cells. After overnight growth, 3 mL of bacterial culture was transferred to 4-L flasks containing 1–1.2 L Luria-Bertani medium and 50 μg mL^−1^ kanamycin. Cultures were then grown at 37 °C and 220 RPM for approximately 4 h. Once OD_600_ = 0.6–0.8, protein expression was induced by the addition of isopropyl β-D-1-thiogalactopyranoside (IPTG, 0.5 mM final), and the cultures were continually shaken at 220 RPM and 20 °C for an additional 16–19 h. Cells were harvested by centrifugation, with the resulting pellets resuspended in a lysis buffer (300 mM NaCl, 10 mM imidazole, 50 mM NaH_2_PO_4_ pH 7.8). Cells were lysed by way of sonication on ice for 40 min in total in cycles of 20 s pulses followed by 30 s rest periods (Fisher Scientific Model FB505; Thermo Fisher Scientific, Waltham, MA). Cultures were disturbed gently after every 10 min of sonication to ensure complete lysis. To remove unwanted cellular debris and insoluble protein, the lysed cells were spun down at 16,000 RPM for 1 h at 10 °C. The supernatant, containing the expressed recombinant NphB with an N-terminal His_6_-tag, was purified using a 5-mL Ni Sepharose resin affinity chromatography column from GE Healthcare (Piscataway, NJ). Protein was eluted at 1.5 mL/min using a gradient of 2 to 10% solution B over 25 mL, a gradient of 10 to 100% solution B over 50 mL, and an isocratic flow of 100% solution B for 50 mL (solution A = 200 mM NaCl, 10 mM imidazole, and 50 mM NaH_2_PO_4_ pH 7.8; solution B = 200 mM NaCl, 500 mM imidazole, and 50 mM NaH_2_PO_4_ pH 7.8). The purified protein was concentrated through centrifugal filtration and exchanged to a new buffer condition (50 mM KCl, 25 mM Tris-HCl pH 8) using a PD10 column from GE Healthcare. Purity was evaluated by SDS-PAGE (Supplementary Material Fig. S2), and the resulting protein bands indicated both a high grade of purity and the appropriate molecular weight (~ 36 kDa) for NphB. Final protein concentration was determined using a NanoDrop™ One Microvolume UV-vis Spectrophotometer (Thermo Fisher Scientific, Waltham, MA).

### Analytical NphB-catalyzed reactions

Two sets of in vitro NphB analytical reactions were performed using the same conditions: (1) an initial screening of drug molecules to identify acceptor substrates of NphB and (2) a screen of the synthetic alkyl-PPs against the acceptors 1,6-DHN and sulfabenzamide. The initial acceptor screen utilized DMAPP (**2**) and GPP (**32**) as potential donors and the following drug molecules as potential acceptors: amoxicillin, chloramphenicol, equilin, ethinylestradiol, mebendazole, pyrimethamine, sulfabenzamide, sulfadoxine, and tetracycline. Subsequent reactions were conducted in a final volume of 20 μL, consisting of 1.2 mM alkyl-PP analogs, 1 mM 1,6-DHN (Alfa Aesar) or sulfabenzamide (Sigma-Aldrich), and 6 μM purified NphB in a reaction buffer (25 mM Tris pH 8.0, 5 mM MgCl_2_, 50 mM KCl). Reactions were incubated at 35 °C for 16 h and quenched using 40 μL cold methanol, which was followed by centrifugation (9000*g* for 30 min) to remove precipitated protein. Reaction analysis was completed using reverse-phase high-performance liquid chromatography (RP-HPLC) on an Agilent 1220 system equipped with a DAD detector, and it employed a Gemini-NX C-18 (5 μm, 4.6 mm × 250 mm) column (Phenomenex, Torrance, California, USA). 1,6-DHN analytical reactions were separated using the following method: gradient of 10% B to 100% B over 22 min, isocratic flow of 100% B for 2 min, gradient of 100% B to 10% B over 0.1 min, and isocratic flow of 10% B over 5.9 min (A = ddH_2_O with 0.1% TFA; B = acetonitrile) at flow rate = 1 mL min^−1^; A_254_. Sulfabenzamide analytical reactions were separated using the following method: gradient of 1% B to 10% B over 10 min, 10% B to 50% B over 5 min, 50% B to 100% B over 12 min, 100% B to 1% B over 1 min, and isocratic flow of 1% B for 7 min (A = ddH_2_O with 0.1% TFA; B = acetonitrile) at flow rate = 1 mL min^−1^; A_254_. The reactions were monitored by the retention time differences between the starting material and product(s).

### Large-scale NphB-catalyzed sulfabenzamide reactions

NphB large-scale reactions were conducted in a volume of 10 mL consisting of 4.16 mM alkyl-PP analog, 6 mM sulfabenzamide, and 10 μM purified NphB in a reaction buffer (25 mM Tris pH 8.0, 5 mM MgCl_2_, 50 mM KCl). Putative products were subsequently isolated by semi-preparative RP-HPLC on an Agilent 1220 system equipped with a DAD detector. The method used a Gemini-NX, C-18 (5 μm, 10 × 250 mm) column (Phenomenex, Torrance, California, USA) to purify the sulfabenzamide analogs (gradient of 1% B to 10% B over 10 min, 10% B to 50% B over 5 min, 50% B to 100% B over 12 min, 100% B to 1% B over 1 min, and isocratic flow of 1% B for 7 min (A = ddH_2_O with 0.1% formic acid; B = acetonitrile) flow rate = 2 mL min^−1^; A_254_). The identity of each derivative was confirmed by NMR spectroscopy and HRMS using positive (+) and/or negative (−) mode(s) as previously described for the alkyl-PP analogs.

### In silico docking studies

Sulfabenzamide was manually docked into the crystal structure of wild-type NphB originally containing 1,6-DHN and GSPP (PDB ID: 1ZB6, Kuzuyama et al. 2005) using PyMol (Schrödinger, New York City, NY).

## Results

### Identification of sulfabenzamide as an acceptor substrate for NphB

**Fig. 2:**
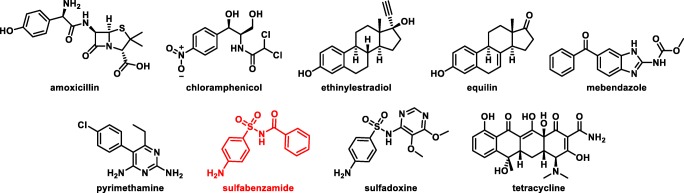
Drug molecules screened as potential acceptors in NphB-catalyzed reactions. The identified hit is highlighted in red

Initial analytical screens using two natural prenyl donors, **2** and **32**, were performed to test the acceptor promiscuity of NphB toward various drug scaffolds (Fig. 2). Based on the known acceptor profile of NphB (Fig. 1b), potential acceptors were chosen based on the inclusion of phenol or a phenol-like moiety with unsubstituted positions ortho to the hydroxy or analogous group. Of all eighteen reactions tested on an analytical scale, only one showed turnover via RP-HPLC: sulfabenzamide with **2**, which was surprising given the enzyme’s native geranylating activity (Kumano et al. 2008; Kuzuyama et al. 2005; Shindo et al. 2011). Thus, the new findings implied a potential link between acceptor and donor specificity which could further expand the utility of NphB in biocatalytic systems if fully elucidated. To more firmly establish this link, a library of diverse alkyl-PPs was synthesized and screened against the newly identified acceptor as well as the known acceptor 1,6-DHN.

### Synthesis of alkyl-PP library

**Fig. 3:**
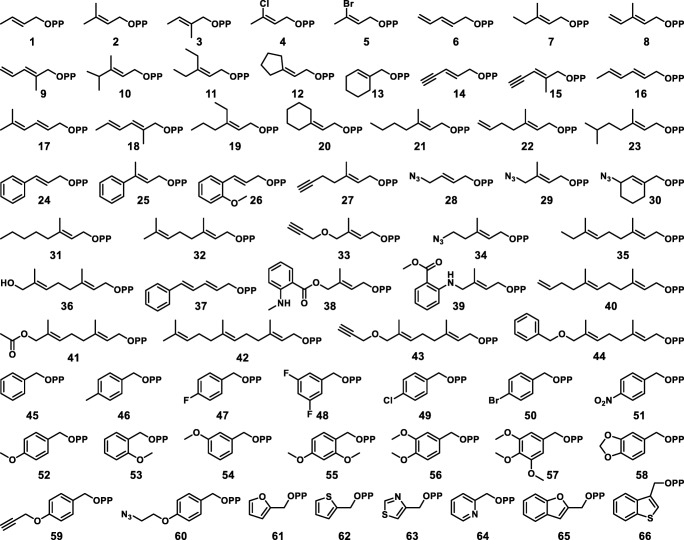
Library of alkyl-PP analogs used in this work

The design of alkyl-PP analogs (Fig. 3) was consistent with the need of PTs to accept alkyl-donors containing a double bond at the β-position (Bandari et al. 2017). With this limitation in mind, the final library of 66 native and non-native alkyl-PP analogs, roughly demarcated between allylic (**1**–**44**) and aromatic (**45**–**66**) groups, was synthesized to explore as large of a chemical space as was feasible. The allylic analogs were designed either to match the natural prenyl donors dimethylallyl pyrophosphate (DMAPP, **2**), geranyl pyrophosphate (GPP, **32**), and farnesyl pyrophosphate (FPP, **42**) (Winkelblech et al. 2015a) or to resemble them with variations in alkyl-chain length, electronic character, and the presence or positioning of branching methyl groups. The resulting library included aliphatic analogs published previously (**1**, **4**–**7**, **9**, **11**–**13**, **17**, **18**, **20**, **24**–**26**, **37**) (Bandari et al. 2019; Bandari et al. 2017), novel GPP derivatives with various terminal groups (**35**, **36**, **40**, **41**, **43**, **44**), and new alkyl-PP analogs with chain lengths between DMAPP and GPP (**21**–**23**, **31**). The aromatic donors were similarly designed to test variations in the number and position of benzene substitutions, the effect of electron-donating and electron-withdrawing groups on the benzene ring, and the presence of various heteroaromatic systems. Syntheses and utility of most of the benzylic alkyl-PPs were published previously to demonstrate their acceptability with the tyrosine-*O*-PT SirD and the tryptophan *C*4-PT FgaPT2 (**45**–**57**) (Bandari et al. 2019), but three novel benzylic analogs were synthesized to either understand the effects of conformational constraint (**58**) or to introduce synthetic handles (**59**, **60**). Furthermore, the abundance of heterocycles across the spectrum of known drugs (Taylor et al. 2016) prompted the synthesis of novel alkyl-PPs with diverse heteroaromatic characters (**61**–**66**). Finally, to explore the potential applications of alkylated products, a small pool of alkyl-PPs was synthesized to contain functionalities amenable to either selective downstream chemistry or direct utility in research settings: alkynes (**14**, **15**, **27**, **33**, **43**, **59**) and azides (**28**–**30**, **34**, **60**) for Cu-mediated click chemistry (Meghani et al. 2017), dienes (**6**, **8**, **9**, **16**–**18**, **37**) and terminal alkenes (**22**, **40**) for Diels-Alder chemistry (Bouchez et al. 2016; Gregoritza and Brandl 2015), and fluorescent probes (**38**, **39**) for bioimaging (Lu et al. 2019). With these factors in mind, the final alkyl-PP library contained 66 compounds that covered a vast chemical space and was capable of thoroughly probing the donor specificity of NphB.

### Donor profiles of 1,6-DHN and sulfabenzamide with NphB

**Fig. 4:**
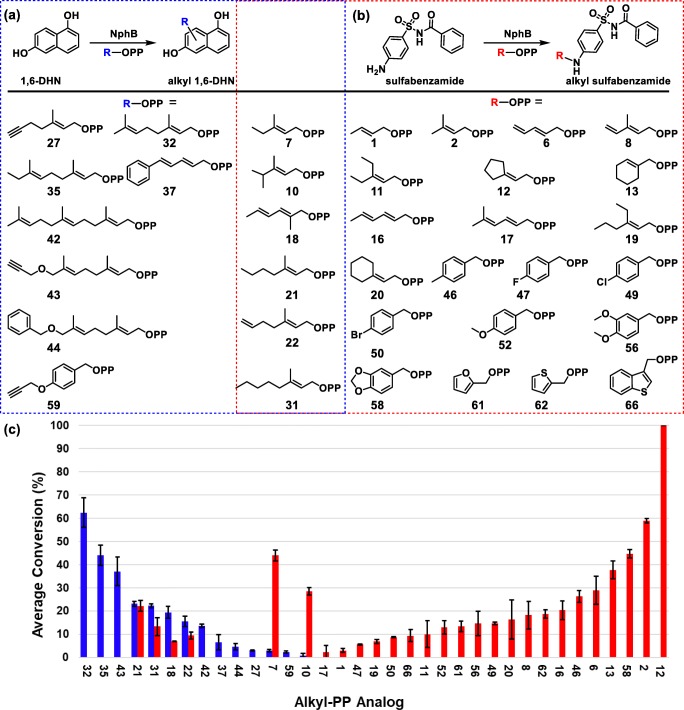
Donor profiles of wild-type NphB-catalyzed reactions with acceptors 1,6-DHN and sulfabenzamide. **a** The general reaction scheme of donor screening with 1,6-DHN and the utilized alkyl-PP donors (boxed in blue). **b** The general reaction scheme of donor screening with sulfabenzamide and the utilized alkyl-PP donors (boxed in red). Analogs contained in both boxes were utilized with both acceptors. **c** Conversion rates of successful analytical scale reactions between aromatic acceptors and alkyl-PP donors, using the same color scheme as in **a** and **b**. Each reaction was carried out in a 20-μL volume and contained 1.2 mM alkyl-PP analog, 1 mM 1,6-DHN or sulfabenzamide, and 6 μM purified NphB in a reaction buffer consisting of 25 mM Tris pH 8.0, 5 mM MgCl_2_, 50 mM KCl incubated at 35 °C for 16 h

1,6-DHN was chosen as one of the acceptor substrates for this study due to its status as the putative substrate of NphB and its assumed similarity to the true natural acceptor (Kuzuyama et al. 2005). Therefore, it was screened against the 66 alkyl-PPs in analytical scale reactions using NphB as the catalyst. The resulting profile (Fig. 4, Supplementary Material Table S1 and Fig. S3) indicates the enzyme transferred 14 of the 66 analogs, of which 13 were allylic (**7**, **10**, **18**, **21**, **22**, **27**, **31**, **32**, **35**, **37**, **42**–**44**) and one was benzylic (**59**). In terms of quantified conversion, a single analog (**32**, GPP) showed > 50%, two (**35**, **43**) showed > 30%, two (**21**, **31**) showed 20–25%, three (**18**, **22**, **42**) showed 10–20%, and the remaining six (**7**, **10**, **27**, **37**, **44**, **59**) showed < 10%. Furthermore, the chromatograms of five reactions (**18**, **32**, **35**, **42**, **43**, Supplementary Material Fig. S3) indicated the formation of multiple products. Based on these observations and the correlating structures (Fig. 4a), NphB generally tended to transfer groups onto 1,6-DHN with longer (> 6 unbranched carbons) aliphatic chains usually lacking bulky aromatic rings or hydrophilic moieties within 5 unbranched carbons of the pyrophosphate. Within this generalized aliphatic structure, the natural geranyl donor **32** showed the highest turnover at 62 ± 6%, while even relatively minor modifications to the geranyl scaffold (terminal methylation with **35**, terminal di-demethylation with **22**) resulted in much reduced conversion (difference > 10%) compared with **32**. As for functionalized moieties, NphB utilized three alkynes (**27**, **43**, **59**), two dienes (**18**, **37**), and the shorter terminal alkene (**22**). Though the turnovers for most of these reactions were low (< 10%), the success of these reactions, particularly with **43**, hints at the possibility of using NphB to introduce chemical handles on molecules for downstream chemistry. In terms of its general utility, however, NphB appears to prefer GPP and its derivatives for alkylation of 1,6-DHN.

Sulfabenzamide was screened against the alkyl-PP library under the same conditions as 1,6-DHN (Fig. 4b, Supplementary Material Table S2). The resulting donor profile contained twenty-seven alkyl-PPs in total, split among seventeen allylic moieties (**1**, **2**, **6**–**8**, **10**–**13**, **16**–**22**, **31**), seven benzylic groups (**46**, **47**, **49**, **50**, **52**, **56**, **58**), and three heterocycles (**61**, **62**, **66**). Two of these alkyl-PPs showed conversion > 50% (**2**, **12**), six showed 25–50% (**6**, **7**, **10**, **13**, **46**, **58**), eleven showed 10–25% (**8**, **11**, **16**, **20**, **21**, **31**, **49**, **52**, **56**, **61**, **62**), and the remaining eight showed < 10% (**1**, **17**–**19**, **22**, **47**, **50**, **66**). All reactions, however, seemed to have generated a single product based on the chromatograms (Supplementary Material Fig. S4). Analysis of the accepted analog structures (Fig. 4b) revealed NphB tended to transfer aliphatic groups onto sulfabenzamide with short carbon chain lengths (≤ 6 unbranched carbons) while also accommodating several aryl (**46**, **47**, **49**, **50**, **52**, **56**, **58**, **61**, **62**, **66**) and non-aromatic cyclic (**12**, **13**, **20**) groups close to C1′ of the donor. In fact, despite **2** being the natural prenyl donor DMAPP, the cyclopentyl analog **12** showed the greatest conversion at a consistent 100% (compared with 58.9 ± 0.9% for **2**). The compound with the third highest conversion (45 ± 2%) was also cyclic, the benzodioxole-containing benzylic analog **58**. As for the functionalized alkyl-PPs, NphB transferred six in total, including five dienes (**6**, **8**, **16**–**18**) and a single terminal alkene (**22**). Three of these moieties (dienes **6**, **8**, **16**) even showed conversion > 10%, with **6** approaching ~ 30%. This is in stark contrast to 1,6-DHN, where all the functionalized alkyl-PPs turned over > 10% using standard reaction conditions. The simple switch in acceptor increasing the activity of NphB with these types of groups provides further evidence of its possible utility in introducing reactive handles onto aromatic scaffolds. Overall, though, the choice of sulfabenzamide as the acceptor substrate appears to limit the donor specificity to DMAPP, DMAPP derivatives, and compounds with cyclic moieties attached within 2 carbons lengths of C1′.

Taken together, the donor profile of sulfabenzamide was distinct from that of 1,6-DHN (Fig. 4). Although six donors were utilized with both acceptors (**7**, **10**, **18**, **21**, **22**, **31**), the switch from 1,6-DHN to sulfabenzamide introduced new activity with twenty-one donors not previously observed (**1**, **2**, **6**, **8**, **11**–**13**, **16**, **17**, **19**, **20**, **46**, **47**, **49**, **50**, **52**, **56**, **58**, **61**, **62**, **66**). Most of these were alkyl-PPs with short allylic chains (~ 6 unbranched carbons), though several benzylic groups and three heterocycles were also utilized. Thus, the major difference between the donors exclusive to one acceptor seems to be their generalized sizes (both length and nature of substituent). This trend even holds for the overlapping alkyl-PPs, as the smaller analogs (≤ 5 unbranched carbons: **7**, **10**) gave conversions at least 15% higher with sulfabenzamide than with 1,6-DHN. Meanwhile, overlapping groups with chains of intermediate length (6–8 unbranched carbons: **18**, **21**, **22**, **31**) displayed either greater conversion (~ 7% difference) or comparable conversion with 1,6-DHN. Collectively, the donor profiles of 1,6-DHN and sulfabenzamide establish a dependency of donor substrate specificity on the acceptor substrate in NphB-catalyzed reactions.

### Determination of regiospecificity in sulfabenzamide reactions

Consistent with the known multi-site prenylation of 1,6-DHN by NphB (Kumano et al. 2008; Kuzuyama et al. 2005), the HPLC chromatograms of several reactions (**18**, **32**, **35**, **42**, **43**, Supplementary Material Fig. S3) of 1,6-DHN by NphB indicated the presence of multiple products. In contrast, NphB-catalyzed alkylation reactions with sulfabenzamide resulted in a single product. Therefore, to understand the regioselectivity of the newly discovered activity with sulfabenzamide, a representative set of diverse sulfabenzamide reactions were scaled up, purified by semi-preparative RP-HPLC, and characterized by NMR spectroscopy. Most of the analyzed products came from reactions displaying > 25% average conversion in the initial analytical screen and possessed an alkyl group with unique electronic or structural features, i.e., carbon chain length, conjugated systems, cyclization, and aromaticity. These include derivatives obtained from reactions with DMAPP (**2**); ethyl and isopropyl DMAPP derivatives (**7**, **10**); diene analogs with unbranched carbon chain lengths of 5 and 6 (**6**, **16**); two non-aromatic, cyclized pyrophosphates (**12**, **13**); and two benzylic analogs (**46**, **58**). Additionally, the successful reactions with heterocyclic donors (**61**, **62**) were included due to the prevalence of heterocycles in medicinal chemistry (Taylor et al. 2016). The resulting purified sulfabenzamide products were subjected to ^1^H and COSY experiments. Additionally, HMBC and HSQC spectra were recorded for representative sufabenzamide derivatives of **10**, **58**, **61**, and **62** for unambiguous determination of the position of alkylation.

**Fig. 5:**
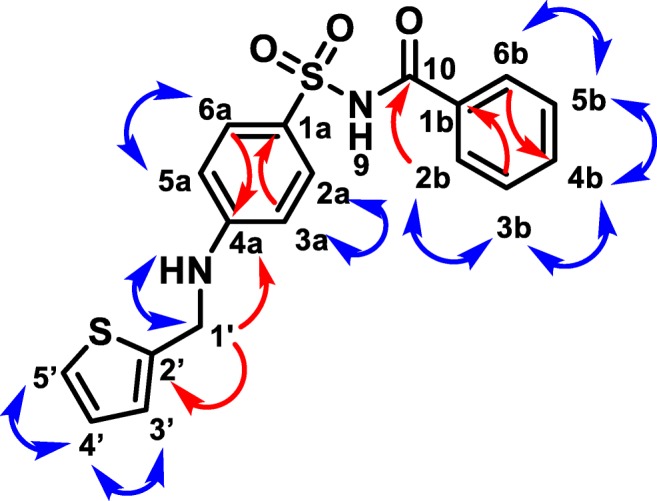
A representative example (**77**) of NMR correlations for scaled-up sulfabenzamide derivatives. Observed ^1^H-^1^H COSY correlations are shown as blue double-headed arrows. Relevant ^1^H-^13^C HMBC correlations are shown as red single-headed arrows

The complete NMR analysis (chemical shift values, splitting pattern, COSY and/or HMBC correlations) of all scaled-up sulfabenzamide products established that NphB-catalyzed regiospecific and chemoselective *N*-alkylation of the 4-aminobenzenesulfonyl ring (Fig. 5, Supplementary Material Table S5). The ^1^H NMR of the derivatives, when compared with the parent sulfabenzamide, revealed all aromatic protons in the 6.0–8.0 ppm range which were accounted for on both rings of the parent compound, strongly suggesting an *N*-substitution. Signals corresponding to the two aromatic rings could be differentiated by their splitting pattern, with the benzamide ring displaying three distinct mutliplet peaks integrating to a 2:2:1 ratio and the 4-aminobenzenesulfonyl ring having two doublet peaks integrating to two protons each. Assignments of the aromatic proton signals were further supported by the COSY spectra showing correlation peaks between the H2a/6a-H3a/5a on the 4-aminobenzenesulfonyl ring as well as correlation peaks between H2b/6b-H3b/5b and H4b-H3b/5b on the benzamide ring (see Supplementary Material). In some cases, the COSY spectra also showed a correlation between the proton on the amino NH and the H1′ of the substituent alkyl or benzyl group. In addition, the ^1^H–^13^C HMBC and ^1^H–^13^C HSQC spectra displayed characteristic resonances and connectivity between H1′ of the donor group and the C4a of the 4-aminobenzenesulfonyl ring, unequivocally confirming the *N*-alkylation. Each scale up reaction was additionally confirmed by HRMS data, supporting the presence of a single mono-alkylated product per reaction (Supplementary Material Table S4). To our knowledge, this is the first reported instance of NphB-catalyzed *N*-alkylation, which further expands the applicability of NphB in biocatalytic systems involving alkylation of natural products.

## Discussion

**Fig. 6:**
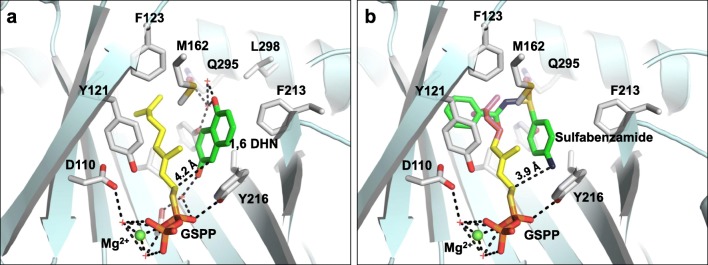
**a** Active site structure of NphB bound to 1,6-DHN and GSPP (PDB ID: 1ZB6, Kuzuyama et al. 2005). **b** A docked model of the NphB active site containing sulfabenzamide with its amine group within hydrogen bonding distance (3.9 Å) of C1′ of GSPP. The C1′–C5′ of GSPP are colored yellow, while the C6′–C10′ are colored magenta

The donor profiles of 1,6-DHN and sulfabenzamide in reactions catalyzed by NphB showed highly distinct trends, with 1,6-DHN favoring longer aliphatic moieties (~ 8–9 unbranched atoms) and sulfabenzamide showing a preference for shorter ones (~ 5–6 unbranched atoms). Furthermore, NphB catalyzed far more reactions with aromatic alkyl-PPs, both benzylic and heterocyclic, when sulfabenzamide was the acceptor (twelve vs. two with 1,6-DHN), while only reactions with 1,6-DHN could form multiple products. To explain the observed trends, sulfabenzamide was docked in the active site of the NphB crystal structure bound to geranylthiolopyrophosphate (GSPP) and 1,6-DHN (PDB ID: 1ZB6; Kuzuyama et al. 2005; Fig. 6a). In the crystal structure, the geranyl tail of GSPP is highly stabilized through *π*-*π* stacking interactions with 1,6-DHN and Tyr121, along with additional hydrophobic interactions with Val49, Phe123, Met162, and Tyr288 (Fig. 6a). In the docked model, sulfabenzamide replaced 1,6-DHN and was placed in a similar orientation, such that the amine group was within hydrogen bonding distance to C1′ of the donor to facilitate *N*-alkylation (Fig. 6b) as observed (**67**–**77**). The NphB structure, along with the docked model, indicates a reduction in available space that occurred in the active site when switching from 1,6-DHN to sulfabenzamide (Fig. 6), which can be rationalized by each molecule’s structure. In terms of the relative size, sulfabenzamide would be considered larger than 1,6-DHN based solely on the number of non-hydrogen atoms in each (19 and 12, respectively). Additionally, the fused bicyclic aromatic system of 1,6-DHN causes it to be planar and compact, while the flexible sulfonamide linker of sulfabenzamide introduces both three-dimensionality and a greater two-dimensional distance between the ends of the rings. This linker would thus force the unsubstituted ring to partially occlude the donor binding site (Fig. 6b). Coupled with the inherent rigidity of the geranyl group, this occlusion would likely cause GPP to be excluded from the active site and no product to be formed, matching the experimental data obtained in this study. The occlusion would also explain the generally shorter alkyl groups transferred onto sulfabenzamide, as they would fit more easily into the reduced active site space. NphB did, however, show measurable conversion for sulfabenzamide with **31**, a GPP homolog missing a terminal methyl group and the C6′–C7′ double bond. Although the two alkyl-PPs are approximately of the same length from end to end, the removal of these two features most likely increases the flexibility of the chain in **31** such that the last four C–C *σ* bonds can freely rotate away from the occluding ring of sulfabenzamide. The required rotations would, however, greatly reduce the number of conformations viable for reaction, thus decreasing the conversion of **31** compared with an alkyl group like **2** small enough to fit in the active site without additional rotation. This could explain the relatively low conversion of **31** (13.3 ± 3%) compared with **2** (58.9 ± 0.9%).

Size alone, however, is not enough to explain the finer trends in the diverse donor profile of sulfabenzamide. The mechanism of NphB-catalyzed reactions, in which the carbocation is attacked by the nucleophilic acceptor (Luk and Tanner 2009), exists in a delicate equilibrium of stability, as it must be (i) stabilized enough to exist before the nucleophilic attack (Yang et al. 2012) and (ii) desolvated completely to prevent premature quenching (Lesburg et al. 1998). Both the active site residues and the donor structure contribute to this balance in complex ways. For example, PTs often contain aromatic residues like Tyr, Trp, and Phe in the donor binding site to introduce *π*-*π* stacking interactions that further stabilize the allylic carbocation (Metzger et al. 2010). Therefore, to interpret the finer details of the donor profiles considering the same active site and acceptor, one must closely inspect both the steric and electronic differences between donors that may contribute to carbocation stability and reactivity. Within this context, the highest conversion in the sulfabenzamide analytical screens was observed with **12** (100%) as opposed to the natural prenyl donor **2** (DMAPP, 58.9 ± 0.9%). Assuming the same binding mode for sulfabenzamide in Fig. 6b, C1′ in **12** could have been closer to the site of alkylation compared with C1′ in **2**. The two additional methylenes of **12** would be constrained out of planarity with the rest of the alkyl tail by the *sp*^*3*^-hybridized carbons to which they are bonded, introducing additional steric interactions with sulfabenzamide. These new interactions likely caused the cyclopentane ring to tilt away from the acceptor compared with **2** (colored yellow in Fig. 6b), forcing C1′ closer to the site of alkylation due to the planarity of the rest of the molecule.

As for the aromatic moieties, there appears to be a complex interplay between the steric and electronic characteristics of each within the binding pocket. For example, **58** showed the greatest conversion among all benzylic alkyl-PPs with sulfabenzamide (45 ± 2%), far surpassing its non-cyclized homolog **56** (15 ± 5%), while **46** showed the next highest conversion (26 ± 3%). The conformational constraint of the cyclized methylenedioxy group of **58** compared with the two methoxy groups of **56** would explain this first difference with a purely steric argument. However, the intermediate conversion of **46** was likely due to its capacity for lower steric hindrance than **56** or **58** balanced by a carbocation less stabilized through induction with a methyl group vs. resonance with a *p*-methoxy group. As for the non-benzylic heterocycles, turnover was observed for sulfabenzamide with **61** (13 ± 2%), **62** (19 ± 2%), and **66** (9 ± 3%). Again, steric and electronic factors seem to be working in complex ways to determine substrate specificity; **62** had the highest turnover likely because it combines a smaller ring size (5 vs. 6 atoms) with the higher hydrophobic character and increased resonance stabilization of the carbocation by sulfur vs. oxygen. The 5-membered ring of **61**, however, seemed to balance its smaller size with its relative hydrophilicity and decreased carbocation stabilization, giving the second highest conversion over the larger **66**. Meanwhile, the higher hydrophobicity of the sulfur in **66** compared with the oxygen in **65** was enough for the former to react at all with sulfabezamide. There were also interesting differences in the transfer of functionalized moieties onto sulfabenzamide, which only accepted five dienes (**6**, **8**, **16**–**18**) and a single terminal alkene (**22**). The accepted moieties were relatively short in length (~ 6 unbranched atoms), which aligned with the general trend observed for alkyl groups transferred to sulfabenzamide. However, the inability of NphB to transfer alkyne substituents onto sulfabenzamide, even those resembling **2** in length (~ 6 unbranched atoms), could be related to the steric clashes caused by the rotation of the linear alkynes around C–C *σ* bonds. The same could be true for the linear azide groups, although the fully charged nitrogens may also be excluded from the overall hydrophobic NphB binding pocket. Overall, these trends illustrate that even slight modifications to donor structure can have a considerable effect on the enzyme’s ability to catalyze a reaction of interest.

Understanding how these structural variations in the donor affect activity with a desired acceptor will be crucial to further develop NphB as a biocatalyst. Thus far, the enzyme has been utilized in biocatalytic systems almost exclusively for its ability to geranylate diverse aromatic acceptors (Qian et al. 2019; Valliere et al. 2019; Zirpel et al. 2017). However, the donor promiscuity established in this work opens up the possibility of utilizing NphB for the late-stage modification of aromatic compounds. Furthermore, using the known crystal structure of NphB (Kuzuyama et al. 2005), one could anticipate how engineering specific residues within the active site alters the donor specificity with an acceptor of interest, thereby introducing activity with a preferred donor. However, such applications are not limited to NphB; PTs at large are known for their promiscuity toward acceptors (Araya-Cloutier et al. 2017; Awakawa and Abe 2019; Elshahawi et al. 2017; Rudolf and Poulter 2013; Schuller et al. 2012; Wunsch et al. 2015). Therefore, the underlying concepts of acceptor-dependent donor specificity established for NphB most likely apply to the enzyme class as a whole.

**Fig. 7:**
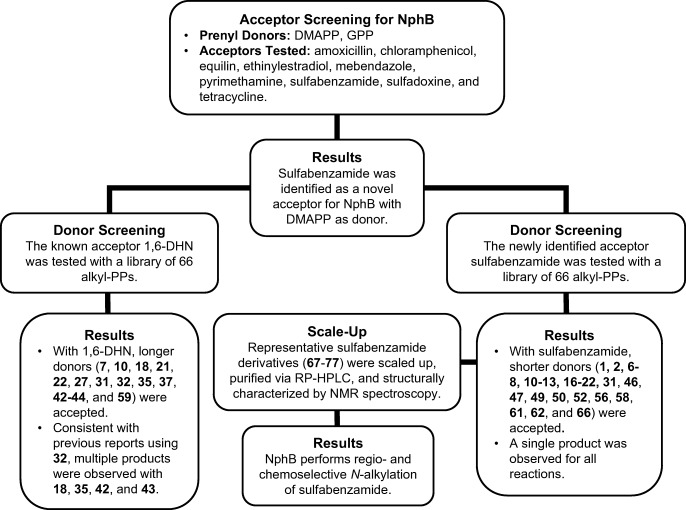
Flow chart summarizing the logical progression of the methods used in this study and the major results of each step

In summary, the present study established donor specificity profiles of NphB with the putative acceptor 1,6-DHN and the newly identified acceptor sulfabenzamide using a synthetic library of diverse alkyl-PPs (Fig. 7). The two profiles revealed not only the general donor promiscuity of NphB but also the dependence of this promiscuity on the identity of the acceptor substrate. Specifically, the donor profiles of 1,6-DHN and sulfabenzamide reflect the size difference between the two acceptors, which could imply similar behavior for other prenyltransferases with known acceptor promiscuity. Additionally, NMR characterization of representative sulfabenzamide derivatives generated in this work revealed the first reported instance of NphB-catalyzed *N*-alkylation. Taken together, the findings of the present study complement the known utility of NphB as a biocatalyst while simultaneously establishing its capabilities beyond geranyl transfer reactions.

## Electronic supplementary material

ESM 1 (PDF 4.54 mb)

HPLC chromatographs, HRMS and NMR table (PDF)
