# Supplementary material for: Acceptor substrate determines donor specificity of an aromatic prenyltransferase: expanding the biocatalytic potential of NphB
Source: Appl Microbiol Biotechnol. 2020 Mar 18;104(10):4383–95. doi: 10.1007/s00253-020-10529-8 (PMC7190591; doi:10.1007/s00253-020-10529-8)
Supplement: Supplementary file 1 — (PDF 4.54 mb) [file 253_2020_10529_MOESM1_ESM.pdf]

Applied Microbiology and Biotechnology

Supplementary Material

Acceptor Substrate Determines Donor Specificity of an Aromatic Prenyltransferase: Expanding the Biocatalytic Potential of NphB

Bryce P. Johnson[†,‡], Erin M. Scull[†,‡], Dustin A. Dimas[†], Tejaswi Bavineni [†], Chandrasekhar Bandari[†], Andrea L. Batchev[†], Eric D. Gardner[†], Susan L. Nimmo[†] and Shanteri Singh[†,*]

[†]Department of Chemistry and Biochemistry, University of Oklahoma, Stephenson Life Sciences Research Center, 101 Stephenson Parkway, Norman, OK 73019, USA.

[‡] These authors contributed equally.
[*] To whom correspondence should be addressed. Email: shanteri.singh@ou.edu

Table of contents

1. **Figure S1**. Amino acid sequence of recombinant His_6NphB S2
2. **Figure S2**. SDS-PAGE results for purification of recombinant His_6-NphB S3
3. **Figure S3**. RP-HPLC chromatograms of 1,6-DHN analytical-scale reactions S4
4. **Figure S4.** RP-HPLC chromatograms sulfabenzamide analytical-scale reactions S5-S6
5. **Table S1.** Yield of 1,6-DHN alkylated products in analytical-scale reactions S7
6. **Table S2.** Yield of analytical-scale sulfabenzamide reaction products S8
7. **Table S3.** HRMS data for 1,6-DHN alkylated products S9
8. **Table S4.** HRMS data for sulfabenzamide alkylated products S10
9. **Table S5.** NMR data for scaled-up, alkylated sulfabenzamide products S11-S13
10. Structural data and yields for novel alkyl pyrophosphates S14-S18
11. HRMS and NMR spectra of novel alkyl pyrophosphates S19-S126
12. HRMS spectra of alkylated 1,6-DHN products S127-S140
13. NMR spectra of commercial sulfabenzamide S141-S142
14. HRMS and NMR spectra of scaled-up, alkylated sulfabenzamide products S143-S175
15. HRMS spectra of analytical-scale, alkylated sulfabenzamide products S176-S201

A

ATGAGTGAAGCGGCGGATGTGGAACGTGTGTATGCGGCAATGGAAGAAGCGGCTGGTCTGCTGGGTGT
GGCGTGTGCTCGTGATAAAATCTATCCGCTGCTGAGCACCTTTCAGGATACGCTGGTTGAAGGCGGTT
CTGTGGTTGTCTTCAGCATGGCCTCTGGCCGCCATAGTACCGAACTGGATTTTAGTATTTCCGTTCCG
ACGTCCCACGGTGACCCGTACGCGACCGTGGTTGAAAAAGGCCTGTTTCCGGCCACGGGTCATCCGGT
GGATGACCTGCTGGCAGATACCCAAAAACACCTGCCGGTCAGCATGTTTGCTATTGACGGCGAAGTGA
CCGGCGGTTTCAAGAAAACCTATGCGTTTTTCCCGACCGATAACATGCCGGGTGTGGCCGAACTGTCA
GCAATCCCGTCGATGCCGCCGGCAGTTGCAGAAAATGCTGAACTGTTCGCGCGTTACGGCCTGGATAA
AGTTCAGATGACCTCAATGGACTATAAAAAACGCCAAGTCAACCTGTACTTTAGTGAACTGTCCGCCC
AGACCCTGGAAGCAGAATCGGTCCTGGCTCTGGTGCGTGAACTGGGCCTGCATGTCCCGAATGAACTG
GGTCTGAAATTTTGCAAACGCTCATTCTCGGTGTATCCGACCCTGAACTGGGAAACGGGCAAAATTGA
TCGTCTGTGTTTCGCAGTGATCTCCAATGACCCGACGCTGGTTCCGAGCTCTGATGAAGGTGACATCG
AAAAATTTCACAACTATGCAACCAAAGCTCCGTATGCGTACGTTGGCGAAAAACGTACGCTGGTCTAC
GGTCTGACCCTGAGCCCGAAAGAAGAATATTACAAACTGGGTGCGTATTACCACATTACGGACGTGCA
ACGCGGTCTGCTGAAAGCATTTGACTCTCTGGAAGATTGA

B

MGSSHHHHHHSSGLVPAGSHMSEAADVERVYAAMEEAAGLLGVACARDKIYPLLSTFQDTLVEGGSVV
VFSMASGRHSTELDFSISVPTSHGDPYATVVEKGLFPATGHPVDDLLADTQKHLPVSMFAIDGEVTGG
FKKTYAFFPTDNMPGVAELSAIPSMPPAVAENAELFARYGLDKVQMTSMDYKKRQVNLYFSELSAQTL
EAESVLALVRELGLHVPNELGLKFCKRSFSVYPTLNWETGKIDRLCFAVISNDPTLVPSSDEGDIEKF
HNYATKAPYAYVGEKRTLVYGLTLSPKEEYYKLGAYYHITDVQRGLLKAFDSLED

Fig. S1. (**A**) Sequence of the codon-optimized synthetic gene for NphB used in this study. (**B**) Amino acid sequence of the recombinant His_6-NphB used in this study. The His_6-tag and spacer are shown in bold and were supplied by the pET-28a vector.

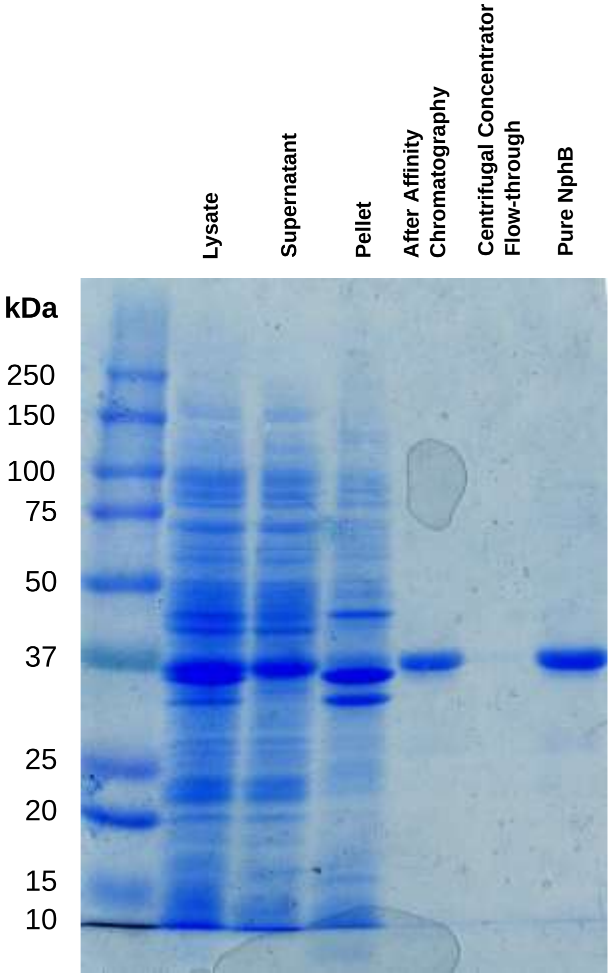

Fig. S2. SDS-PAGE of recombinant His_6-NphB purification by Ni-NTA affinity chromatography. The gel was composed of 12% SDS, and the electrophoresis buffer consisted of 25 mM Tris pH 8.3, 250 mM glycine, and 0.001% SDS (w/v).

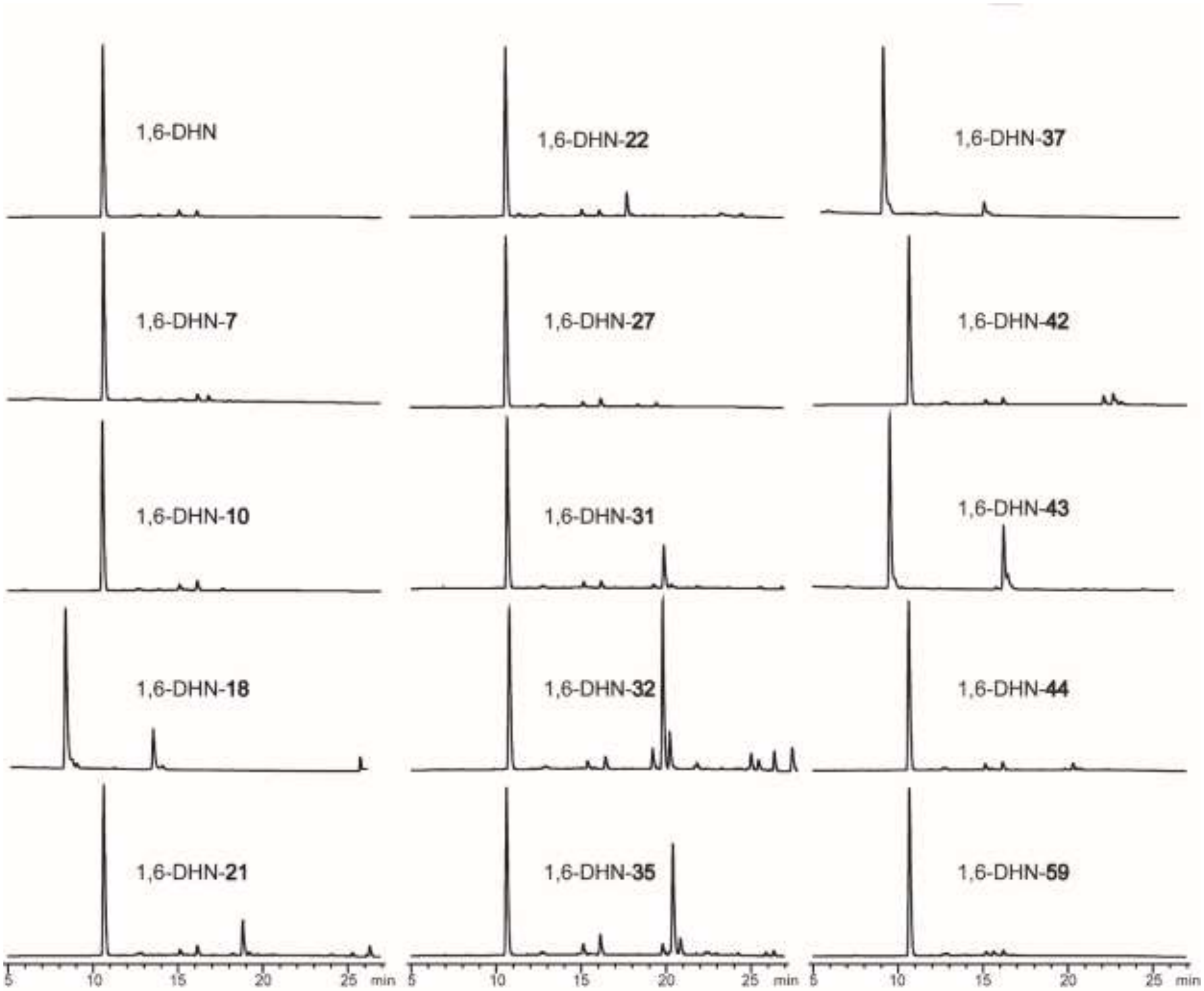

Fig. S3. RP-HPLC chromatograms of incubation mixtures of 1,6-DHN with pyrophosphate analogues leading to alkylated 1,6-DHN derivatives. Each product is named using "1,6-DHN" and the number of the corresponding alkyl-PP. Reactions were performed in a total volume of 20 μL with 6 μM purified NphB in 25 mM Tris buffer pH 8.0, 5 mM $MgCl_2$, 50 mM KCl and were incubated at 35 °C for 16 h. Chromatograms for 1,6-DHN-**18**, 1,6-DHN-**37**, and 1,6-DHN-**43** were obtained using slightly different gradients from the other products.

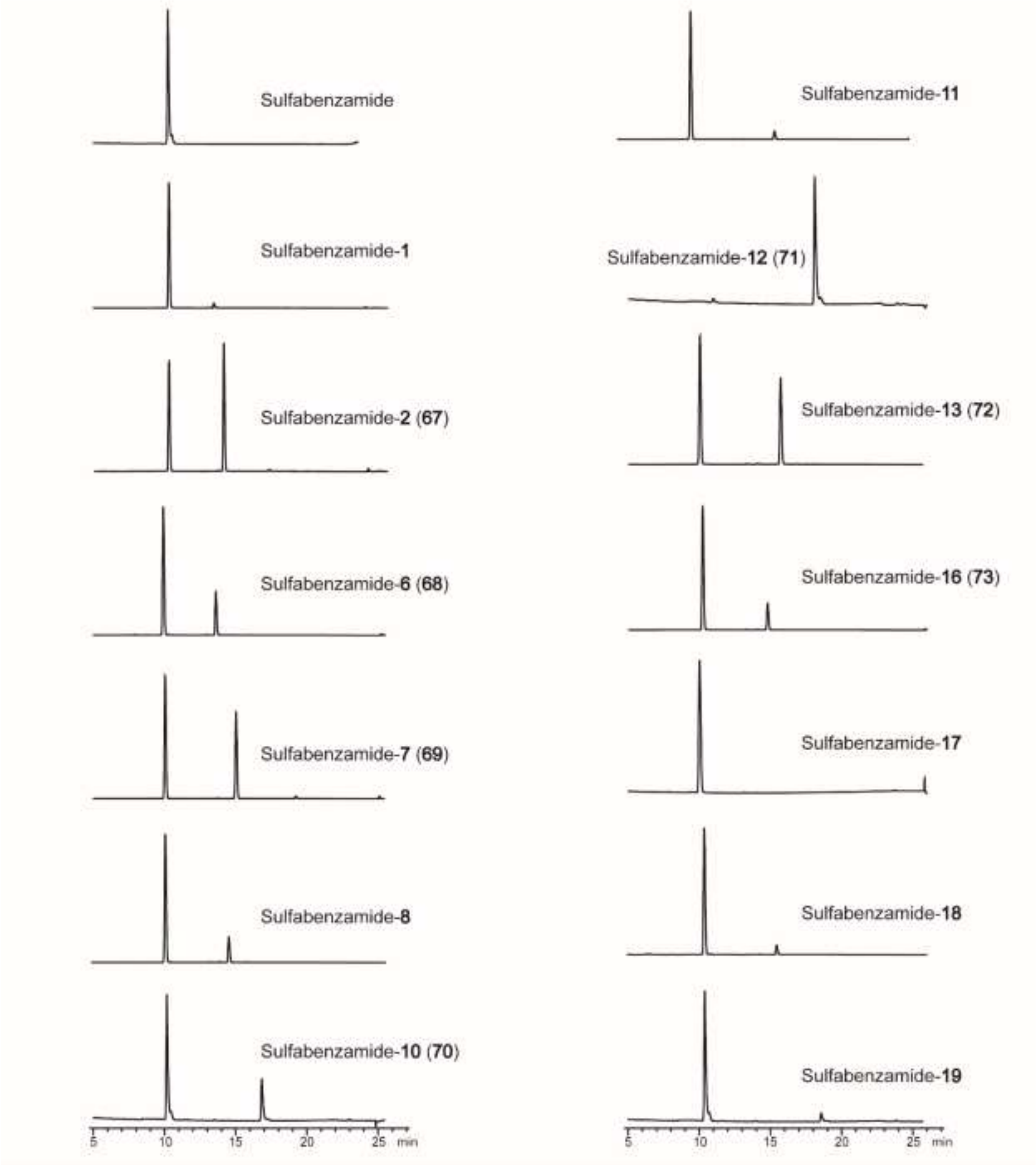

Sulfabenzamide
Sulfabenzamide-1
Sulfabenzamide-2 (67)
Sulfabenzamide-6 (68)
Sulfabenzamide-7 (69)
Sulfabenzamide-8
Sulfabenzamide-10 (70)
Sulfabenzamide-11
Sulfabenzamide-12 (71)
Sulfabenzamide-13 (72)
Sulfabenzamide-16 (73)
Sulfabenzamide-17
Sulfabenzamide-18
Sulfabenzamide-19
5
10
15
20
25
min

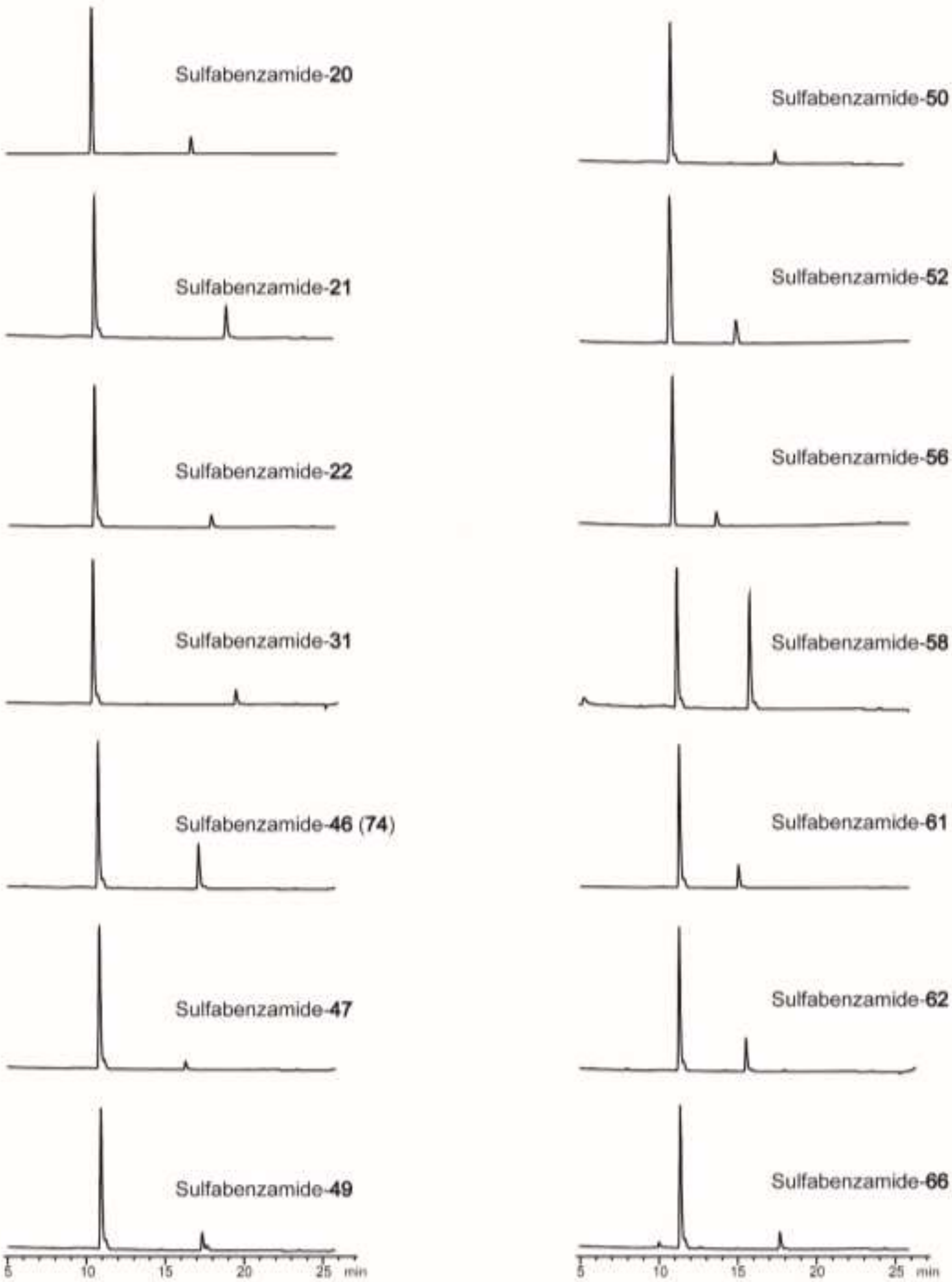

Fig. S4. RP-HPLC chromatograms of incubation mixtures of sulfabenzamide with pyrophosphate analogues leading to alkylated sulfabenzamide derivatives. Each product is named using “sulfabenzamide” and the number of the corresponding alkyl-PP. Reactions were performed in a total volume of 20 µL with 6 µM purified NphB in 25 mM Tris buffer pH 8.0, 5 mM $MgCl_2$, 50 mM KCl and were incubated at 35 °C for 16 h.

Table S1. Percent yield for each NphB-catalyzed analytical-scale reaction of 1,6-DHN accounting for all regioisomers (mean±SD, n=2)

Enzyme Product	Percent Yield (%)
1,6-DHN-7	2.95 ± 0.57
1,6-DHN-10	0.91 ± 0.81
1,6-DHN-18	19.4 ± 2.6
1,6-DHN-21	23.1 ± 1.0
1,6-DHN-22	15.4 ± 2.2
1,6-DHN-27	3.05 ± 0.20
1,6-DHN-31	22.22 ± 0.87
1,6-DHN-32	62.4 ± 6.4
1,6-DHN-35	44.1 ± 4.4
1,6-DHN-37	6.6 ± 3.3
1,6-DHN-42	13.55 ± 0.73
1,6-DHN-43	37.1 ± 6.2
1,6-DHN-44	4.7 ± 1.4
1,6-DHN-59	2.40 ± 0.49

Table S2. Percent yield for each NphB-catalyzed analytical-scale reaction of sulfabenzamide (mean±SD, n=2)

Enzyme Product	Percent Yield (%)
Sulfabenzamide-1	3.03 ± 0.78
Sulfabenzamide-2 (67)	58.89 ± 0.93
Sulfabenzamide-6 (68)	28.9 ± 6.1
Sulfabenzamide-7 (69)	43.9 ± 2.3
Sulfabenzamide-8	18.2 ± 5.9
Sulfabenzamide-10 (70)	28.5 ± 1.6
Sulfabenzamide-11	10.0 ± 5.7
Sulfabenzamide-12 (71)	100 ± 0
Sulfabenzamide-13 (72)	37.8 ± 3.9
Sulfabenzamide-16 (73)	20.3 ± 4.0
Sulfabenzamide-17	2.3 ± 2.9
Sulfabenzamide-18	6.95 ± 0.10
Sulfabenzamide-19	6.92 ± 0.79
Sulfabenzamide-20	16.4 ± 8.4
Sulfabenzamide-21	22.2 ± 2.3
Sulfabenzamide-22	9.5 ± 1.5
Sulfabenzamide-31	13.3 ± 3.8
Sulfabenzamide-46 (74)	26.3 ± 2.5
Sulfabenzamide-47	5.63 ± 0.16
Sulfabenzamide-49	14.73 ± 0.54
Sulfabenzamide-50	8.78 ± 0.16
Sulfabenzamide-52	13.0 ± 2.8
Sulfabenzamide-56	14.7 ± 5.3
Sulfabenzamide-58 (75)	44.7 ± 1.8
Sulfabenzamide-61 (76)	13.4 ± 2.3
Sulfabenzamide-62 (77)	18.7 ± 1.9
Sulfabenzamide-66	9.4 ± 2.5

Table S3: Summary of HRMS data for 1,6-DHN analogues from NphB-catalyzed alkylation reactions with synthetic alkyl-PP analogs

Enzyme Product	Chemical Formula	Calculated Mass (Da)	Observed Mass (Da)	Error(ppm)
1,6-DHN-7	$C_{16}H_{17}O_2$ $[M-H]^-$	241.1234	241.1237	1.2
1,6-DHN-10	$C_{17}H_{19}O_2$ $[M-H]^-$	255.1390	255.1394	1.6
1,6-DHN-18	$C_{17}H_{17}O_2$ $[M-H]^-$	253.1234	253.1238	1.6
1,6-DHN-21	$C_{18}H_{21}O_2$ $[M-H]^-$	269.1547	269.1548	0.4
1,6-DHN-22	$C_{18}H_{19}O_2$ $[M-H]^-$	267.1390	267.1391	0.4
1,6-DHN-27	$C_{18}H_{17}O_2$ $[M-H]^-$	265.1234	265.1244	3.8
1,6-DHN-31	$C_{19}H_{23}O_2$ $[M-H]^-$	283.1703	283.2645	334*
1,6-DHN-32	$C_{20}H_{23}O_2$ $[M-H]^-$	295.1703	295.1704	0.4
1,6-DHN-35	$C_{21}H_{25}O_2$ $[M-H]^-$	309.1860	309.1859	0.3
1,6-DHN-37	$C_{21}H_{17}O_2$ $[M-H]^-$	301.1234	301.1235	0.3
1,6-DHN-42	$C_{25}H_{31}O_2$ $[M-H]^-$	363.2329	363.2339	2.8
1,6-DHN-43	$C_{23}H_{25}O_3$ $[M-H]^-$	349.1809	349.1801	2.3
1,6-DHN-44	$C_{27}H_{29}O_3$ $[M-H]^-$	401.2122	401.2132	2.5
1,6-DHN-59	$C_{20}H_{15}O_3$ $[[M-H]^-$	303.1027	303.1031	1.3

*The value of this error means the mass is considered low resolution.

Table S4: Summary of HRMS data for sulfabenzamide analogues from NphB-catalyzed alkylation reactions with synthetic alkyl-PP analogs.

Enzyme Product	Chemical Formula	Calculated Mass (Da)	Observed Mass (Da)	Error (ppm)
Sulfabenzamide-1	$C_{17}H_{19}N_2O_3S$ [M+H]$^+$	331.1116	331.1120	1.2
Sulfabenzamide-2 (67)	$C_{18}H_{21}N_2O_3S$ [M+H]$^+$	345.1272	345.1276	1.2
Sulfabenzamide-6 (68)	$C_{18}H_{19}N_2O_3S$ [M+H]$^+$	343.1116	343.1118	0.6
Sulfabenzamide-7 (69)	$C_{19}H_{23}N_2O_3S$ [M+H]$^+$	359.1429	359.1442	3.6
Sulfabenzamide-8	$C_{19}H_{21}N_2O_3S$ [M+H]$^+$	357.1273	357.1270	0.8
Sulfabenzamide-10 (70)	$C_{20}H_{24}N_2O_3SNa$ [M+Na]$^+$	395.1406	395.1425	4.8
Sulfabenzamide-11	$C_{20}H_{25}N_2O_3S$ [M+H]$^+$	373.1586	373.1592	1.6
Sulfabenzamide-12 (71)	$C_{20}H_{22}N_2O_3SNa$ [M+Na]$^+$	393.1249	393.1266	4.3
Sulfabenzamide-13 (72)	$C_{20}H_{23}N_2O_3S$ [M+H]$^+$	371.1429	371.1435	1.6
Sulfabenzamide-16 (73)	$C_{19}H_{21}N_2O_3S$ [M+H]$^+$	357.1273	357.1283	2.8
Sulfabenzamide-17	$C_{20}H_{23}N_2O_3S$ [M+H]$^+$	371.1429	371.1435	1.6
Sulfabenzamide-18	$C_{20}H_{23}N_2O_3S$ [M+H]$^+$	371.1429	371.1431	0.5
Sulfabenzamide-19	$C_{21}H_{26}N_2O_3SNa$ [M+Na]$^+$	409.1562	409.1576	3.4
Sulfabenzamide-20	$C_{21}H_{25}N_2O_3S$ [M+H]$^+$	385.1580	385.1590	2.6
Sulfabenzamide-21	$C_{21}H_{26}N_2O_3SNa$ [M+Na]$^+$	409.1562	409.1577	3.7
Sulfabenzamide-22	$C_{21}H_{24}N_2O_3SNa$ [M+Na]$^+$	407.1406	407.1420	3.4
Sulfabenzamide-31	$C_{22}H_{28}N_2O_3SNa$ [M+Na]$^+$	423.1719	423.1733	3.3
Sulfabenzamide-46 (74)	$C_{21}H_{20}N_2O_3SNa$ [M+Na]$^+$	403.1093	403.1106	3.2
Sulfabenzamide-47	$C_{20}H_{17}FN_2O_3SNa$ [M+Na]$^+$	407.0842	407.0858	3.9
Sulfabenzamide-49	$C_{20}H_{17}ClN_2O_3SNa$ [M+Na]$^+$	423.0546	423.0553	1.7
Sulfabenzamide-50	$C_{20}H_{17}BrN_2O_3SNa$ [M+Na]$^+$	467.0042	467.0060	3.9
Sulfabenzamide-52	$C_{21}H_{20}N_2O_3SNa$ [M+Na]$^+$	419.1042	419.1029	3.1
Sulfabenzamide-56	$C_{22}H_{22}N_2O_5SNa$ [M+Na]$^+$	449.1147	449.1166	4.2
Sulfabenzamide-58 (75)	$C_{21}H_{18}N_2O_5SNa$ [M+Na]$^+$	433.0834	433.0838	0.9
Sulfabenzamide-61 (76)	$C_{18}H_{16}N_2O_4SNa$ [M+Na]$^+$	379.0729	379.0733	1.1
Sulfabenzamide-62 (77)	$C_{18}H_{16}N_2O_3S_2Na$ [M+Na]$^+$	395.0500	395.0499	0.3
Sulfabenzamide-66	$C_{22}H_{18}N_2O_3S_2Na$ [M+Na]$^+$	445.0657	445.0665	1.8

Table S5. Summary of NMR data for sulfabenzamide analogs scaled up and purified by semi-preparative RP-HPLC

**Compound**	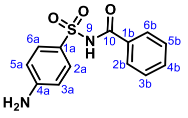 **Sulfabenzamide**		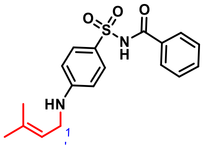 **67**	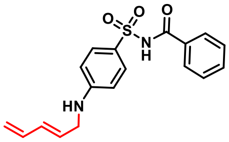 **68**	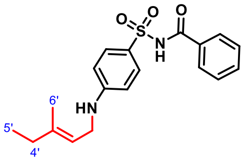 **69**
**Position**	**δ_H, multi (J)**	**δ_C**	**δ_H, multi (J)**	**δ_H, multi (J)**	**δ_H, multi (J)**
**NH**	12.07, s		8.13, s	8.14, s	8.13, s
**NH**	6.15, s (NH_2)		6.56, s	6.56, s	6.52, s
**1a**					
**2a**	7.63, d (8.8)	130.6	7.59, d (8.4)	7.54, d (8.4)	7.62, d (8.7)
**3a**	6.61, d (8.8)	112.7	6.52, d (8.4)	6.49, d (8.4)	6.57, d (8.7)
**4a**					
**5a**	6.61, d (8.8)	112.7	6.52, d (8.4)	6.49, d (8.4)	6.57, d (8.7)
**6a**	7.63, d (8.8)	130.6	7.59, d (8.4)	7.54, d (8.4)	7.62, d (8.7)
**1b**					
**2b**	7.83, dd (8.4, 1.3)	128.7	7.80, dd (8.4, 1.3)	7.84, dd (8.3, 1.6)	7.84, d (7.1)
**3b**	7.47, dd (8.4, 7.4)	128.9	7.36, m	7.30, m	7.40, t (7.1)
**4b**	7.60, tt (7.4, 1.3)	133.5	7.44, m	7.34, m	7.50, m
**5b**	7.47, dd (8.4, 7.4)	128.9	7.36, m	7.30, m	7.40, t (7.1)
**6b**	7.83, dd (8.4, 1.3)	128.7	7.80, dd (8.4, 1.3)	7.84, dd (8.3, 1.6)	7.84, d (7.1)
**1′**			3.64, dd (6.5, 5.9)	3.75, t (5.6)	3.67, t (5.7)
**2′**			5.22, t (6.5)	5.79, dt (15,3, 5.6)	5.22, t (5.7)
**3′**				6.22, dd (15.3, 10.5)	
**4′**			1.68, s	6.35, dt (16.9, 10.5)	1.99, q (7.8)
**5′**			1.69, s	5.16, dd (16.9, 1.4)<br>5.02, dd (10.5, 1.4)	0.96, t (7.8)
**6′**					1.68, s
**Solvent**	DMSO-d6		DMSO-d6	DMSO-d6	DMSO-d6

Compound	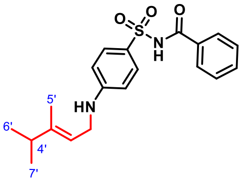 70		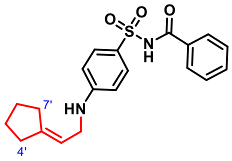 71		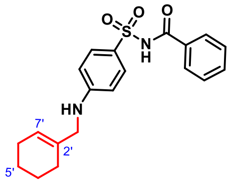 72	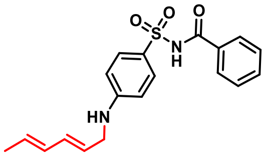 73
**Position**	**δ$_H$, multi (J)**	**δ$_C$**	**δ$_H$, multi (J)**	**δ$_C$**	**δ$_H$, multi (J)**	**δ$_H$, multi (J)**
**NH**			8.14, brs		8.12, brs	8.12, brs
**NH**			6.36, brs		6.54, s	6.54, s
**1a**		124.09		129.42		
**2a**	7.93, d (8.9)	127.71	7.59, d (8.8)	129.45	7.60, d (8.4)	7.63, d (8.7)
**3a**	6.60, d (8.9)	111.44	6.52, d (8.8)	110.8	6.56, d (8.4)	6.59, d (8.7)
**4a**		152.8		151.8		
**5a**	6.60, d (8.9)	111.44	6.52, d (8.8)	110.8	6.56, d (8.4)	6.59, d (8.7)
**6a**	7.93, d (8.9)	127.71	7.59, d (8.8)	129.49	7.60, d (8.4)	7.63, d(8.7)
**1b**		124.09		136.76		
**2b**	7.79, dd (8.4,1.3)	130.97	7.86, d (8.0)	128.24	7.85, d (7.7)	7.84, dd (8.3, 1.4)
**3b**	7.44, t, (8.4, 7.4)	128.9	7.36 t, (7.6)	128.24	7.39, t (7.6)	7.42, dd (8.3, 7.5)
**4b**	7.55, tt (7.5, 1.3)	133.22	7.44, t (7.3)	131.49	7.49, m	7.53, tt (7.5, 1.4)
**5b**	7.44, t, (8.4, 7.4)	128.9	7.36 t, (7.6)	128.21	7.39, t (7.6)	7.42, dd (8.3, 7.5)
**6b**	7.79, dd (8.4,1.3)	130.97	7.86, d (8.0)	128.7	7.85, d (7.7)	7.84, dd (8.3, 1.4)
**1′**	3.75, d (6.6)	41.24	3.61, d (6.0)	42.35	3.57, d (5.7)	3.75, td(5.7, 1.5)
**2′**	5.30, t (6.7)	117.84	5.34, p (4.1, 2.1)			5.58, dt (15.1, 5.7)
**3′**		146.11		145.5	1.94, m	6.17, ddt (15.1, 10.5, 1.5)
**4′**	2.28, hept (7.3,7.0)	36.71	2.24, m	28.89	1.57, m	6.05, ddd(15.1, 10.5, 1.5)
**5′**	1.68, s	14.23	1.64, p (7.0, 6.9)	26.39	1.50, m	5.63, dq (15.1, 6.6)
**6′**	1.02, d (6.9)	21.24	1.64, p (7.1, 7.0)	26.24	1.95, m	1.69, dd (6.6, 1.5)
**7′**	1.02, d (6.9)	21.24	2.22, m	33.66	5.58, s	
**10**		164.14				
**Solvent**	$CDCl_3$		DMSO-d6		DMSO-d6	DMSO-d6

Compound	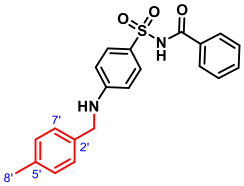 74	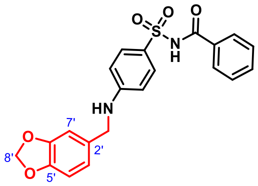 75		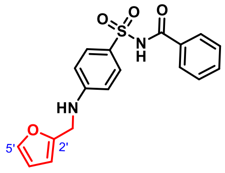 76		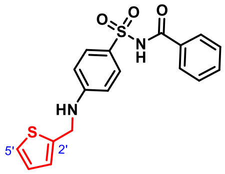 77	
**Position**	**δ$_H$, multi (J)**	**δ$_H$, multi (J)**	**δ$_C$**	**δ$_H$, multi (J)**	**δ$_C$**	**δ$_H$, multi (J)**	**δ$_C$**
**NH**	8.41, s	8.16, brs		8.29, brs		8.3, brs	
**NH**	6.53, t (5.8)	6.64, brs		6.45, t (6.0)		6.62, t (6.0)	
**1a**			132.16		133.9		134.1
**2a**	7.49, d (8.5)	7.53, dd (8.8)	128.96	7.53, d (8.7)	128.65	7.53, d (8.7)	128.68
**3a**	6.46, d (8.5)	6.51, d (8.9)	110.93	6.56, d (8.8)	110.94	6.55, d (8.9)	111.02
**4a**			150.58		149.97		150.03
**5a**	6.46, d (8.5)	6.51, d (8.9)	110.93	6.56, d (8.8)	110.94	6.55, d (8.7)	111.02
**6a**	749, d (8.5)	7.53, d (8.8)	128.96	7.53, d (8.7)	128.65	7.53, d (8.7)	128.68
**1b**			138.85		140.79		139.64
**2b**	7.85, d (7.1)	7.86, dd (8.4,1.3)	128.71	7.87, dd (7.9, 1.4)	128.69	7.87, dd (8.4,1.4)	128.74
**3b**	7.26, dd (7.1, 6.6)	7.30 t, (7.6,7.4)	127.9	7.27, dd (8.2, 6.5)	127.73	7.27, tt (7.2, 1.4)	127.72
**4b**	7.31, t (6.6)	7.36, t (7.5, 7.5)	130.47	7.33, dd (8.2, 6.5)	129.94	7.33, tt (7.4, 1.4)	129.97
**5b**	7.26, dd (7.1, 6.6)	7.30 t, (7.6,7.4)	127.9	7.27, dd (8.2, 6.5)	127.73	7.27, tt (7.2, 1.4)	127.72
**6b**	7.85, d (7.1)	7.86, dd (8.4,1.3)	128.71	7.87, dd (7.9, 1.4)	128.69	7.87, dd (8.4,1.4)	128.74
**1′**	4.22, d (5.8)	4.18, d (5.1)	46.26	4.25, d (5.8)	40.07	4.45, d (4.45)	42.04
**2′**			134.13		153.52		
**3′**	7.21, d (7.7)	6.89, s	108.08	6.29, d (3.2)	107.36	7.03, dd (3.4, 1.1)	125.29
**4′**	7.1, d (7.7)	6.82, m	108.5	6.36, dd (3.2, 1.8)	110.76	6.94, dd (5.0,3.4)	127.19
**5′**			146.43	7.02, d (2.5)	121.08	7.34, dd (5.1,1.3)	125
**6′**	7.1, d (7.7)		147.68				
**7′**	7.21, d (7.7)	6.83, m	120.63				
**8′**	2.24, s	5.95, s	146.43				
**10**			169.14				
**Solvent**	DMSO-d6	DMSO-d6		DMSO-d6		DMSO-d6	

Structural Data and Yields of Novel Alkyl Pyrophosphates

*(*E*)-2-methylbut-2-en-1-yl pyrophosphate (**3**)*
The title product was obtained as a solid (31.6%) from (*E*)-2-methylbut-2-en-1-ol. TLC (^{i}PrOH: NH_4OH: H_2O 7:2:1 v/v): R_f = 0.31; ^{1}H NMR (400 MHz, D_2O) δ 5.73 – 5.57 (m, 1H), 4.34 (d, *J* = 6.4 Hz, 2H), 1.70 (s, 3H), 1.66 – 1.62 (m, 3H). ^{31}P NMR (243 MHz, D_2O) δ -10.90 (the two signals are overalpping). HRMS-ESI: Calc for $C_5H_{11}O_7P_2$ [M-H]$^-$: 244.99799; Found: 244.9956.

*(*E*)-3-methylpenta-2,4-dien-1-yl pyrophosphate (**8**)*
The title product was obtained as a white solid (30.7%) from (*E*)-3-methylpenta-2,4-dien-1-ol. TLC (^{i}PrOH: NH_4OH: H_2O 7:2:1 v/v): R_f = 0.39; ^{1}H NMR (400 MHz, D_2O) δ 6.50 (dd, *J* = 17.5, 10.7 Hz, 1H), 5.74 (t, *J* = 7.0 Hz, 1H), 5.34 (d, *J* = 17.5 Hz, 1H), 5.15 (d, *J* = 10.7 Hz, 1H), 4.61 (t, *J* = 7.0 Hz, 2H), 1.83 (s, 3H). ^{31}P NMR (162 MHz, D_2O) δ -7.28 (d, *J* = 21.6 Hz), -10.38 (d, *J* = 21.6 Hz). HRMS-ESI: Calc for $C_6H_{11}O_7P_2$ [M-H]$^-$: 256.99799; Found: 256.9987.

*(*E*)-3,4-dimethylpent-2-en-1-yl pyrophosphate (**10**)*
The title product was obtained as a solid (28.3%) from (*E*)-3,4-dimethylpent-2-en-1-ol. TLC (^{i}PrOH: NH_4OH: H_2O 7:2:1 v/v): R_f = 0.32; ^{1}H NMR (400 MHz, D_2O) δ 5.45 (t, *J* = 7.1 Hz, 1H), 4.46 (t, *J* = 6.6 Hz, 2H), 2.28 (h, *J* = 6.9 Hz, 1H), 1.67 (s, 3H), 0.98 (d, *J* = 6.8 Hz, 6H). ^{31}P NMR (162 MHz, D_2O) δ -8.18 (d, *J* = 21.5 Hz), -10.58 (d, *J* = 21.5 Hz). MS-ESI: Calc for $C_7H_{15}O_7P_2$ [M-H]$^-$: 273.02909; Found: 273.0304.

*(*E*)-pent-2-en-4-yn-1-yl pyrophosphate (**14**)*
The title product was obtained as a brown solid (27.5%) from (*E*)-pent-2-en-4-yn-1-ol. TLC (^{i}PrOH: NH_4OH: H_2O 7:2:1 v/v): R_f = 0.35; ^{1}H NMR (400 MHz, D_2O) δ 6.40 (dd, *J* = 13.2, 8.0 Hz, 1H), 5.86 (d, *J* = 15.9 Hz, 1H), 4.52 (t, *J* = 6.5 Hz, 2H), 3.31 (s, 1H). ^{31}P NMR (162 MHz, D_2O) δ -7.69 (d, *J* = 21.7 Hz), -10.83 (d, *J* = 22.1 Hz). HRMS-ESI: Calc for $C_5H_7O_7P_2$ [M-H]$^-$: 240.96669; Found: 240.9658.

*(*E*)-2-methylpent-2-en-4-yn-1-yl pyrophosphate (**15**)*
The title product was obtained as a brown solid (25.5%) from (*E*)-2-methylpent-2-en-4-yn-1-ol. TLC (^{i}PrOH: NH_4OH: H_2O 7:2:1 v/v): R_f = 0.36; ^{1}H NMR (400 MHz, D_2O) δ 5.65 (s, 1H), 4.44 (d, *J* = 7.4 Hz, 2H), 3.51 (d, *J* = 2.3 Hz, 1H), 1.92 (s, 3H).^{31}P NMR (162 MHz, D_2O) δ -8.11 (d, *J* = 21.1 Hz), -10.86 (d, *J* = 21.5 Hz). HRMS-ESI: Calc for $C_6H_9O_7P_2$ [M-H]$^-$: 254.98234; Found: 254.9830.

*(2*E*,4*E*)-hexa-2,4-dien-1-yl pyrophosphate (**16**)*
The title product was obtained as a solid (29.7%) from (2*E*,4*E*)-hexa-2,4-dien-1-ol. TLC (^{i}PrOH: NH_4OH: H_2O 7:2:1 v/v): R_f = 0.37; ^{1}H NMR (400 MHz, D_2O) δ 6.54 – 5.99 (m, 2H), 5.83 (dt, *J* = 14.4, 6.6 Hz, 1H), 5.78 – 5.62 (m, 1H), 4.41 (t, *J* = 6.9 Hz, 2H), 1.71 (d, *J* = 6.9 Hz, 3H). ^{31}P NMR (162 MHz, Deuterium Oxide) δ -7.50 (d, *J* = 21.6 Hz), -10.57 (d, *J* = 21.5 Hz). HRMS-ESI: Calc for $C_6H_{11}O_7P_2$ [M-H]$^-$: 256.99799; Found: 256.9982.

*(*E*)-3-ethylhex-2-en-1-yl pyrophosphate (**19**)*
The title product was obtained as an ivory solid (30.4%) from (*E*)-3-ethylhex-2-en-1-ol. TLC (^{i}PrOH: NH_4OH: H_2O 7:2:1 v/v): R_f = 0.36; ^{1}H NMR (400 MHz, D_2O) δ 5.45 (dt, *J* = 15.4, 7.2 Hz, 0H), 4.51 (q, *J* = 6.0 Hz, 1H), 2.40 – 1.77 (m, 1H), 1.44 (dq, *J* = 14.2, 7.2 Hz, 1H), 1.00 (dt, *J* = 9.6, 7.5 Hz, 1H), 0.94 – 0.84 (m, 1H). ^{31}P NMR (162 MHz, D_2O) δ -9.02 (d, *J* = 25.0 Hz), -10.66 (d, *J* = 21.3 Hz). MS-ESI: Calc for $C_8H_1O_7P_2$ [M-H]$^-$: 287.04494; Found: 287.0466.

*(*E*)-3-methylhept-2-en-1-yl pyrophosphate (**21**)*
The title product was obtained as an ivory solid (29.8%) from (*E*)-3-methylhept-2-en-1-ol. TLC (^{i}PrOH: NH_4OH: H_2O 7:2:1 v/v): R_f = 0.35; ^{1}H NMR (400 MHz, D_2O) δ 5.46 (t, *J* = 7.3 Hz, 1H), 4.47 (t, *J* = 6.7 Hz, 2H), 2.07 (t, *J* = 7.6 Hz, 2H), 1.71 (s, 3H), 1.57 – 1.09 (m, 4H), 0.88 (t, *J* = 7.4 Hz, 3H). ^{31}P NMR (162 MHz, D_2O) δ -8.08 (d, *J* = 21.5 Hz), -10.51 (d, *J* = 21.5 Hz). HRMS-ESI: Calc for $C_8H_1O_7P_2$ [M-H]$^-$: 287.04494; Found: 287.0459.

*(E)-3-methylhepta-2,6-dien-1-yl pyrophosphate (**22**)*
The title product was obtained as an off-white solid (32.2%) from (*E*)-3-methylhepta-2,6-dien-1-ol. TLC (^{i}PrOH: NH_4OH: H_2O 7:2:1 v/v): R_f = 0.38; ^{1}H NMR (400 MHz, D_2O) δ 5.91 (ddt, *J* = 17.0, 11.7, 6.0 Hz, 1H), 5.47 (d, *J* = 6.1 Hz, 1H), 5.10 (d, *J* = 17.2 Hz, 1H), 5.01 (d, *J* = 10.1 Hz, 1H), 4.50 (t, *J* = 6.9 Hz, 2H), 2.21 (dd, *J* = 15.2, 8.3 Hz, 4H), 1.73 (s, 3H). ^{31}P NMR (162 MHz, D_2O) δ -10.13 (d, *J* = 21.4 Hz), -10.76 (d, *J* = 20.7 Hz). HRMS-ESI: Calc for $C_8H_{15}O_7P_2$ $[M-H]^-$: 285.02929; Found: 285.0282.

*(E)-3,6-dimethylhept-2-en-1-yl pyrophosphate (**23**)*
The title product was obtained as an ivory solid (31.2%) from (*E*)-3,6-dimethylhept-2-en-1-ol. TLC (^{i}PrOH: NH_4OH: H_2O 7:2:1 v/v): R_f = 0.36; ^{1}H NMR (300 MHz, D_2O) δ 5.42 (d, *J* = 7.7 Hz, 1H), 4.48 (t, *J* = 6.8 Hz, 2H), 4.30 (dt, *J* = 14.6, 7.4 Hz, 1H), 2.04 (t, *J* = 8.1 Hz, 2H), 1.89 – 1.60 (m, 3H), 1.51 (dt, *J* = 13.4, 6.2 Hz, 5H), 1.37 – 1.10 (m, 6H). ^{31}P NMR (122 MHz, D_2O) δ -10.68 (d, *J* = 19.3 Hz), -11.56 (d, *J* = 24.5 Hz). HRMS-ESI: Calc for $C_9H_{19}O_7P_2$ $[M-H]^-$: 301.06059; Found: 301.0612.

*(E)-3-methylhept-2-en-6-yn-1-yl pyrophosphate (**27**)*
The title product was obtained as a brown solid (25.8%) from (*E*)-3-methylhept-2-en-6-yn-1-ol. TLC (^{i}PrOH: NH_4OH: H_2O 7:2:1 v/v): R_f = 0.39; ^{1}H NMR (400 MHz, D_2O) δ 5.53 (t, *J* = 7.1 Hz, 1H), 4.58 – 4.33 (m, 2H), 2.43 – 2.34 (m, 2H), 2.29 (t, *J* = 7.3 Hz, 2H), 2.00 (s, 1H), 1.74 (s, 3H). ^{31}P NMR (162 MHz, D_2O) δ -7.42 (d, *J* = 22.9 Hz), -10.60 (d, *J* = 27.9, 20.5 Hz). Calc for $C_8H_{13}O_7P_2$ $[M-H]^-$: 283.01364; Found: 283.0171.

*(E)-4-azidobut-2-en-1-yl pyrophosphate (**28**)*
The title product was obtained as a brown solid (29.7%) from (*E*)-4-azidobut-2-en-1-ol. TLC (^{i}PrOH: NH_4OH: H_2O 7:2:1 v/v): R_f = 0.38; ^{1}H NMR (400 MHz, D_2O) δ 6.10 – 5.72 (m, 2H), 4.53 – 4.34 (m, 2H), 3.83 (d, *J* = 5.9 Hz, 2H). ^{31}P NMR (162 MHz, D_2O) δ -7.64 (d, *J* = 22.9 Hz), -10.82 (d, *J* = 22.3 Hz). HRMS-ESI: Calc for $C_4H_8N_3O_7P_2$ $[M-H]^-$: 271.98374; Found: 271.9839.

*(E)-4-azido-3-methylbut-2-en-1-yl pyrophosphate (**29**)*
The title product was obtained as a brown solid (24.7%) from (*E*)-4-azido-3-methylbut-2-en-1-ol. TLC (^{i}PrOH: NH_4OH: H_2O 7:2:1 v/v): R_f = 0.37; ^{1}H NMR (300 MHz, D_2O) δ 6.14 (dt, *J* = 1.7, 0.9 Hz, 1H), 3.94 (q, *J* = 6.8 Hz, 2H), 2.35 (t, *J* = 6.8 Hz, 2H), 1.67 (d, *J* = 1.5 Hz, 3H). ^{31}P NMR (122 MHz, D_2O) δ -7.60 (d, *J* = 21.6 Hz), -10.81 (d, *J* = 21.9 Hz). HRMS-ESI: Calc for $C_5H_{10}N_3O_7P_2$ $[M-H]^-$: 285.99939; Found: 285.9984.

*tri(l^5-azaneyl) ((3-azidocyclohex-1-en-1-yl)methyl) pyrophosphate (**30**)*
The title product was obtained as a brown solid (25.7%) from (3-azidocyclohex-1-en-1-yl)methanol. TLC (^{i}PrOH: NH_4OH: H_2O 7:2:1 v/v): R_f = 0.34; ^{1}H NMR (400 MHz, D_2O) δ 5.66 (s, 1H), 4.20 (d, *J* = 6.8 Hz, 2H), 3.91 (s, 1H), 2.02 – 1.77 (m, 2H), 1.60 – 1.40 (m, 4H). ^{31}P NMR (162 MHz, D_2O) δ -7.38 (d, *J* = 22.5 Hz), -10.87 (d, *J* = 22.3 Hz). HRMS-ESI: Calc for $C_7H_{12}N_3O_7P_2$ $[M-H]^-$: 312.01504; Found:312.0155.

*(E)-3-methyloct-2-en-1-yl pyrophosphate (**31**)*
The title product was obtained as an ivory solid (32.9%) from (*E*)-3-methyloct-2-en-1-ol. TLC (^{i}PrOH: NH_4OH: H_2O 7:2:1 v/v): R_f = 0.35; ^{1}H NMR (300 MHz, D_2O) δ 5.42 (t, *J* = 7.2 Hz, 1H), 4.44 (t, *J* = 6.6 Hz, 2H), 4.33 – 4.18 (m, 2H), 2.01 (d, *J* = 7.6 Hz, 2H), 1.72 (s, 1H), 1.67 (s, 2H), 1.54 (d, *J* = 6.0 Hz, 5H), 1.40 (ddd, *J* = 22.1, 13.8, 6.8 Hz, 3H). ^{31}P NMR (122 MHz, D_2O) δ -8.05 (d, *J* = 21.9 Hz), -8.80 (d, *J* = 20.6 Hz), -10.62 (d, *J* = 21.6 Hz), -11.52 (d, *J* = 20.5 Hz). HRMS-ESI: Calc for $C_9H_{19}O_7P_2$ $[M-H]^-$: 301.06059; Found: 301.0609.

*(E)-3,7-dimethylocta-2,6-dien-1-yl pyrophosphate (**32**)*
The title product was obtained as an off-white solid (30.8%) from (*E*)-3,7-dimethylocta-2,6-dien-1-ol. TLC (^{i}PrOH: NH_4OH: H_2O 7:2:1 v/v): R_f = 0.33; ^{1}H NMR (400 MHz, D_2O) δ 5.47 (tq, *J* = 7.2, 1.4 Hz, 1H), 5.28 – 5.15 (m, 1H), 4.49 (t, *J* = 6.8 Hz, 2H), 2.23 – 2.06 (m, 4H), 1.73 (s, 3H), 1.70 (s, 3H), 1.64 (s, 3H). ^{31}P NMR (162 MHz, D_2O) δ -10.22 (d, *J* = 20.6 Hz), -10.76 (d, *J* = 20.9 Hz). HRMS-ESI: Calc for $C_{10}H_{19}O_7P_2$ $[M-H]^-$: 313.06059; Found: 313.0606.

*(E)-3-methyl-4-(prop-2-yn-1-yloxy)but-2-en-1-yl pyrophosphate (**33**)*
The title product was obtained as a brown solid (29.8%) from (*E*)-3-methyl-4-(prop-2-yn-1-yloxy)but-2-en-1-ol**.** TLC (^{i}PrOH: NH_4OH: H_2O 7:2:1 v/v): R_f = 0.32; ^{1}H NMR (300 MHz, D_2O) δ 5.70 (t, *J* = 6.7 Hz, 1H), 4.50 (t, *J* = 6.7 Hz, 2H), 4.16 (dd, *J* = 2.4, 0.7 Hz, 2H), 4.03 (s, 2H), 2.89 – 2.76 (m, 1H), 1.69 (dd, *J* = 1.4, 0.7 Hz, 3H). ^{31}P NMR (122 MHz, D_2O) δ -7.23 (d, *J* = 22.1 Hz), -10.58 (d, *J* = 22.2 Hz). HRMS-ESI: Calc for $C_8H_{13}O_8P_2$ [M-H]$^-$: 299.00856; Found: 299.0086.

*(E)-5-azido-3-methylpent-2-en-1-yl pyrophosphate (**34**)*
The title product was obtained as a brown solid (32.5%) from (*E*)-5-azido-3-methylpent-2-en-1-ol. TLC (^{i}PrOH: NH_4OH: H_2O 7:2:1 v/v): R_f = 0.36; ^{1}H NMR (400 MHz, D_2O) δ 5.51 (t, *J* = 7.1 Hz, 1H), 4.46 (t, *J* = 6.9 Hz, 2H), 3.43 (t, *J* = 6.9 Hz, 2H), 2.33 (t, *J* = 6.9 Hz, 2H), 1.70 (s, 3H). ^{31}P NMR (162 MHz, D_2O) δ -8.37 (d, *J* = 21.1 Hz), -10.71 (d, *J* = 21.7 Hz). HRMS-ESI: Calc for $C_6H_{12}N_3O_7P_2$ [M-H]$^-$: 300.01504; Found: 300.0154.

*(2E,6E)-3,7-dimethylnona-2,6-dien-1-yl pyrophosphate (**35**)*
The title product was obtained as a white solid (30.2%) from (2*E*,6*E*)-3,7-dimethylnona-2,6-dien-1-ol. TLC (^{i}PrOH: NH_4OH: H_2O 7:2:1 v/v): R_f = 0.37; ^{1}H NMR (400 MHz, D_2O) δ 5.49 (s, 1H), 5.25 (s, 1H), 4.49 (t, *J* = 6.5 Hz, 2H), 2.24 – 2.07 (m, 2H), 2.01 (t, *J* = 7.4 Hz, 1H), 1.74 (s, 2H), 1.65 (s, 2H), 0.98 (t, *J* = 7.4 Hz, 2H).^{31}P NMR (162 MHz, D_2O) δ -7.53 (d, *J* = 22.0 Hz), -10.57 (d, *J* = 24.6 Hz). HRMS-ESI: Calc for $C_{11}H_{21}O_7P_2$ [M-H]$^-$: 327.07624; Found: 327.0770.

*(2E,6E)-8-hydroxy-3,7-dimethylocta-2,6-dien-1-yl pyrophosphate (**36**)*
The title product was obtained as a white-brown solid (28.9%) from (2*E*,6*E*)-8-((*tert*-butyldimethylsilyl)oxy)-3,7-dimethylocta-2,6-dien-1-ol. TLC (^{i}PrOH: NH_4OH: H_2O 7:2:1 v/v): R_f = 0.30; ^{1}H NMR (300 MHz, D_2O) δ 5.50 – 5.32 (m, 2H), 4.45 (d, *J* = 6.0 Hz, 2H), 3.93 (d, *J* = 1.1 Hz, 2H), 2.27 – 2.01 (m, 4H), 1.70 (d, *J* = 1.0 Hz, 3H), 1.62 (d, *J* = 1.3 Hz, 3H). ^{31}P NMR (243 MHz, D_2O) δ -10.61 (d, *J* = 20.6 Hz), -10.87 (d, *J* = 20.8 Hz). HRMS-ESI: Calc for $C_{10}H_{19}O_8P_2$ [M-H]$^-$: 329.05550; Found: 329.0554.

*(E)-4-((((l^5-azaneyl)oxy)((bis((l^5-azaneyl)oxy)phosphoryl)oxy)phosphoryl)oxy)-2-methylbut-2-en-1-yl 2-(methylamino)benzoate (**38**)*
The title product was obtained as a brown solid (30.5%) from (*E*)-4-hydroxy-2-methylbut-2-en-1-yl 2-(methylamino)benzoate. TLC (^{i}PrOH: NH_4OH: H_2O 7:2:1 v/v): R_f = 0.37 ^{1}H NMR (400 MHz, D_2O) δ 7.84 (dd, *J* = 8.0, 1.7 Hz, 1H), 7.37 (ddd, *J* = 8.7, 7.1, 1.7 Hz, 1H), 6.73 (dd, *J* = 8.3, 1.0 Hz, 1H), 6.60 (ddd, *J* = 8.2, 7.1, 1.1 Hz, 1H), 5.85 – 5.47 (m, 1H), 4.60 (s, 2H), 4.41 (t, *J* = 6.8 Hz, 2H), 2.72 (s, 3H), 1.65 (s, 3H), 1.01 (d, *J* = 6.2 Hz, 2H). ^{31}P NMR (162 MHz, D_2O) δ -9.30 (d, *J* = 18.3 Hz), -10.78 (d, *J* = 21.7 Hz). HRMS-ESI: Calc for $C_{13}H_{20}NO_9P_2$ [M+H]$^+$: 396.06132; Found: 396.0618.

*Methyl (E)-2-(((4-((((l^5-azaneyl)oxy)((bis((l^5-azaneyl)oxy)phosphoryl)oxy)phosphoryl)oxy)-2-methylbut-2-en-1-yl)amino)methyl)benzoate (**39**)*
The title product was obtained as a brown solid (29.7%) from methyl (*E*)-2-(((4-hydroxy-2-methylbut-2-en-1-yl)amino)methyl)benzoate. TLC (^{i}PrOH: NH_4OH: H_2O 7:2:1 v/v): R_f = 0.31; ^{1}H NMR (400 MHz, D_2O) δ 7.79 (dd, *J* = 8.1, 1.7 Hz, 1H), 7.30 (ddd, *J* = 8.7, 7.1, 1.7 Hz, 1H), 6.76 – 6.66 (m, 1H), 6.58 (ddd, *J* = 8.1, 7.1, 1.1 Hz, 1H), 5.49 (tt, *J* = 5.6, 2.9 Hz, 1H), 4.36 (t, *J* = 6.8 Hz, 2H), 3.74 (s, 4H), 1.59 (s, 3H). ^{31}P NMR (162 MHz, D_2O) δ -8.20 (d, *J* = 21.6 Hz), -10.69 (d, *J* = 21.8 Hz). HRMS-ESI: Calc for $C_{13}H_{20}NO_9P_2$ [M+H]$^+$:396.06132; Found: 396.0612.

*(2E,6E)-3,7-dimethylundeca-2,6,10-trien-1-yl pyrophosphate (**40**)*
The title product was obtained as a white solid (33.1%) from (2*E*,6*E*)-3,7-dimethylundeca-2,6,10-trien-1-ol. TLC (^{i}PrOH: NH_4OH: H_2O 7:2:1 v/v): R_f = 0.35; ^{1}H NMR (400 MHz, D_2O) δ 5.89 (ddt, *J* = 17.0, 10.1, 6.5 Hz, 1H), 5.48 (t, *J* = 7.4 Hz, 1H), 5.25 (t, *J* = 6.8 Hz, 1H), 5.08 (d, *J* = 17.3 Hz, 2H), 5.00 (ddd, *J* = 10.1, 2.4, 1.2 Hz, 2H), 4.49 (t, *J* = 6.6 Hz, 2H), 2.38 – 1.97 (m, 8H), 1.73 (s, 3H), 1.64 (s, 3H). ^{31}P NMR (162 MHz, D_2O) δ -7.38 (s), -10.55 (d, *J* = 21.3 Hz). HRMS-ESI: Calc for $C_{13}H_{23}O_7P_2$ [M-H]$^-$: 353.09189; Found: 353.0926.

*(2*E*,6*E*)-8-((hydroxy(phosphonooxy)phosphoryl)oxy)-2,6-dimethylocta-2,6-dien-1-yl acetate (**41**)*
The title product was obtained as a white-brwon solid (31.0%) from (2*E*,6*E*)-9-hydroxy-2,6-dimethylnona-2,6 dien-1-yl acetate. TLC (^{i}PrOH: NH_4OH: H_2O 7:2:1 v/v): R_f = 0.37; ^{1}H NMR (300 MHz, D_2O) δ 5.37 (t, *J* = 5.9 Hz, 1H), 5.28 (d, *J* = 5.3 Hz, 1H), 4.40 – 3.86 (m, 4H), 2.04 (t, *J* = 7.0 Hz, 2H), 1.99 – 1.84 (m, 5H), 1.54 (s, 3H), 1.49 (s, 3H). ^{31}P NMR (122 MHz, D_2O) δ -8.69 (d, *J* = 20.7 Hz), -10.76 (d, *J* = 21.8 Hz). HRMS-ESI: Calc for $C_{12}H_{21}O_9P_2$ [M-H]$^-$:371.06607; Found: 371.0656.

*(2*E*,6*E*)-3,7,11-trimethyldodeca-2,6,10-trien-1-yl pyrophosphate (**42**)*
The title product was obtained as an ivory solid (32.7%) from (2*E*,6*E*)-3,7,11-trimethyldodeca-2,6,10-trien-1-ol. TLC (^{i}PrOH: NH_4OH: H_2O 7:2:1 v/v): R_f = 0.39; ^{1}H NMR (400 MHz, D_2O) δ 5.47 (t, *J* = 8.2 Hz, 1H), 5.31 – 5.11 (m, 2H), 4.49 (t, *J* = 6.6 Hz, 2H), 2.25 – 1.95 (m, 8H), 1.73 (s, 3H), 1.70 (s, 2H), 1.63 (s, 3H). ^{31}P NMR (162 MHz, D_2O) δ -8.71 (d, *J* = 21.2 Hz), -10.61 (d, *J* = 21.0 Hz). HRMS-ESI: Calc for $C_{15}H_{27}O_7P_2$ [M-H]$^-$: 381.12319; Found: 381.1244.

*(2*E*,6*E*)-3,7-dimethyl-8-(prop-2-yn-1-yloxy)octa-2,6-dien-1-yl pyrophosphate (**43**)*
The title product was obtained as a brown solid (29.7%) from (2*E*,6*E*)-3,7-dimethyl-8-(prop-2-yn-1-yloxy)octa-2,6-dien-1-ol. TLC (^{i}PrOH: NH_4OH: H_2O 7:2:1 v/v): R_f = 0.38; ^{1}H NMR (300 MHz, D_2O) δ 5.59 – 5.47 (m, 1H), 5.46 – 5.35 (m, 1H), 4.44 (t, *J* = 6.7 Hz, 2H), 4.09 (d, *J* = 2.4 Hz, 2H), 3.97 (d, *J* = 0.9 Hz, 3H), 2.83 (t, *J* = 2.4 Hz, 1H), 2.18 (dd, *J* = 15.7, 9.2 Hz, 4H), 1.69 (d, *J* = 1.4 Hz, 3H), 1.61 (d, *J* = 1.3 Hz, 4H). ^{31}P NMR (122 MHz, D_2O) δ -10.11 (d, *J* = 20.7 Hz), -10.84 (d, *J* = 20.9 Hz). HRMS-ESI: Calc for $C_{13}H_{21}O_8P_2$ [M-H]$^-$: 367.07115; Found:367.0718.

*(2*E*,6*E*)-8-(benzyloxy)-3,7-dimethylocta-2,6-dien-1-yl pyrophosphate (**44**)*
The title product was obtained as a white solid (32.6%) from (2*E*,6*E*)-8-(benzyloxy)-3,7-dimethylocta-2,6-dien-1-ol. TLC (^{i}PrOH: NH_4OH: H_2O 7:2:1 v/v): R_f = 0.35; ^{1}H NMR (300 MHz, D_2O) δ 7.52 – 7.20 (m, 5H), 5.57 – 5.33 (m, 2H), 4.46 (s, 4H), 4.04 – 3.80 (m, 2H), 3.04 (q, *J* = 7.3 Hz, 2H), 1.70 (d, *J* = 1.3 Hz, 3H), 1.65 – 1.63 (m, 3H), 1.24 (t, *J* = 7.3 Hz, 2H). ^{31}P NMR (122 MHz, D_2O) δ -9.95 (d, *J* = 20.9 Hz), -10.85 (d, *J* = 20.9 Hz). HRMS-ESI: Calc for $C_{17}H_{25}O_8P_2$ [M-H]$^-$: 419.10245; Found: 419.1027.

*benzo[*d*][1,3]dioxol-5-ylmethyl pyrophosphate (**58**)*
The title product was obtained as a brown-white solid (32.8%) from benzo[*d*][1,3]dioxol-5-ylmethanol. TLC (^{i}PrOH: NH_4OH: H_2O 7:2:1 v/v): R_f = 0.35; ^{1}H NMR (300 MHz, D_2O) δ 7.03 (d, *J* = 1.7 Hz, 1H), 6.95 (d, *J* = 1.7 Hz, 1H), 6.89 (s, 1H), 5.96 (s, 2H), 4.87 (d, *J* = 6.4 Hz, 3H). ^{31}P NMR (122 MHz, D_2O) δ -8.18 (d, *J* = 21.0 Hz), -11.07 (d, *J* = 21.8 Hz). HRMS-ESI: Calc for $C_8H_9O_9P_2$ [M-H]$^-$: 310.97217; Found: 310.9735.

*4-(prop-2-yn-1-yloxy)benzyl pyrophosphate (**59**)*
The title product was obtained as a brown solid (20.6%) from (4-(prop-2-yn-1-yloxy)phenyl)methanol. TLC (^{i}PrOH: NH_4OH: H_2O 7:2:1 v/v): R_f = 0.33; ^{1}H NMR (400 MHz, D_2O) δ 7.49 (d, *J* = 8.4 Hz, 2H), 7.13 – 7.07 (m, 2H), 4.96 (d, *J* = 6.3 Hz, 2H), 4.83 (s, 2H), 2.95 (d, *J* = 2.5 Hz, 1H). ^{31}P NMR (162 MHz, D_2O) δ -7.48 (d, *J* = 21.9 Hz), -10.85 (d, *J* = 21.8 Hz). HRMS-ESI: Calc for $C_{10}H_{11}O_8P_2$ [M-H]$^-$: 320.99291; Found: 320.9925.

*4-(2-azidoethoxy)benzyl pyrophosphate (**60**)*
The title product was obtained as a white-brwon solid (31.1%) from (4-(2-azidoethoxy)phenyl)methanol. TLC (^{i}PrOH: NH_4OH: H_2O 7:2:1 v/v): R_f = 0.36; ^{1}H NMR (400 MHz, D_2O) δ 7.48 (d, *J* = 8.6 Hz, 2H), 7.10 – 7.05 (m, 2H), 4.95 (d, *J* = 6.3 Hz, 2H), 4.28 (t, *J* = 4.8 Hz, 2H), 3.71 (t, *J* = 4.8 Hz, 2H). ^{31}P NMR (162 MHz, D_2O) δ -7.71 (d, *J* = 21.8 Hz), -10.90 (d, *J* = 22.0 Hz). HRMS-ESI: Calc for $C_9H_{12}N_3O_8P_2$ [M-H]$^-$: 352.00995; Found: 352.0092.

*furan-2-ylmethyl pyrophosphate (**61**)*
The title product was obtained as an ivory solid (32.8%) from furan-2-ylmethanol. TLC (^{i}PrOH: NH_4OH: H_2O 7:2:1 v/v): R_f = 0.35; ^{1}H NMR (400 MHz, D_2O) δ 7.55 (d, *J* = 1.9 Hz, 1H), 6.55 (d, *J* = 3.3 Hz, 1H), 6.47 (dd, *J* = 3.3, 1.9 Hz, 1H), 4.95 (d, *J* = 6.7 Hz, 3H). ^{31}P NMR (162 MHz, D_2O) δ -7.09 (d, *J* = 22.1 Hz), -11.07 (d, *J* = 22.1 Hz). HRMS-ESI: Calc for $C_5H_7O_8P_2$ [M-H]$^-$: 256.96161; Found: 256.9625.

*thiophen-2-ylmethyl pyrophosphate (**62**)*
The title product was obtained as an ivory solid (33%) from thiophen-2-ylmethanol. TLC (^{i}PrOH: NH_4OH: H_2O 7:2:1 v/v): R_f = 0.35; ^{1}H NMR (400 MHz, D_2O) δ 7.47 (d, *J* = 5.1 Hz, 1H), 7.21 (s, 1H), 7.07 (d, *J* = 4.9 Hz, 1H), 5.16 (d, *J* = 6.3 Hz, 2H).^{31}P NMR (162 MHz, D_2O) δ -8.25 (d, *J* = 21.5 Hz), -11.36 (d, *J* = 21.7 Hz). HRMS-ESI: Calc for $C_5H_7O_7P_2S$ [M-H]$^-$: 272.93877; Found: 272.9379.

*tri(l^5-azaneyl) (thiazol-4-ylmethyl) pyrophosphate (**63**)*

The title product was obtained as a yellow-brown solid (30.7%) from thiazol-4-ylmethanol. TLC (^{i}PrOH: NH_4OH: H_2O 7:2:1 v/v): R_f = 0.34; ^{1}H NMR (300 MHz, $CDCl_3$ δ 11.43 (d, *J* = 2.1 Hz, 1H), 10.15 – 9.94 (m, 1H), 7.41 (dd, *J* = 6.2, 0.8 Hz, 3H). ^{31}P NMR (122 MHz, $CDCl_3$) δ -6.05 (d, *J* = 20.9 Hz), -8.55 (d, *J* = 21.3 Hz). HRMS-ESI: Calc for $C_{12}H_{21}O_9P_2$ $[M\text{-}H]^-$:371.06607; Found: 371.0656.

*tri(l^5-azaneyl) (pyridin-1-ylmethyl) pyrophosphate (**64**)*

The title product was obtained as a brown solid (25.6%) from pyridin-1-ylmethanol. TLC (^{i}PrOH: NH_4OH: H_2O 7:2:1 v/v): R_f = 0.34; ^{1}H NMR (300 MHz, D_2O) δ 8.33 (dd, *J* = 5.3, 1.7 Hz, 1H), 7.91 – 7.80 (m, 1H), 7.49 (d, *J* = 8.0 Hz, 1H), 7.37 – 7.27 (m, 1H), 4.93 (d, *J* = 7.4 Hz, 2H). ^{31}P NMR (122 MHz, D_2O) δ -7.41 (d, *J* = 21.8 Hz), -10.70 (d, *J* = 21.7 Hz). HRMS-ESI: Calc for $C_6H_8NO_7P_2$ $[M\text{-}H]^-$: 267.97759; Found: 267.9756.

*benzofuran-2-ylmethyl pyrophosphate (**65**)*

The title product was obtained as an off-white solid (31.6%) from benzofuran-2-ylmethanol. TLC (^{i}PrOH: NH_4OH: H_2O 7:2:1 v/v): R_f = 0.34; ^{1}H NMR (300 MHz, D_2O) δ 7.73 – 7.57 (m, 1H), 7.58 – 7.49 (m, 1H), 7.44 – 7.15 (m, 2H), 6.92 (s, 1H), 5.07 (d, *J* = 7.2 Hz, 3H). ^{31}P NMR (122 MHz, D_2O) δ -7.48 (d, *J* = 21.4 Hz), -10.98 (d, *J* = 21.5 Hz). HRMS-ESI: Calc for $C_9H_9O_8P_2$ $[M\text{-}H]^-$: 306.97726; Found: 306.9780.

*benzo[*b*]thiophen-3-ylmethyl pyrophosphate (**66**)*

The title product was obtained as a brown-white solid (29.7%) from benzo[*b*]thiophen-3-ylmethanol. TLC (^{i}PrOH: NH_4OH: H_2O 7:2:1 v/v): R_f = 0.39; ^{1}H NMR (400 MHz, D_2O) δ 8.11 – 8.04 (m, 1H), 8.02 (dt, *J* = 7.9, 0.9 Hz, 1H), 7.74 (s, 1H), 7.53 (ddd, *J* = 8.1, 7.1, 1.3 Hz, 1H), 7.48 (td, *J* = 7.6, 7.1, 1.4 Hz, 1H), 5.28 (dd, *J* = 5.9, 0.9 Hz, 2H). ^{31}P NMR (162 MHz, D_2O) δ -7.90 (d, *J* = 21.9 Hz), -11.03 (d, *J* = 21.9 Hz). HRMS-ESI: Calc for $C_9H_9O_7P_2S$ $[M\text{-}H]^-$: 322.95442; Found: 322.9555.

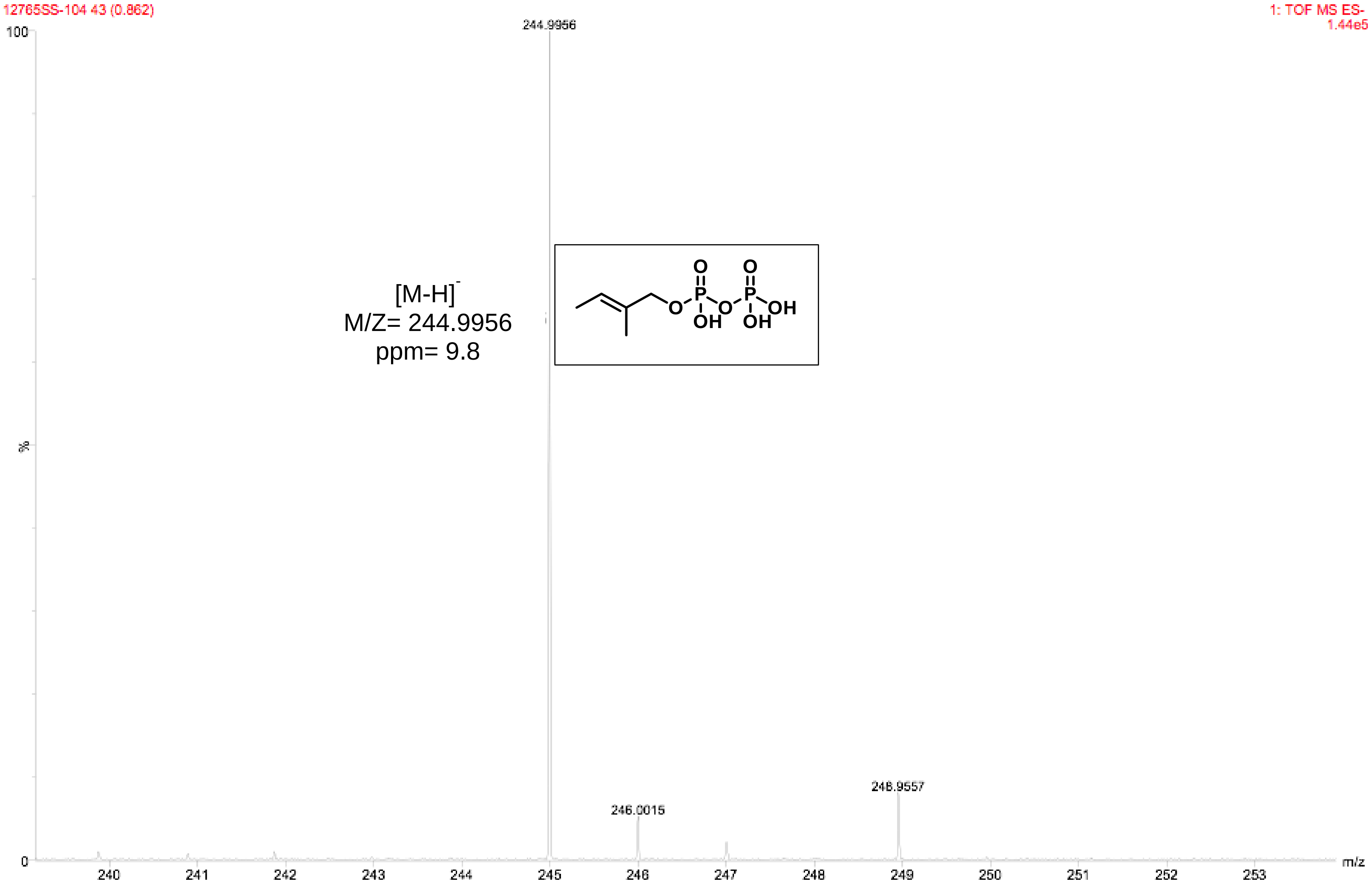

(-)-ESI-MS Spectrum of **3**

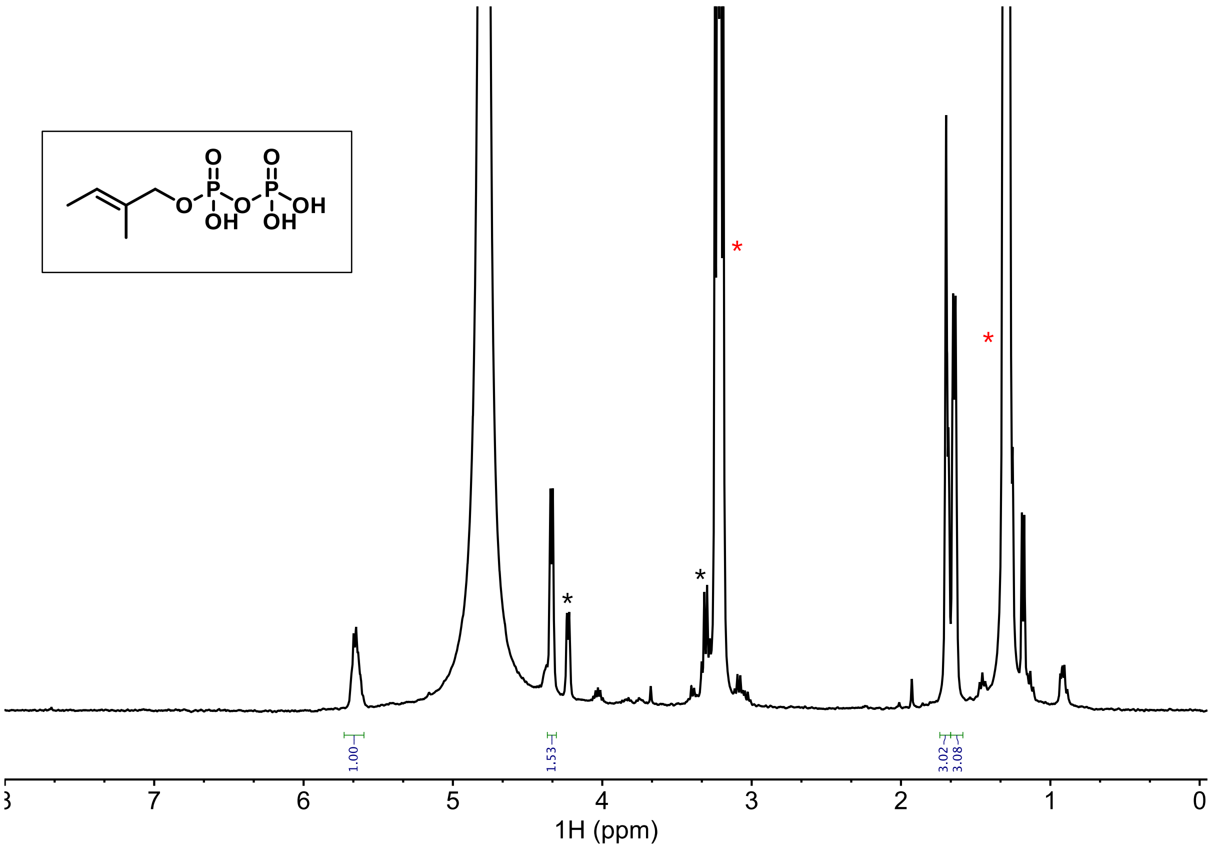

^{1}H NMR Spectrum of **3** (400 MHz, D_2O) *Denotes an impurity. *Denotes TEAP counterion.

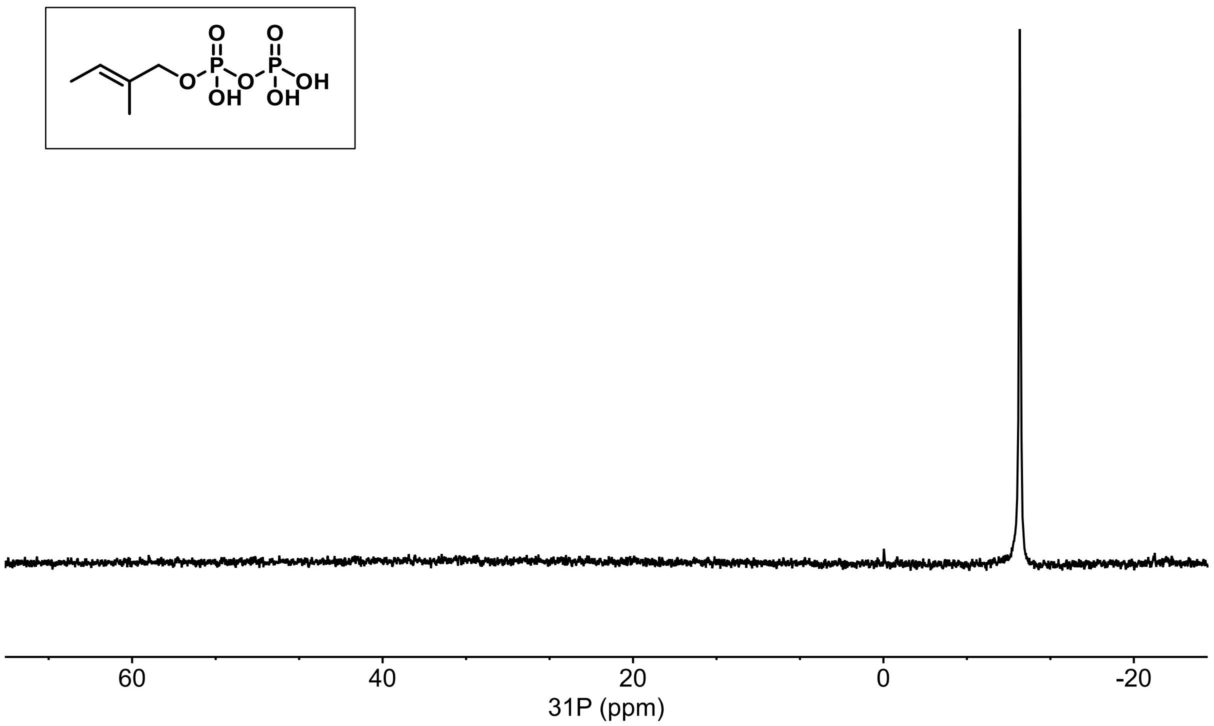

^{31}P NMR Spectrum of **3** (243 MHz, D2O). Both signals are overlapping with each other.

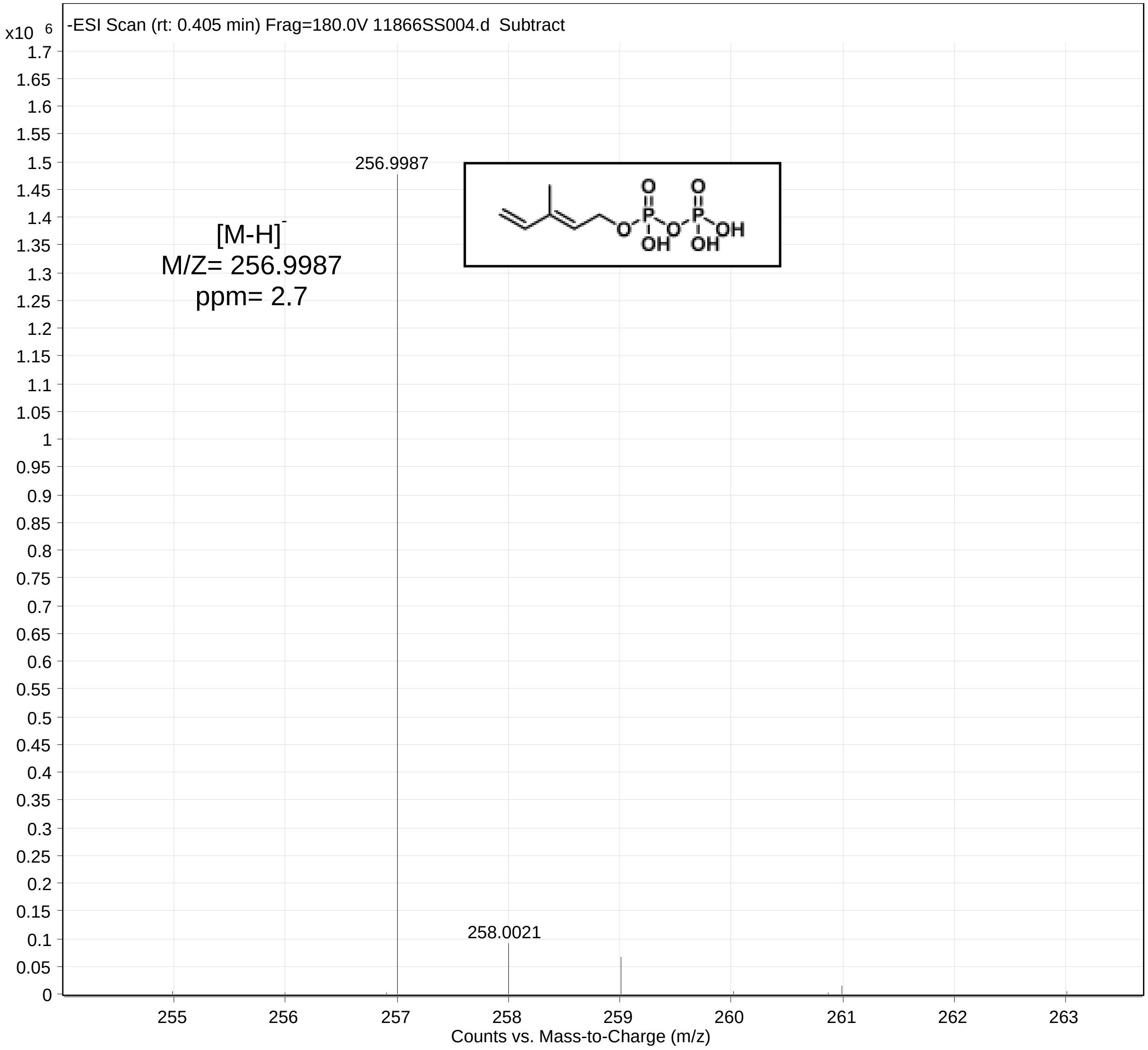

(-)-ESI-HRMS Spectrum of **8**

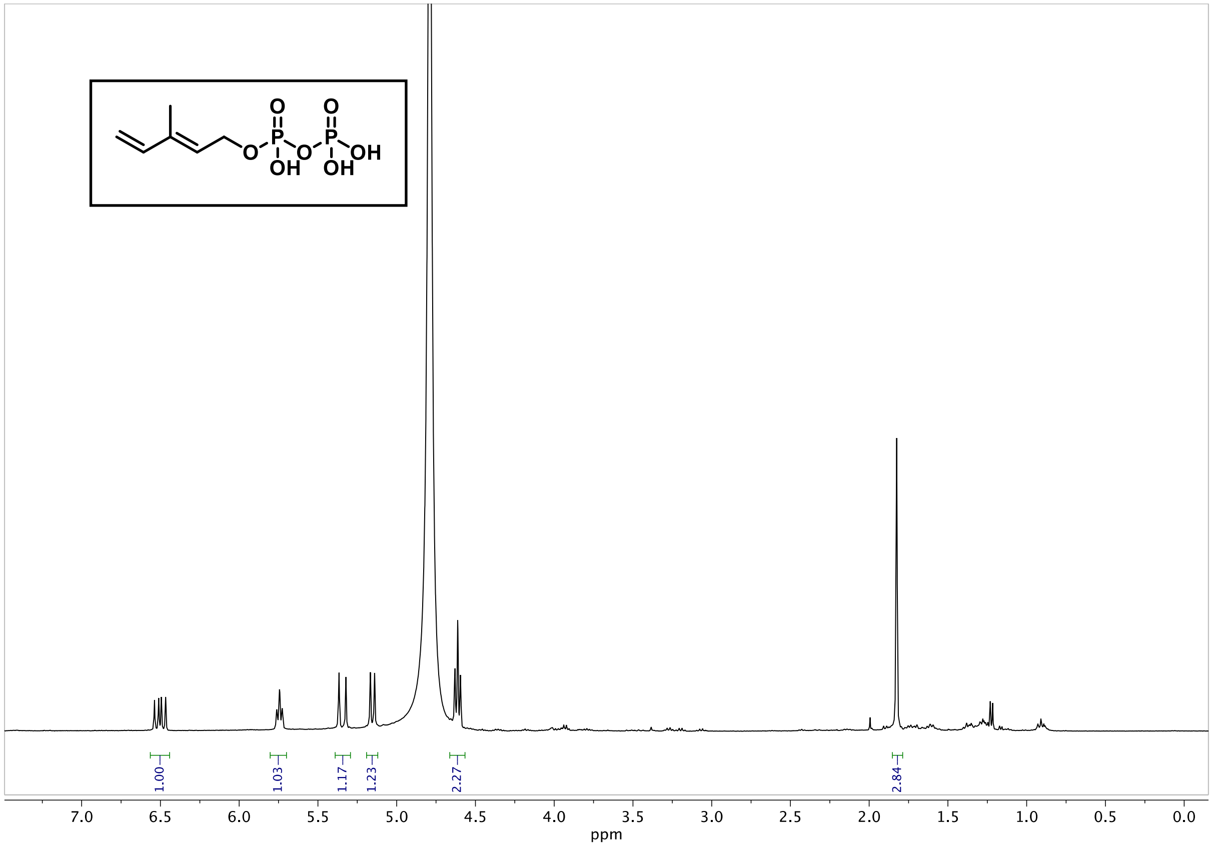

^{1}H NMR Spectrum of **8** (400 MHz, D_2O)

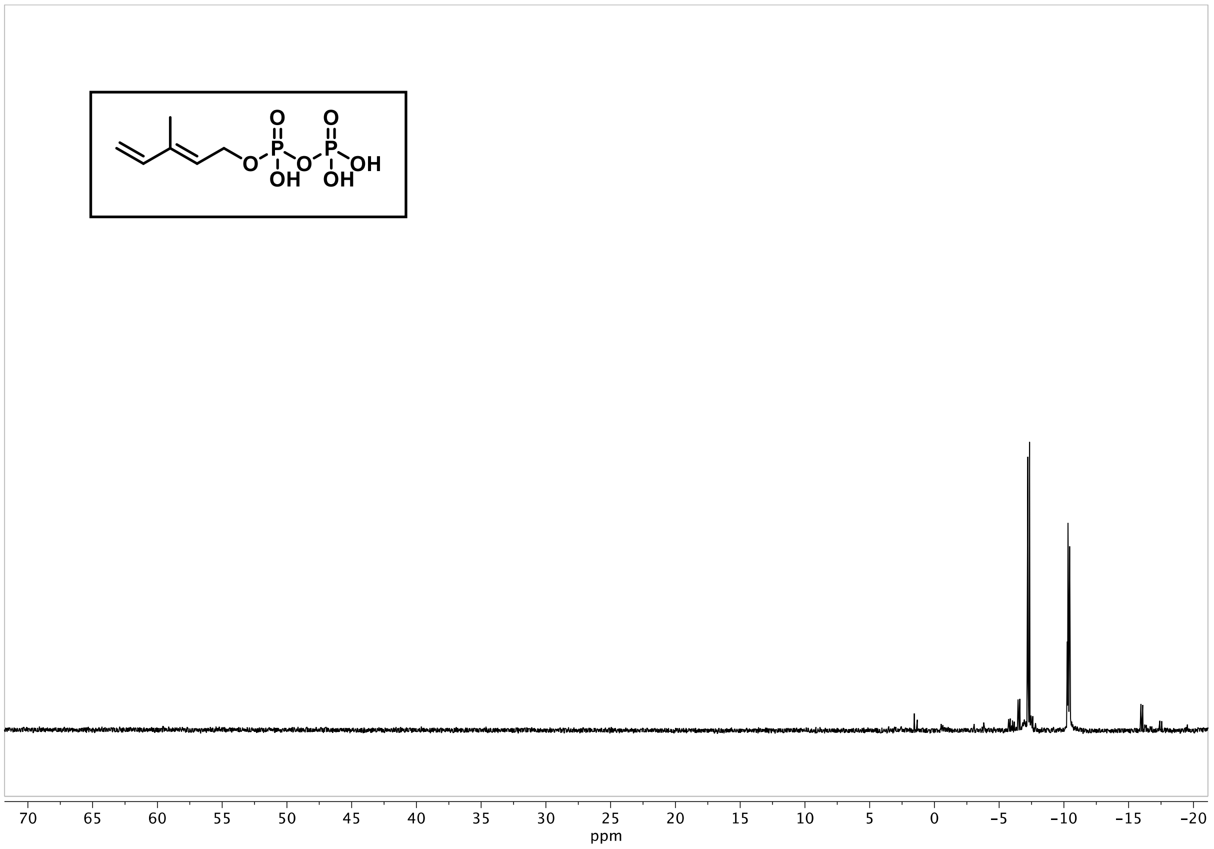

^{31}P NMR Spectrum of **8** (162 MHz, D_2O)

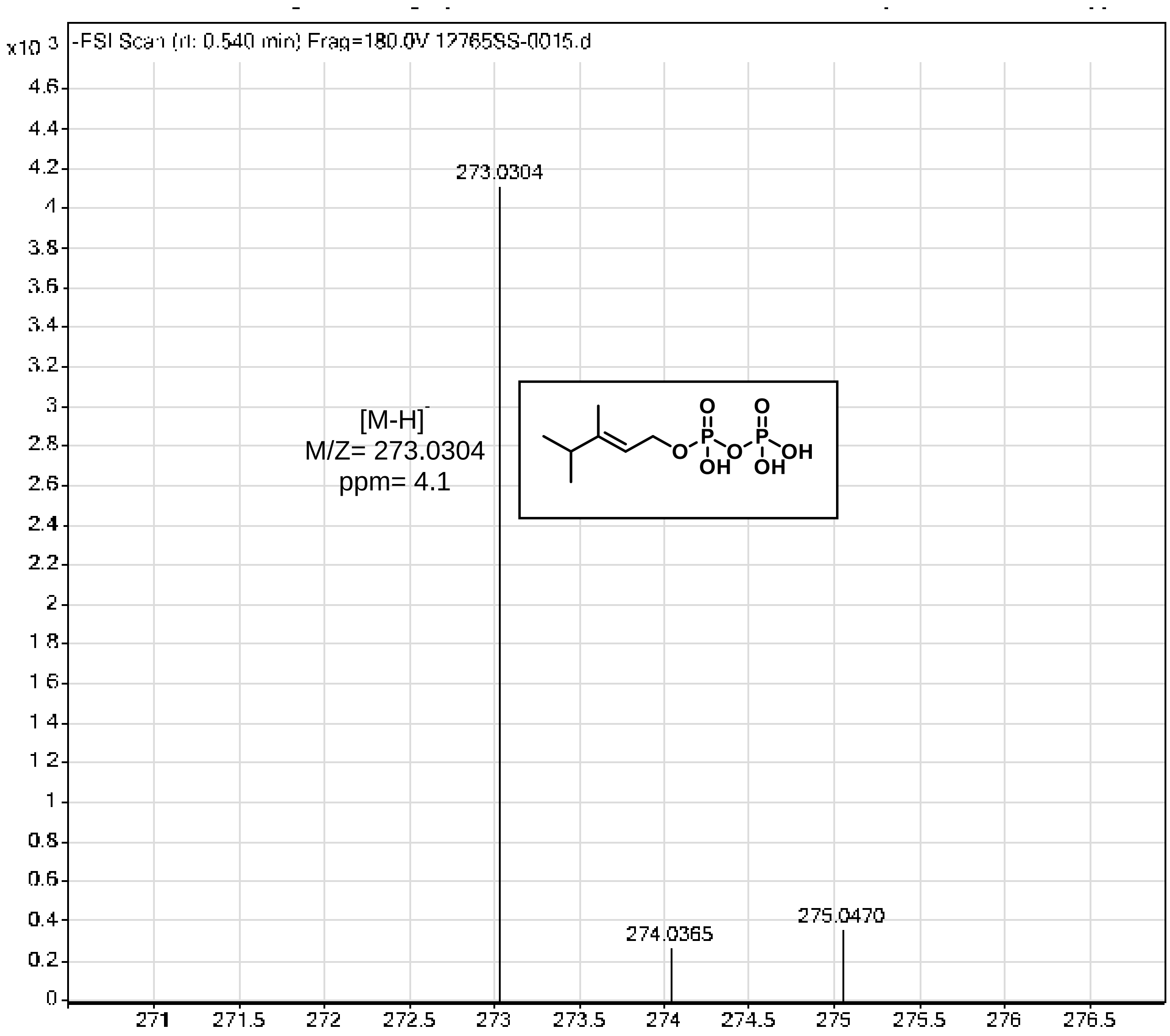

(-)-ESI-HRMS Spectrum of **10**

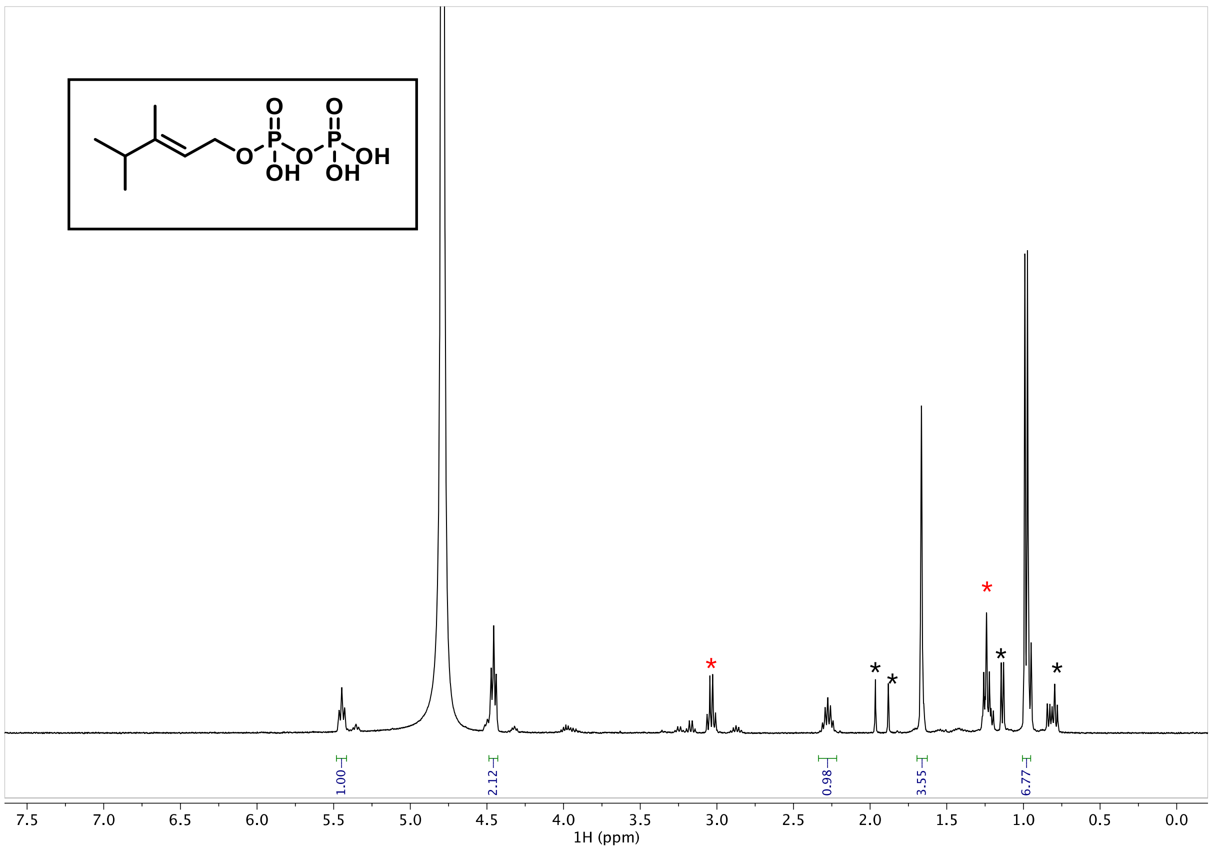

^{1}H NMR Spectrum of **10** (400 MHz, D_2O) *Denotes an impurity. *Denotes TEAP counterion.

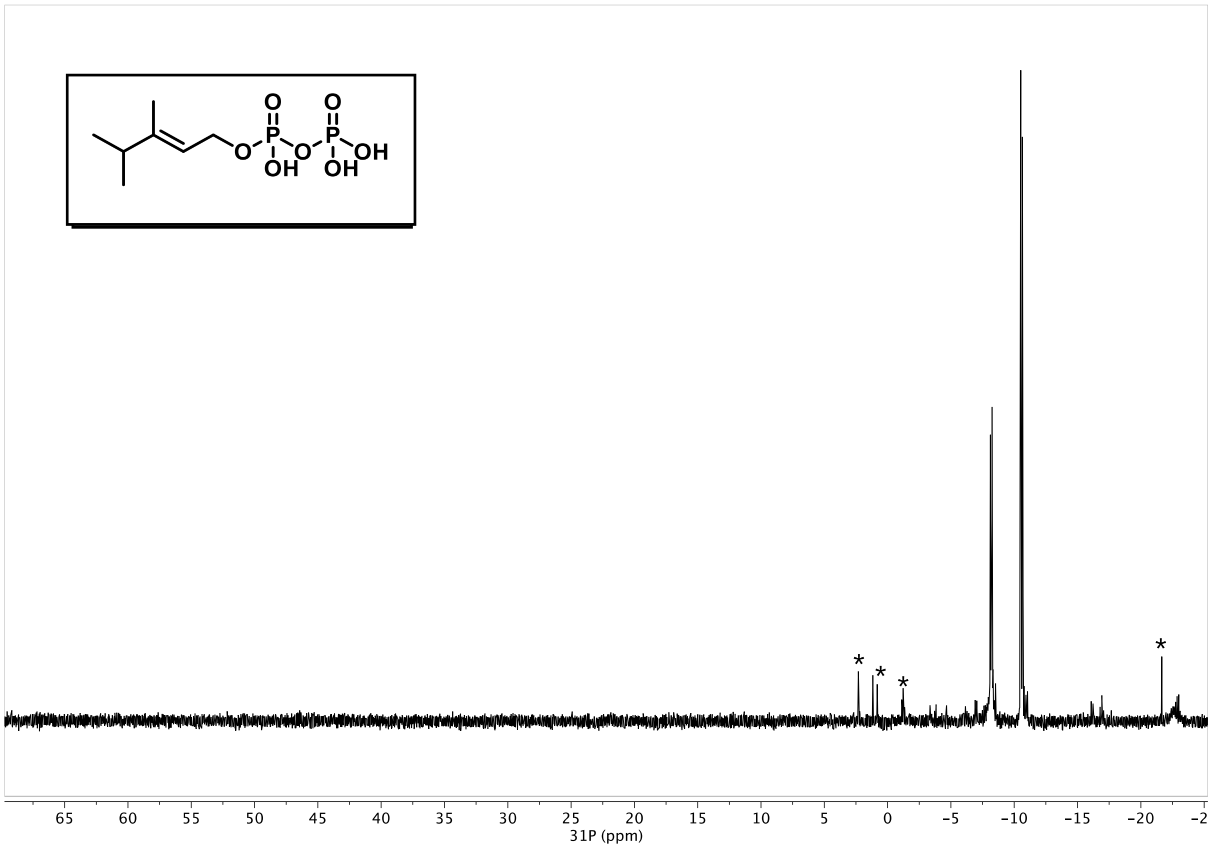

^{31}P NMR Spectrum of **10** (162 MHz, D_2O) *Denotes an impurity.

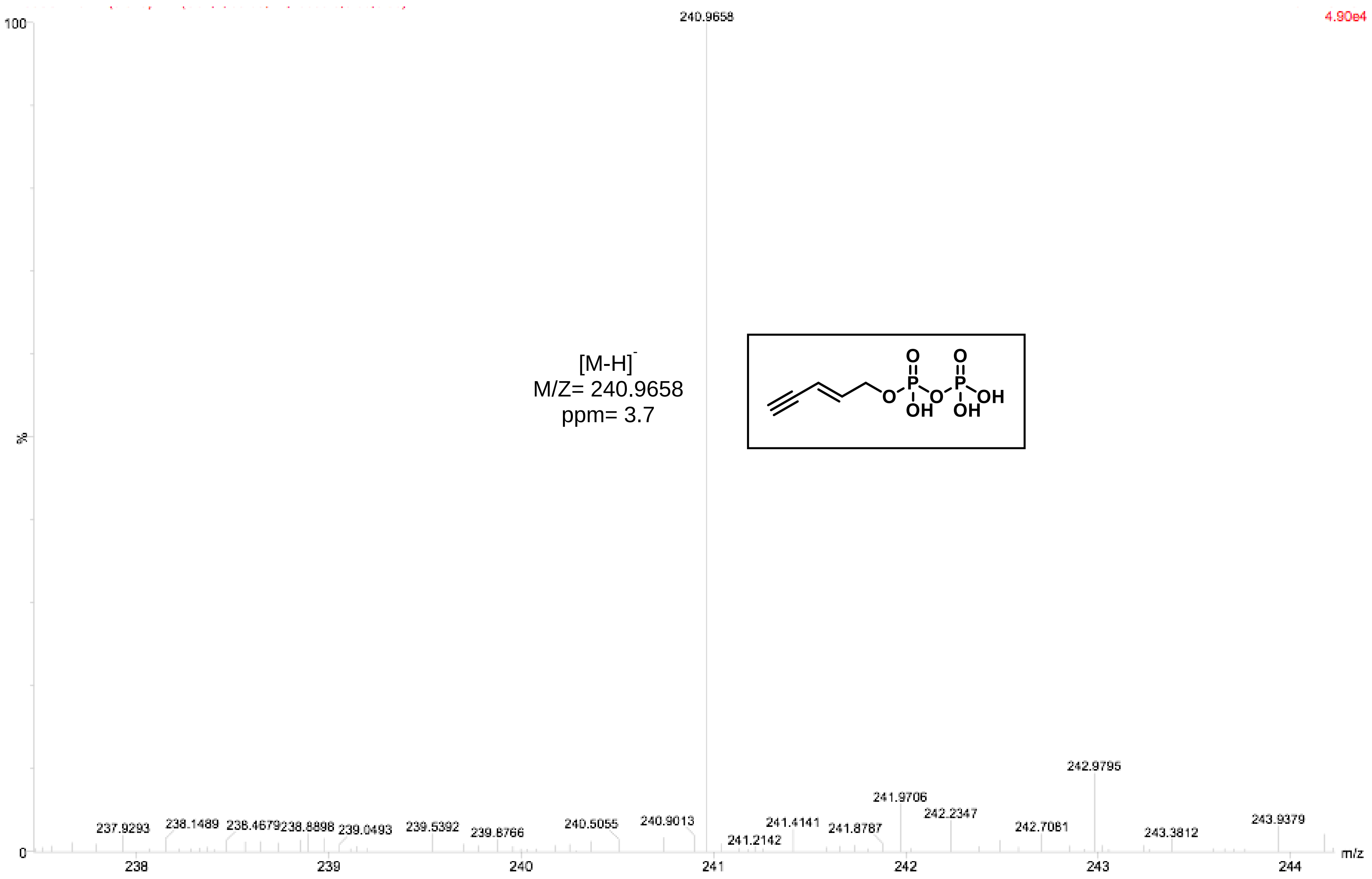

(-)-ESI-HRMS Spectrum of **14**

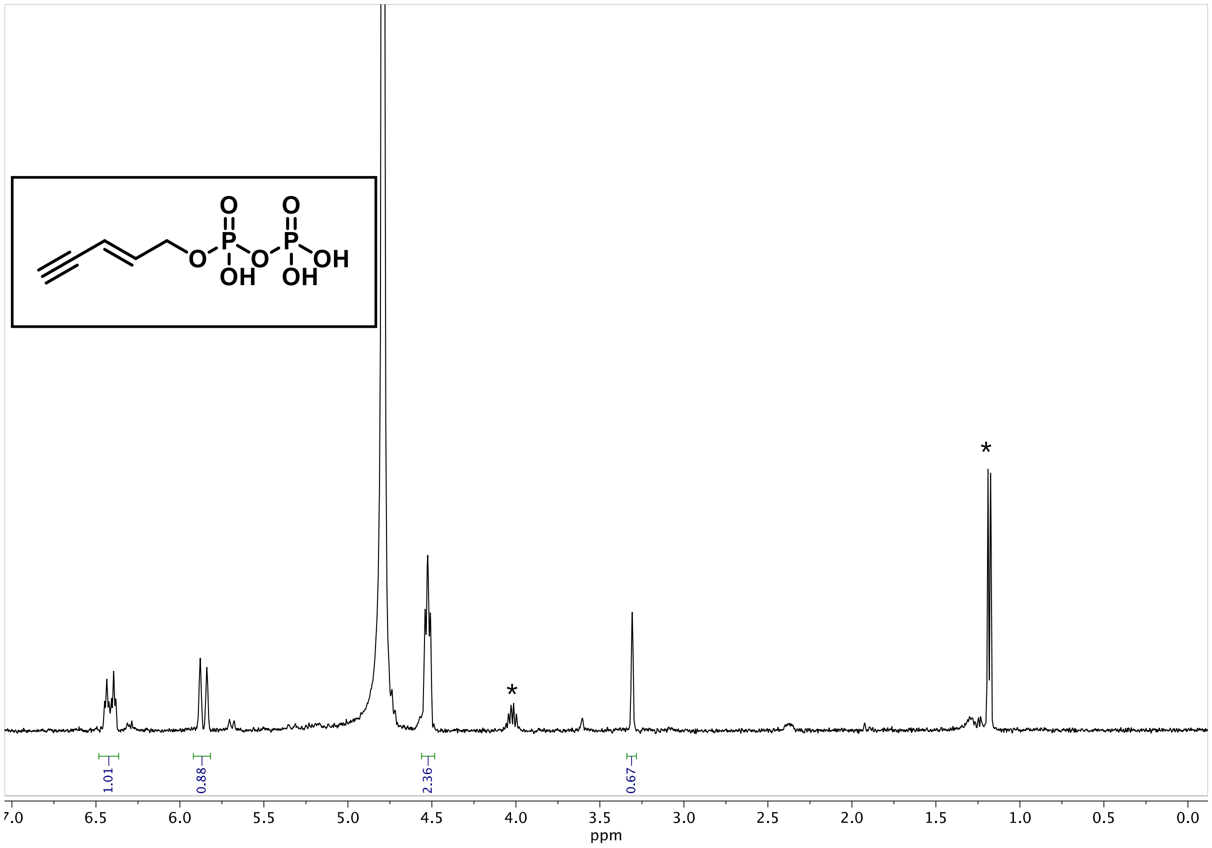

^{1}H NMR Spectrum of **14** (400 MHz, D_2O) *Denotes an impurity.

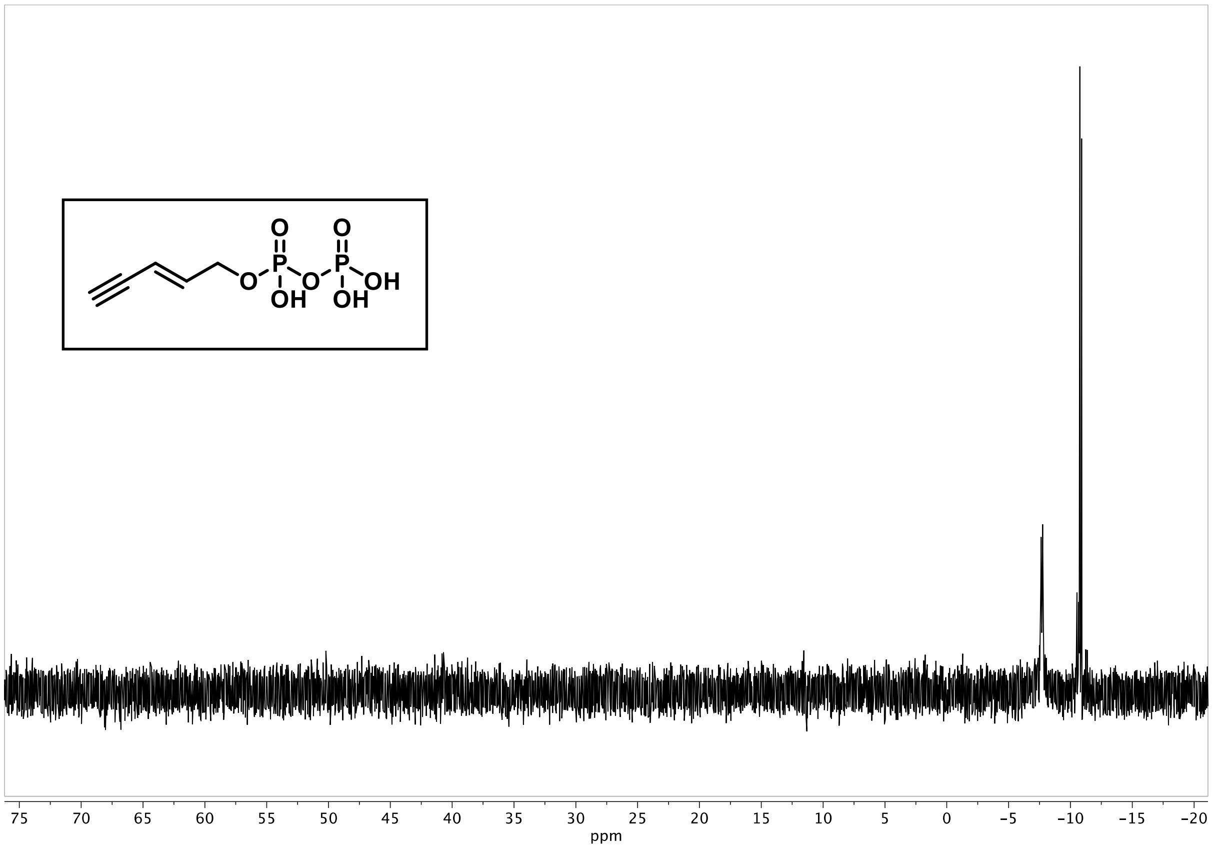

^{31}P NMR Spectrum of **14** (162 MHz, D_2O)

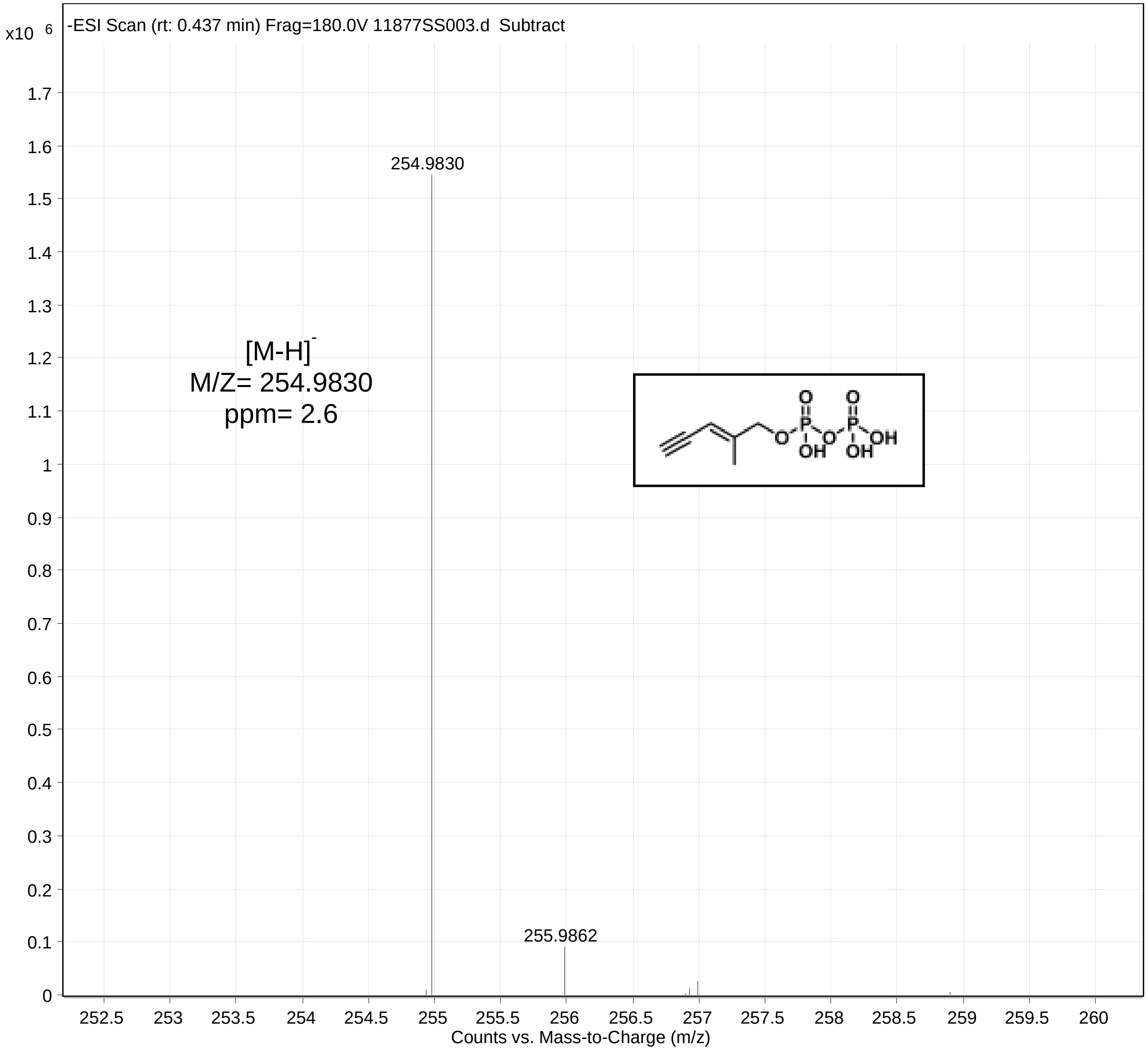

(-)-ESI-HRMS Spectrum of **15**

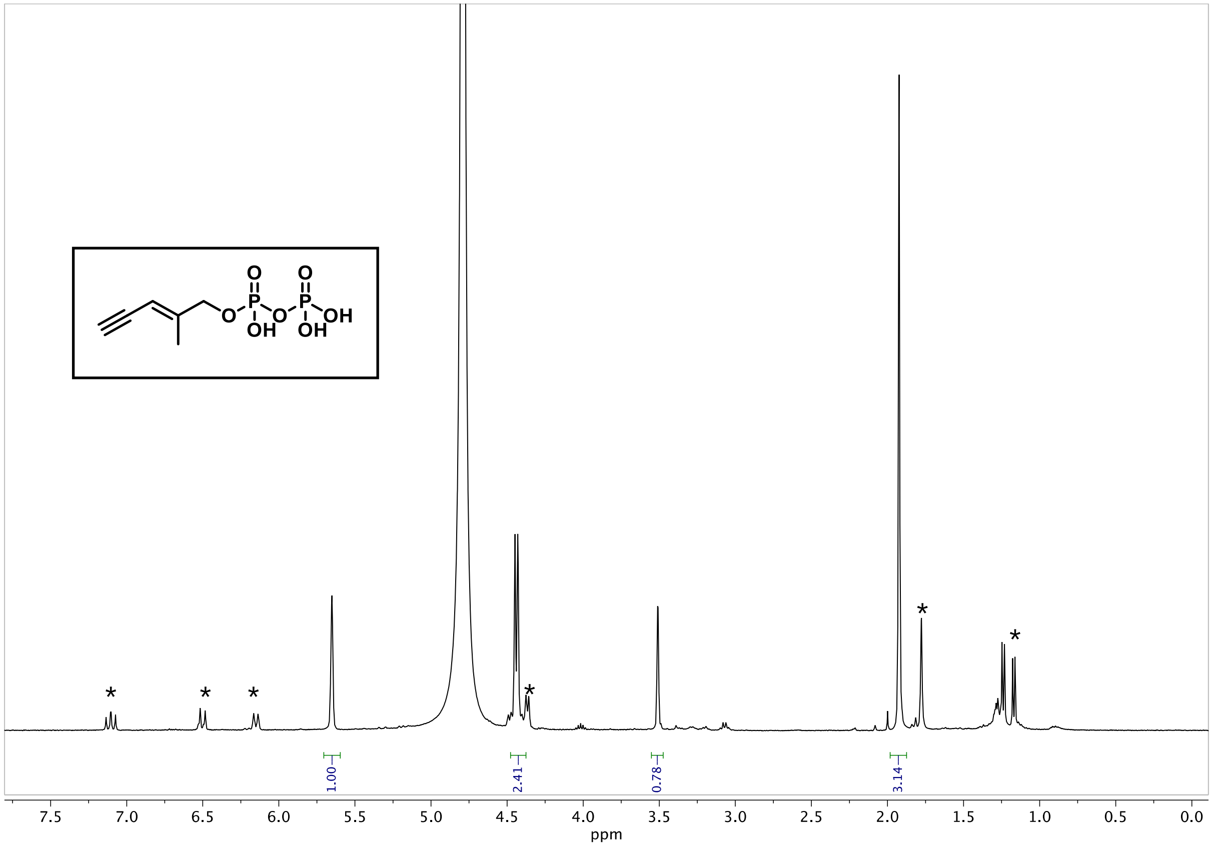

^{1}H NMR of **15** (400 MHz, D_2O) *Denotes an impurity.

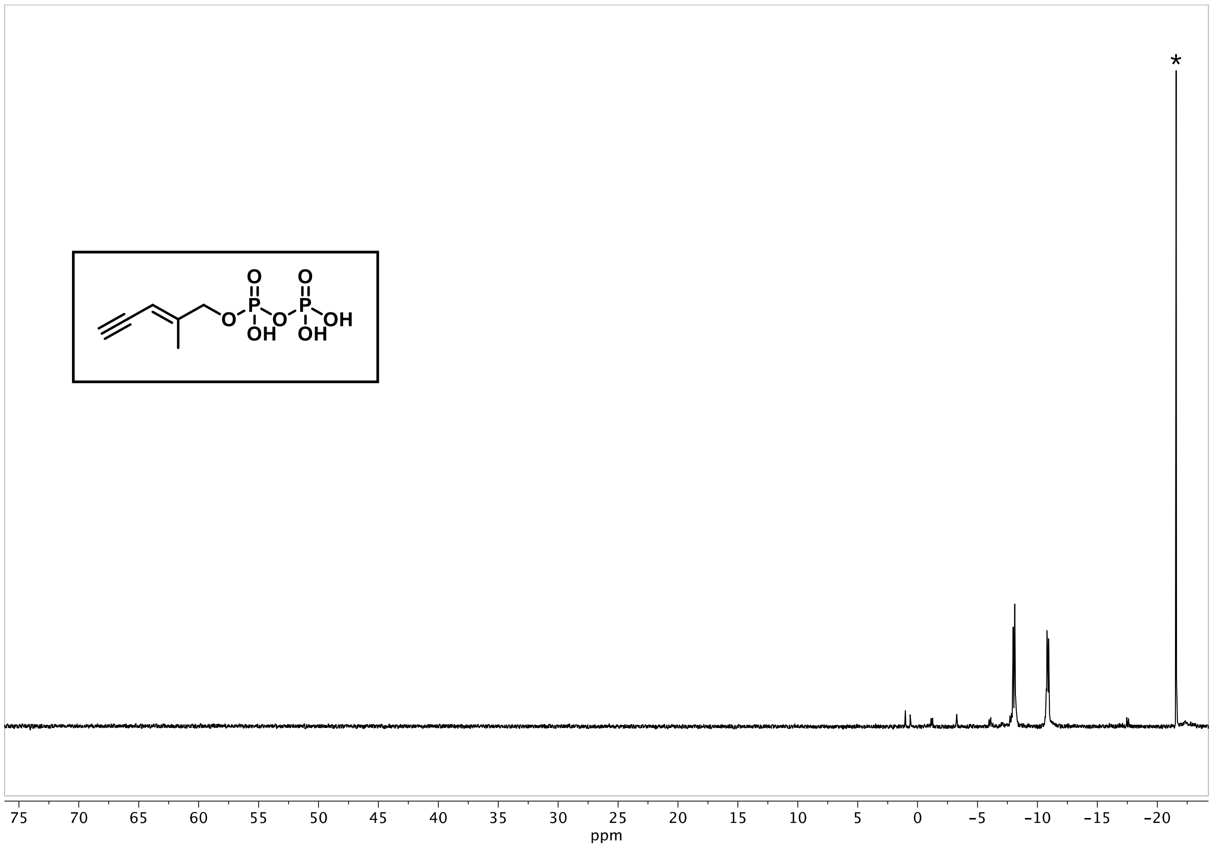

^{31}P NMR of **15** (162 MHz, D_2O) *Denotes an impurity.

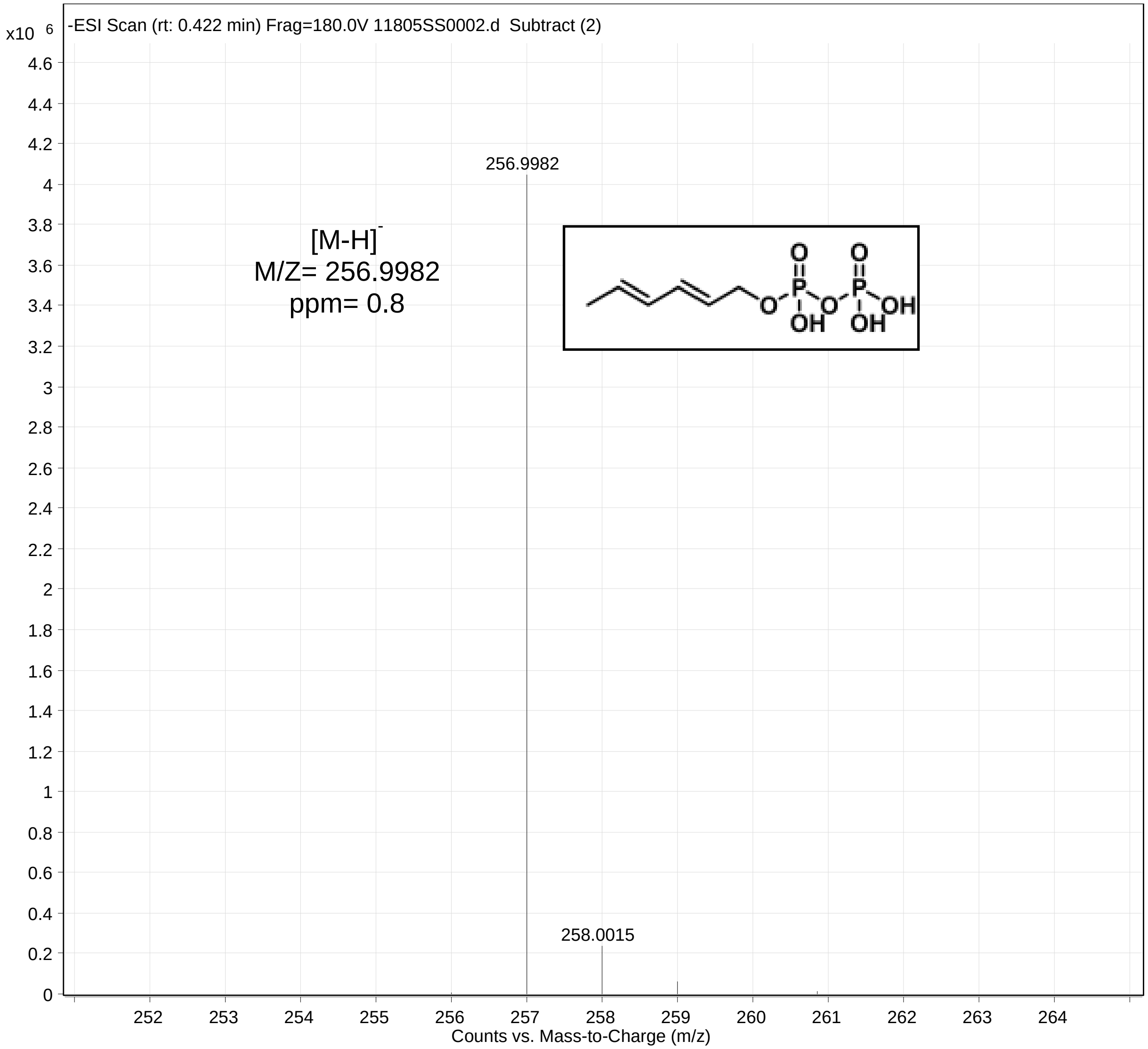

(-)-ESI-HRMS Spectrum of **16**

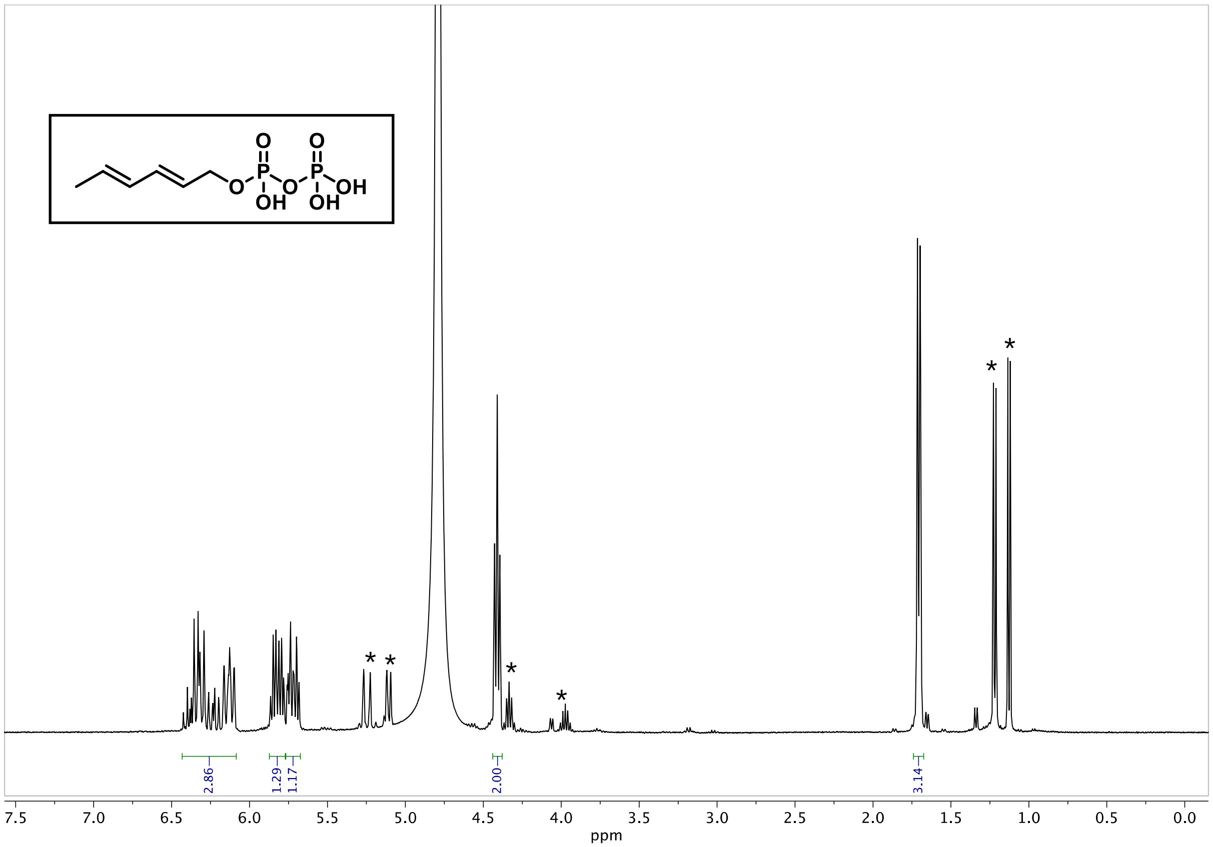

^{1}H NMR Spectrum of **16** (400 MHz, D_2O) *Denotes an impurity.

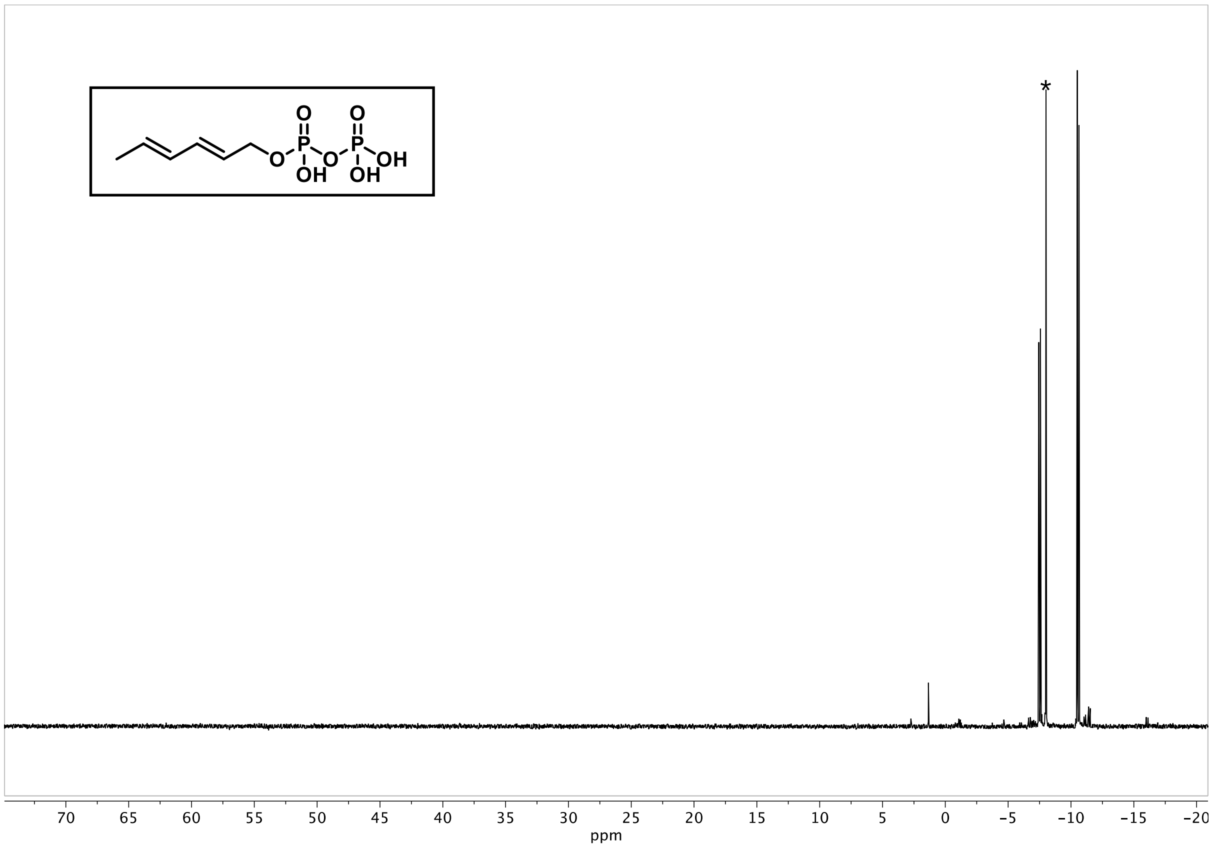

^{31}P NMR Spectrum of **16** (162 MHz, D_2O) *Denotes an impurity.

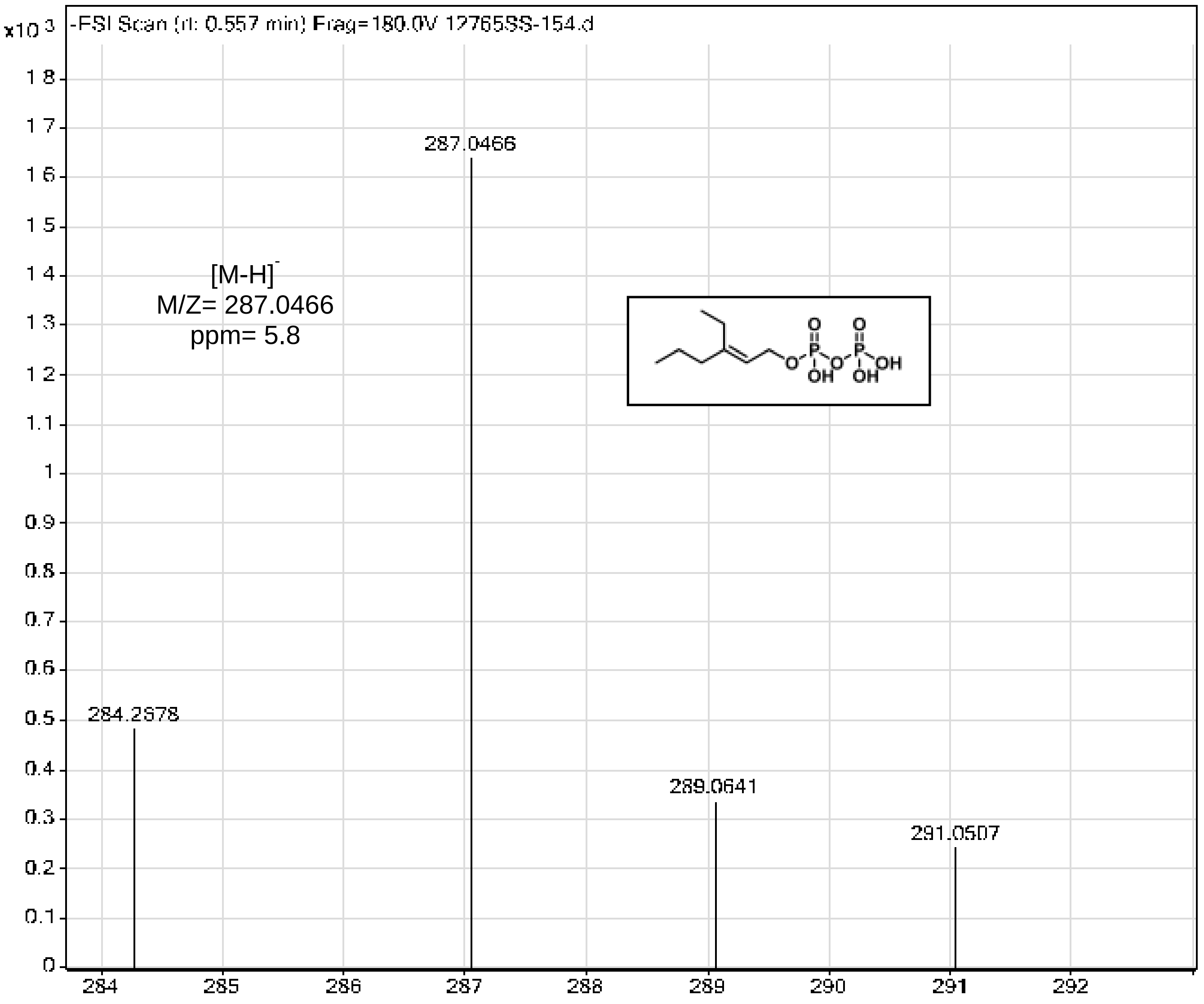

(-)-ESI-MS Spectrum of **19**

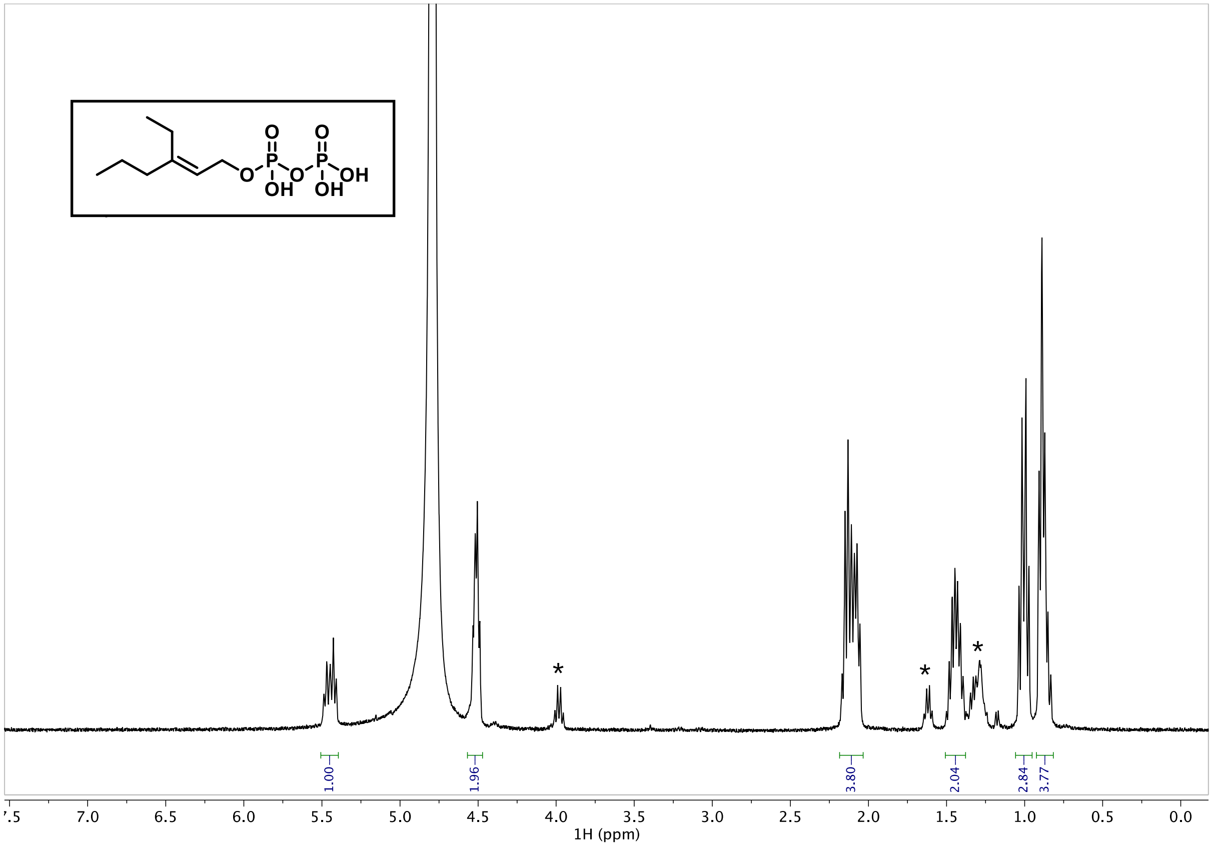

^{1}H NMR Spectrum of **19** (400 MHz, D_2O) *Denotes an impurity.

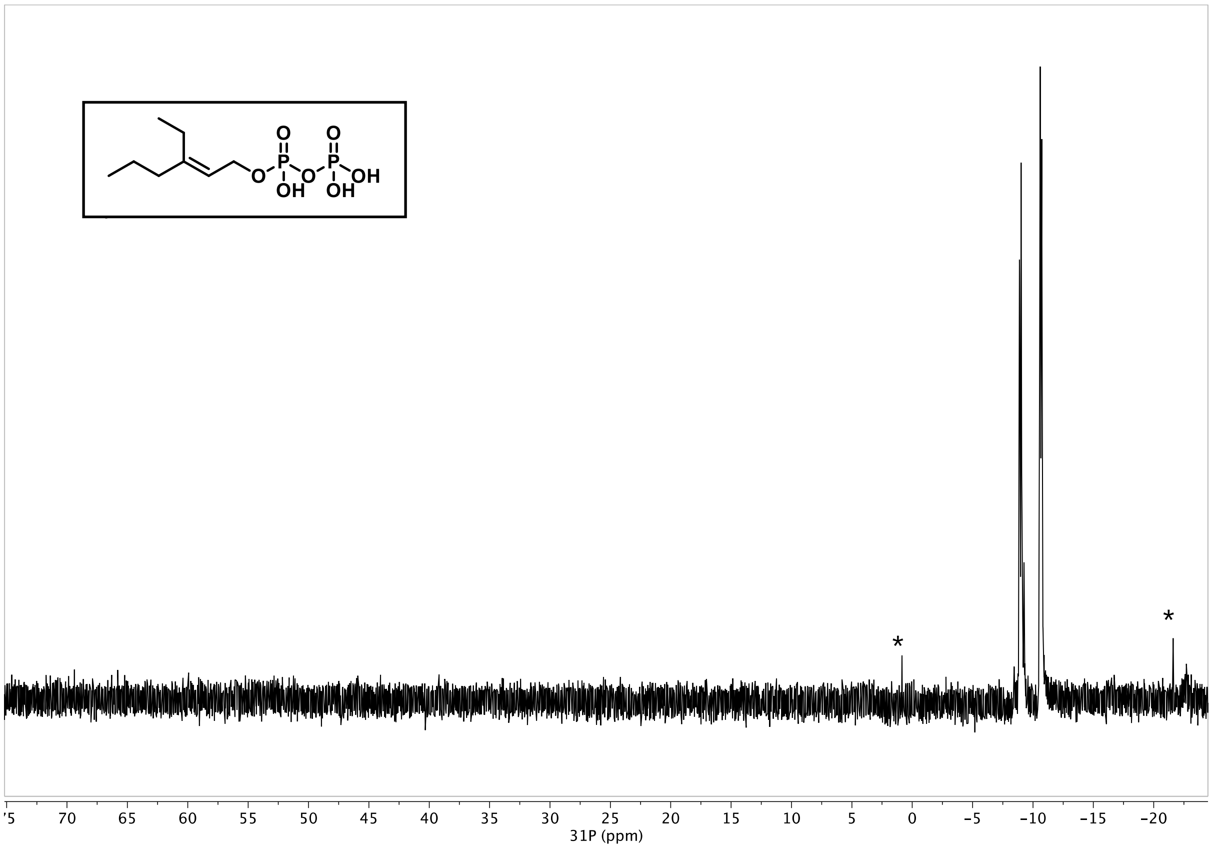

^{31}P NMR Spectrum of **19** (162 MHz, D_2O) *Denotes an impurity.

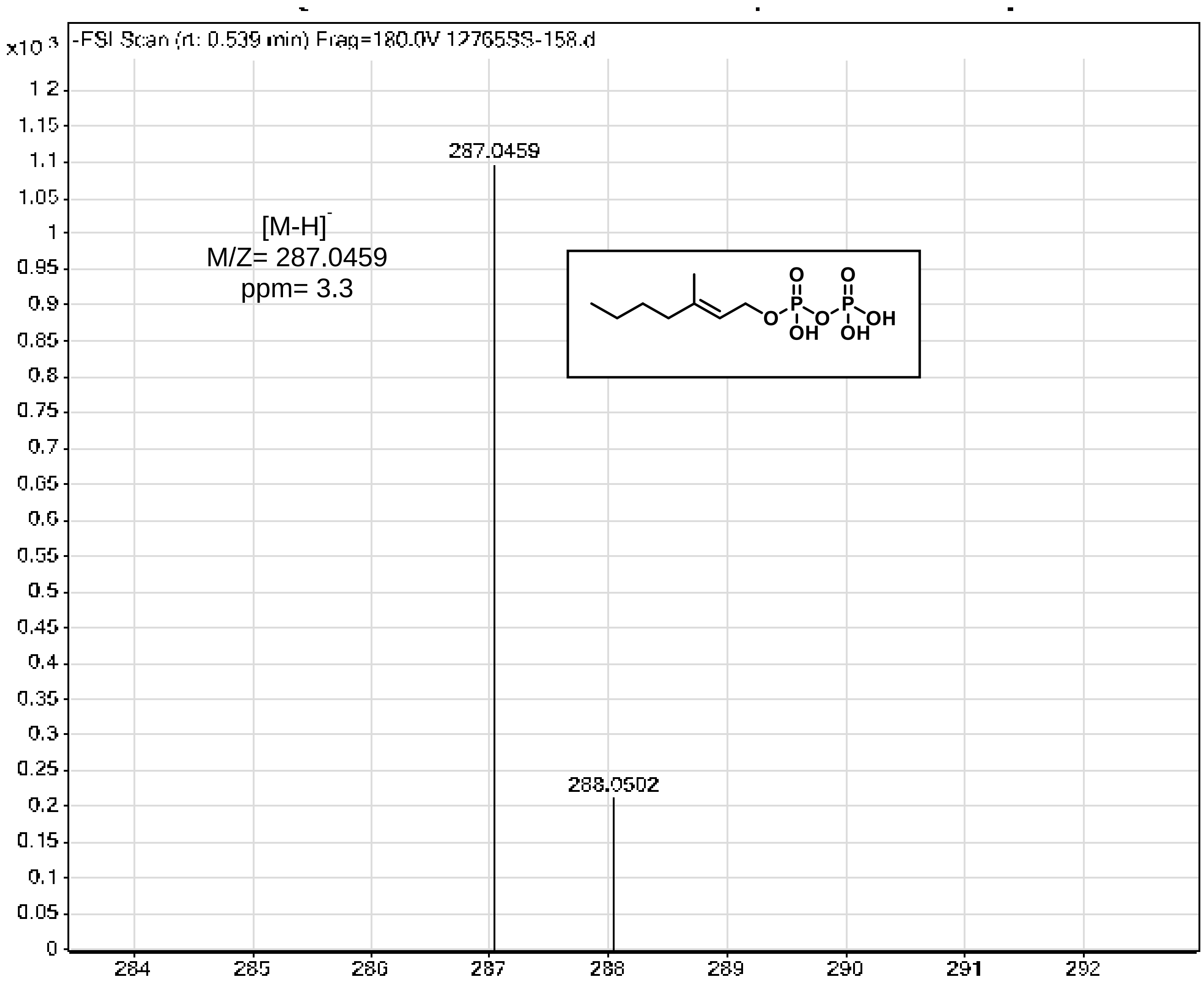

(-)-ESI-HRMS Spectrum of **21**

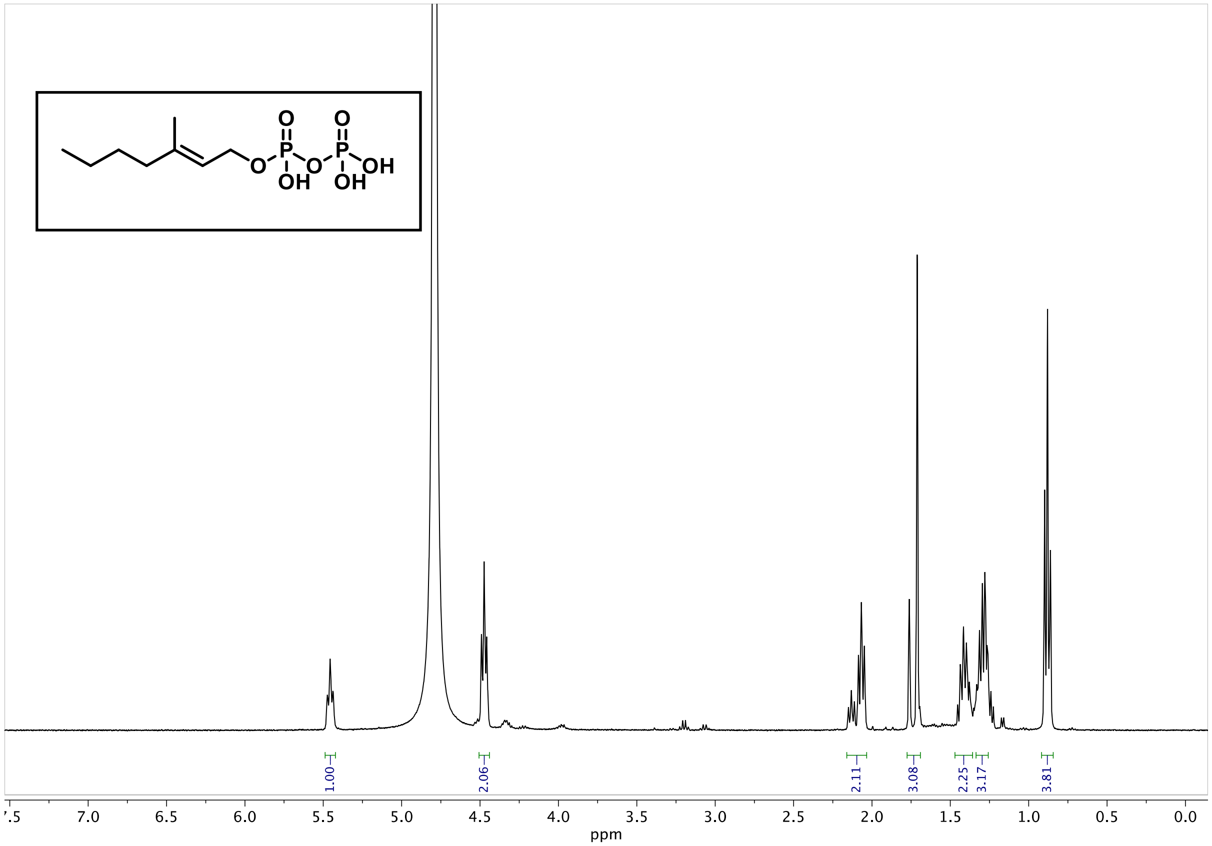

^{1}H NMR Spectrum of **21** (400 MHz, D_2O)

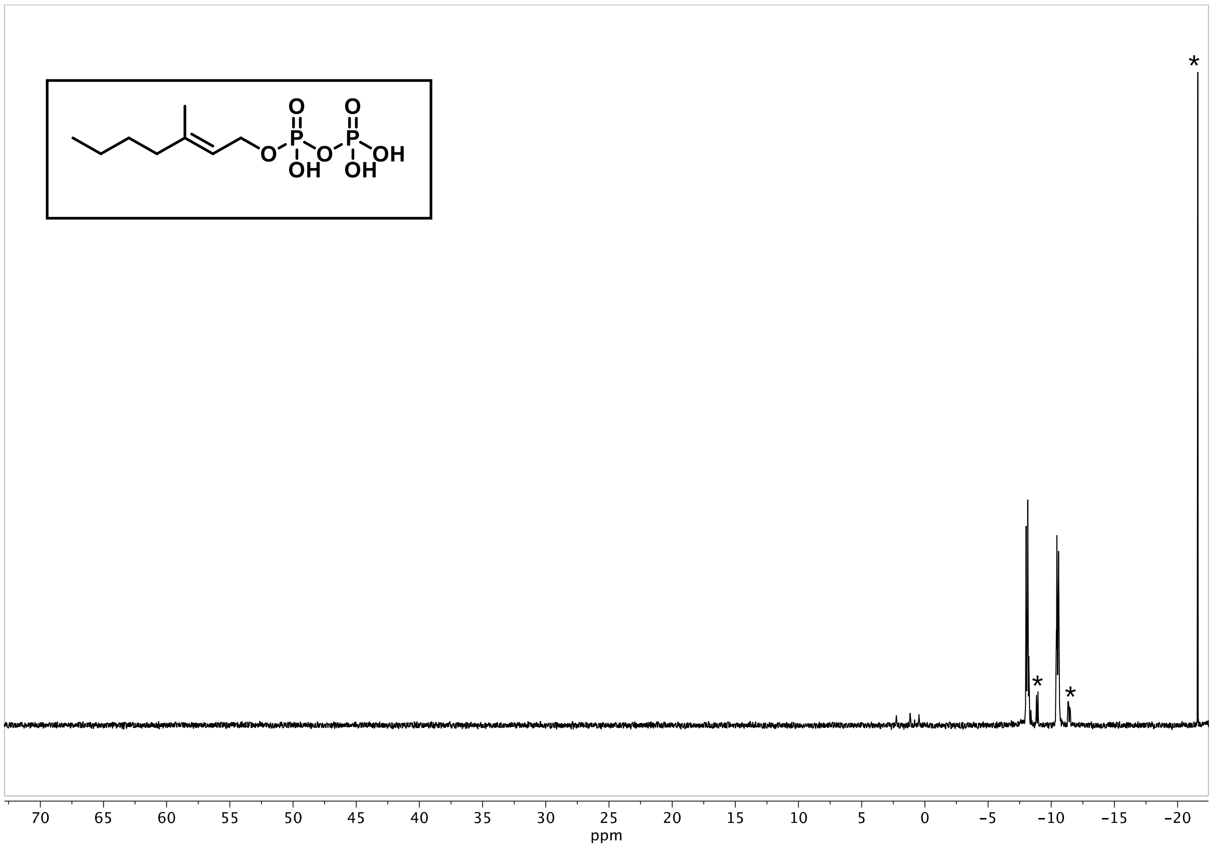

^{31}P NMR Spectrum of **21** (162 MHz, D_2O) *Denotes an impurity.

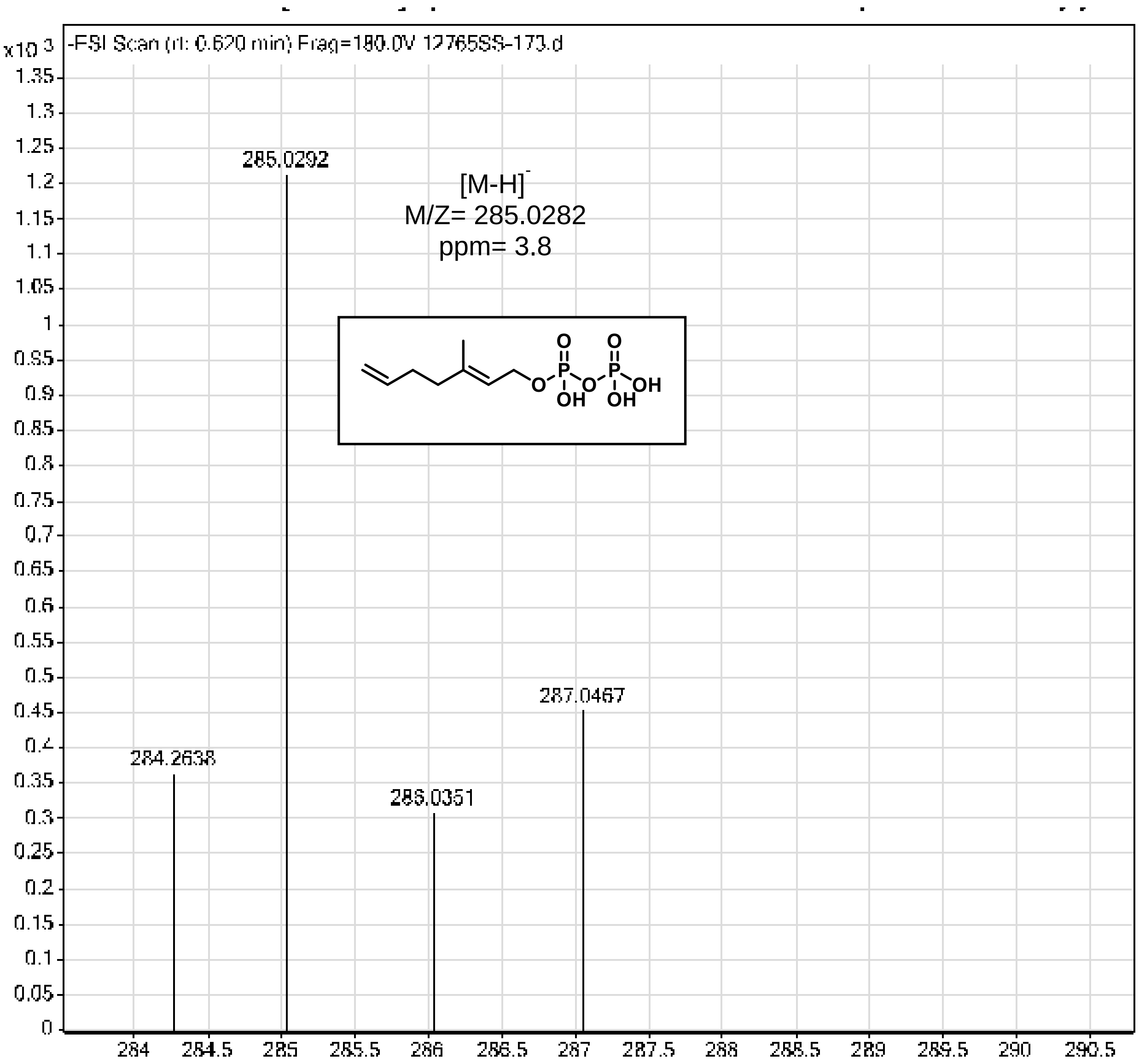

(-)-ESI-HRMS Spectrum of **22**

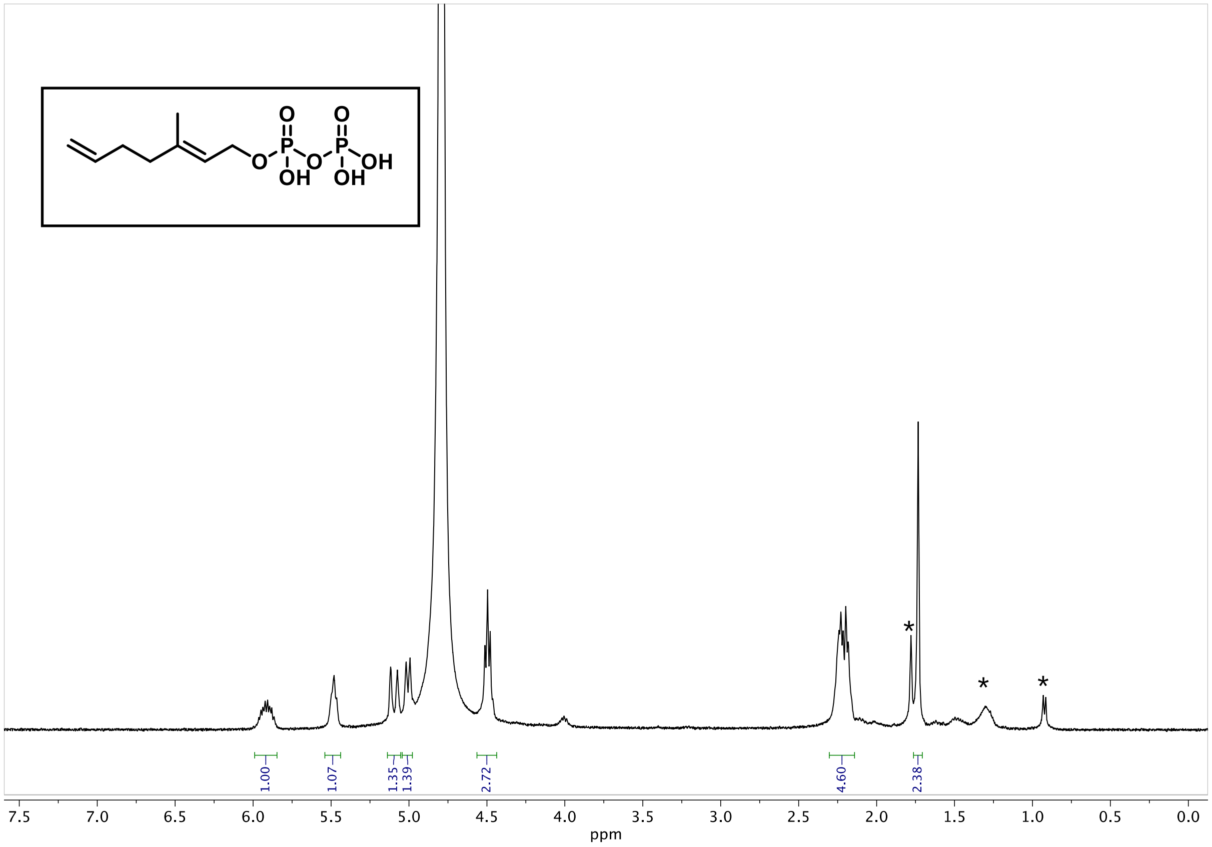

^{1}H NMR Spectrum of **22** (400 MHz, D_2O) *Denotes an impurity.

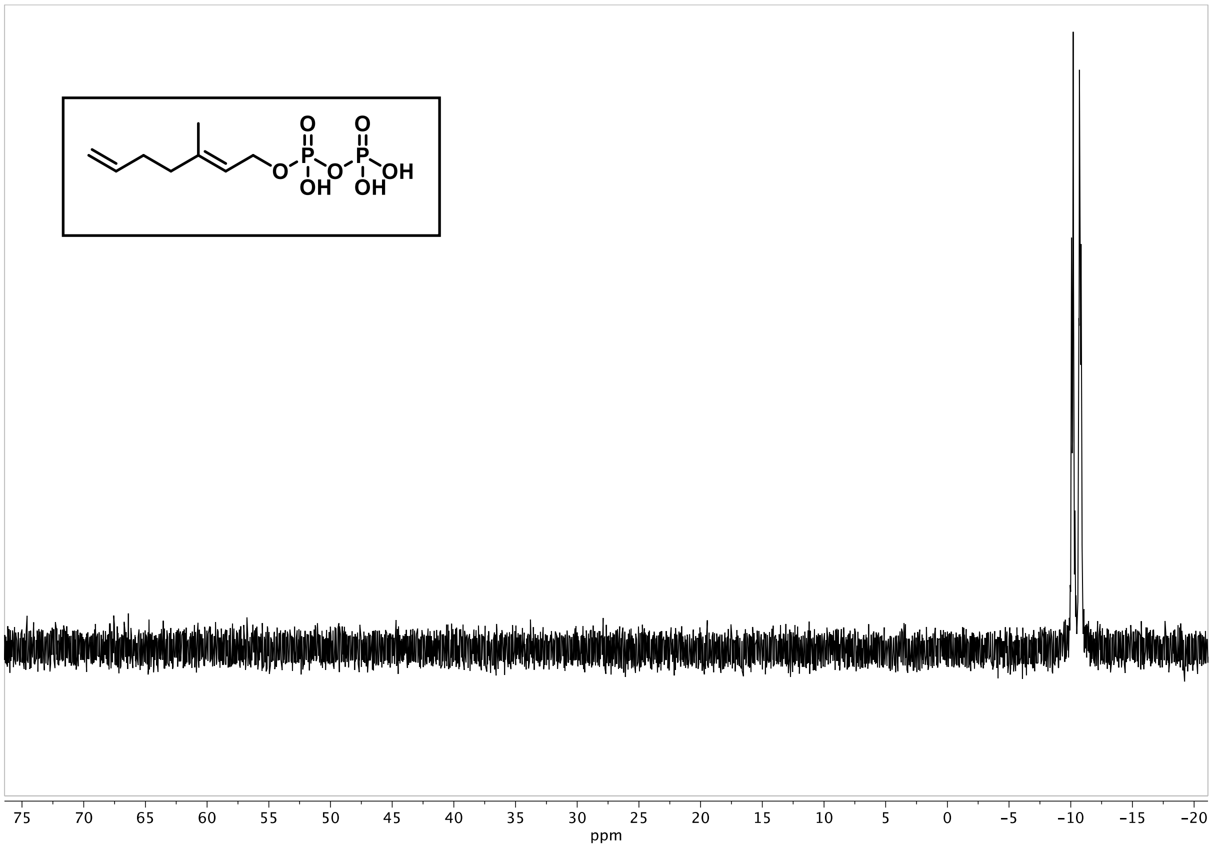

^{31}P NMR Spectrum of **22** (162 MHz, D_2O)

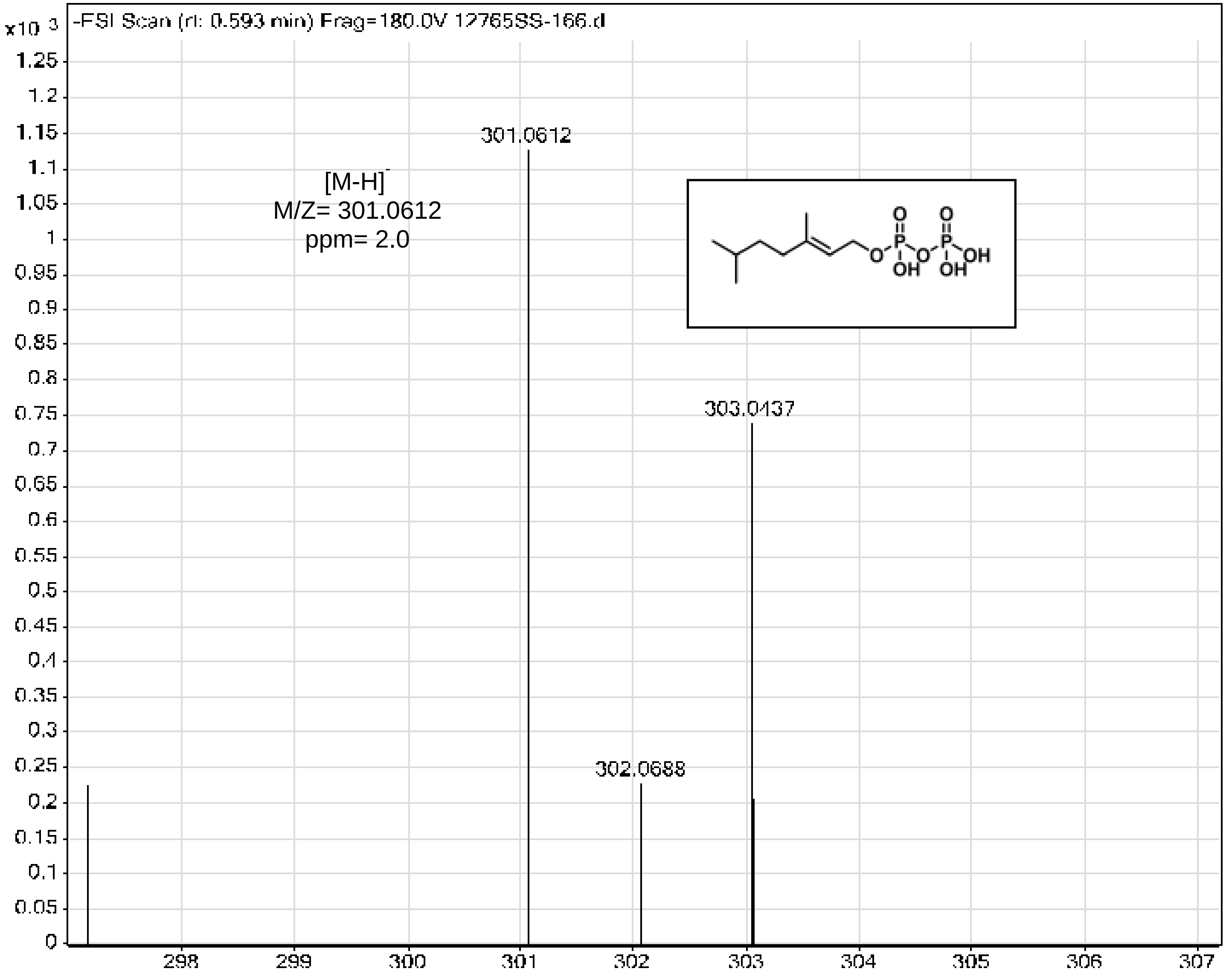

(-)-ESI-HRMS Spectrum of **23**

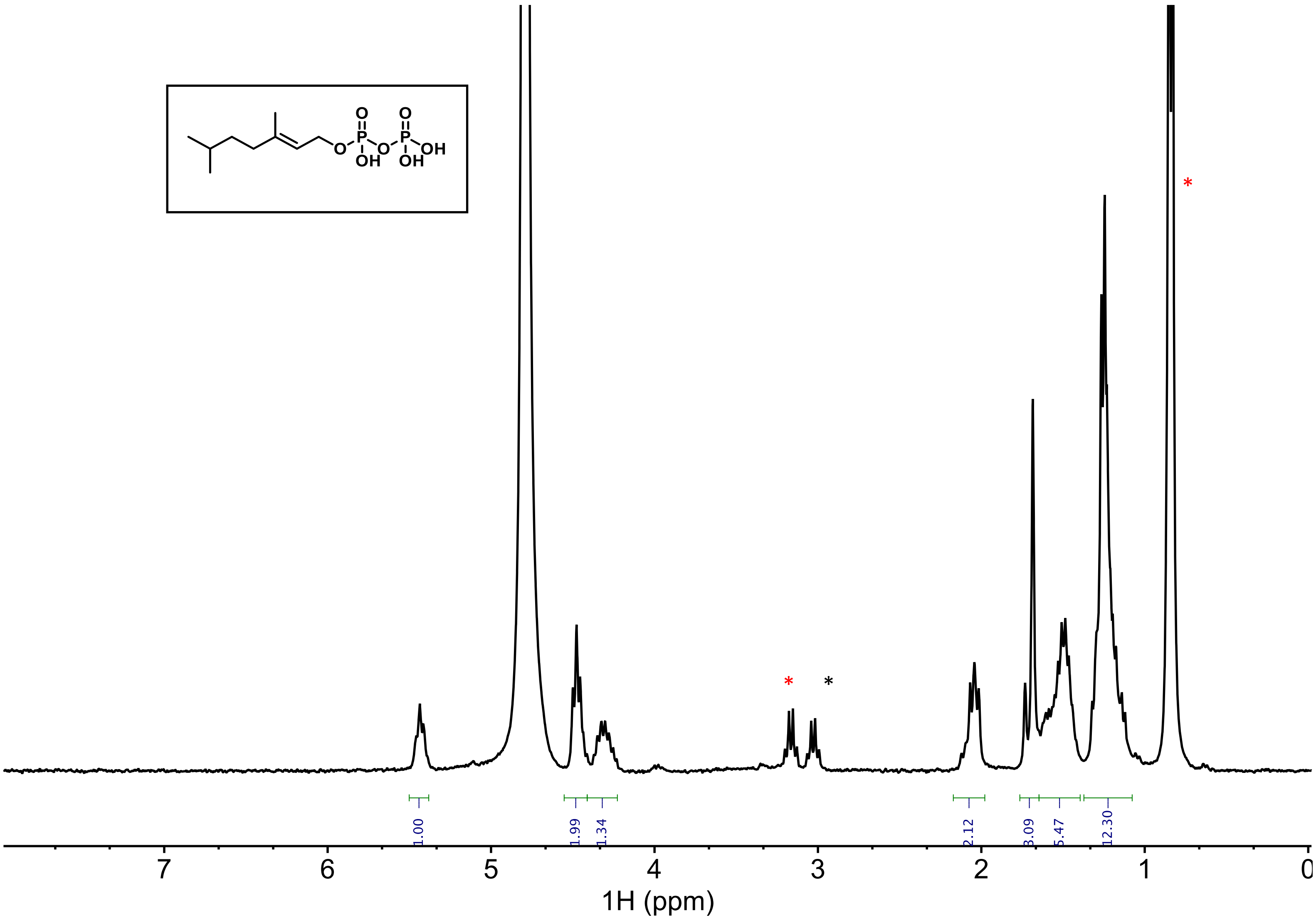

^{1}H NMR Spectrum of **23** (300 MHz, D_2O) *Denotes an impurity. *Denotes TEAP counterions.

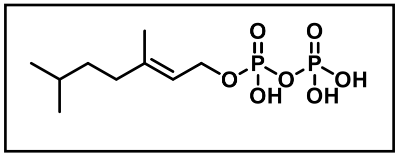

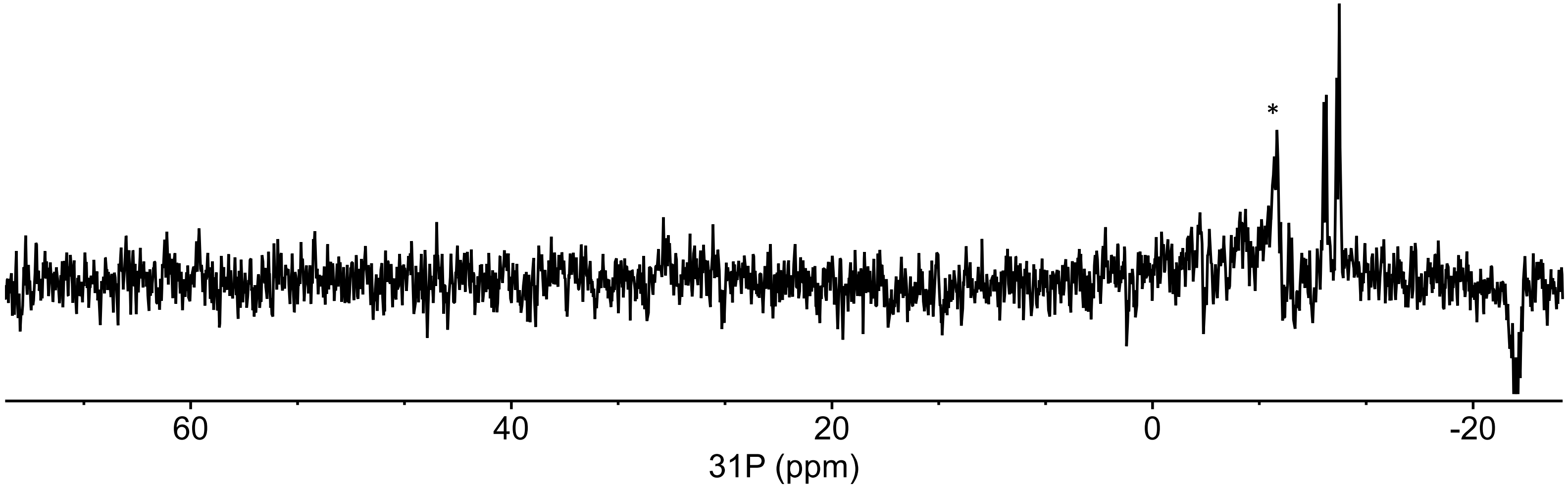

^{31}P NMR Spectrum of **23** (122 MHz, D_2O) *Denotes an impurity.

(-)-ESI-MS Spectrum of **27**

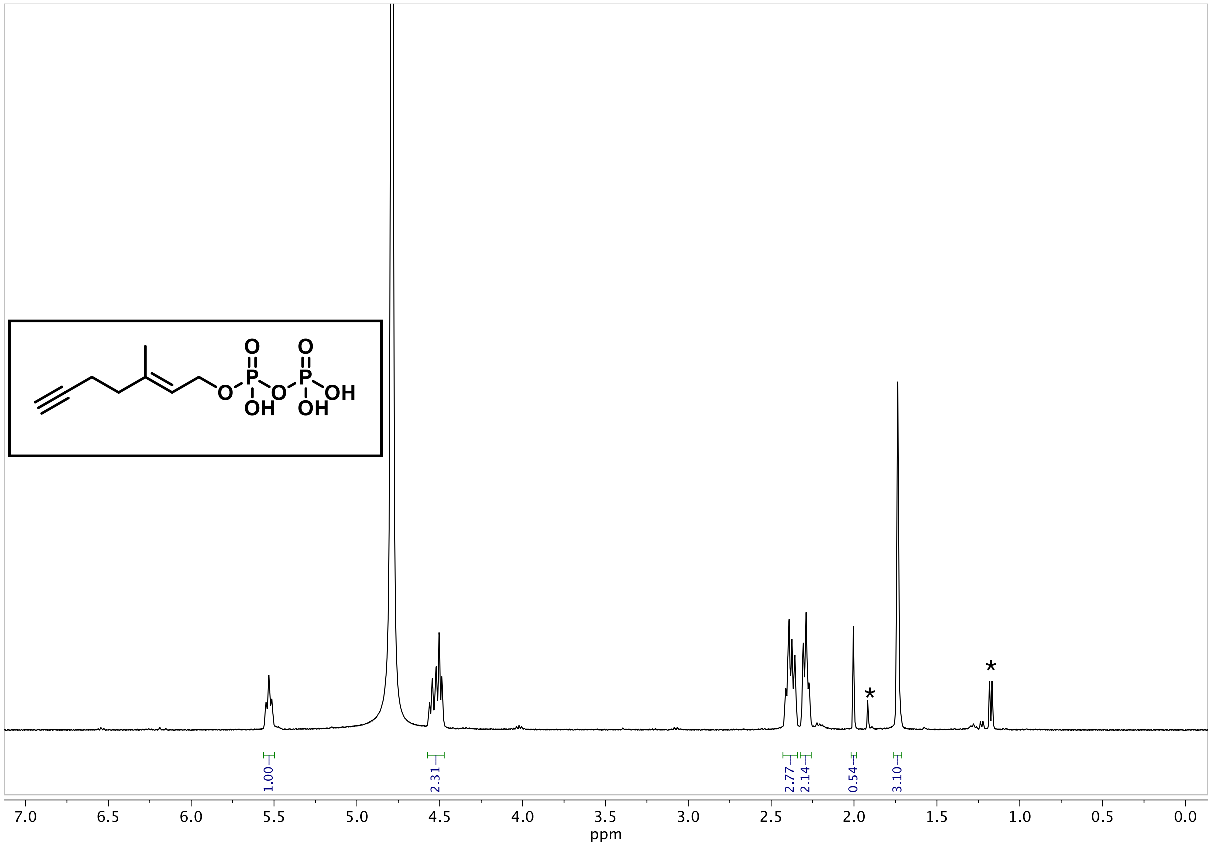

^{1}H NMR Spectrum of **27** (400 MHz, D_2O) *Denotes an impurity.

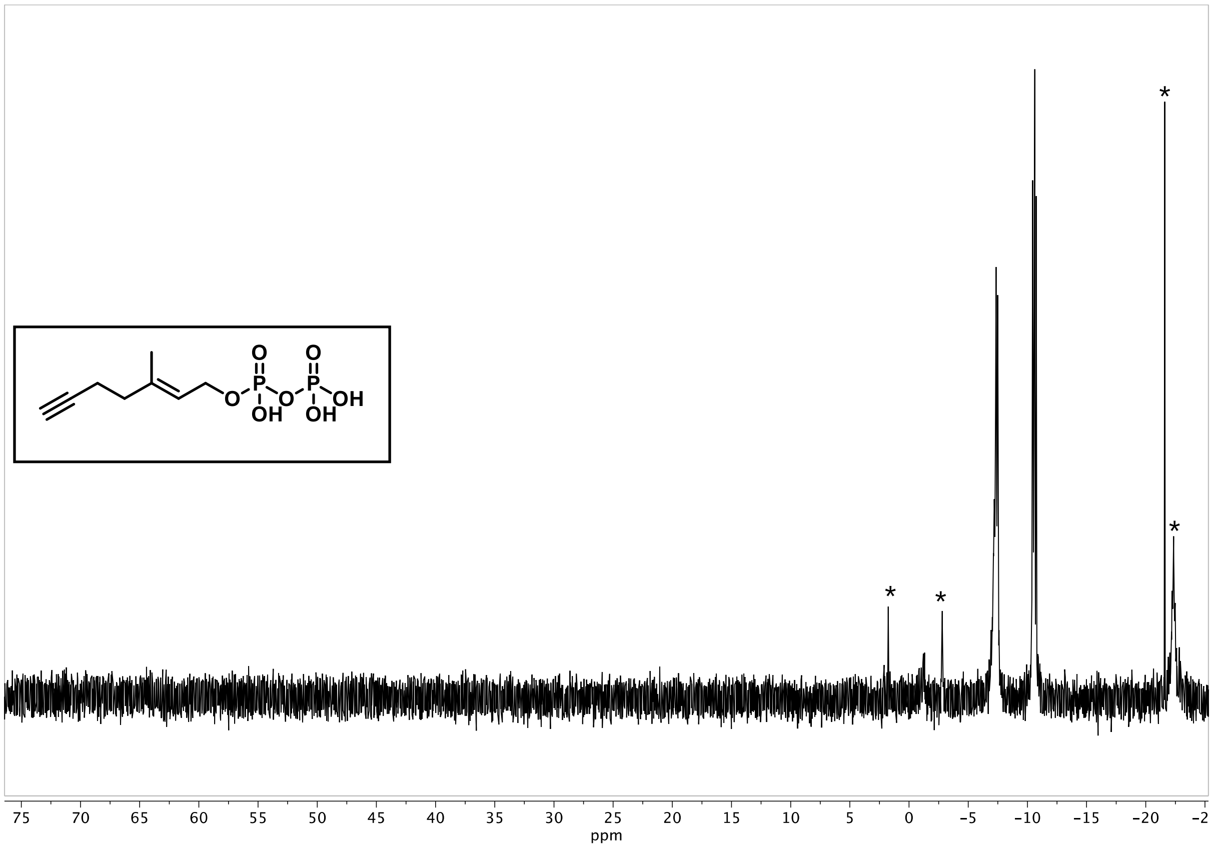

^{31}P NMR Spectrum of **27** (162 MHz, D_2O) *Denotes an impurity.

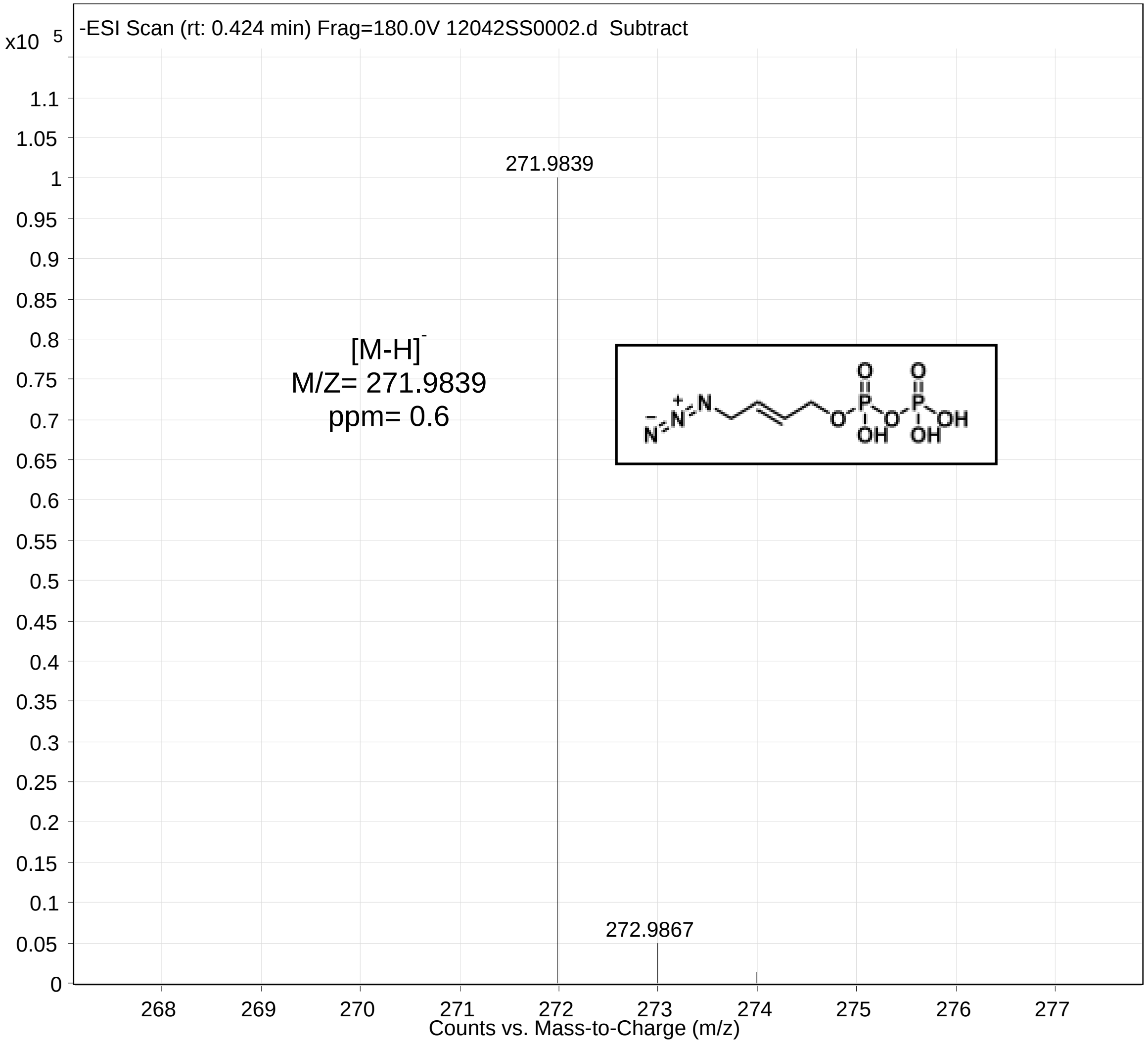

(-)-ESI-HRMS Spectrum of **28**

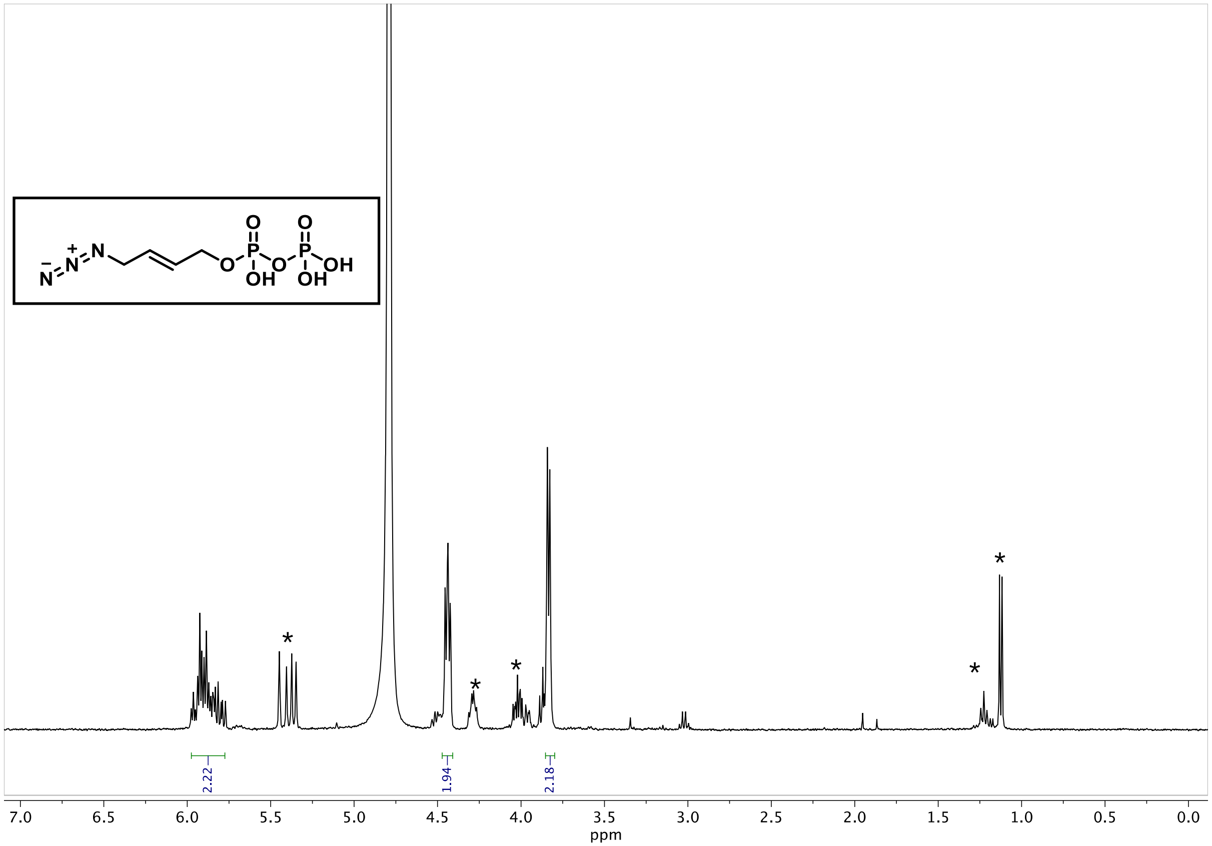

^{1}H NMR Spectrum of **28** (400 MHz, D_2O) *Denotes an impurity.

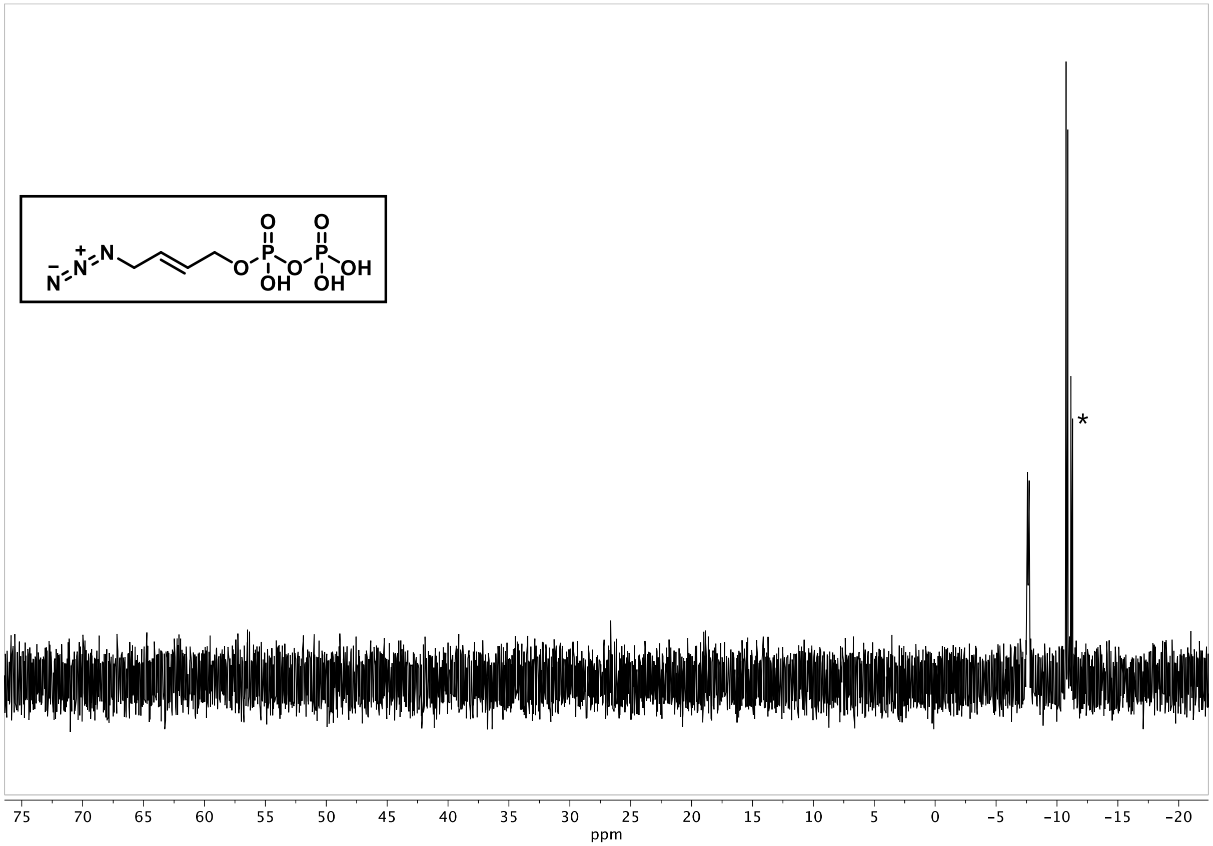

^{31}P NMR Spectrum of **28** (162 MHz, D_2O) *Denotes an impurity.

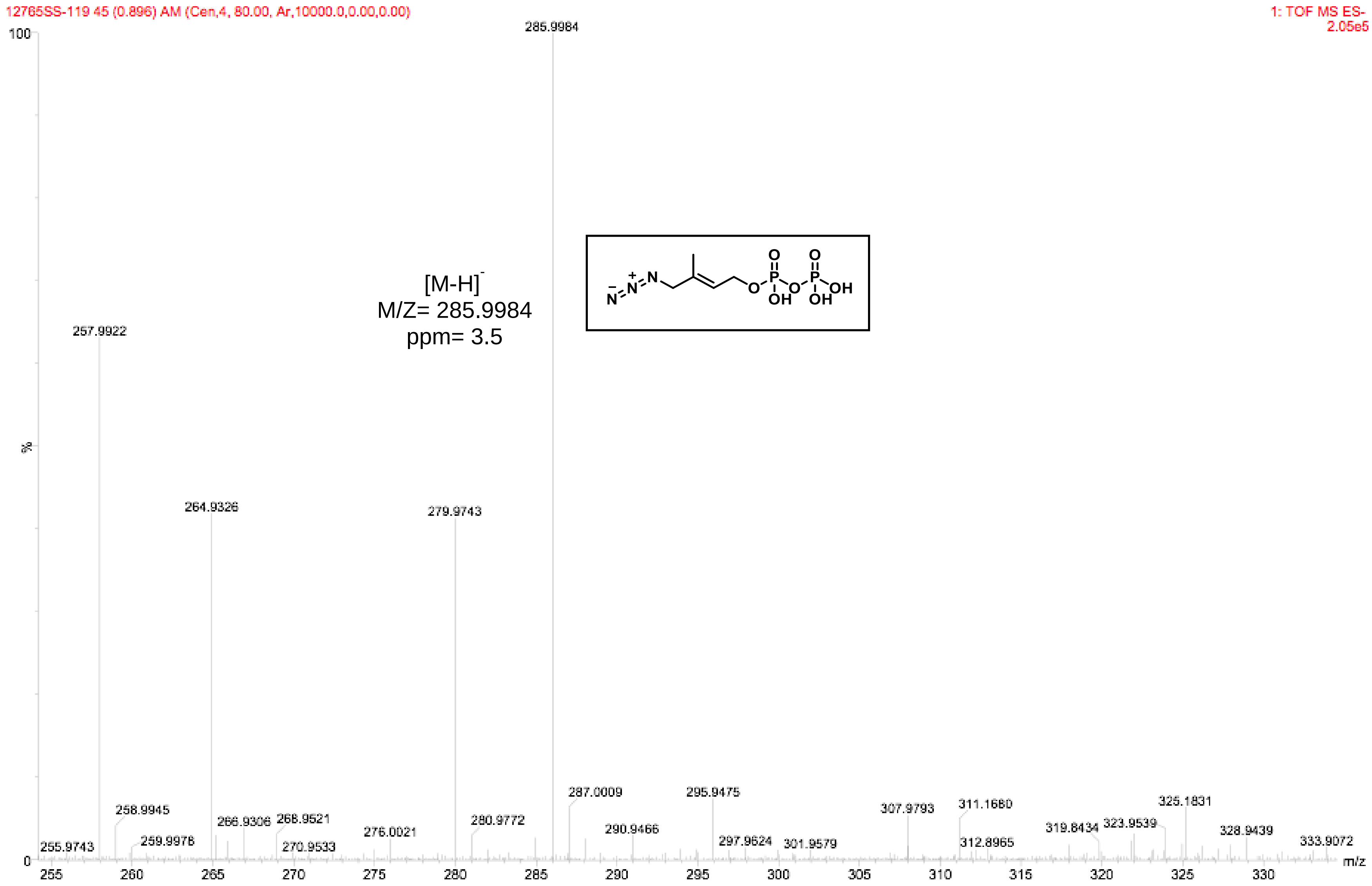

(-)-ESI-HRMS Spectrum of **29**

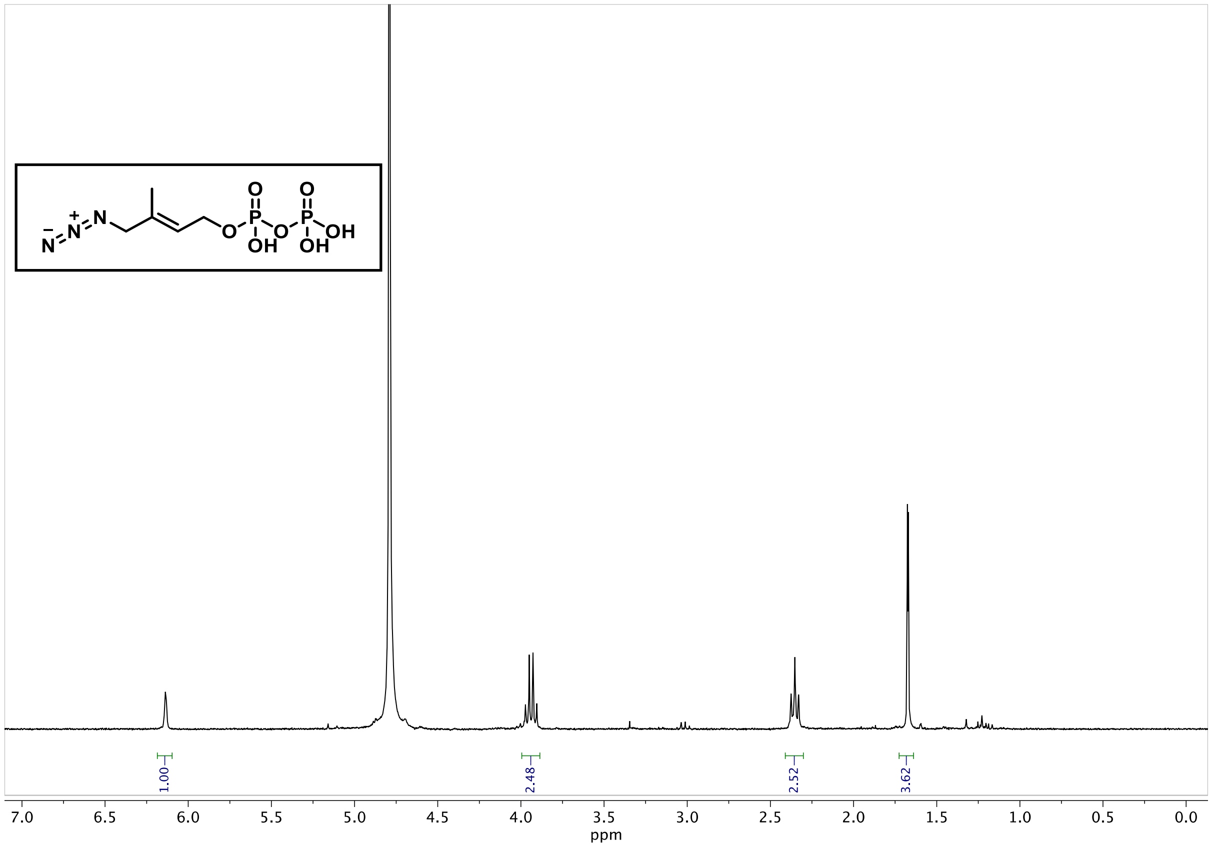

^{1}H NMR Spectrum of **29** (300 MHz, D_2O)

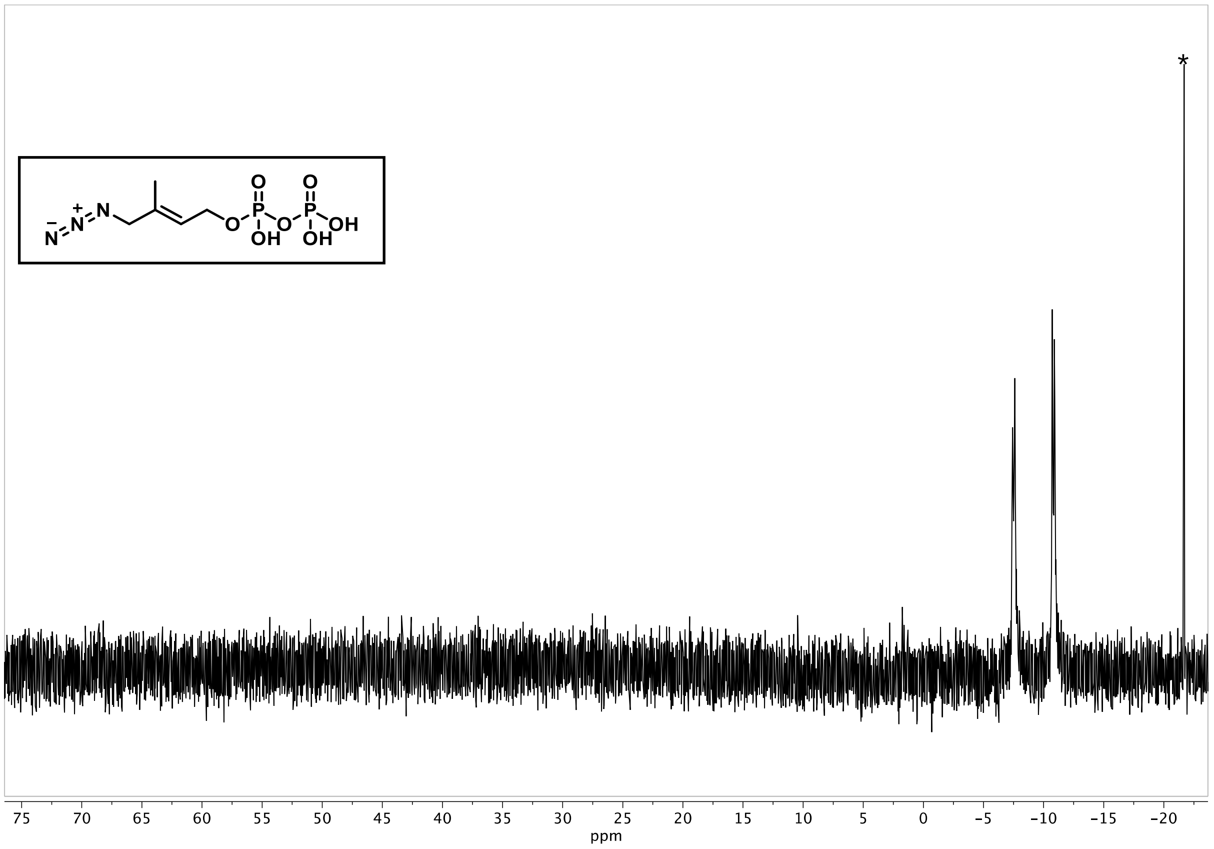

^{31}P NMR Spectrum of **29** (122 MHz, D_2O) *Denotes an impurity.

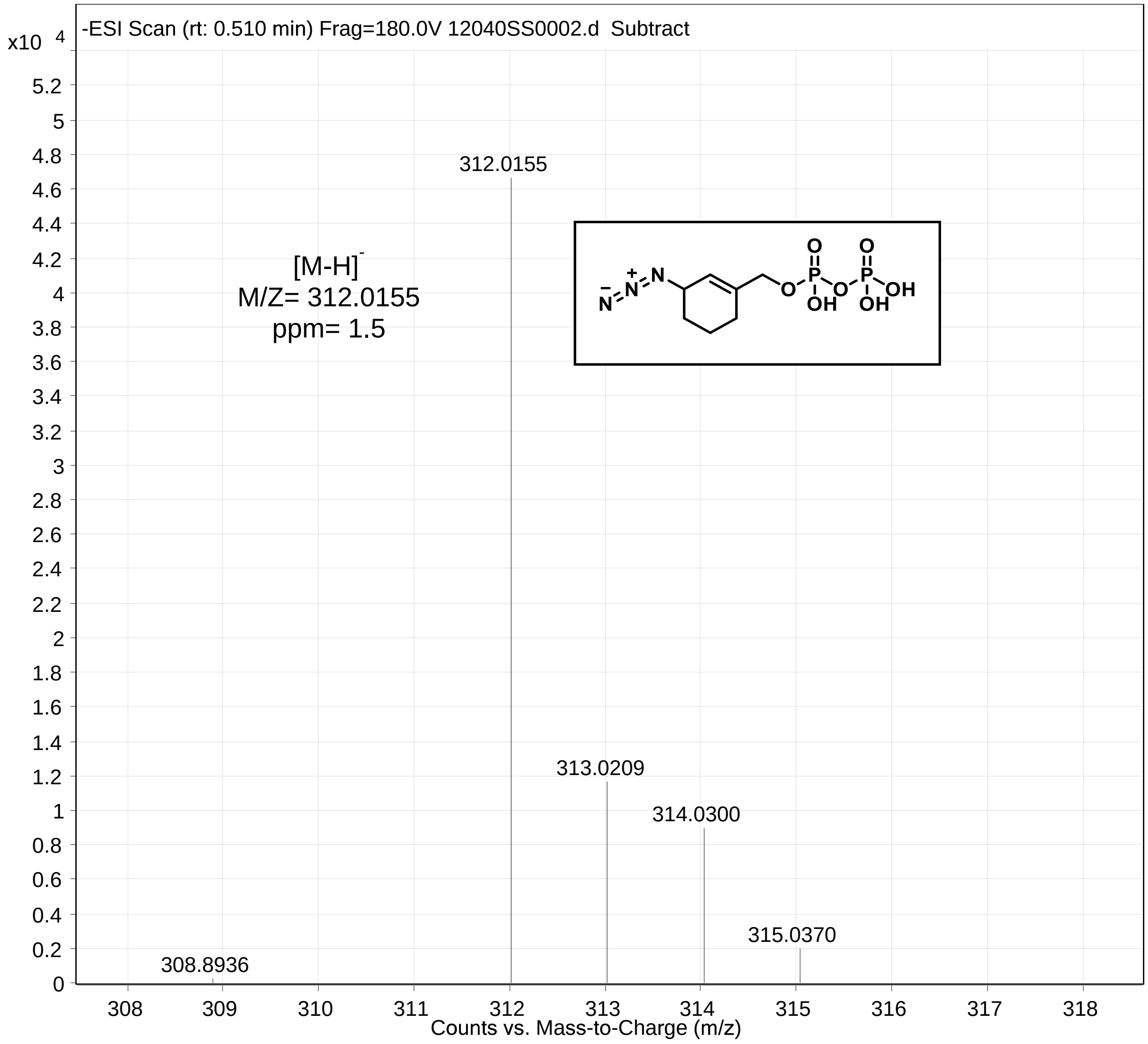

(-)-ESI-HRMS Spectrum of **30**

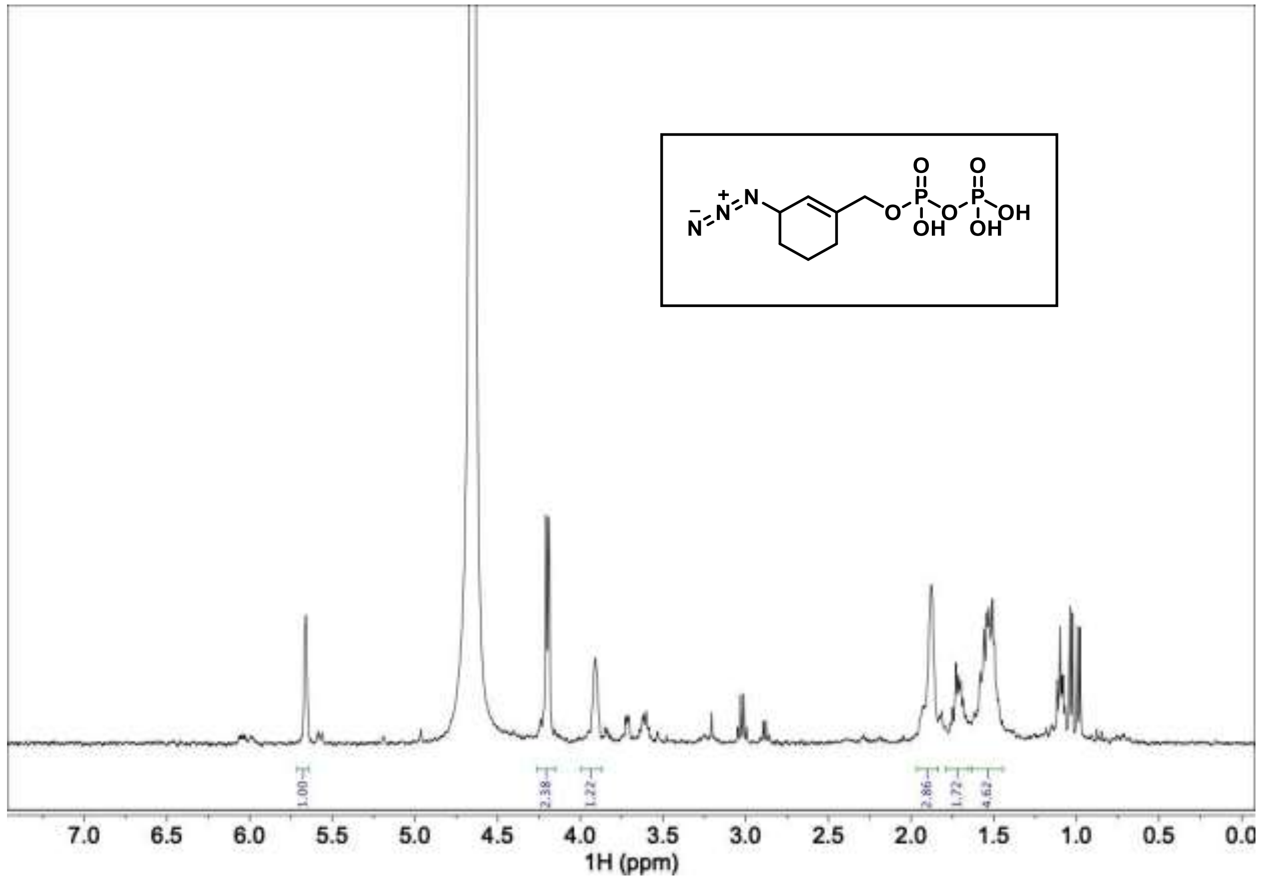

^{1}H NMR Spectrum of **30** (400 MHz, D_2O)

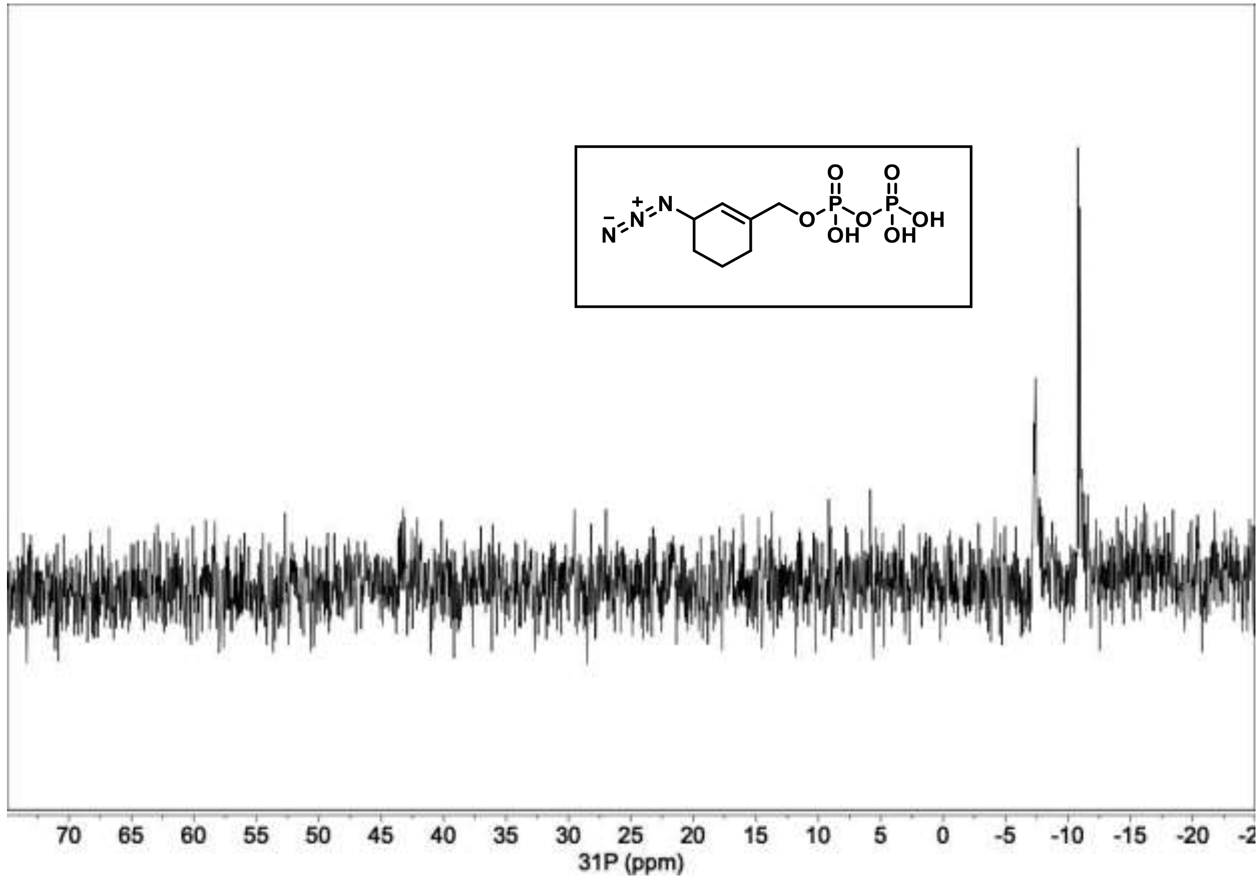

^{31}P NMR Spectrum of **30** (162 MHz, D_2O)

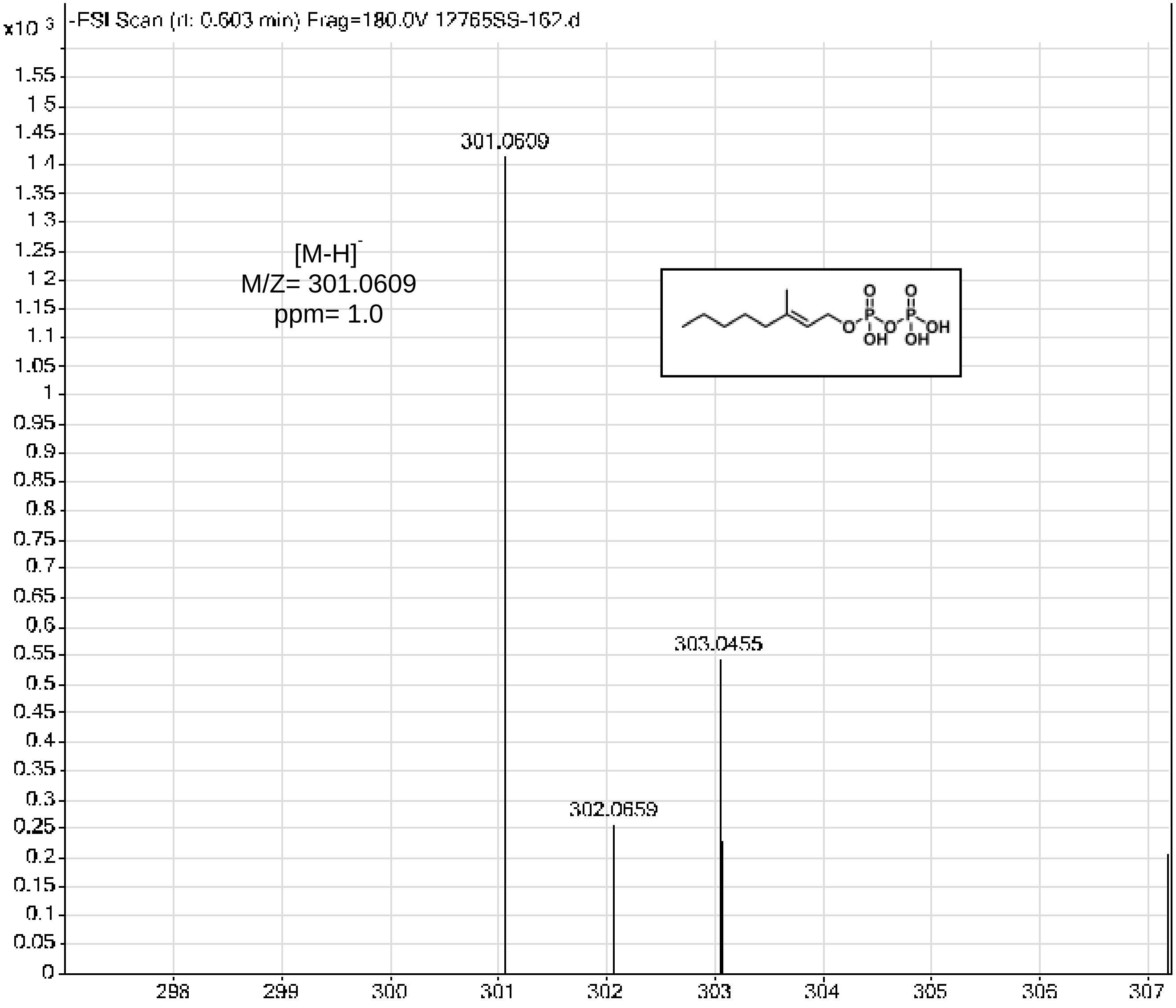

(-)-ESI-HRMS Spectrum of **31**

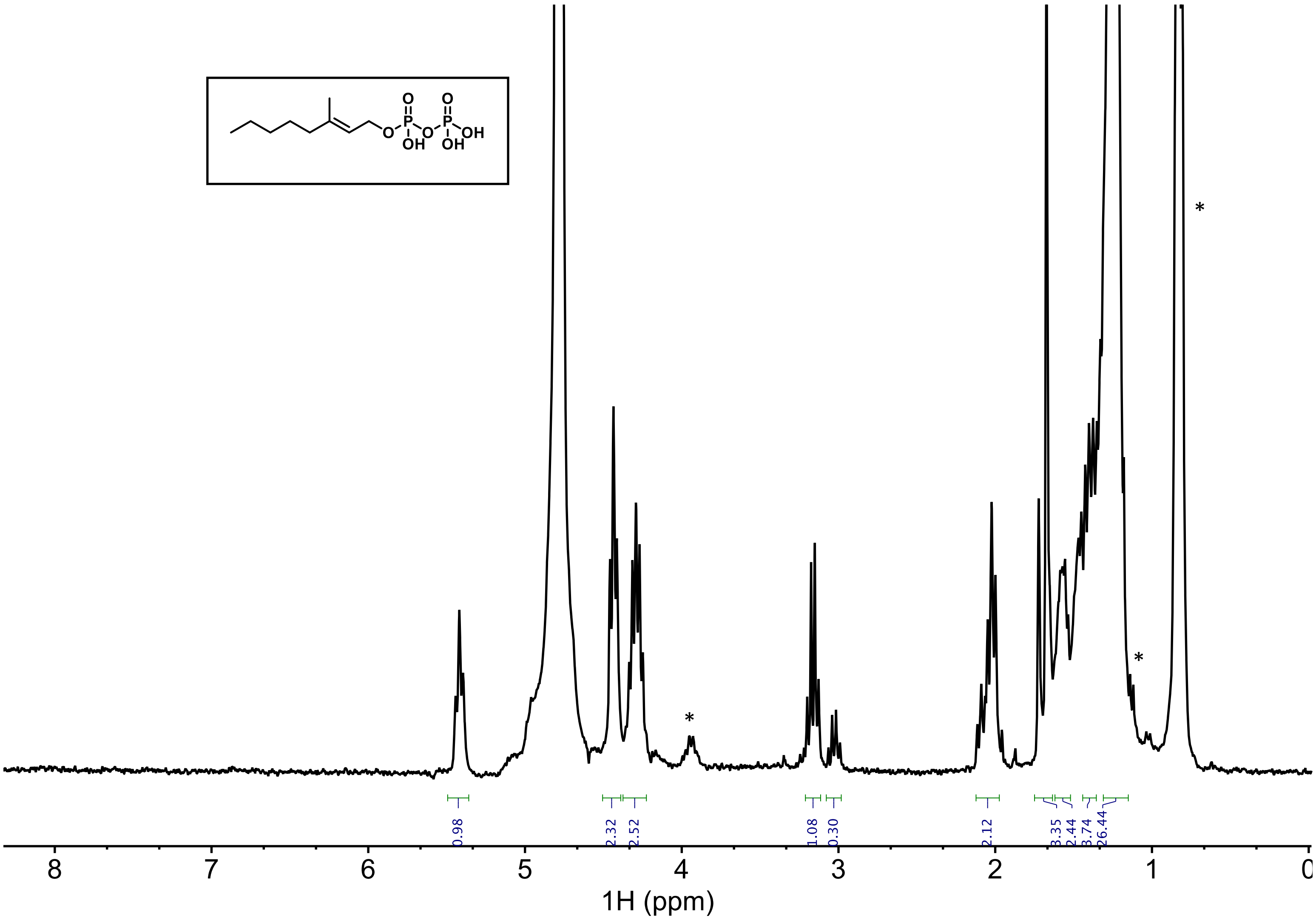

^{1}H NMR Spectrum of **31** (300 MHz, D_2O) *Denotes an impurity.

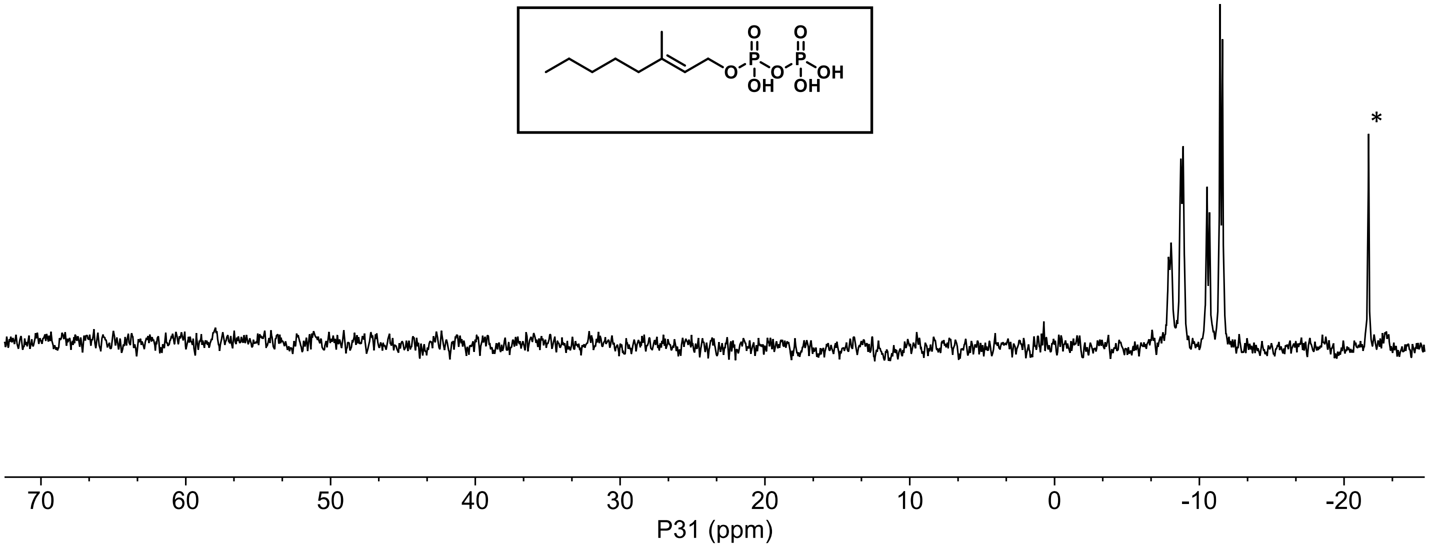

^{31}P NMR Spectrum of **31** (122 MHz, D_2O) *Denotes an impurity. Additionally, doubles of **31** is due to a *trans-cis* conformation.

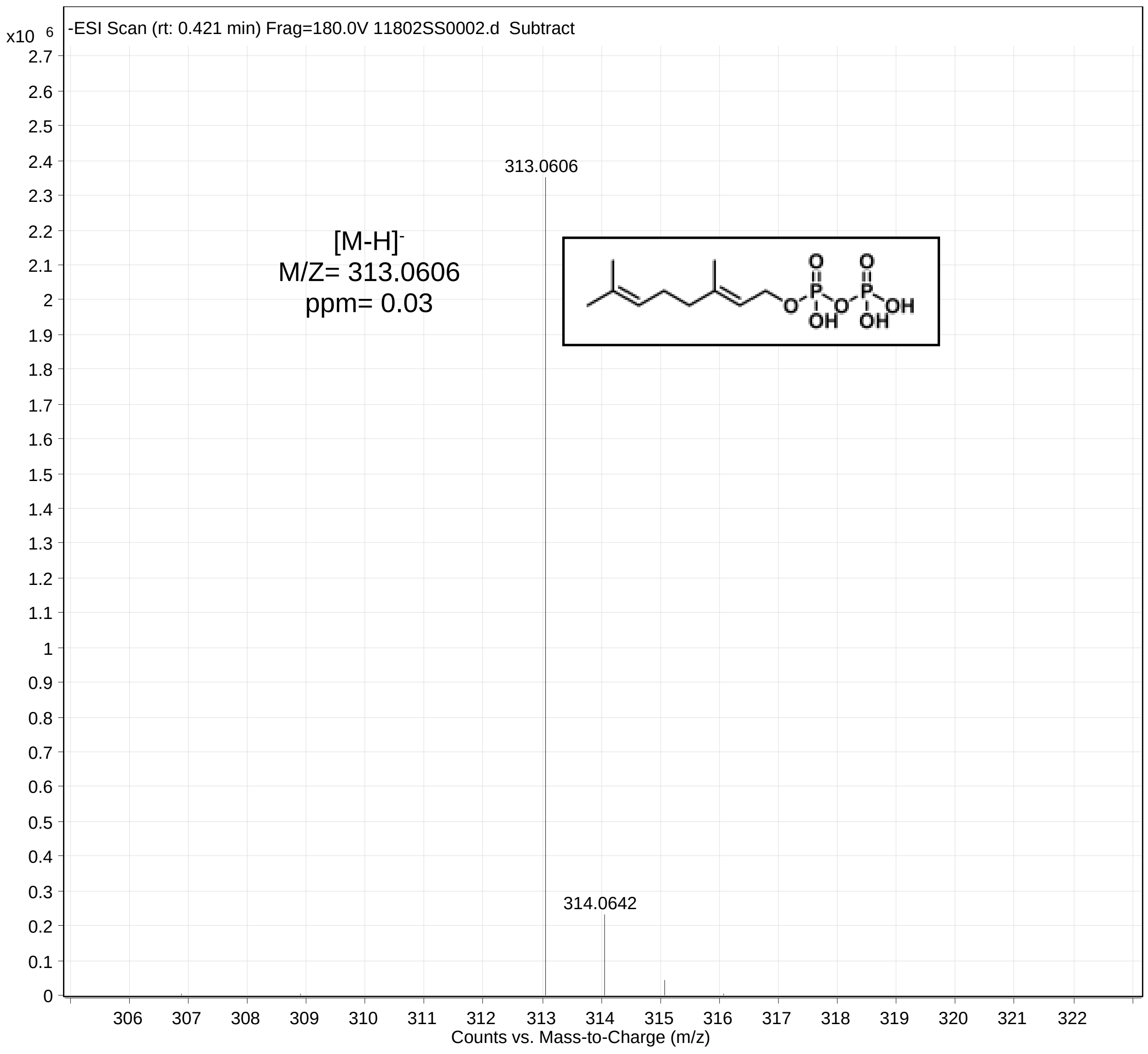

(-)-ESI-HRMS Spectrum of **32**

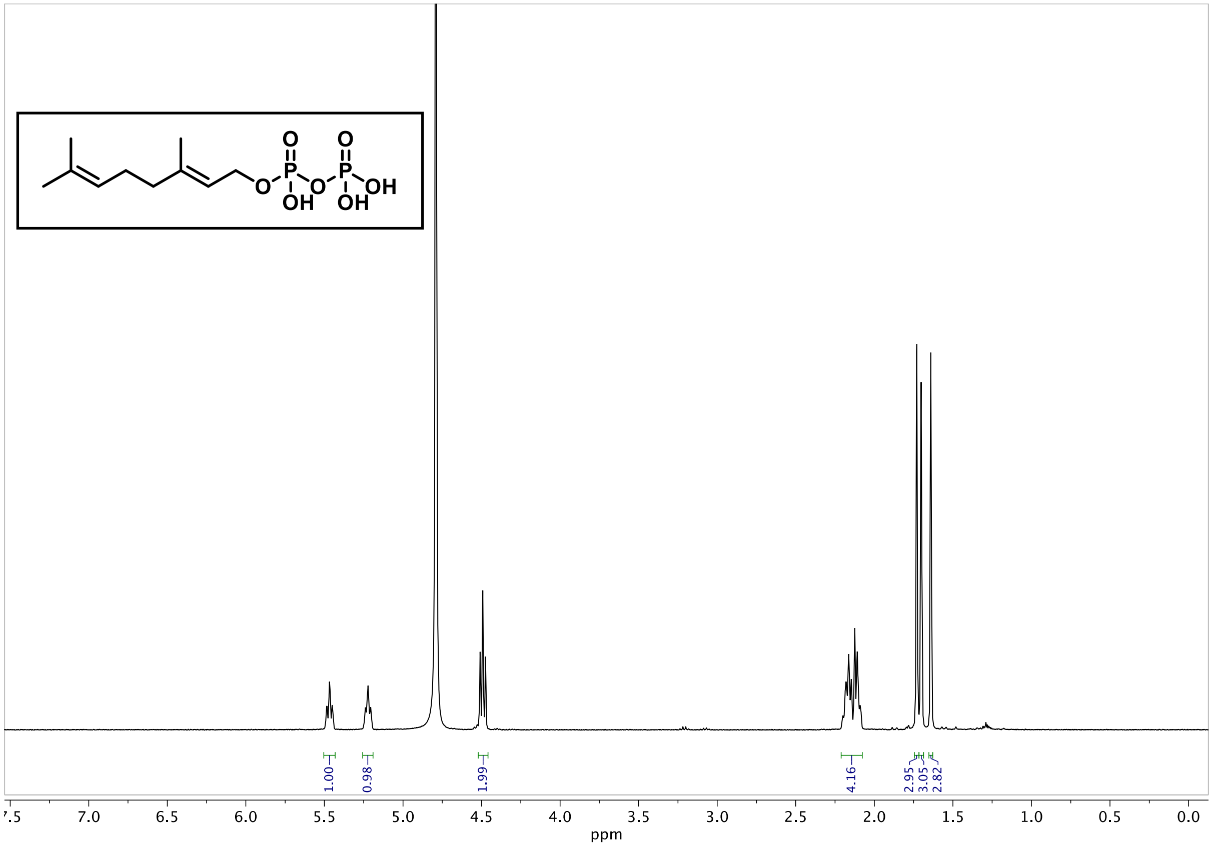

^{1}H NMR Spectrum of **32** (400 MHz, D_2O)

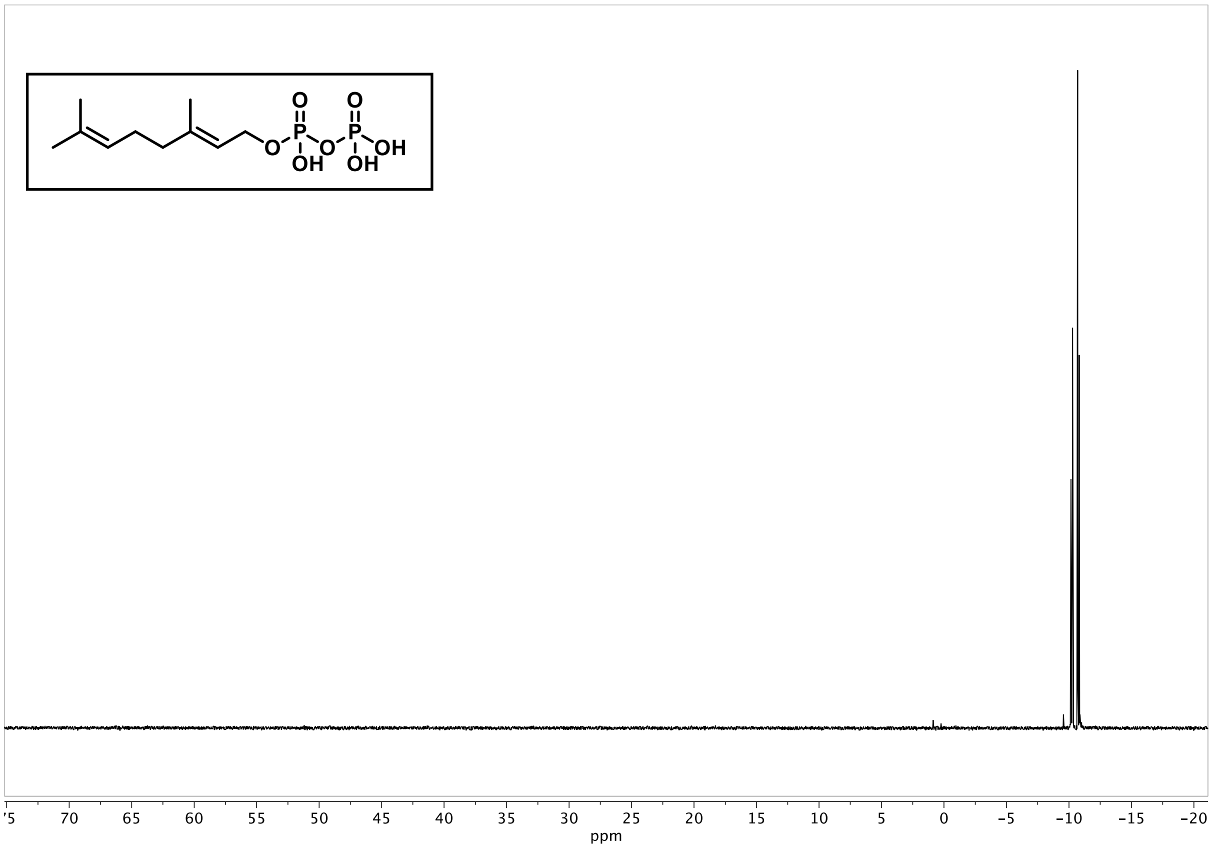

^{31}P NMR Spectrum of **32** (162 MHz, D_2O)

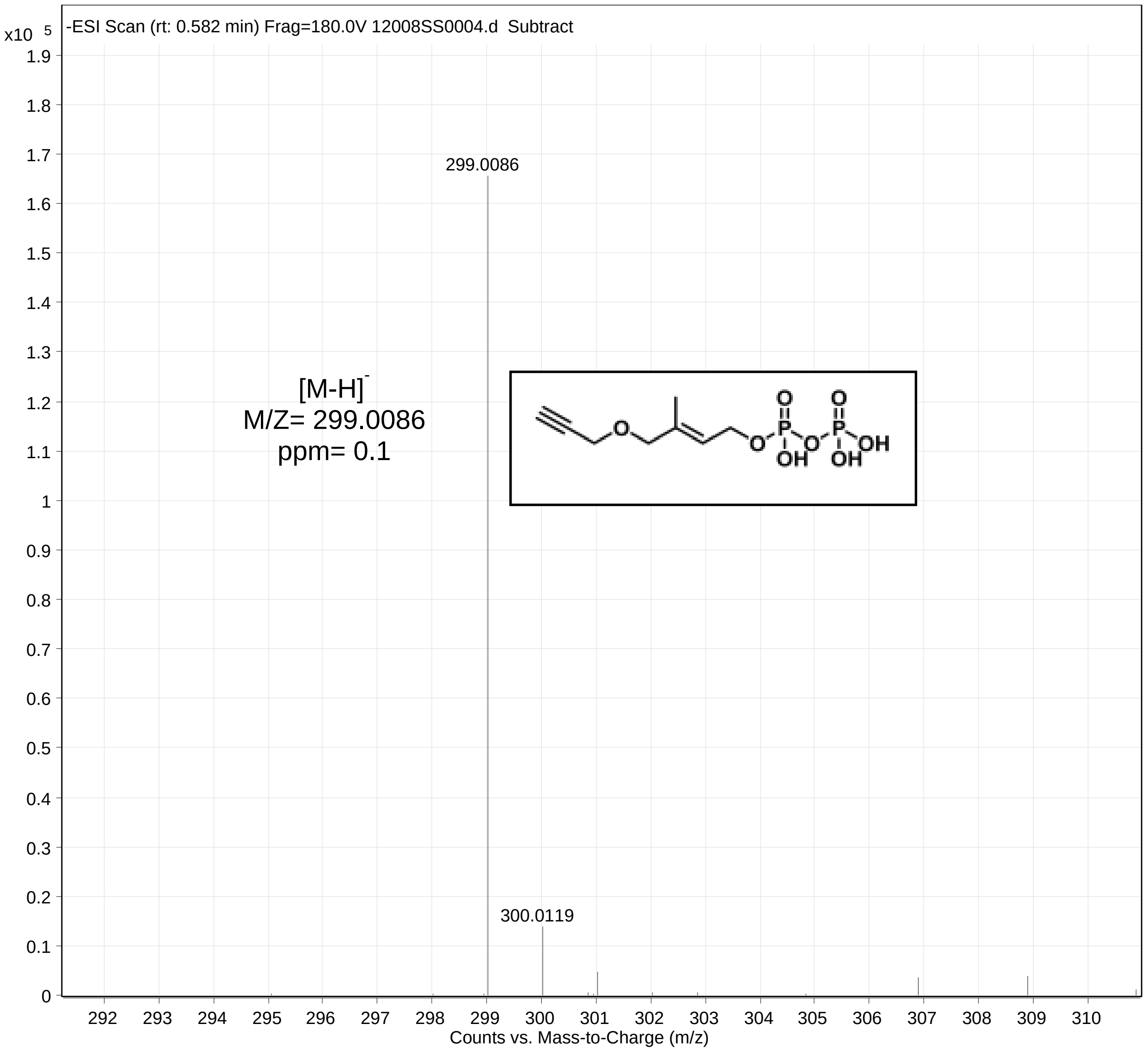

(-)-ESI-HRMS Spectrum of **33**

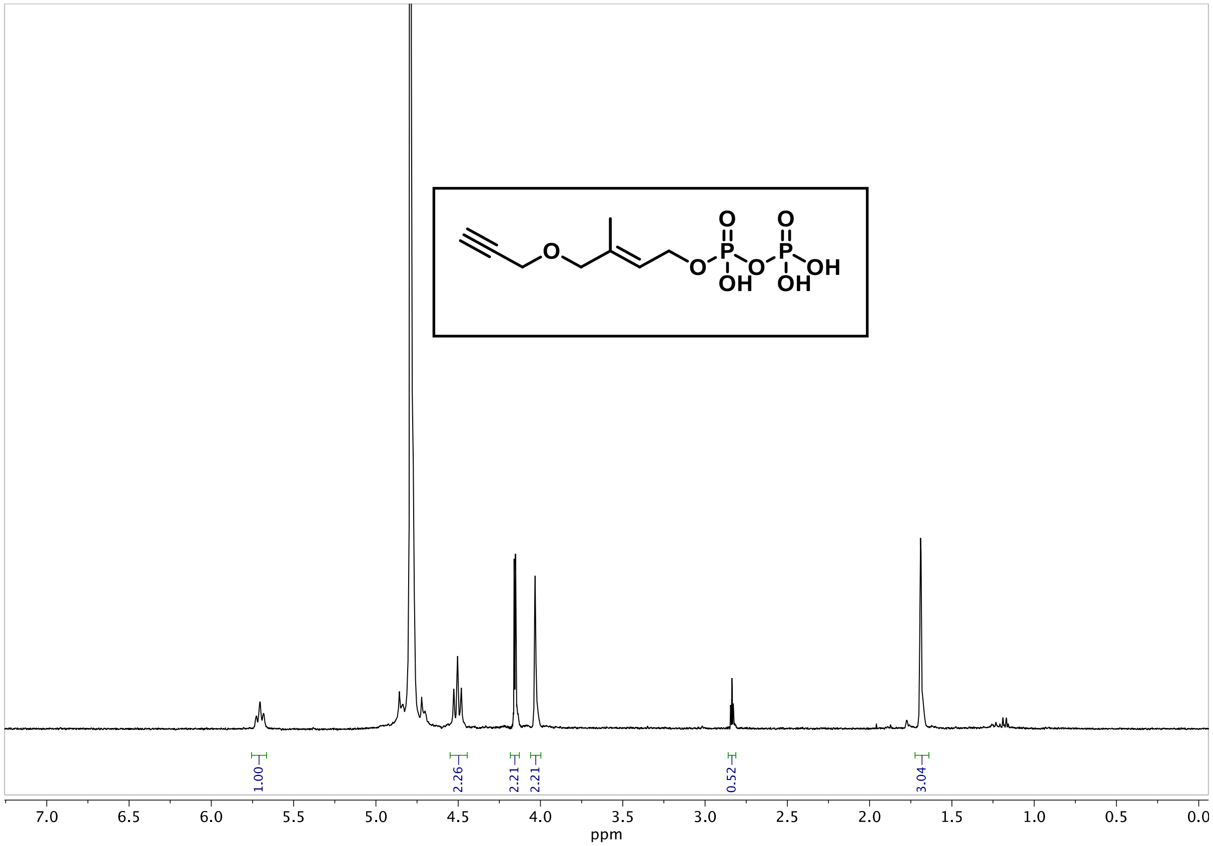

^{1}H NMR Spectrum of **33** (300 MHz, D_2O)

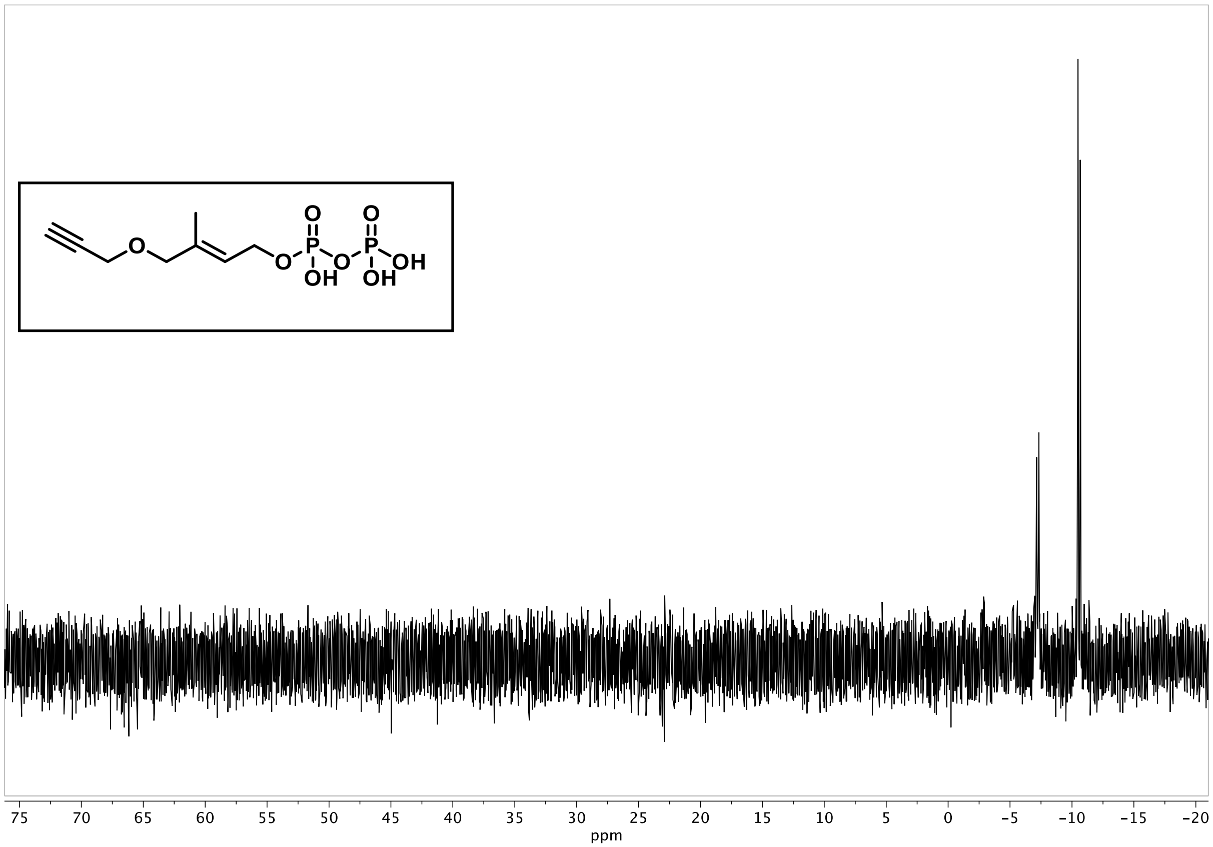

^{31}P NMR Spectrum of **33** (122 MHz, D_2O).

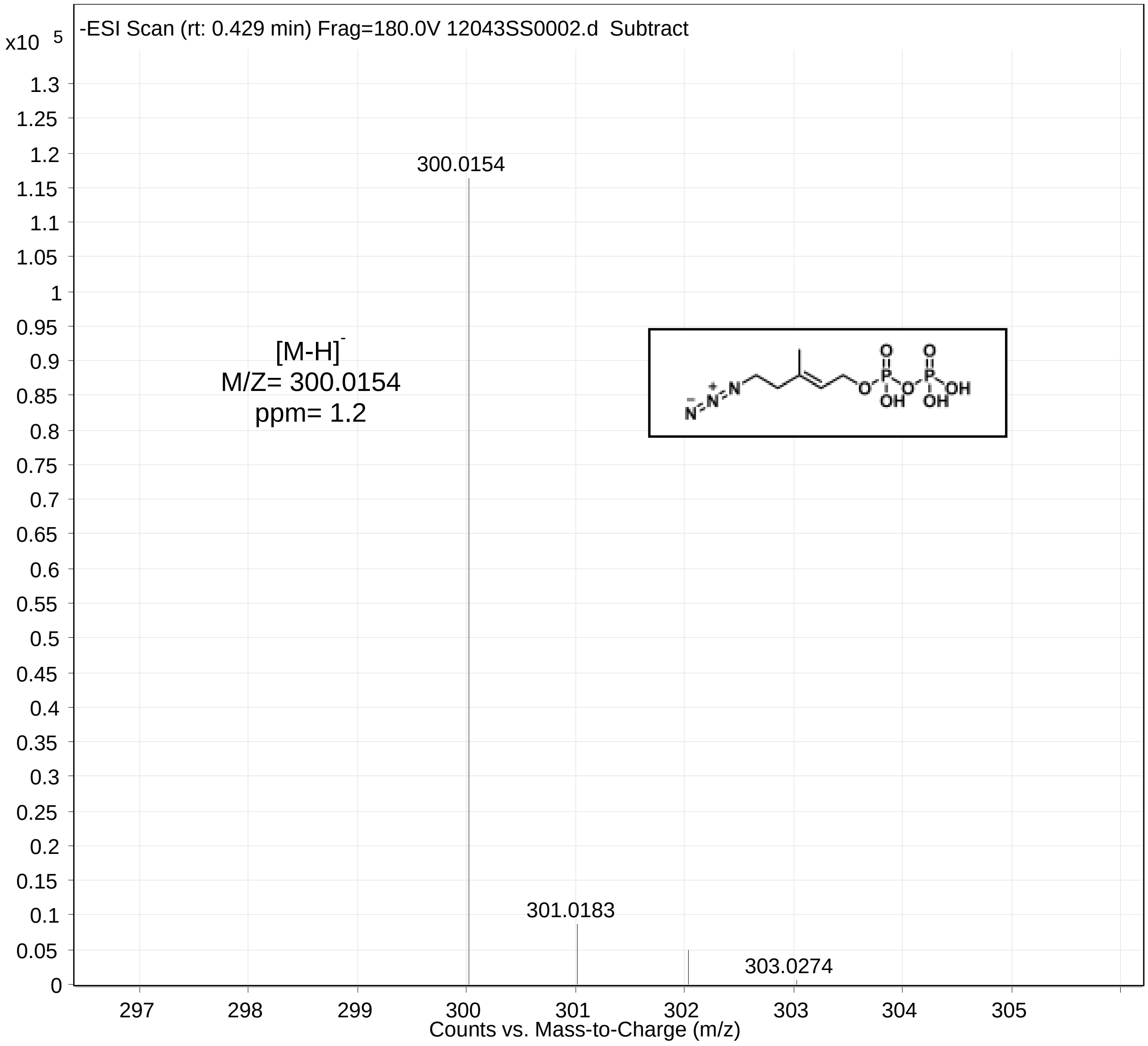

(-)-ESI-HRMS Spectrum of **34**

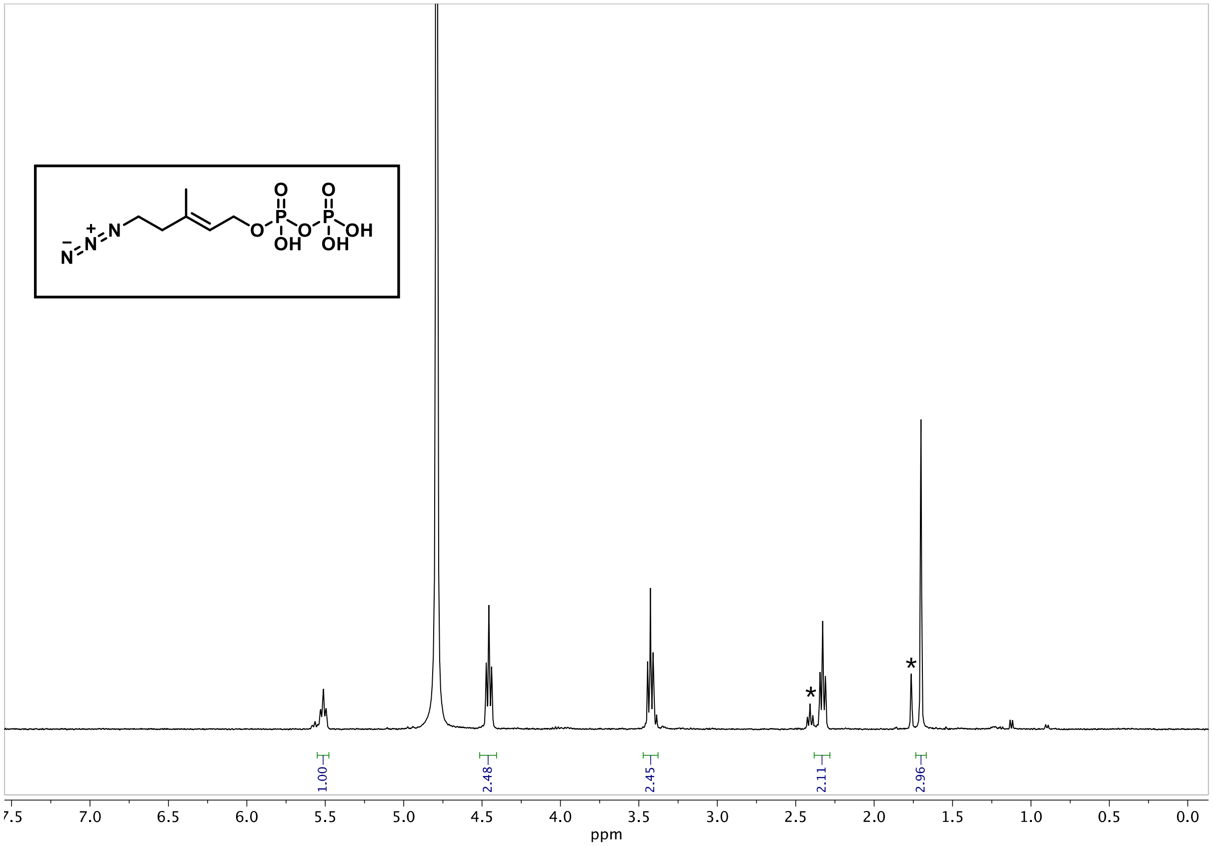

^{1}H NMR Spectrum of **34** (400 MHz, D_2O) *Denotes an impurity.

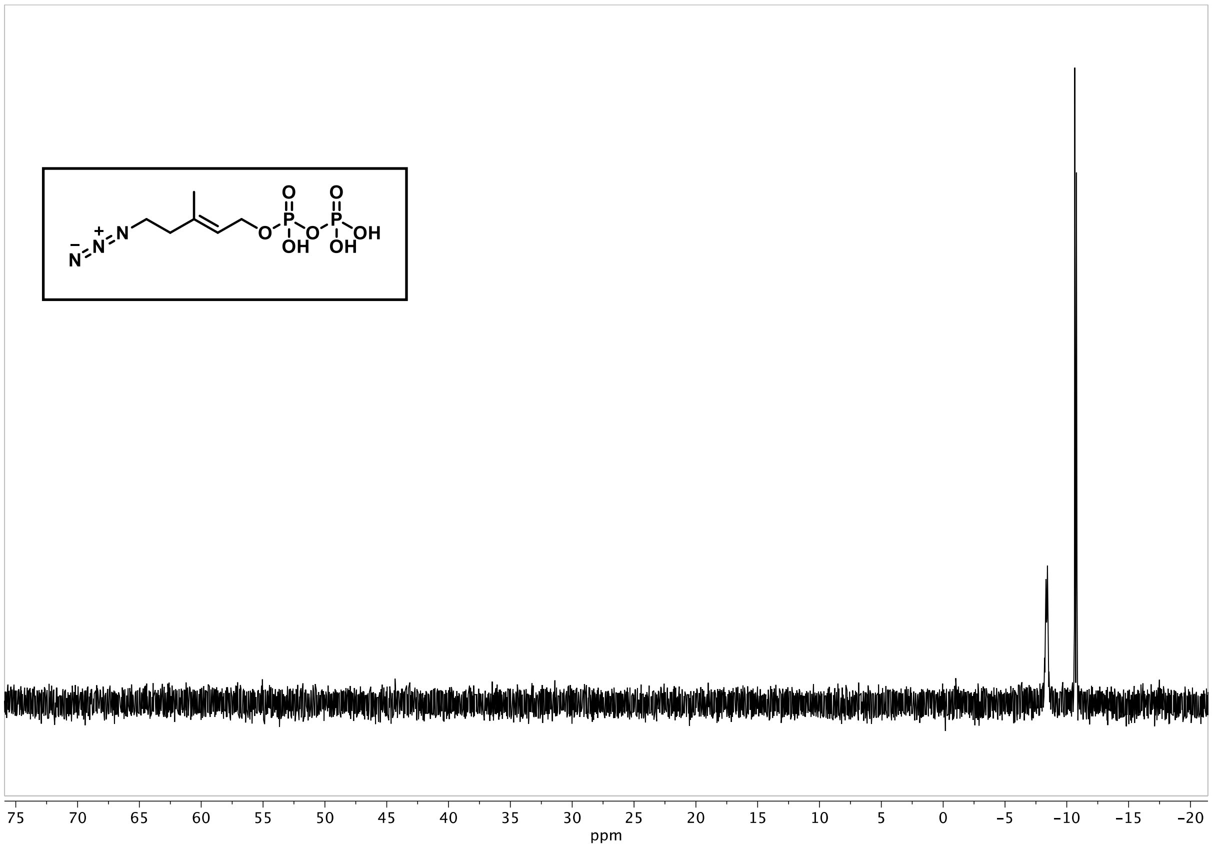

^{31}P NMR Spectrum of **34** (162 MHz, D_2O)

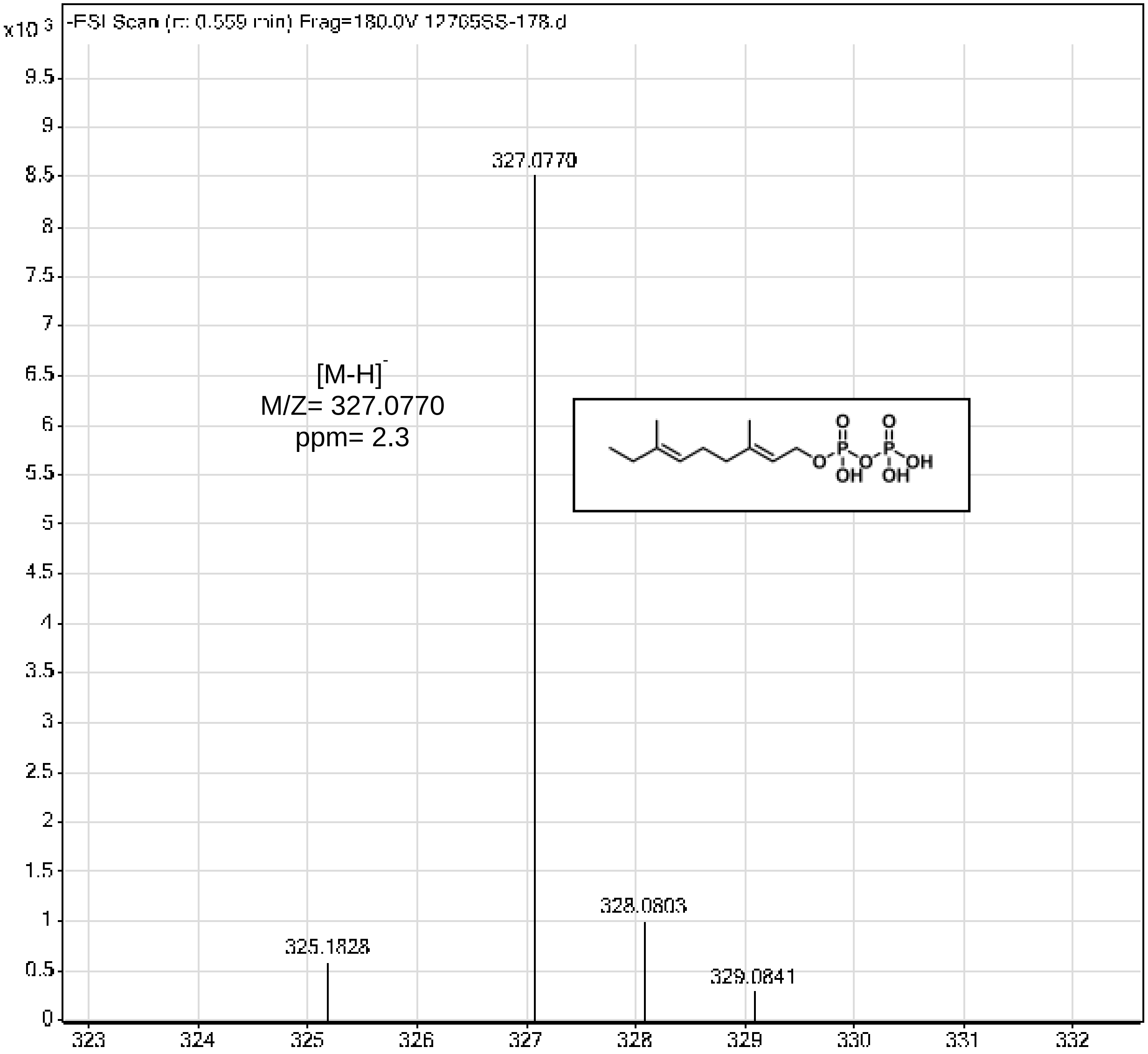

(-)-ESI-HRMS Spectrum of **35**

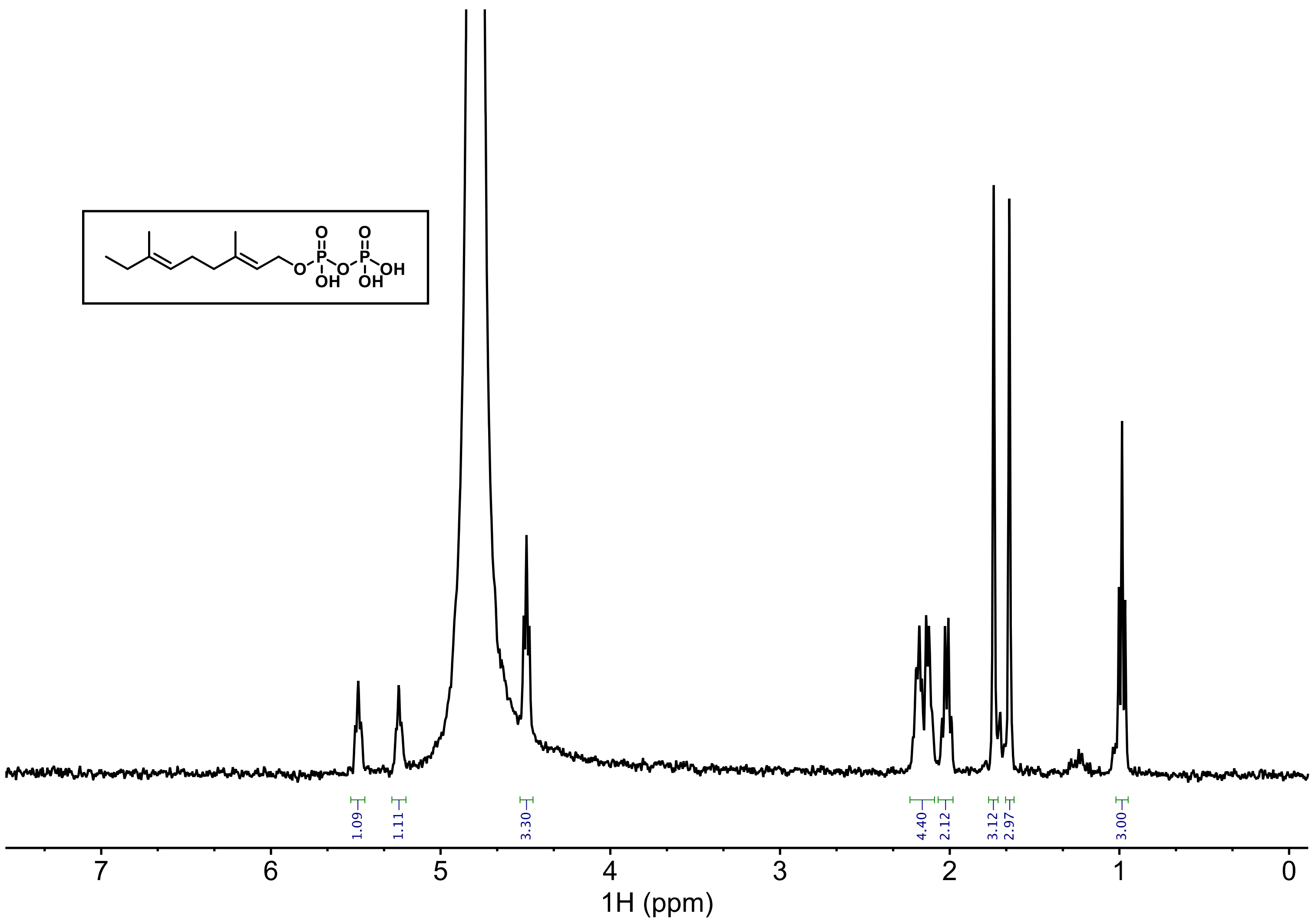

^{1}H NMR Spectrum of **35** (400 MHz, D_2O)

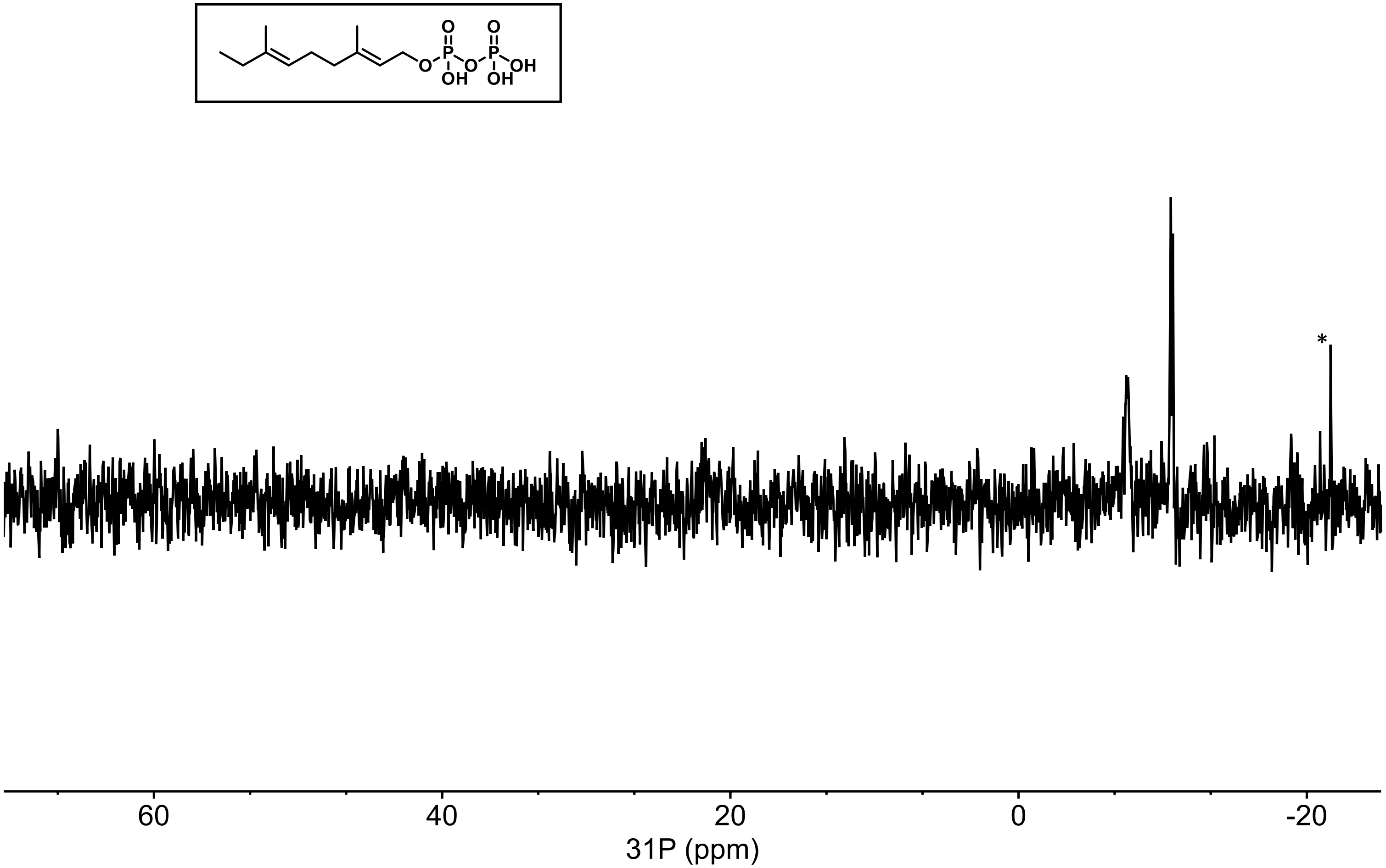

^{31}P NMR Spectrum of **35** (162 MHz, D_2O) *Denotes an impurity.

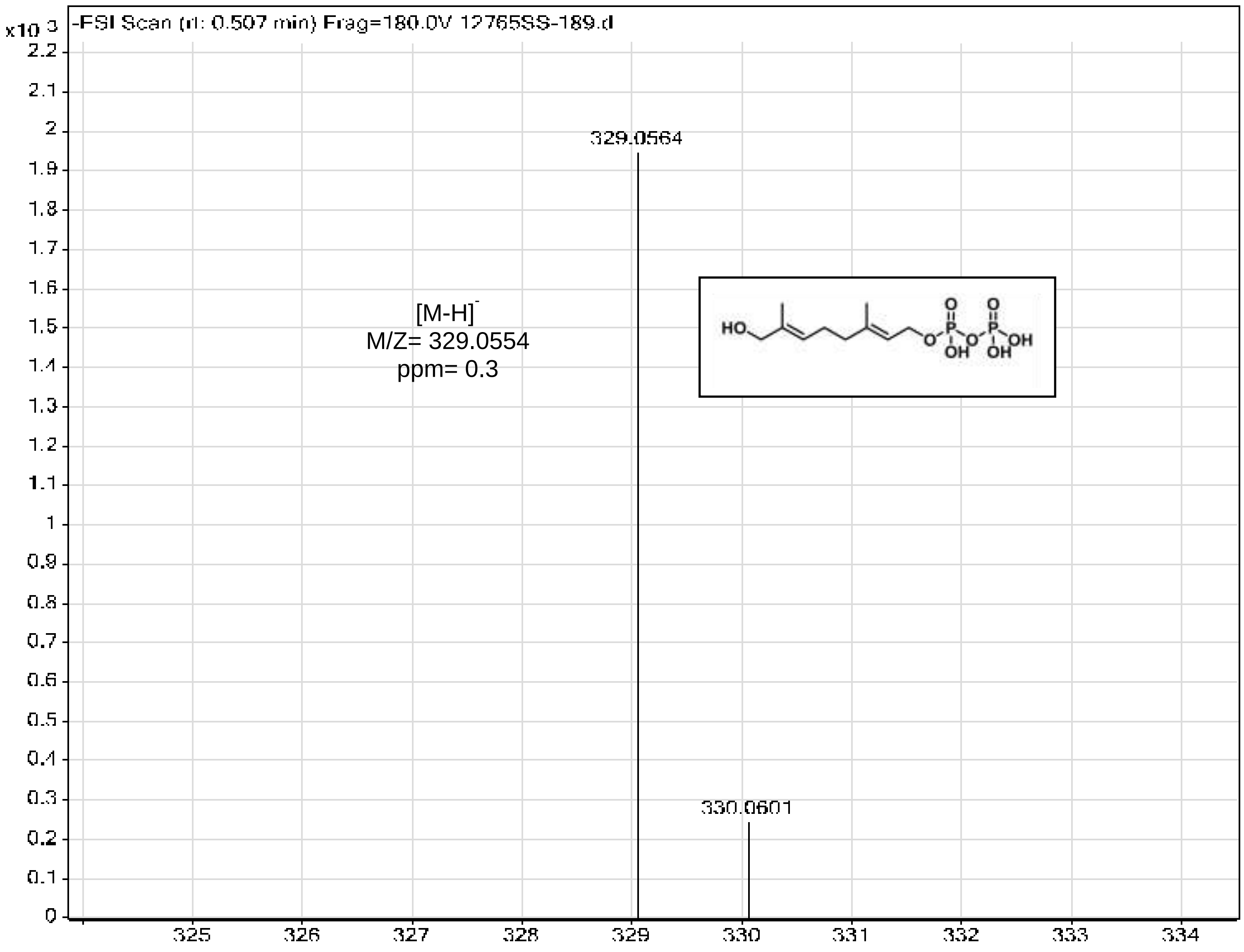

(-)-ESI-HRMS Spectrum of **36**

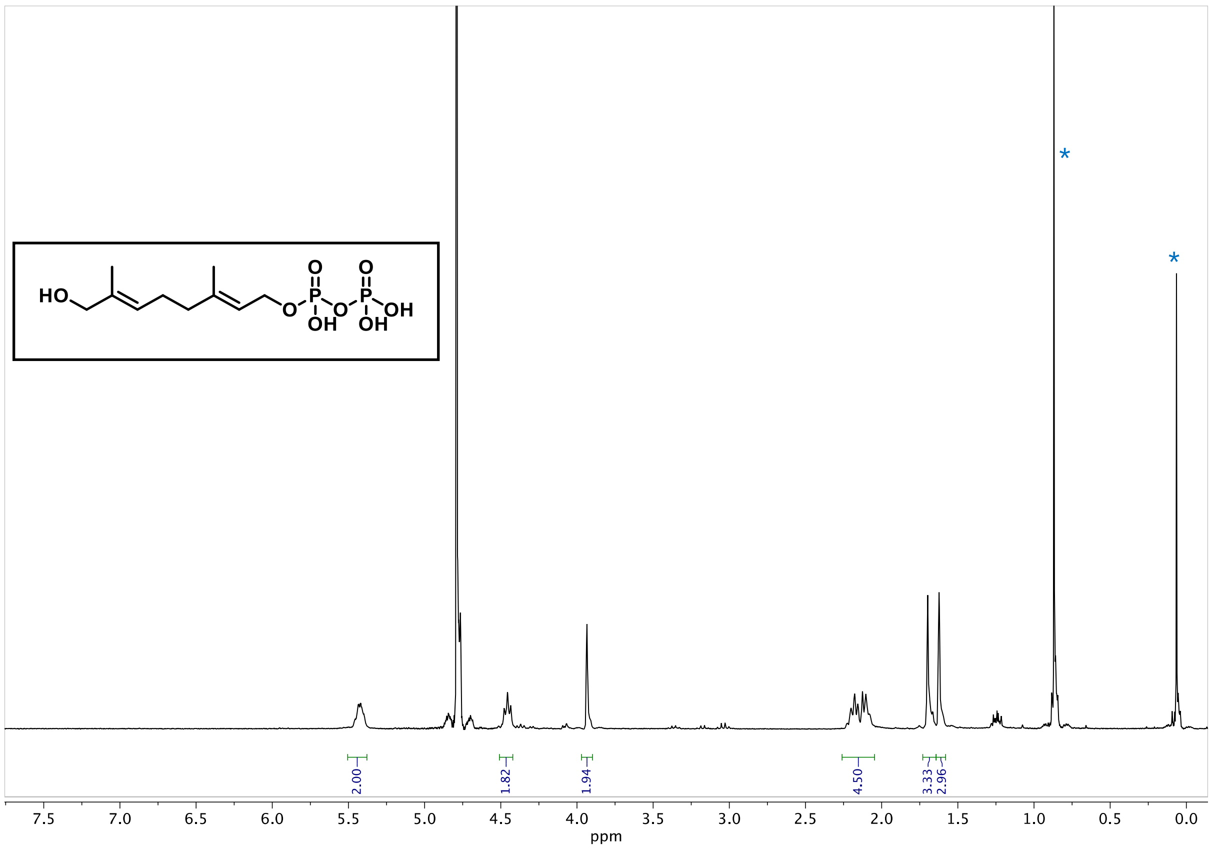

^{1}H NMR Spectrum of **36** (300 MHz, D_2O) *Denotes TBS protecting group impurity.

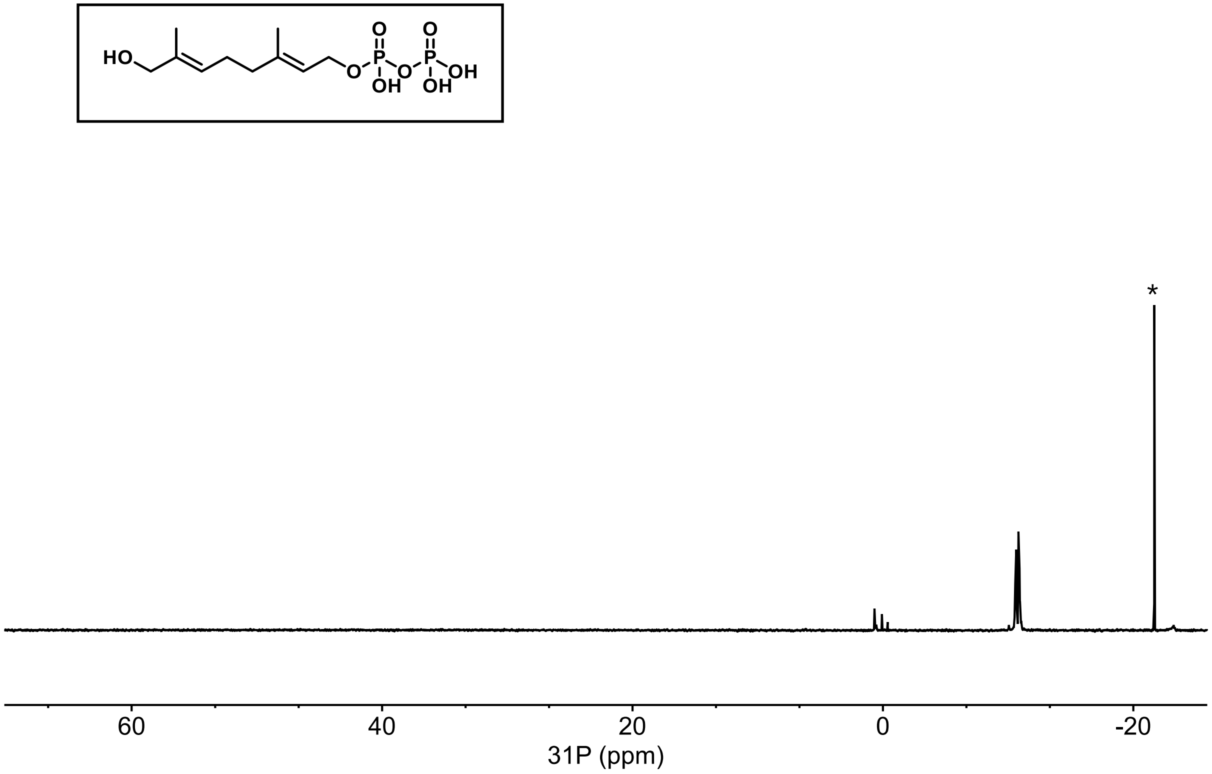

^{31}P NMR Spectrum of **36** (243 MHz, D_2O) *Denotes an impurity.

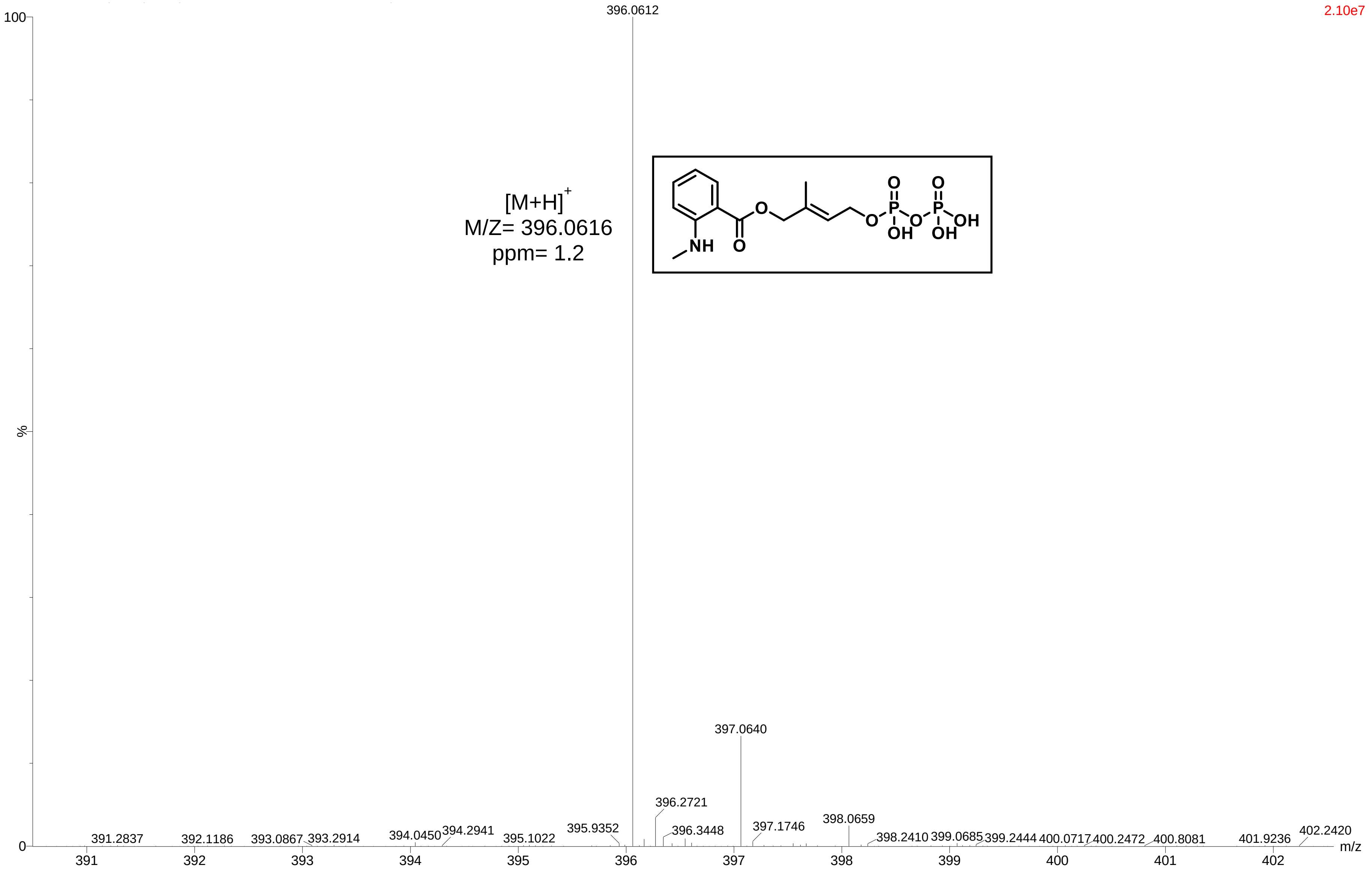

(+)-ESI-HRMS Spectrum of **38**

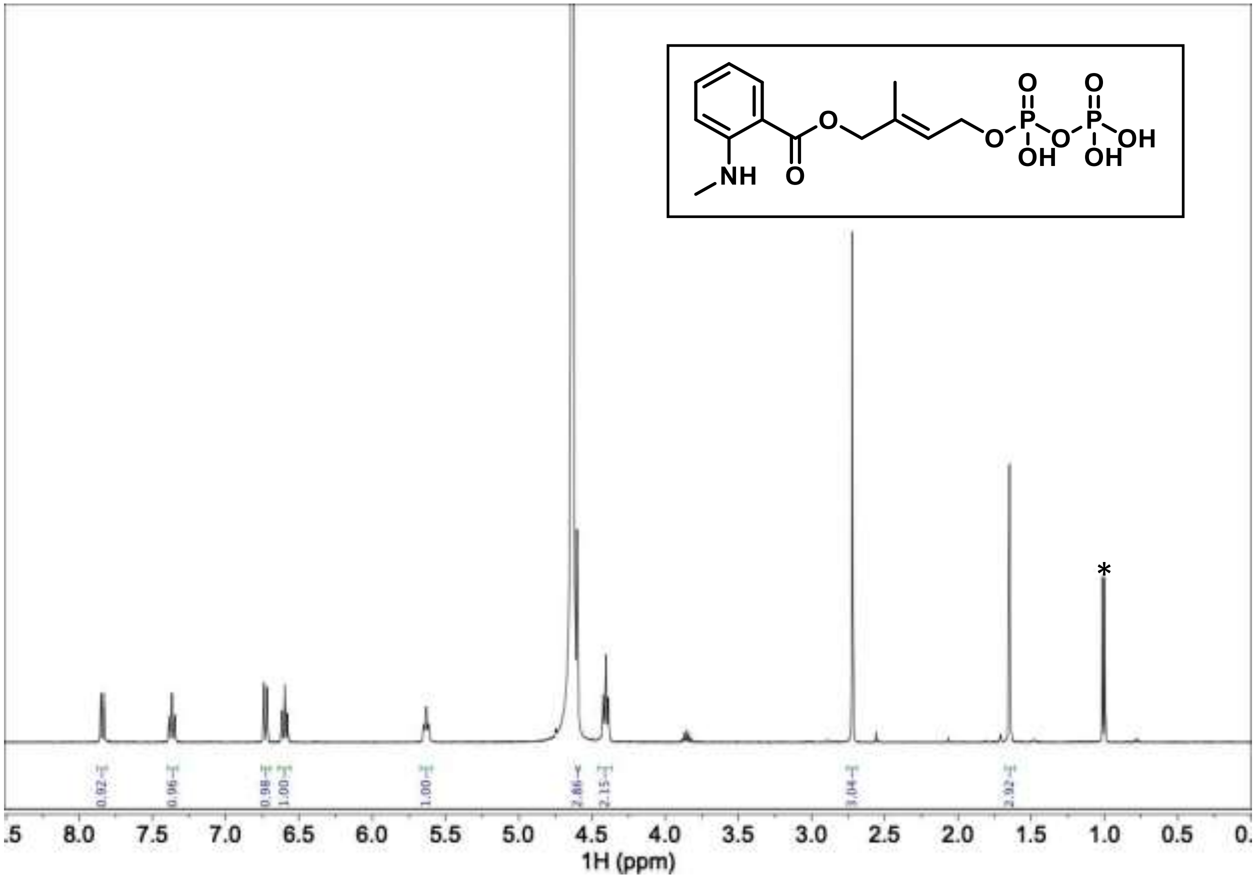

^{1}H NMR Spectrum of **38** (400 MHz, D_2O) *Denotes an impurity.

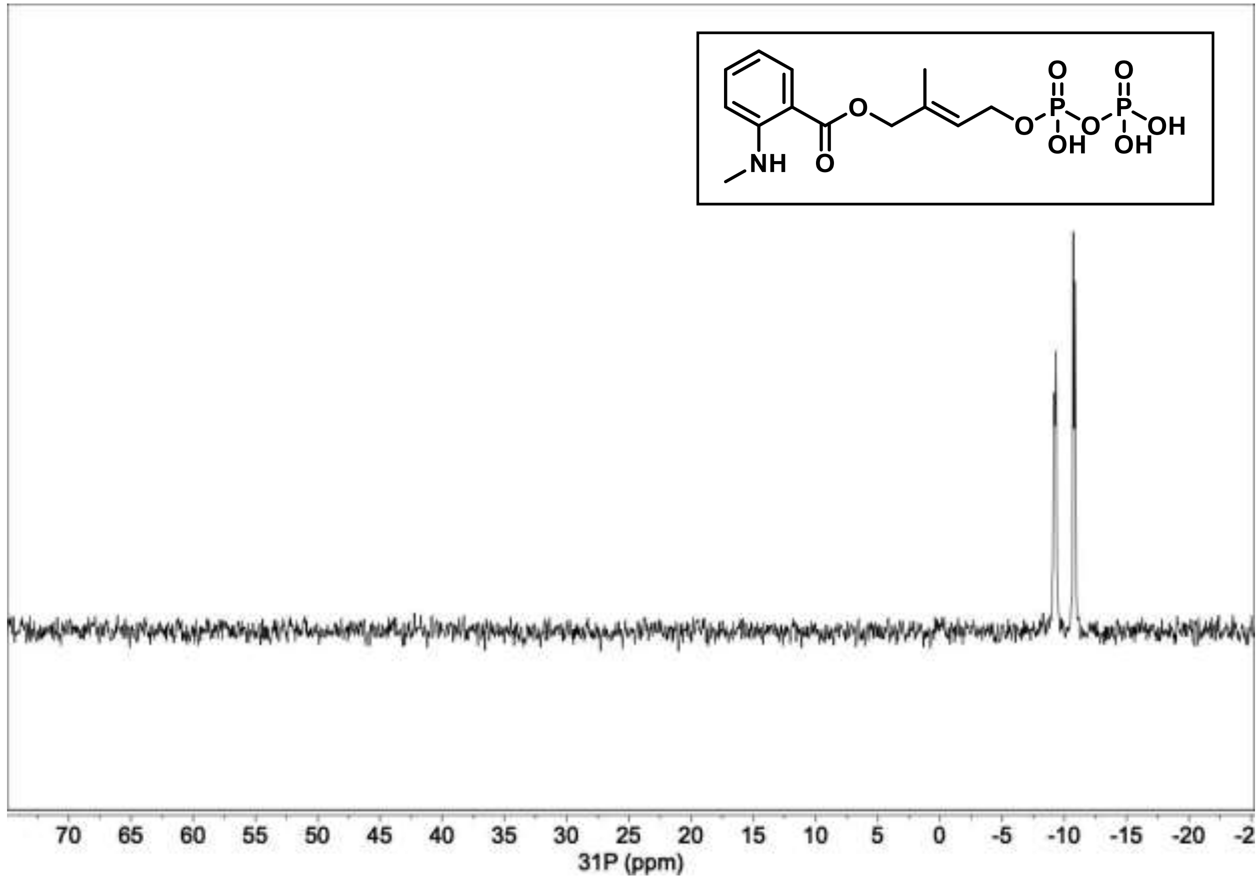

^{31}P NMR Spectrum of **38** (162 MHz, D_2O)

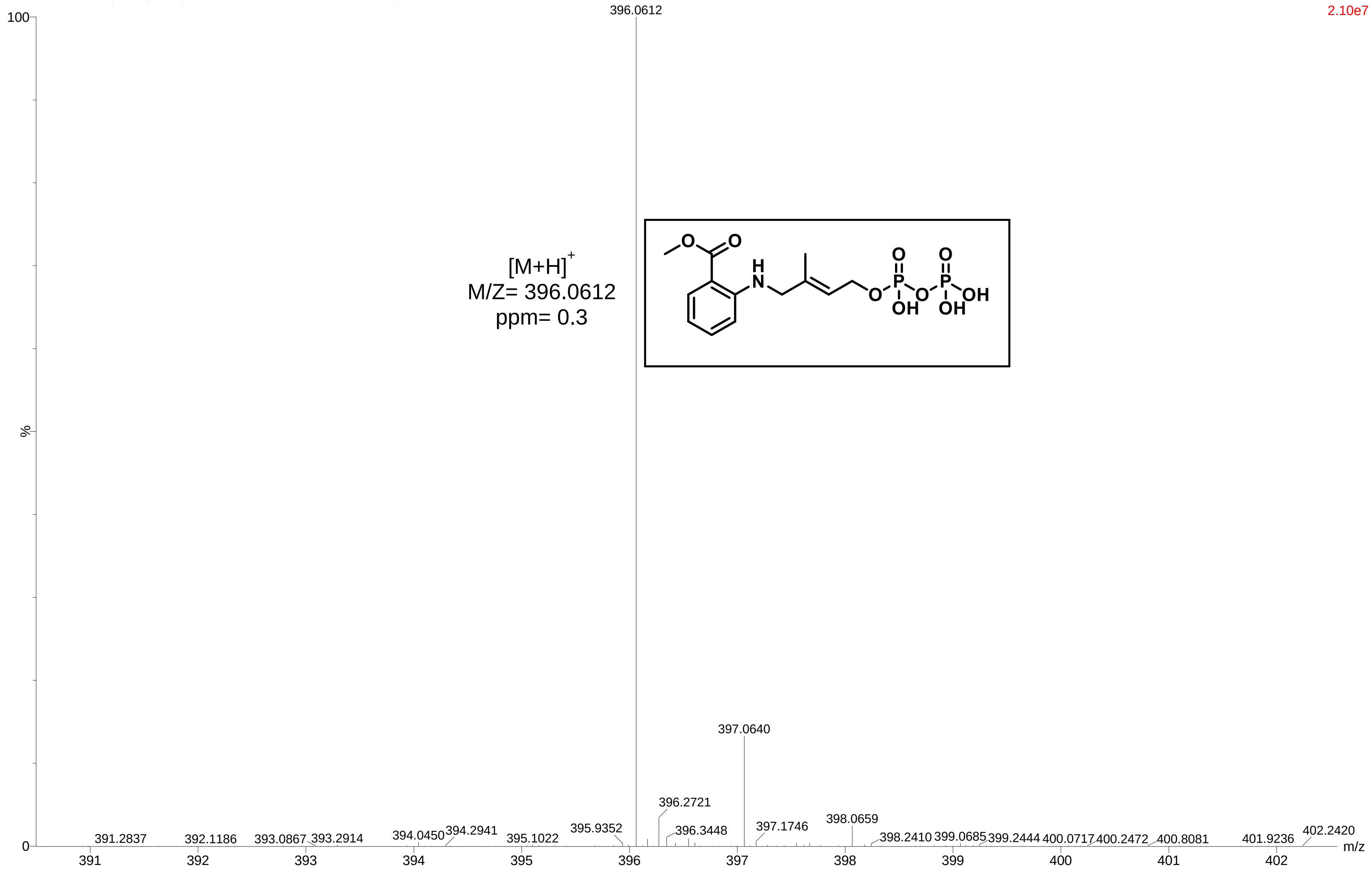

(+)-ESI-HRMS Spectrum of **39**

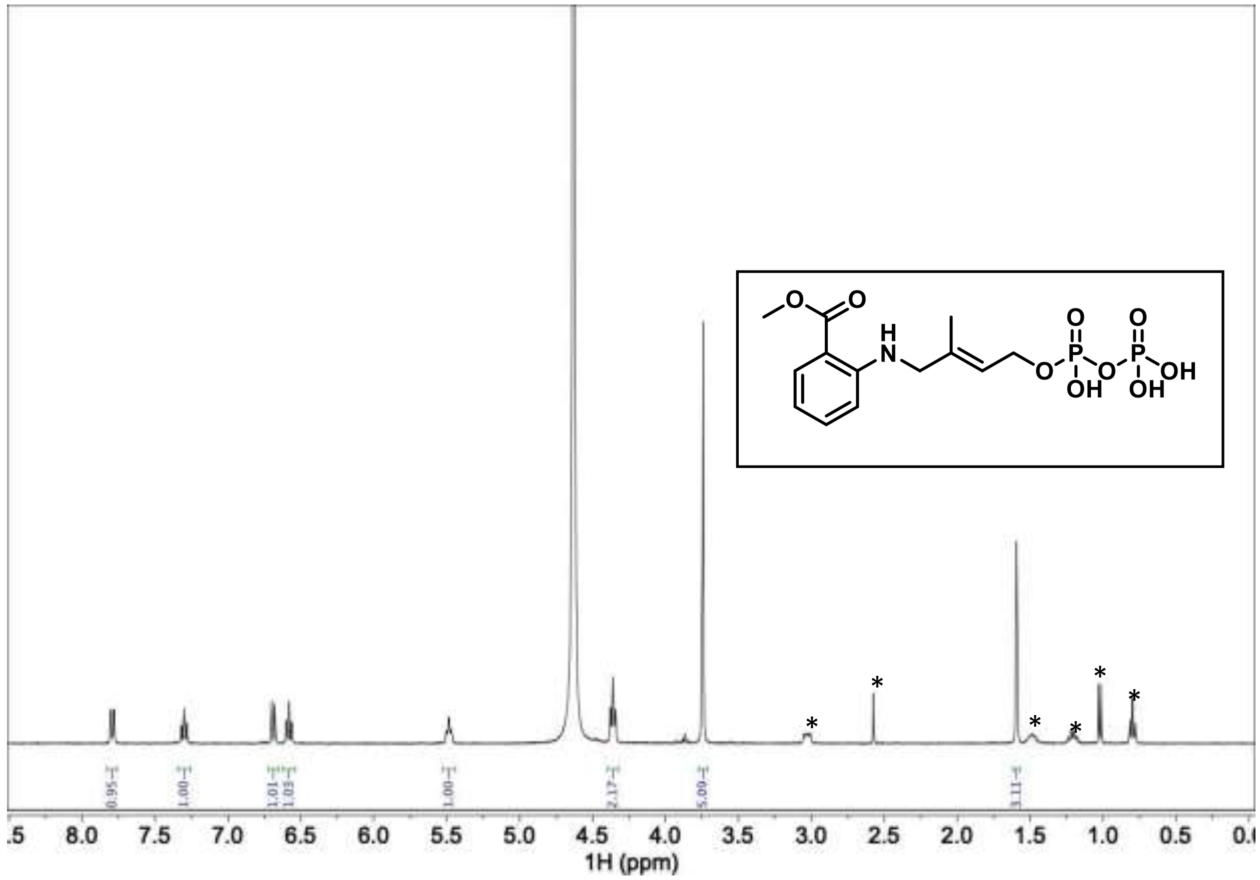

^{1}H NMR Spectrum of **39** (400 MHz, D_2O) *Denotes an impurity.

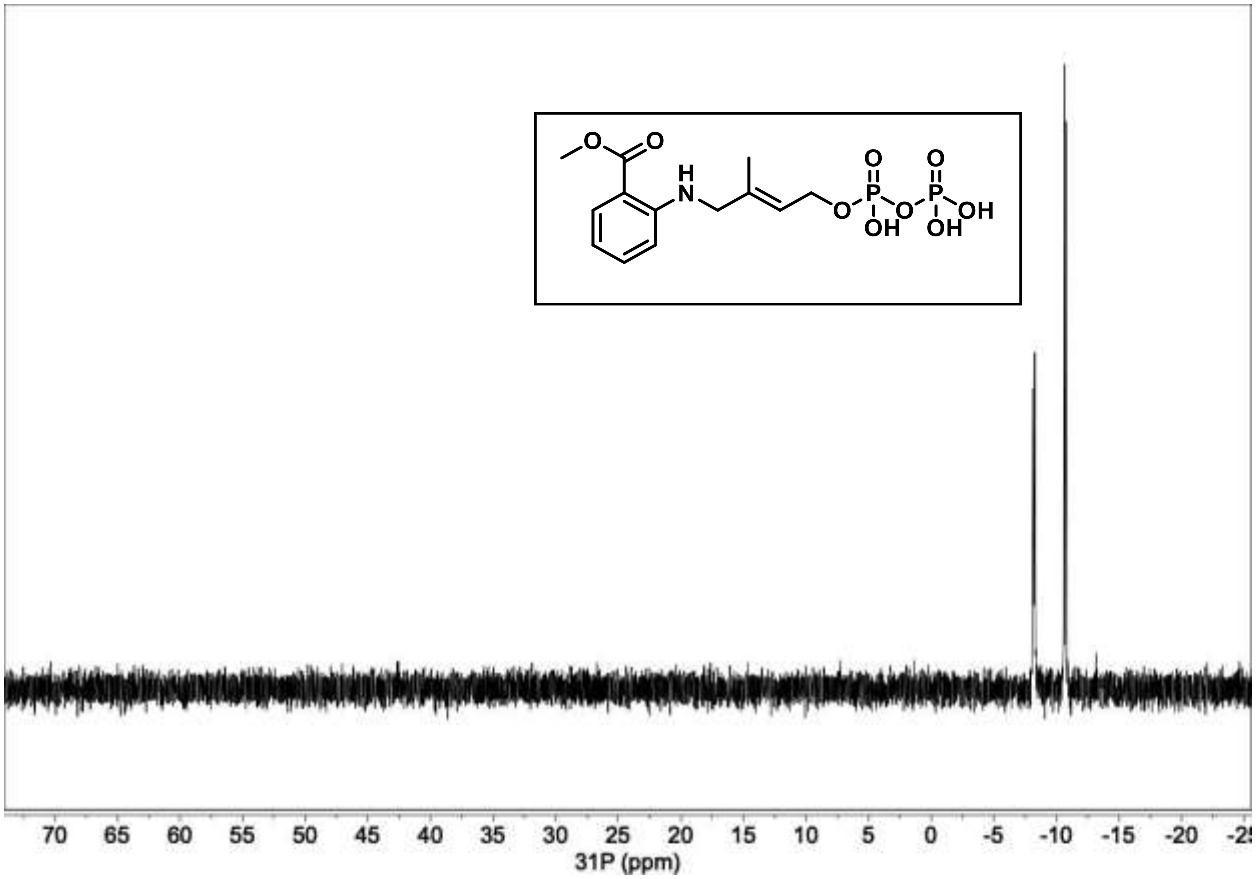

^{31}P NMR Spectrum of **39** (162 MHz, D_2O)

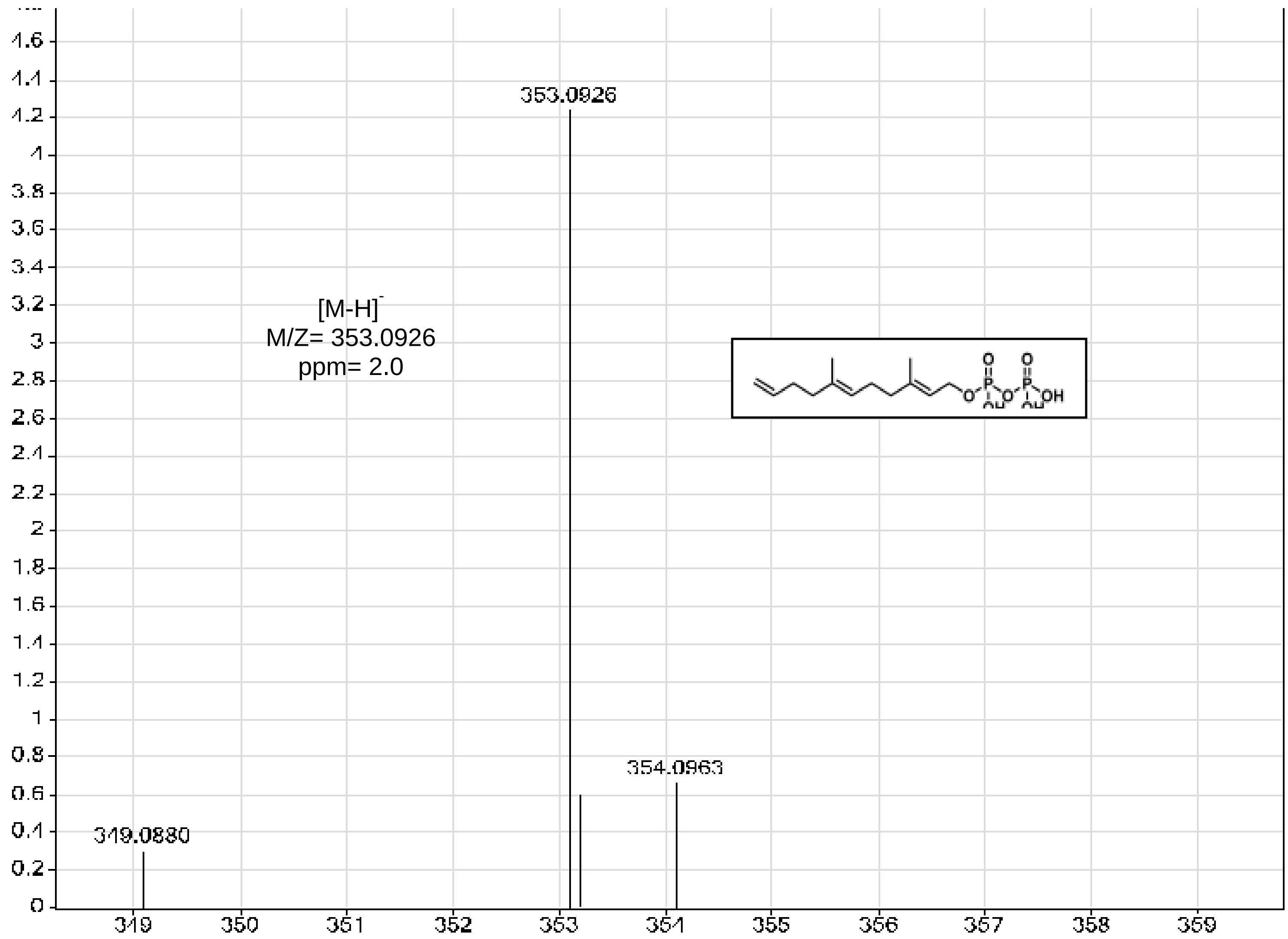

(-)-ESI-HRMS Spectrum of **40**

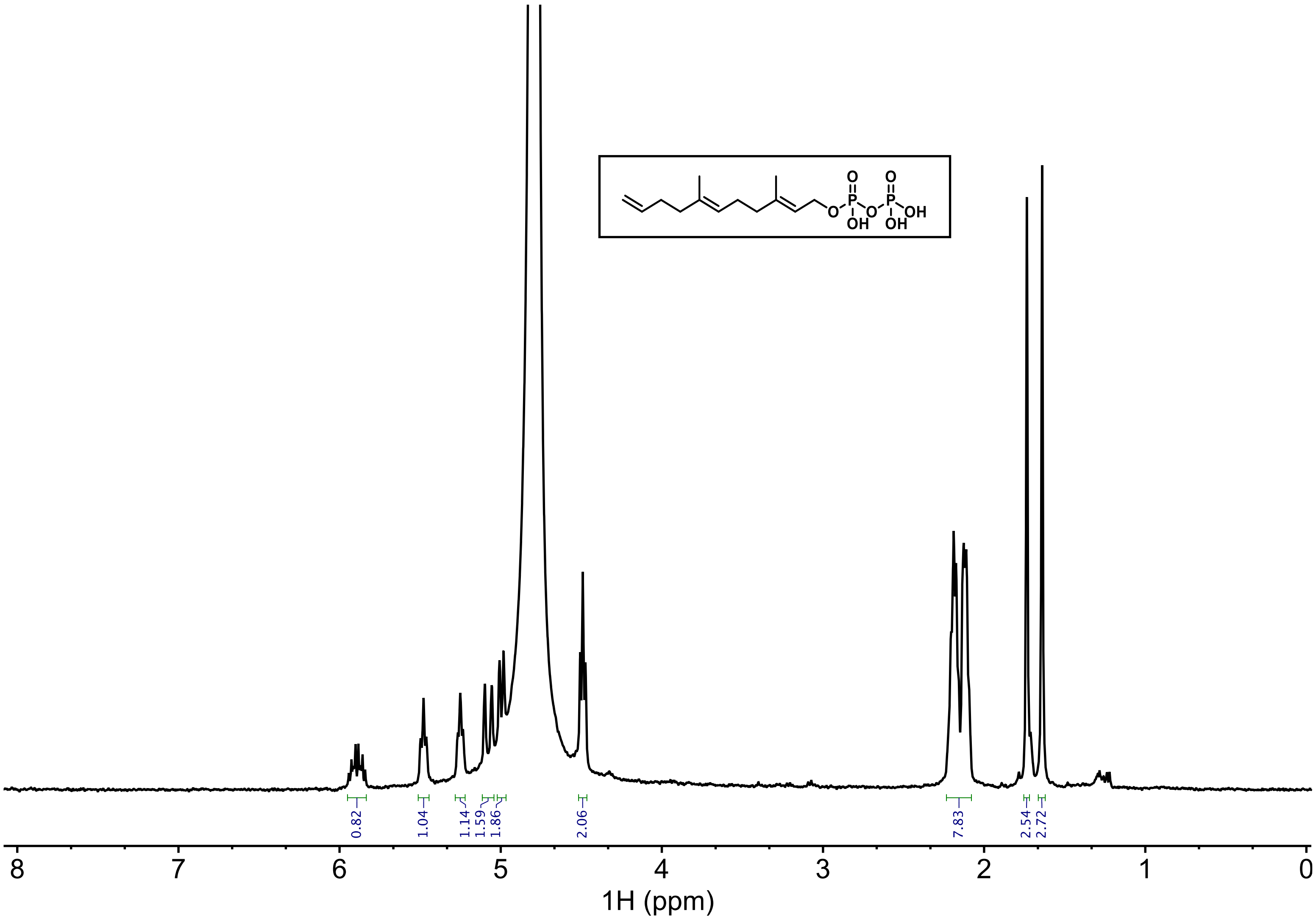

^{1}H NMR Spectrum of **40** (400 MHz, D_2O)

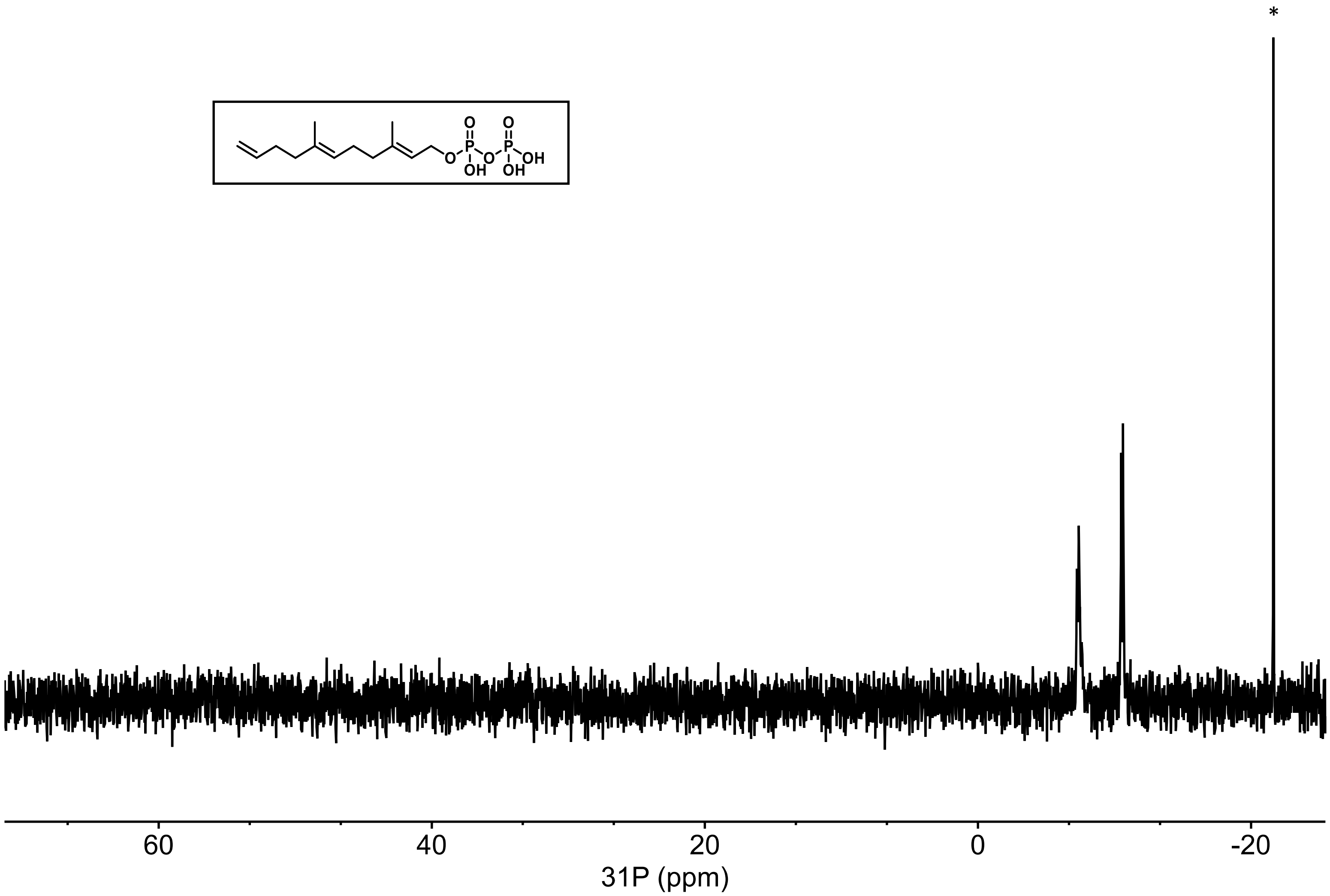

^{31}P NMR Spectrum of **40** (162 MHz, D_2O) *Denotes an impurity.

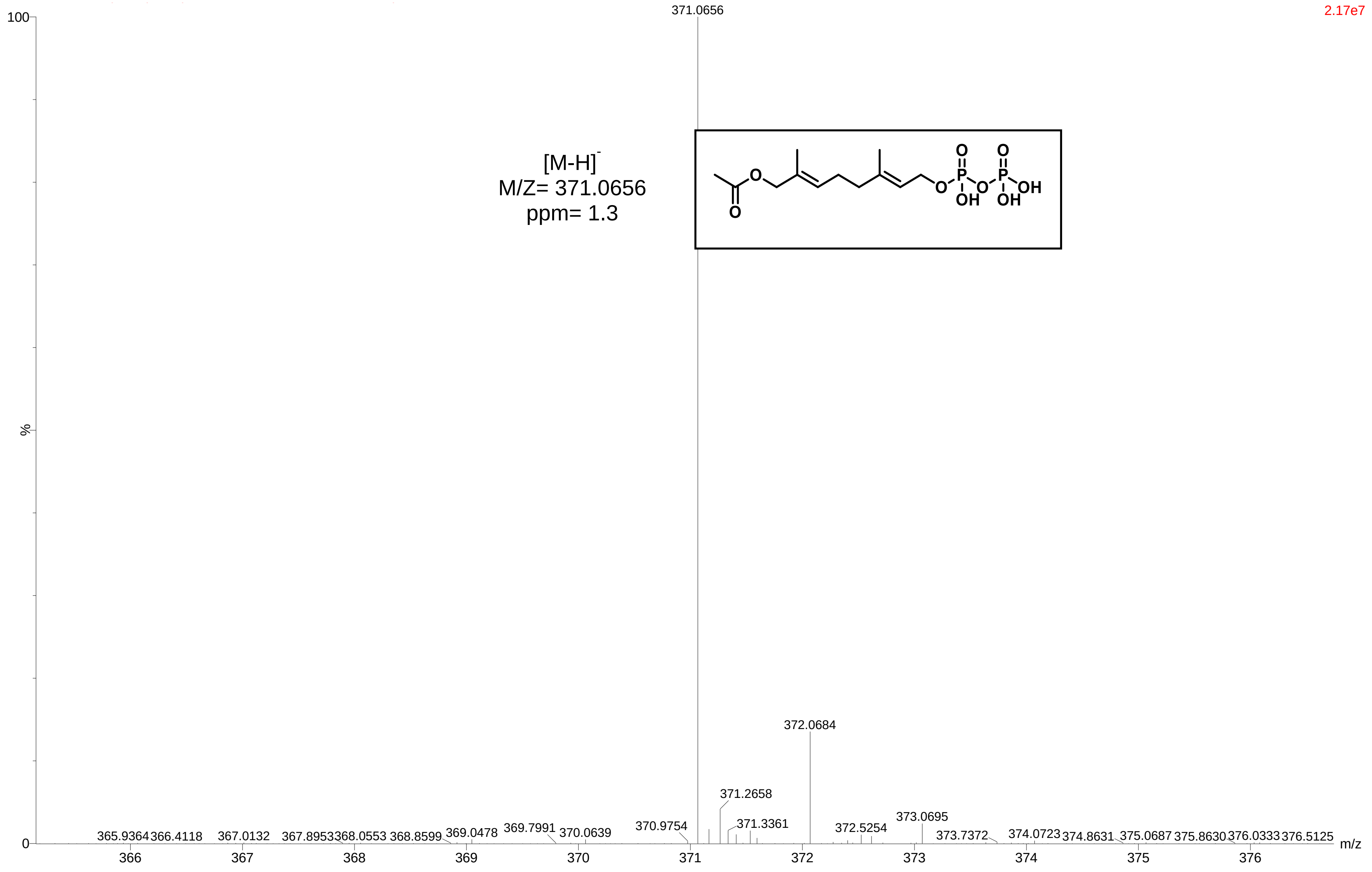

(-)-ESI-HRMS Spectrum of **41**

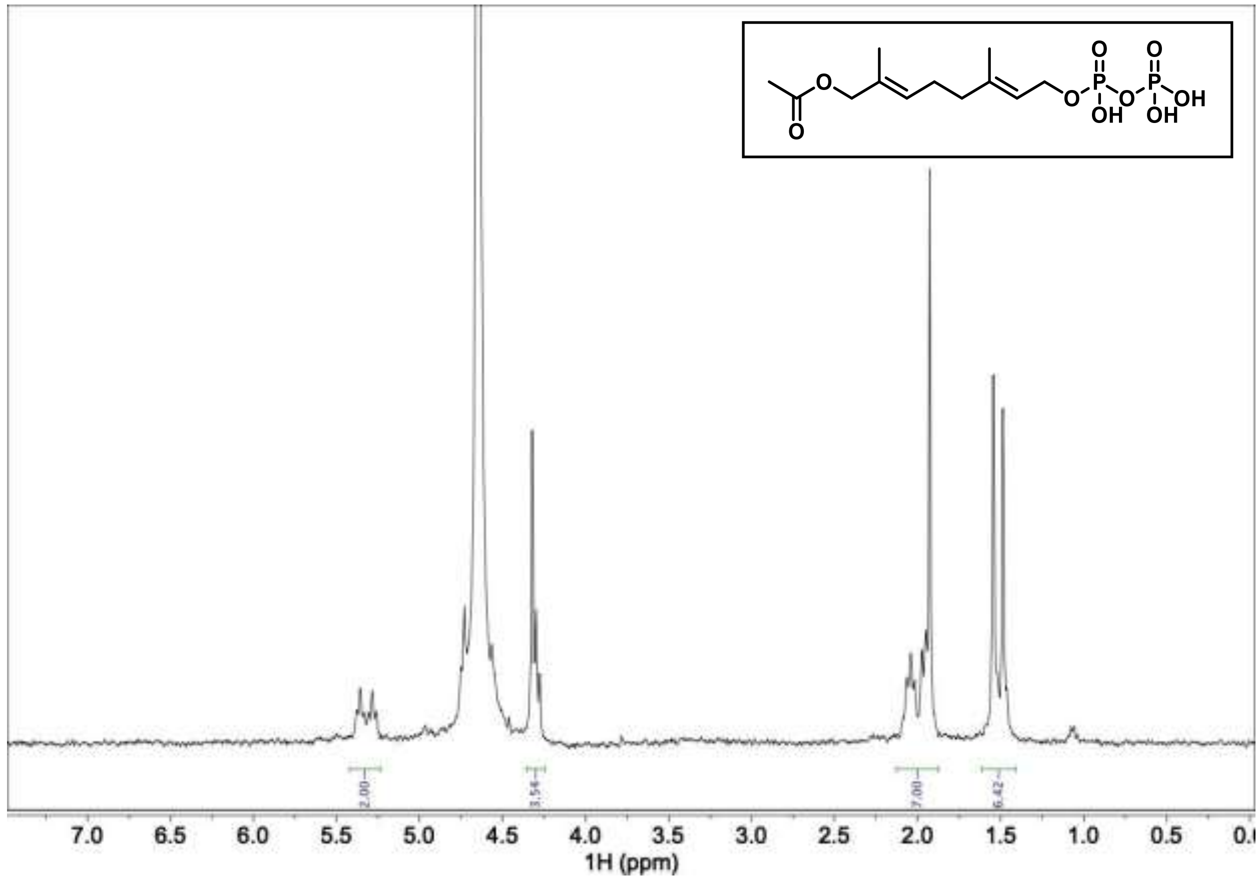

^{1}H NMR Spectrum of **41** (300 MHz, D_2O)

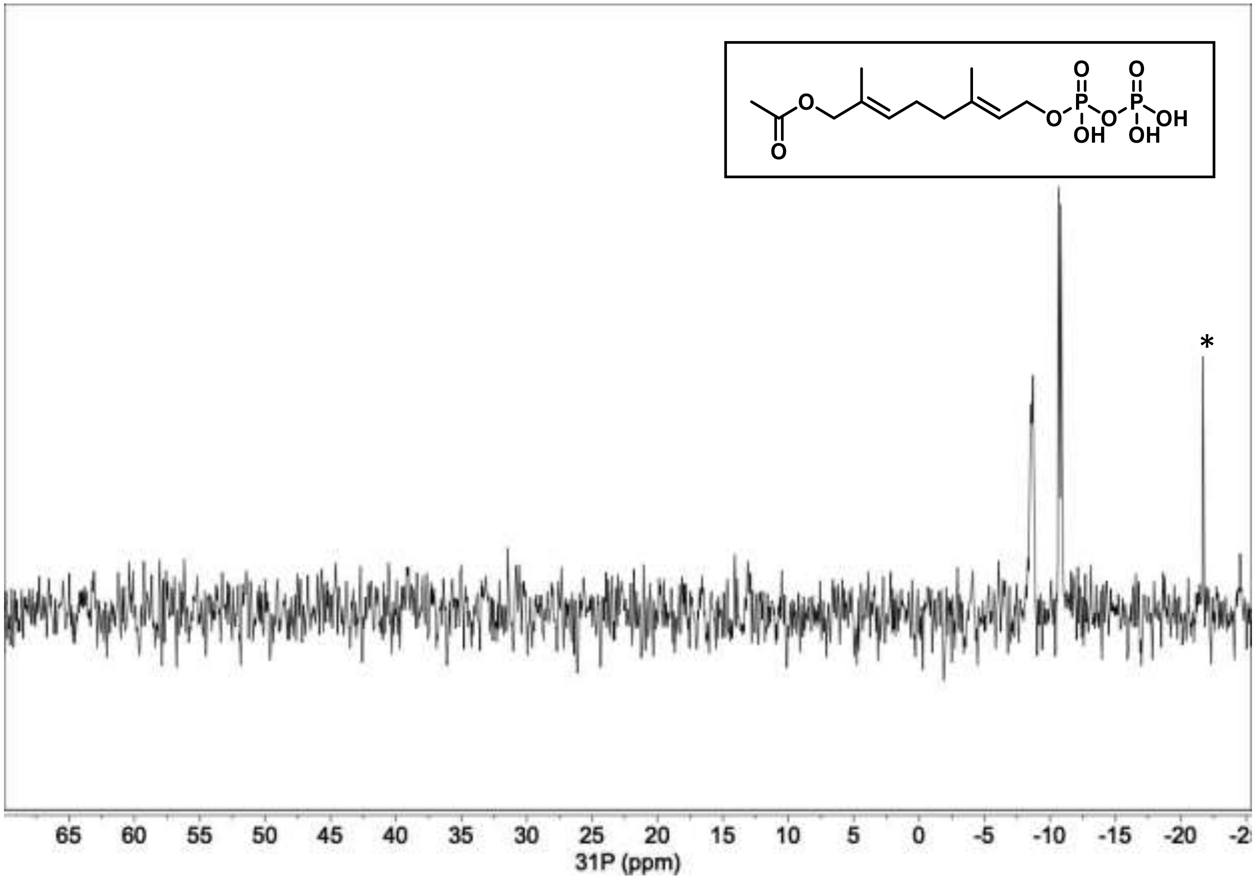

^{31}P NMR Spectrum of **41** (122 MHz, D_2O) *Denotes an impurity.

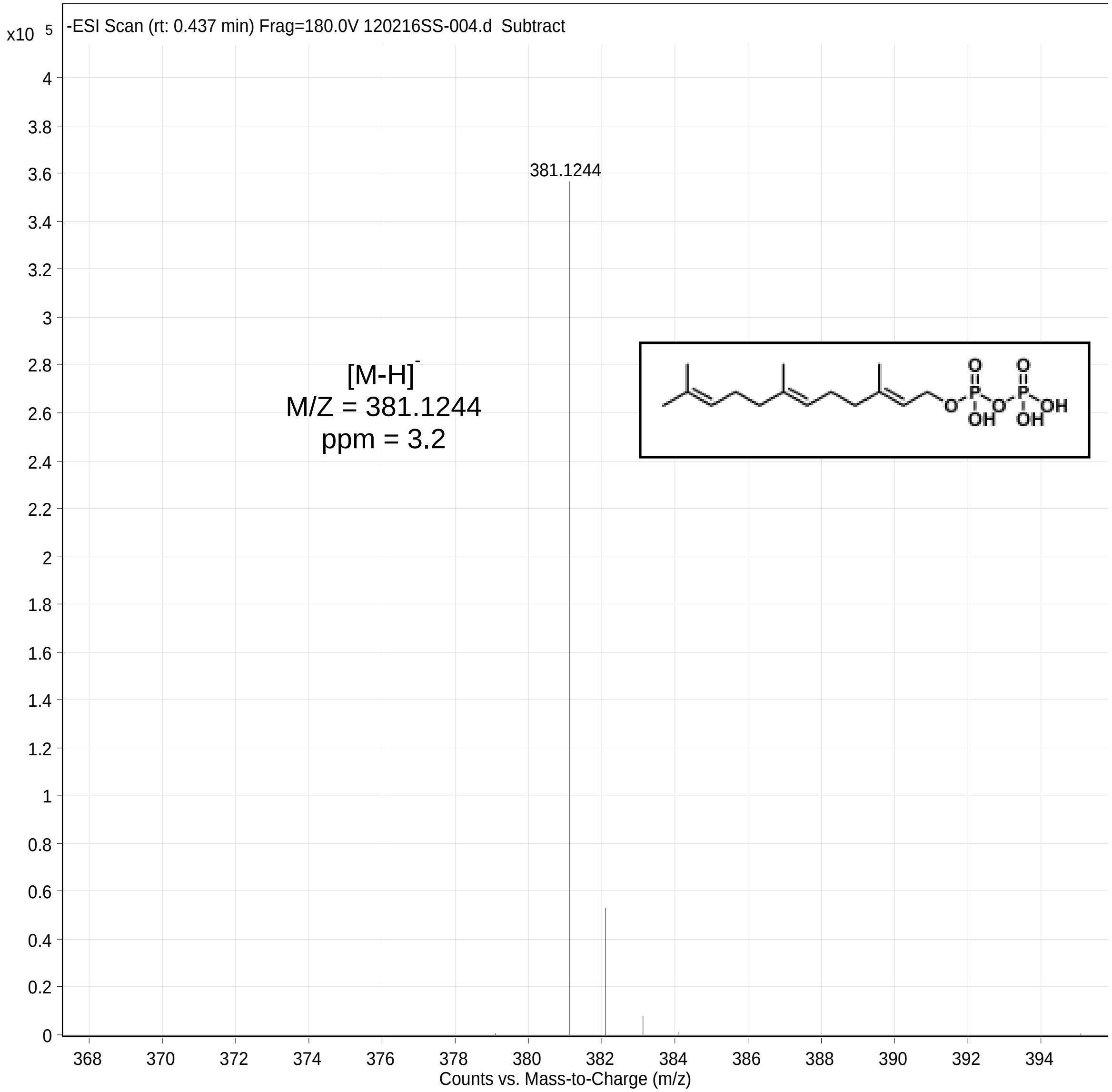

(-)-ESI-HRMS Spectrum of **42**

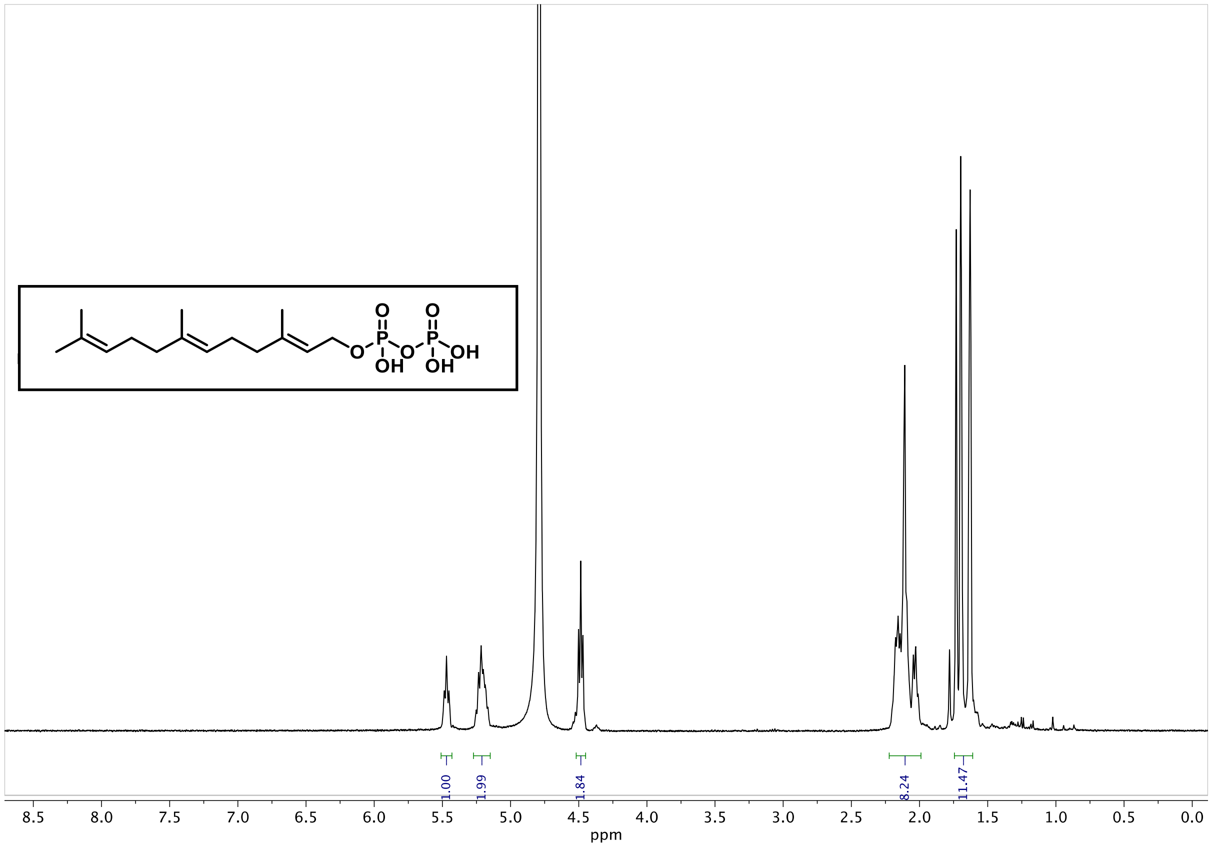

^{1}H NMR Spectrum of **42** (400 MHz, D_2O)

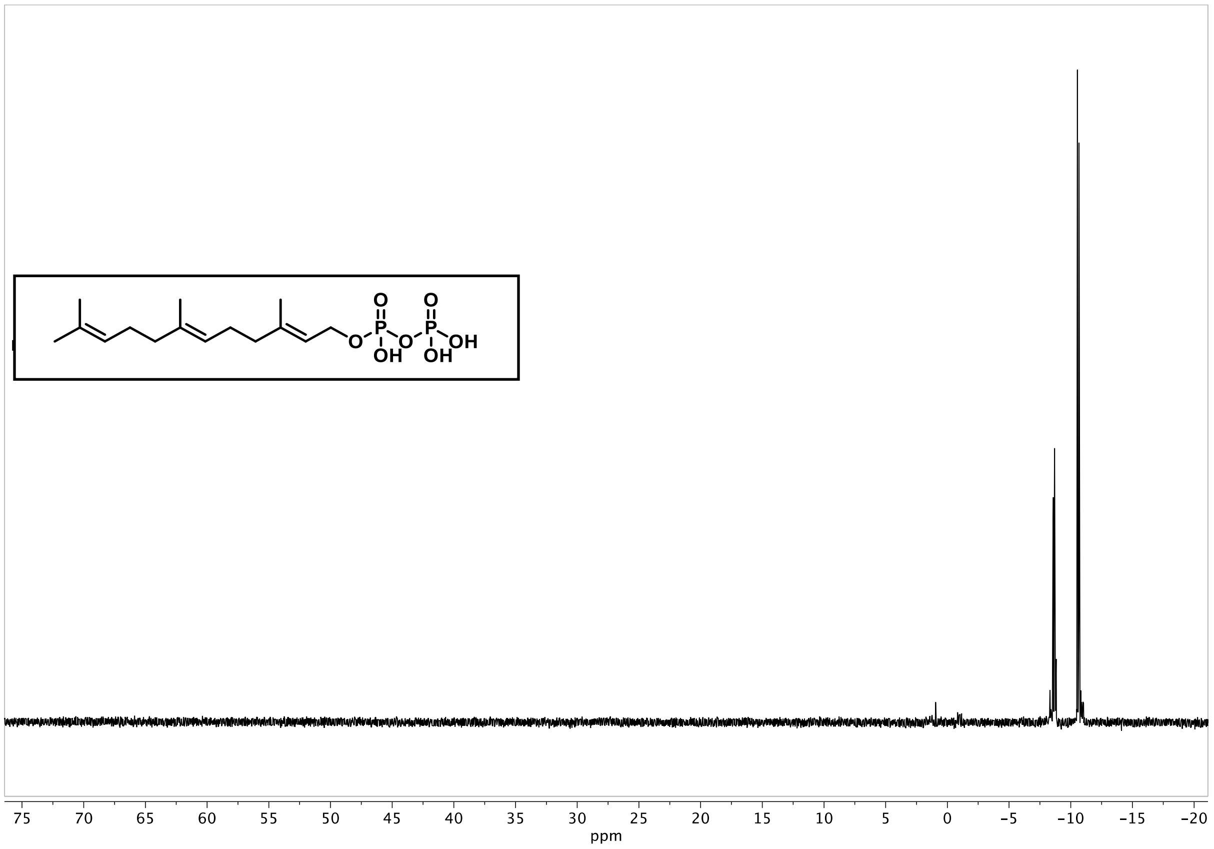

^{31}P NMR Spectrum of **42** (162 MHz, D_2O)

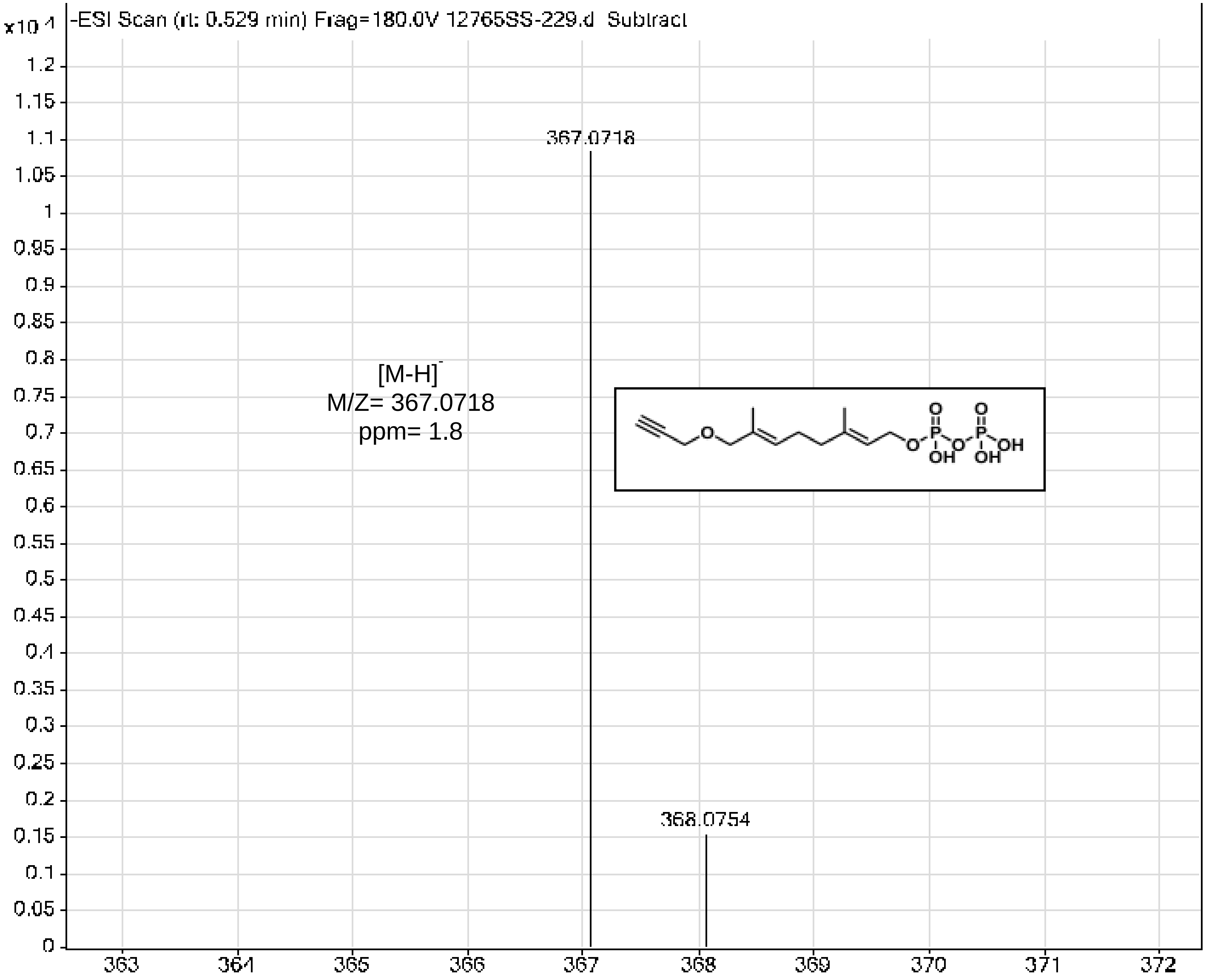

(-)-ESI-HRMS Spectrum of **43**

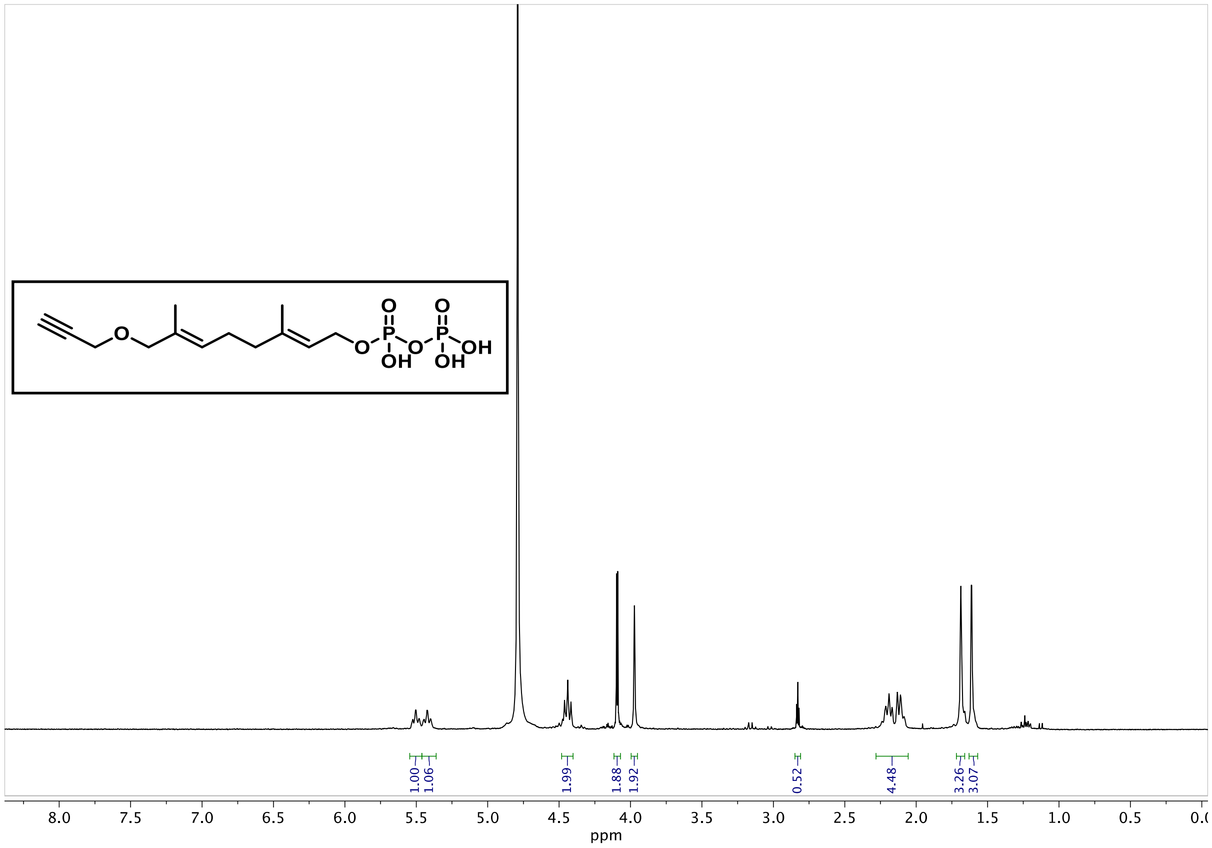

^{1}H NMR Spectrum of **43** (300 MHz, D_2O)

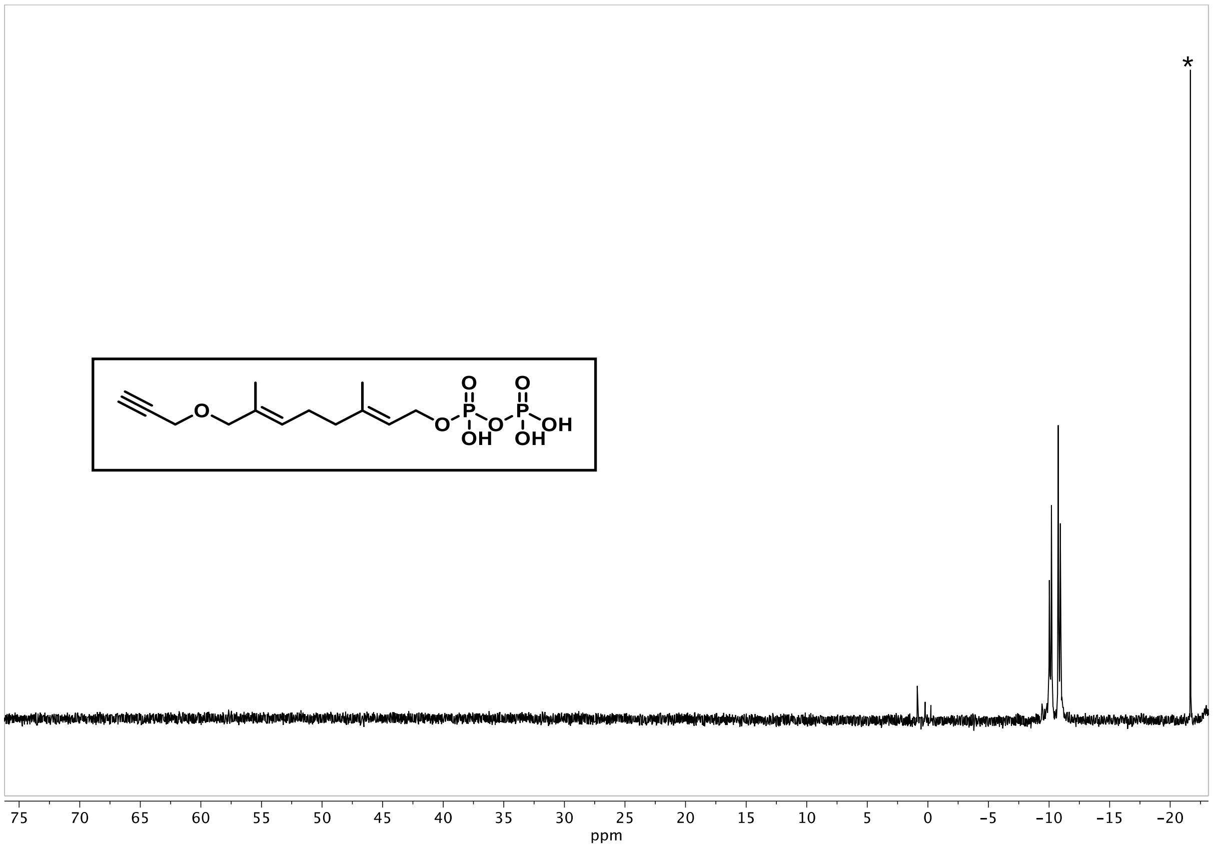

^{31}P NMR Spectrum of **43** (122 MHz, D_2O) *Denotes an impurity.

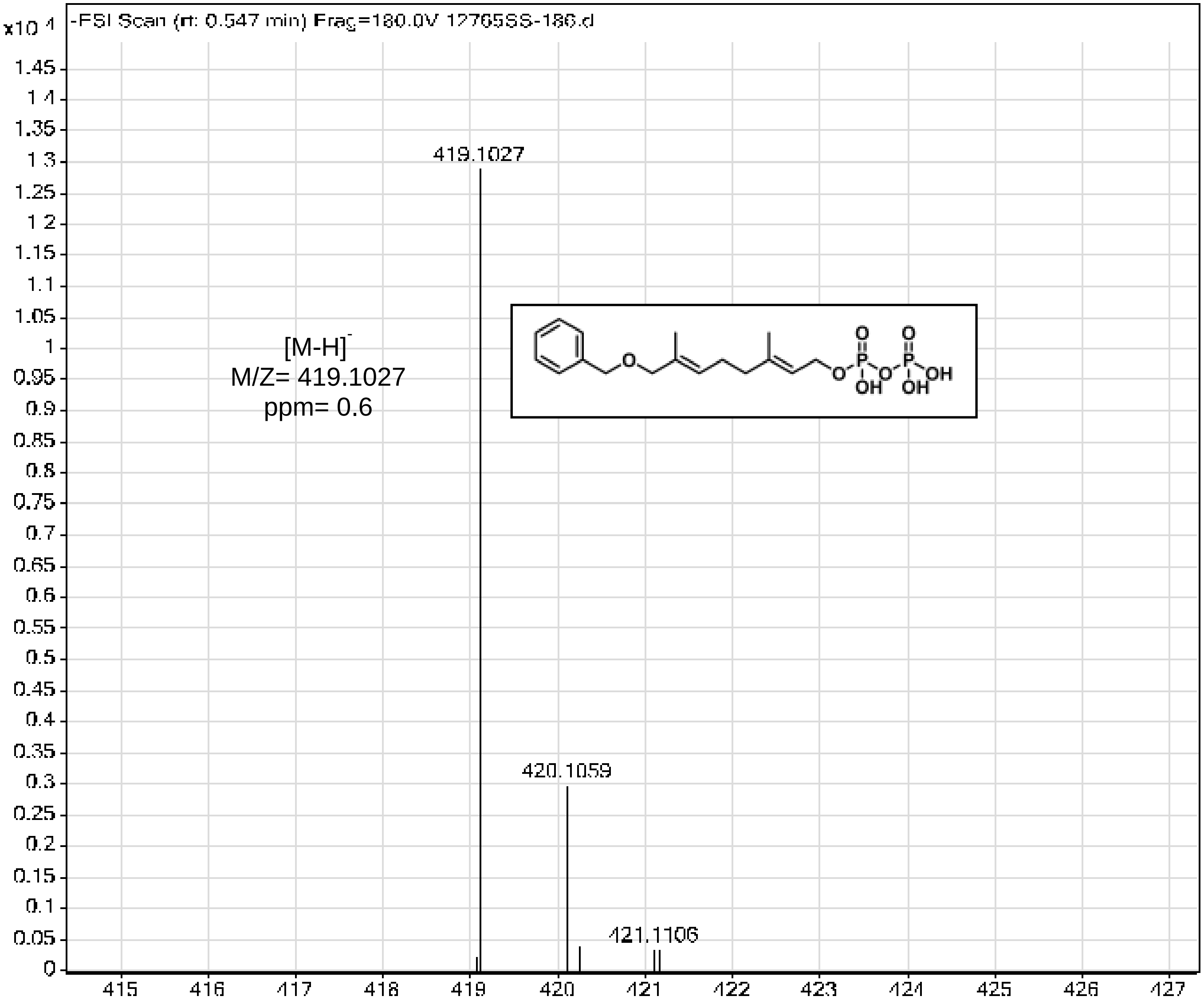

(-)-ESI-HRMS Spectrum of **44**

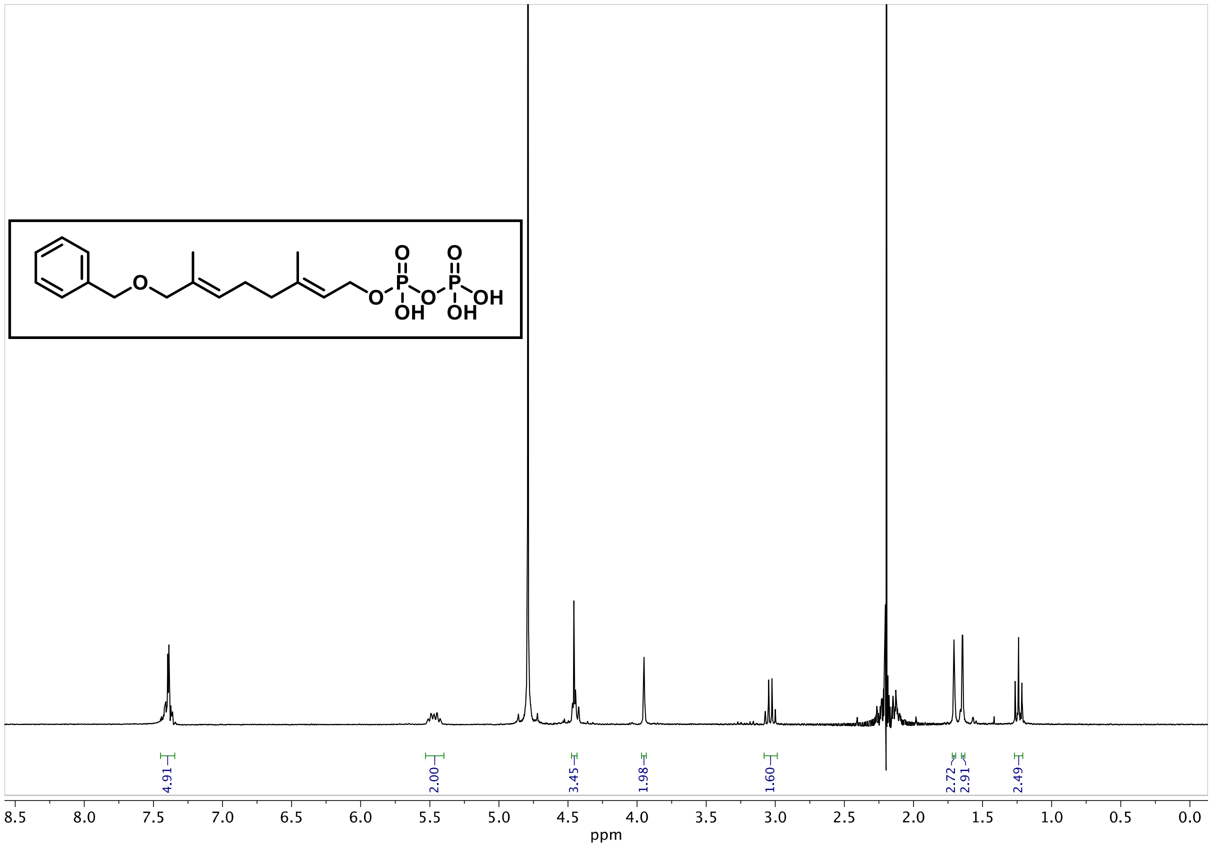

^{1}H NMR Spectrum of **44** (300 MHz, D_2O)

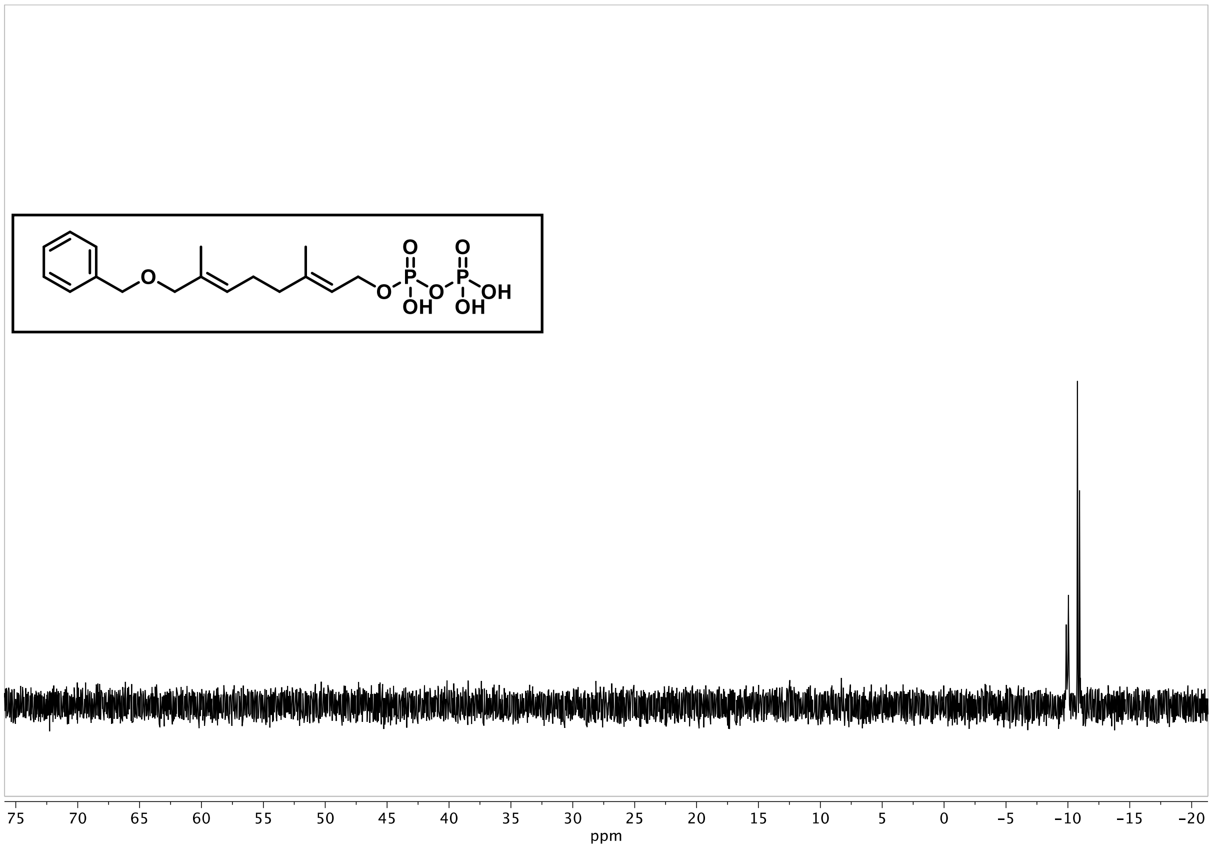

^{31}P NMR Spectrum of **44** (122 MHz, D_2O)

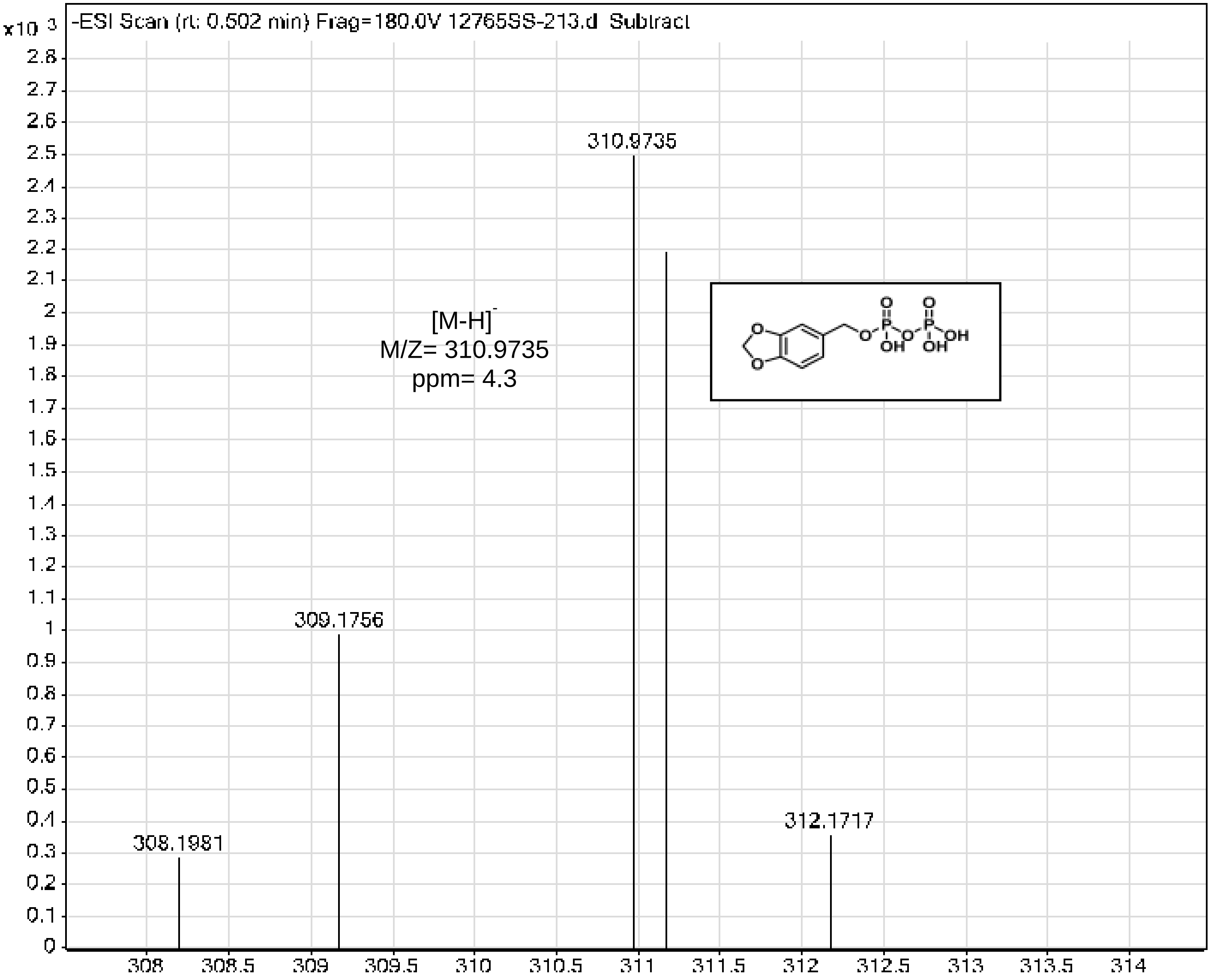

(-)-ESI-HRMS Spectrum of **58**

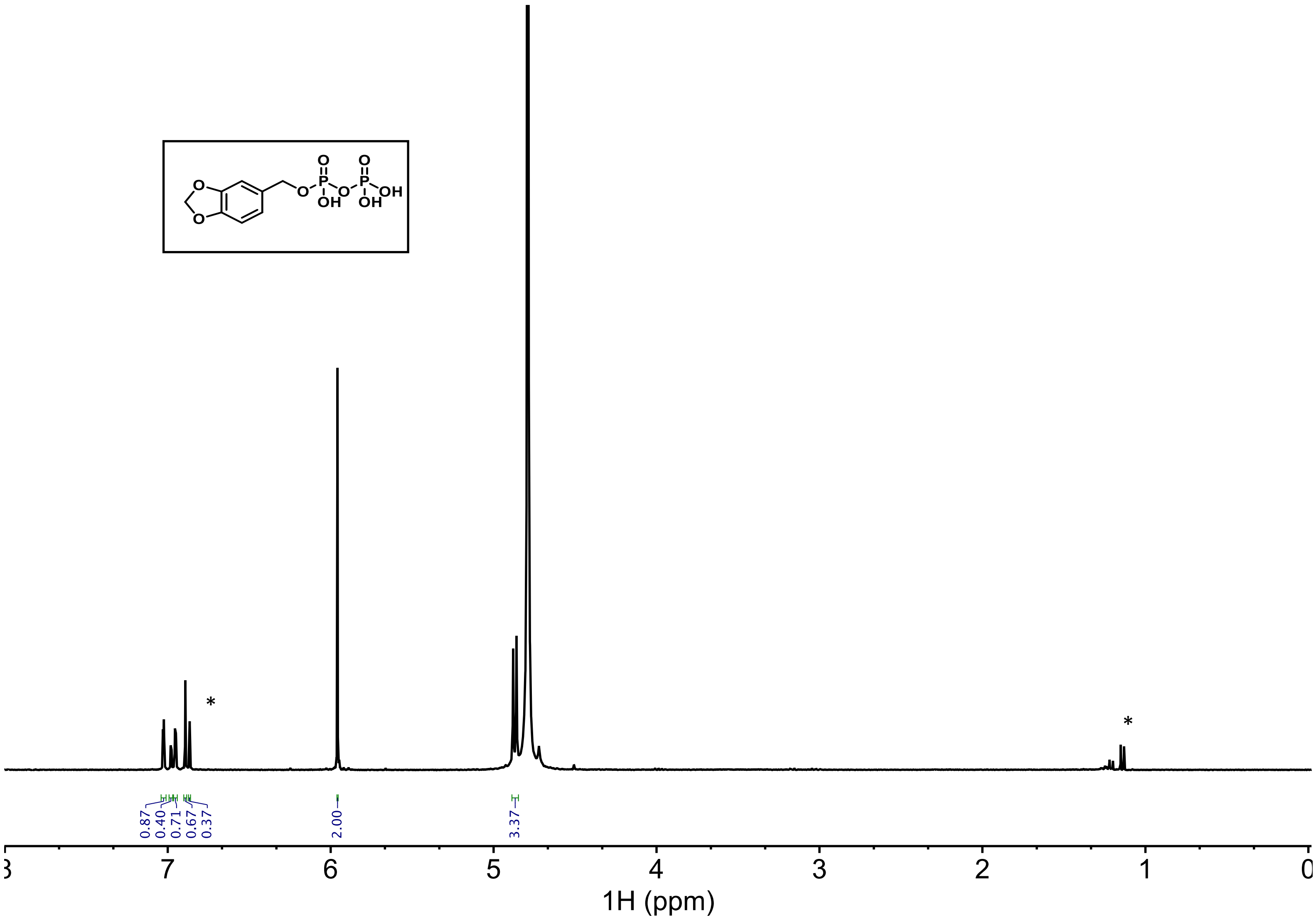

^{1}H NMR Spectrum of **58** (300 MHz, D_2O) *Denotes an impurity.

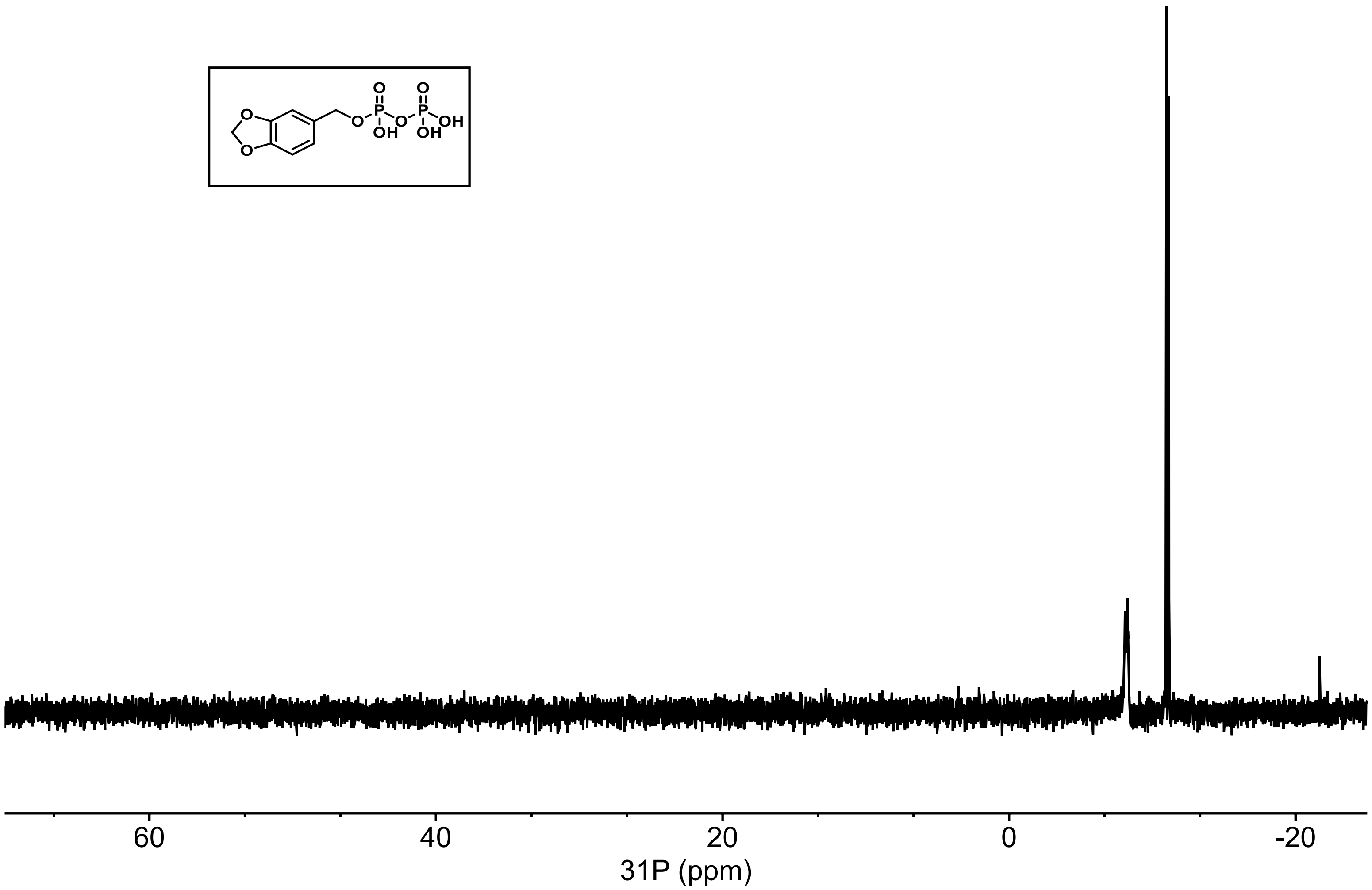

^{31}P NMR Spectrum of **58** (122 MHz, D_2O)

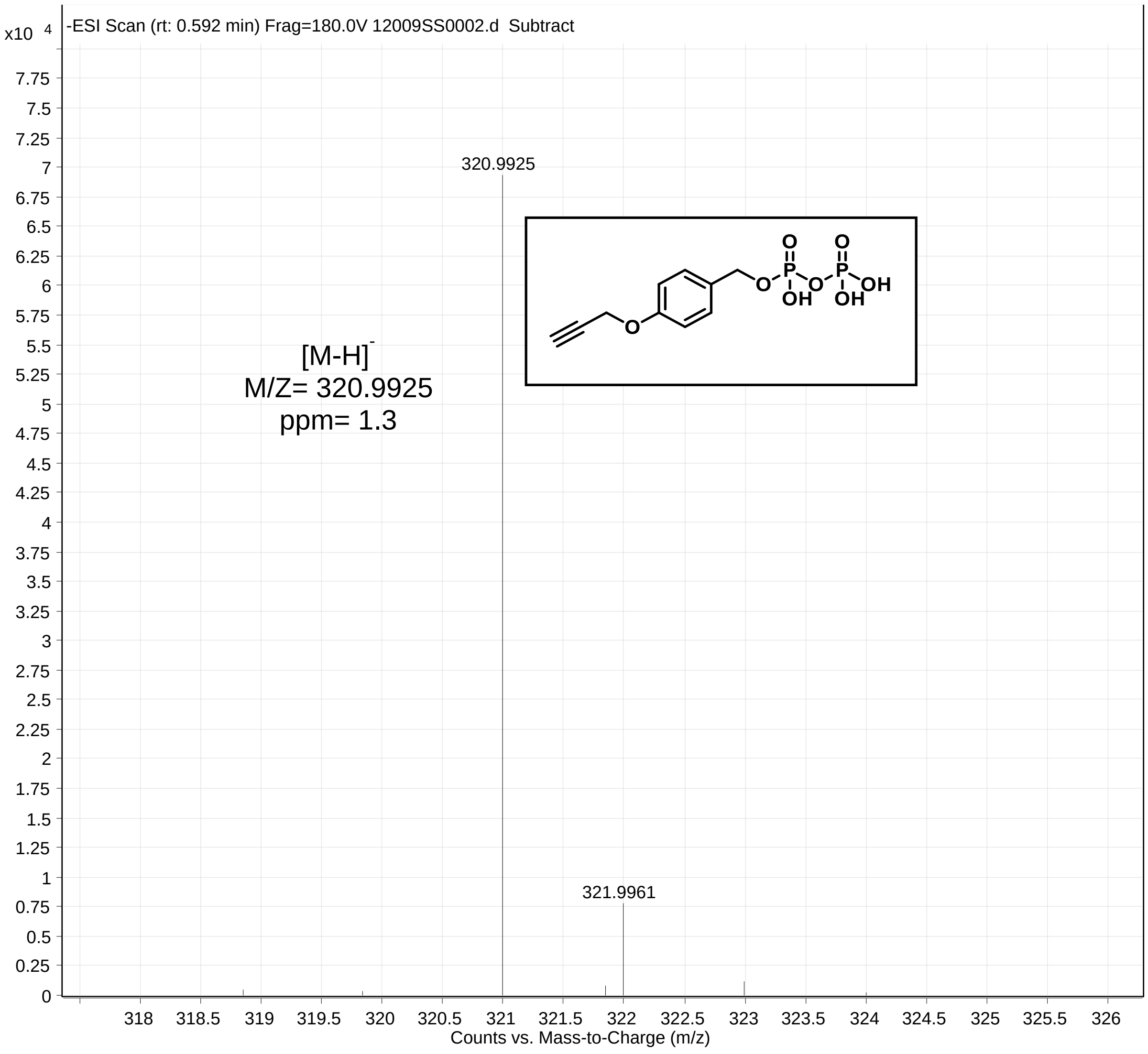

(-)-ESI-HRMS Spectrum of **59**

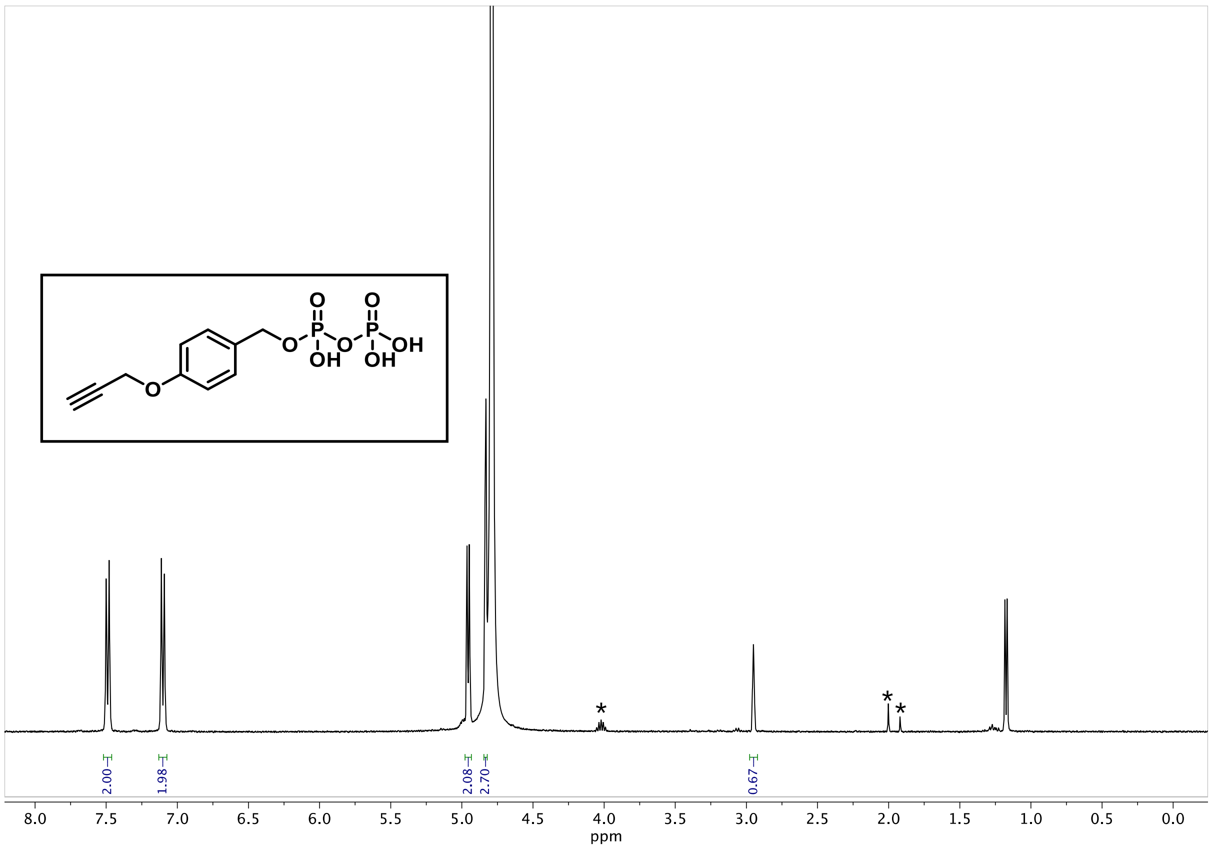

^{1}H NMR Spectrum of **59** (400 MHz, D_2O) *Denotes an impurity.

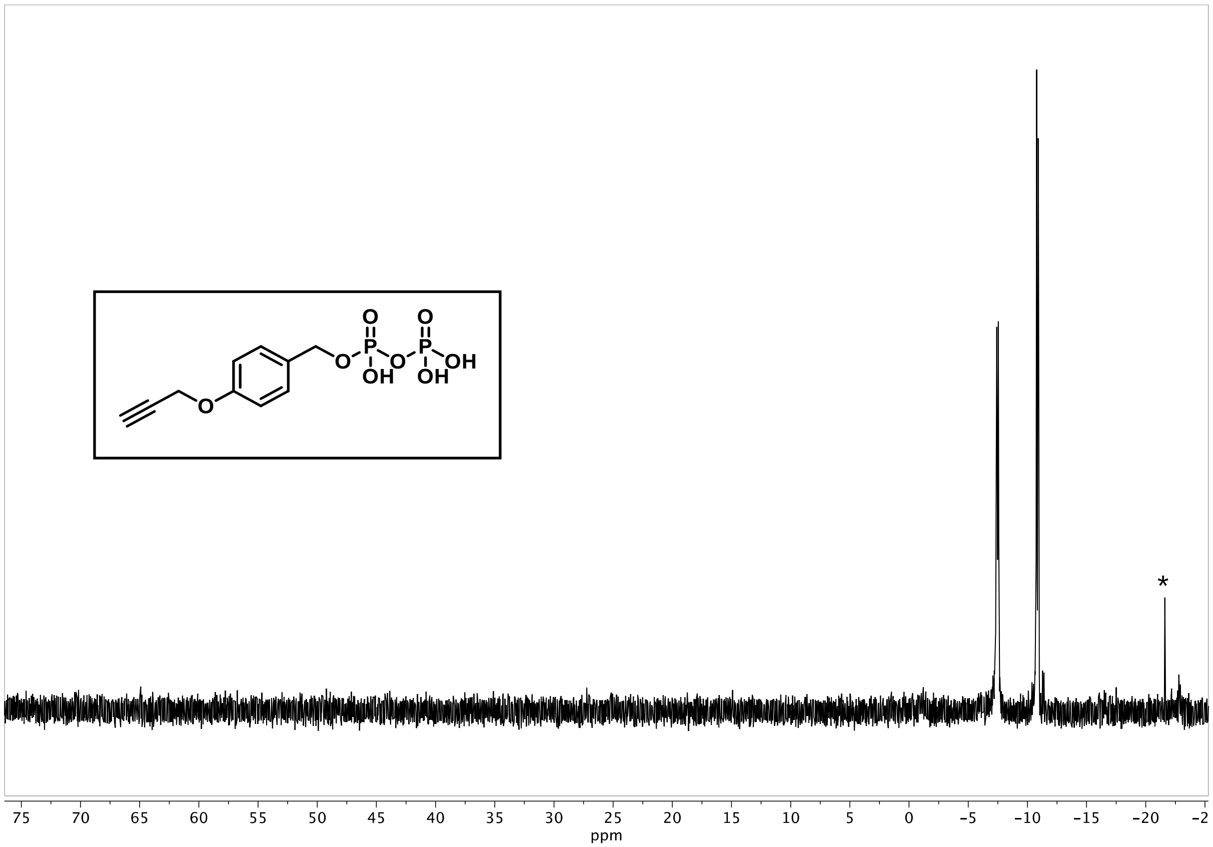

^{31}P NMR Spectrum of **59** (162 MHz, D_2O) *Denotes an impurity.

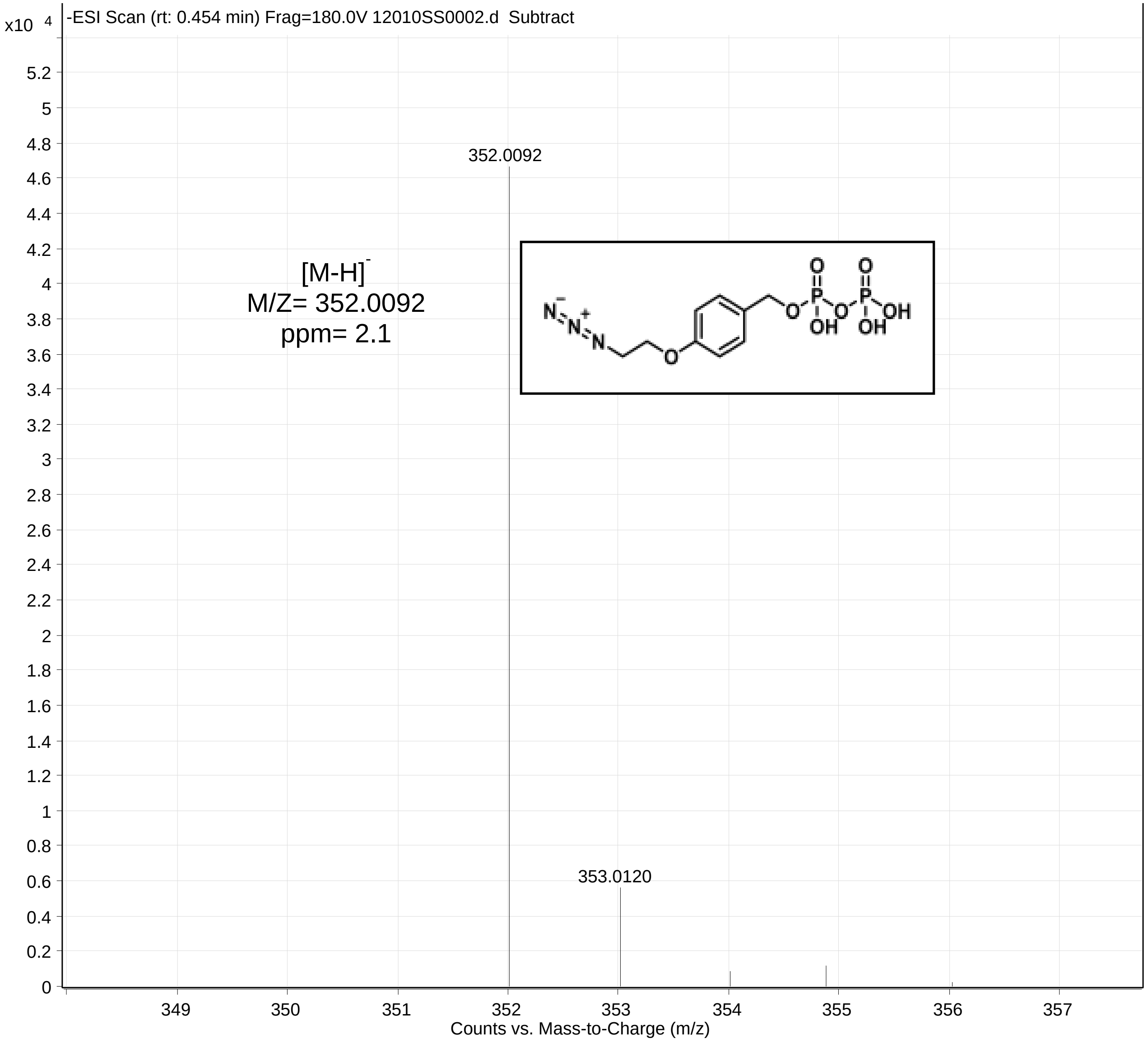

(-)-ESI-HRMS Spectrum of **60**

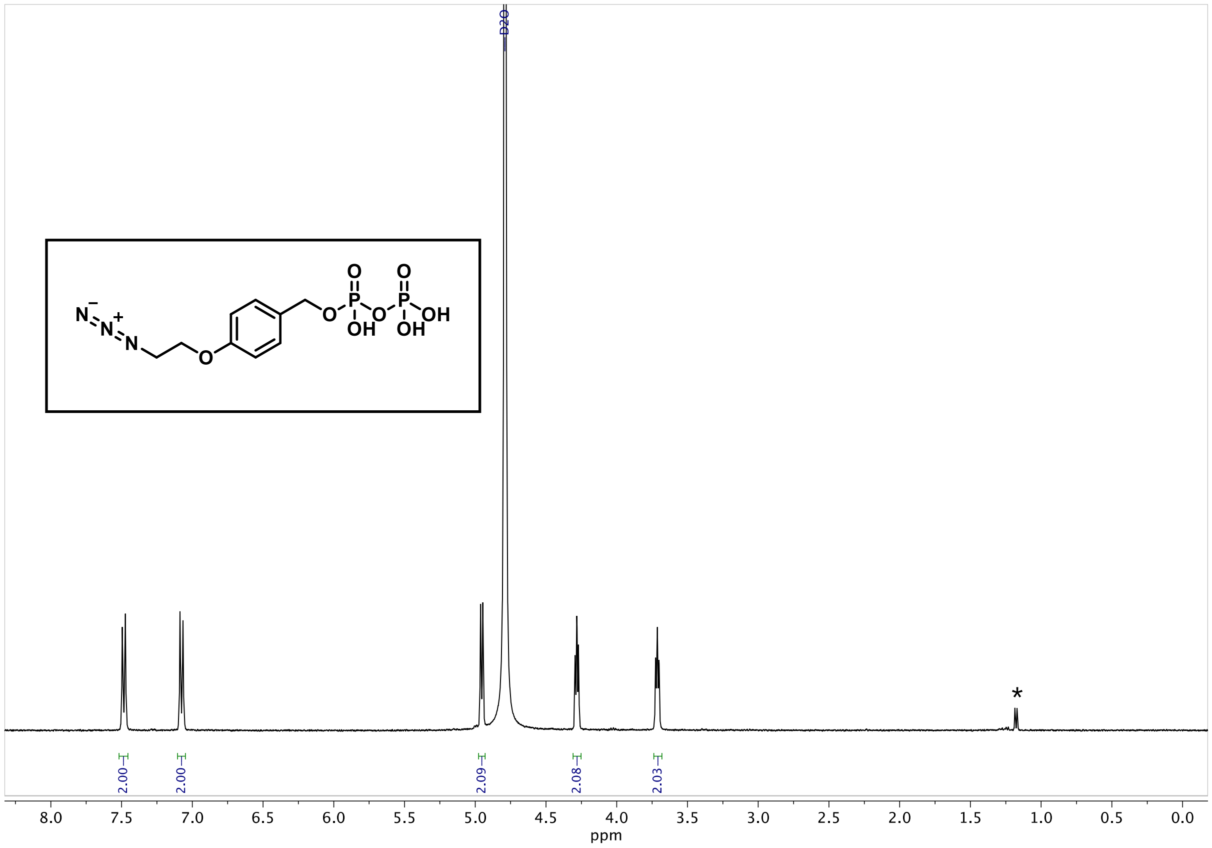

^{1}H NMR Spectrum of **60** (400 MHz, D_2O) *Denotes an impurity.

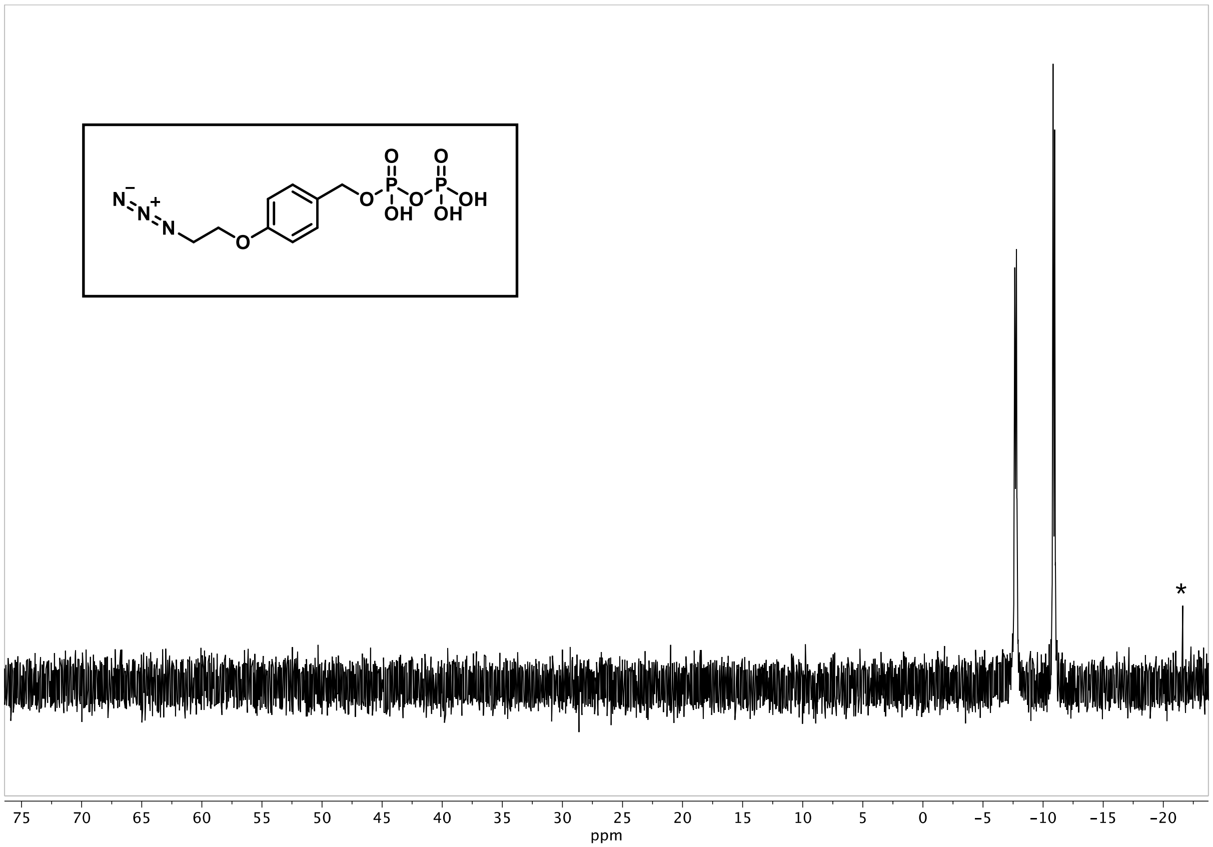

^{31}P NMR Spectrum of **60** (162 MHz, D_2O) *Denotes an impurity.

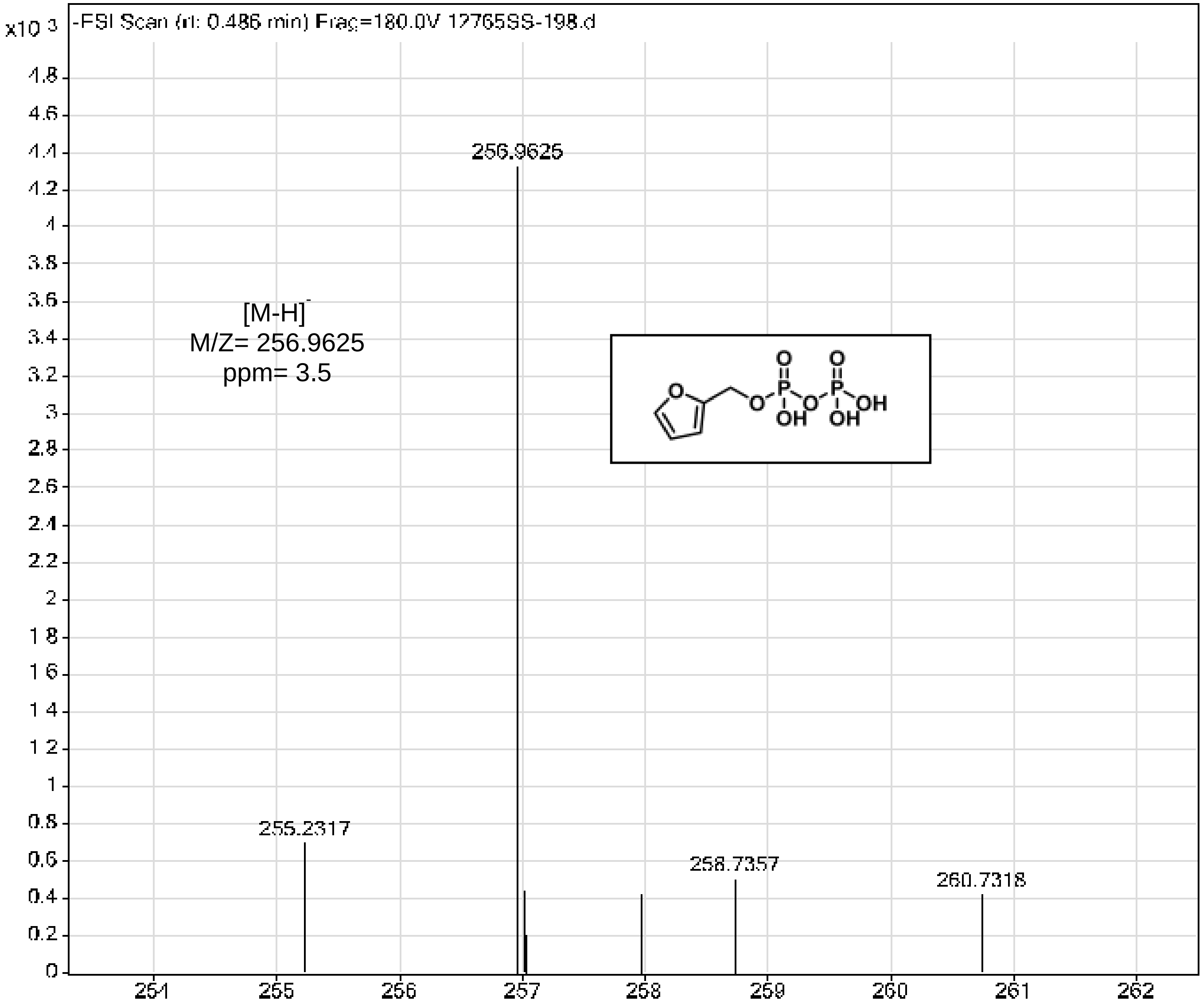

(-)-ESI-HRMS Spectrum of **61**

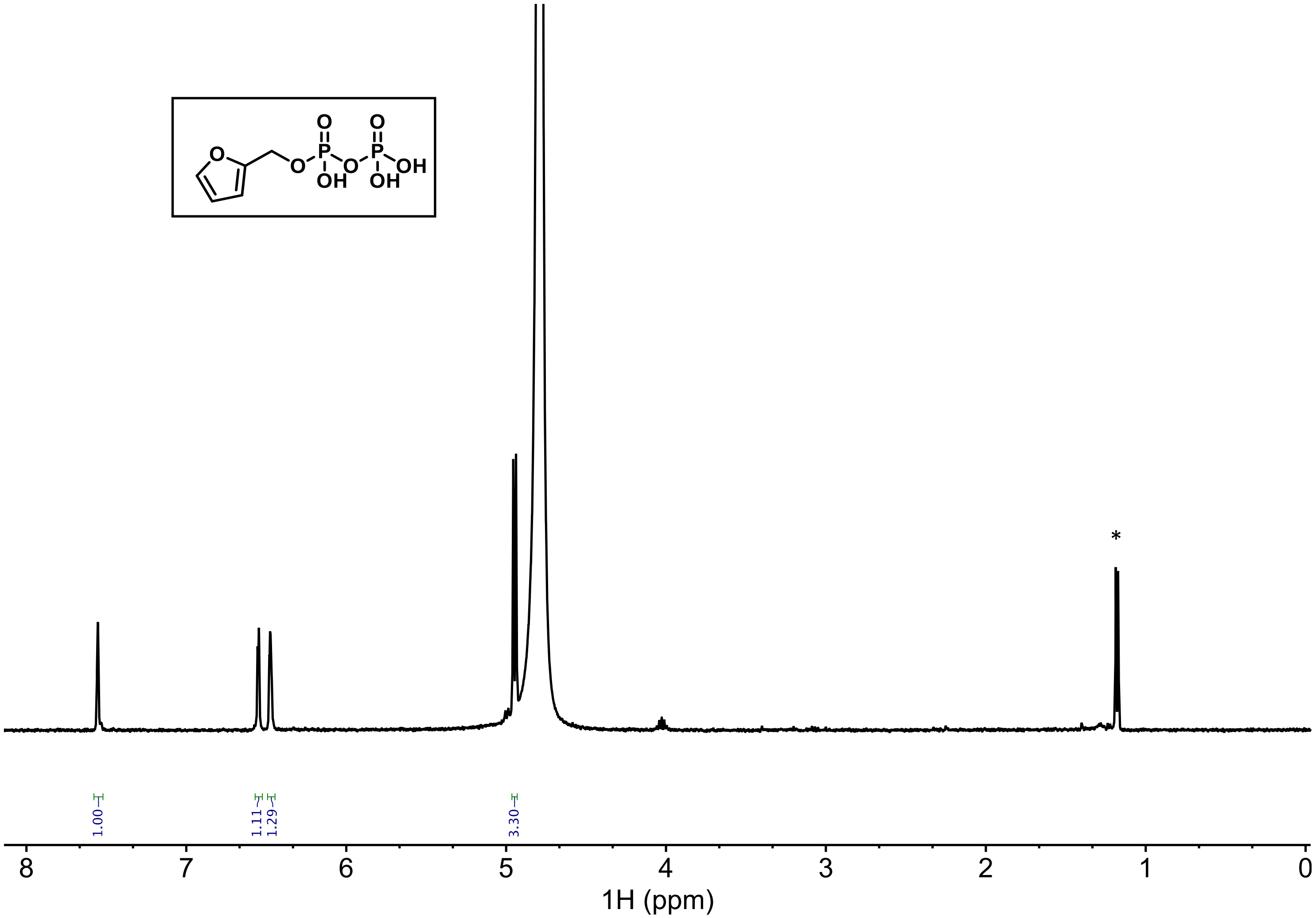

^{1}H NMR Spectrum of **61** (400 MHz, D_2O) *Denotes an impurity.

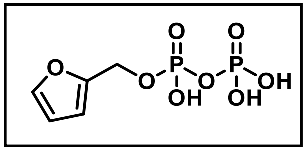

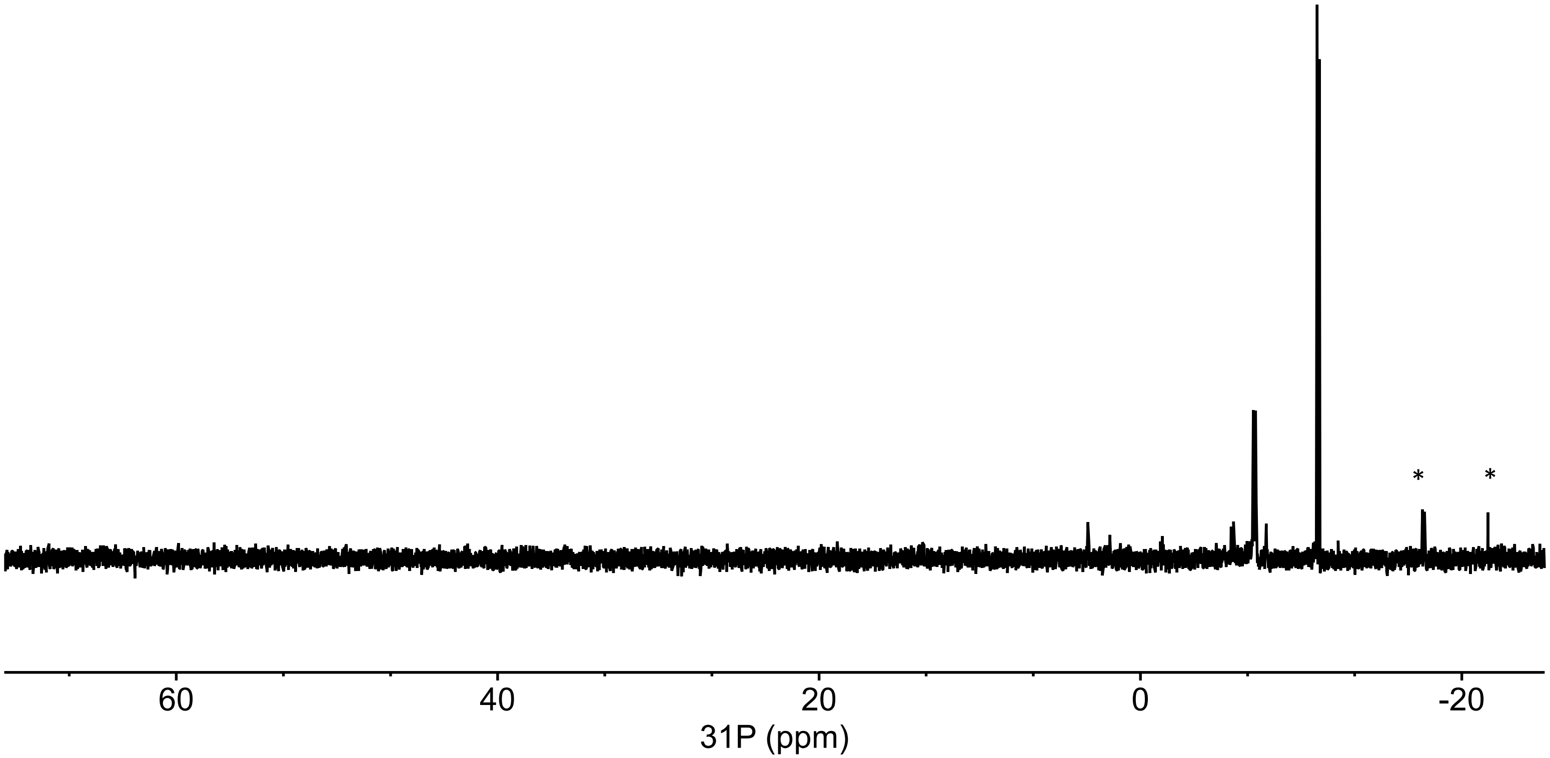

^{31}P NMR Spectrum of **61** (162 MHz, D_2O) *Denotes an impurity.

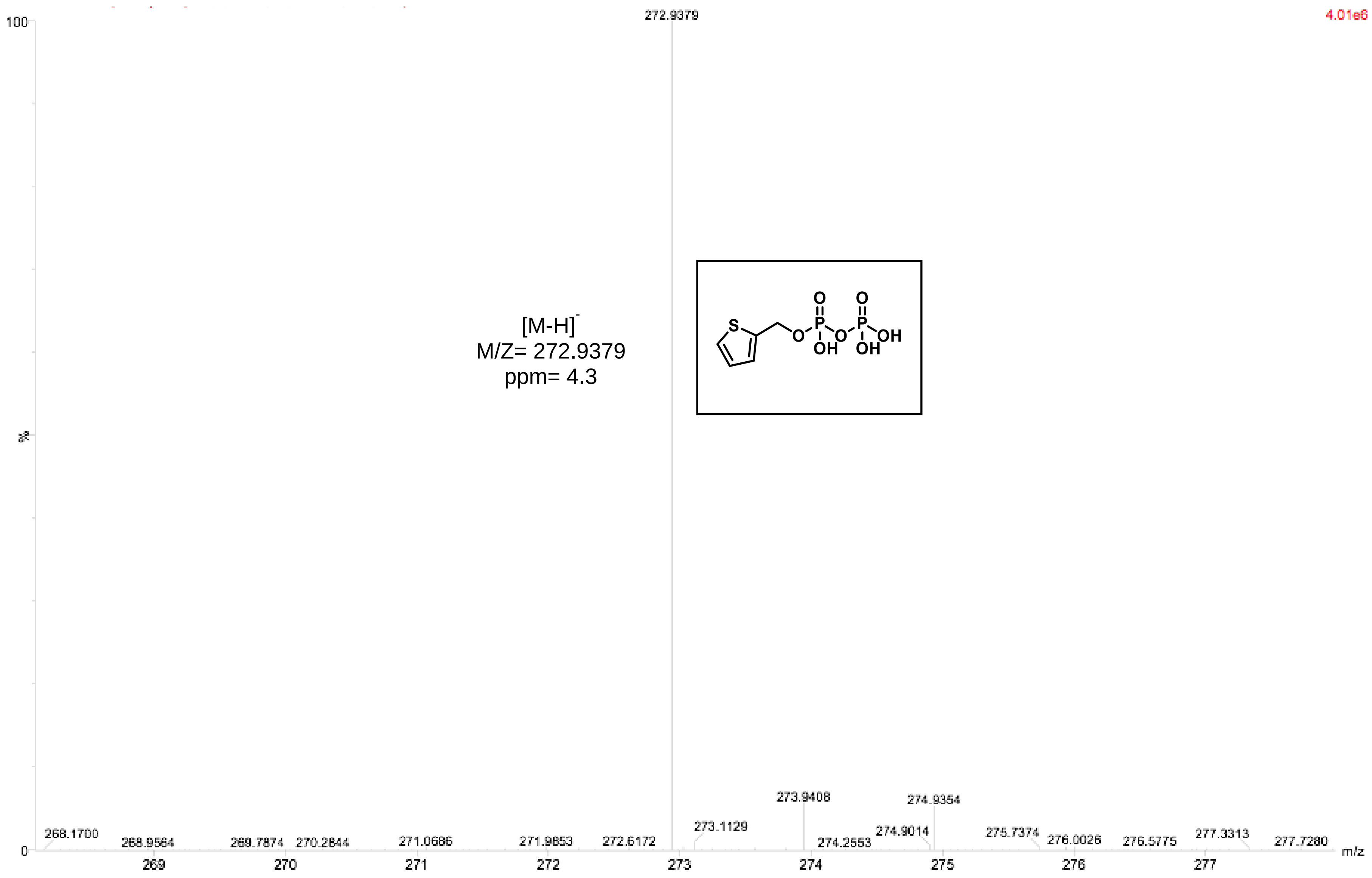

(-)-ESI-HRMS Spectrum of **62**

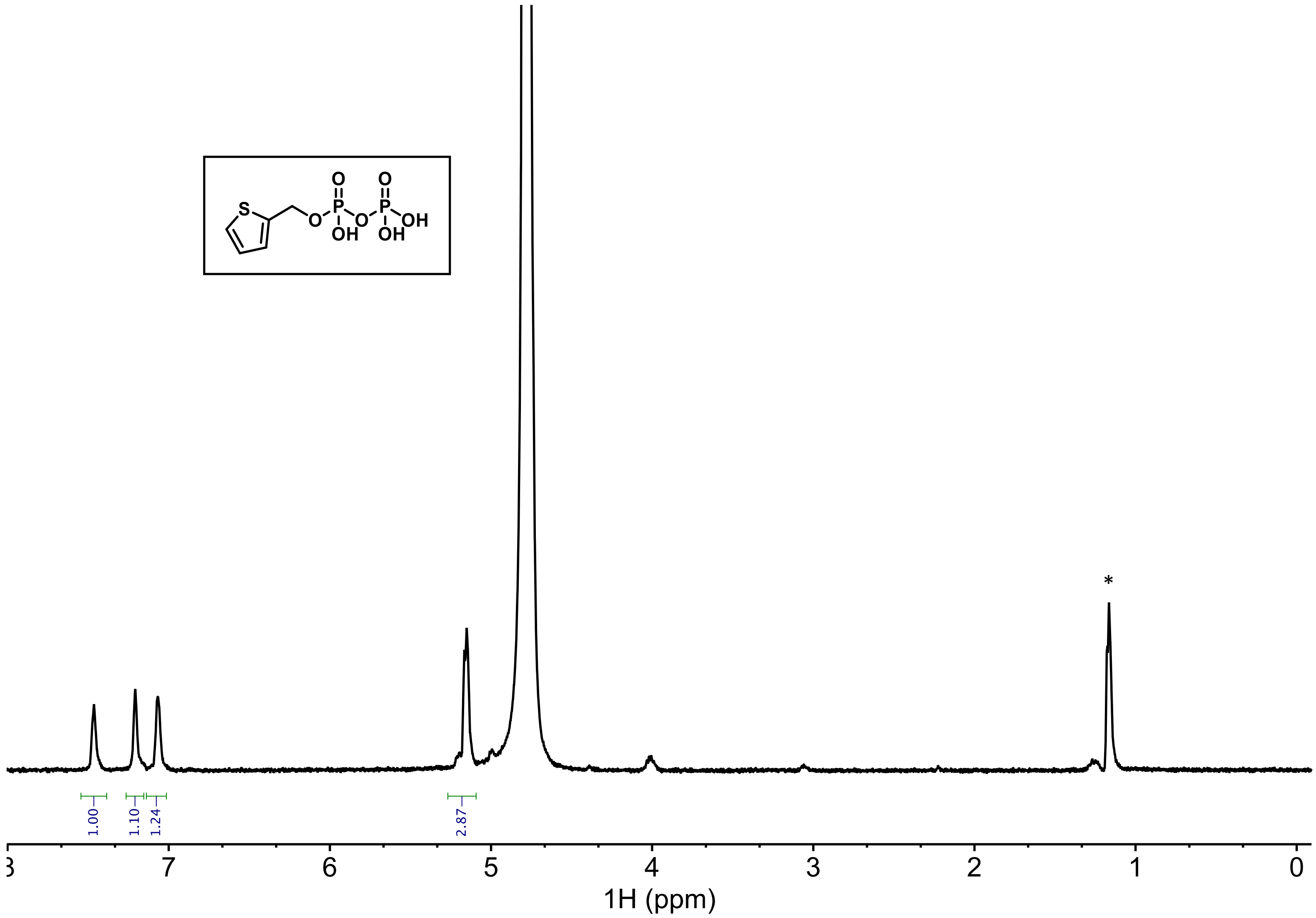

^{1}H NMR Spectrum of **62** (400 MHz, D_2O) *Denotes an impurity.

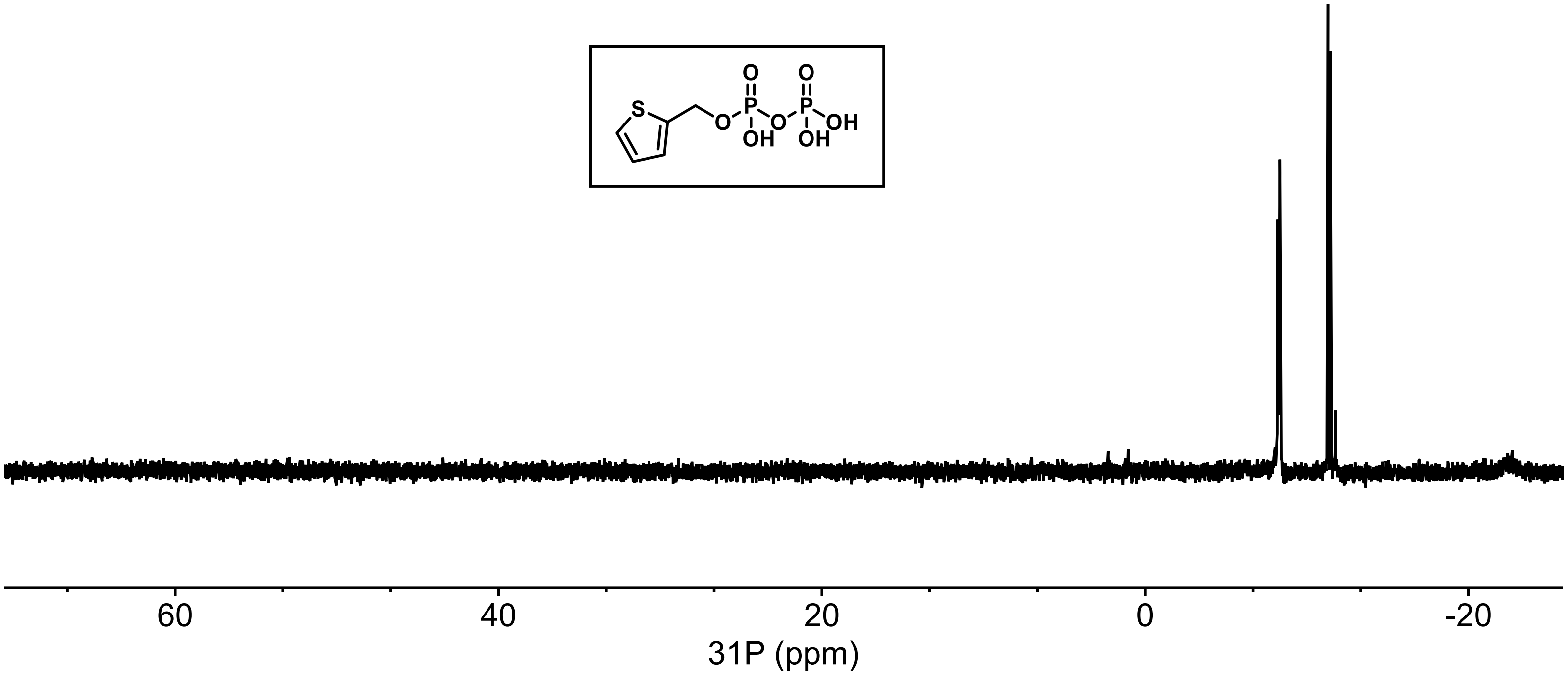

^{31}P NMR Spectrum of **62** (162 MHz, D_2O)

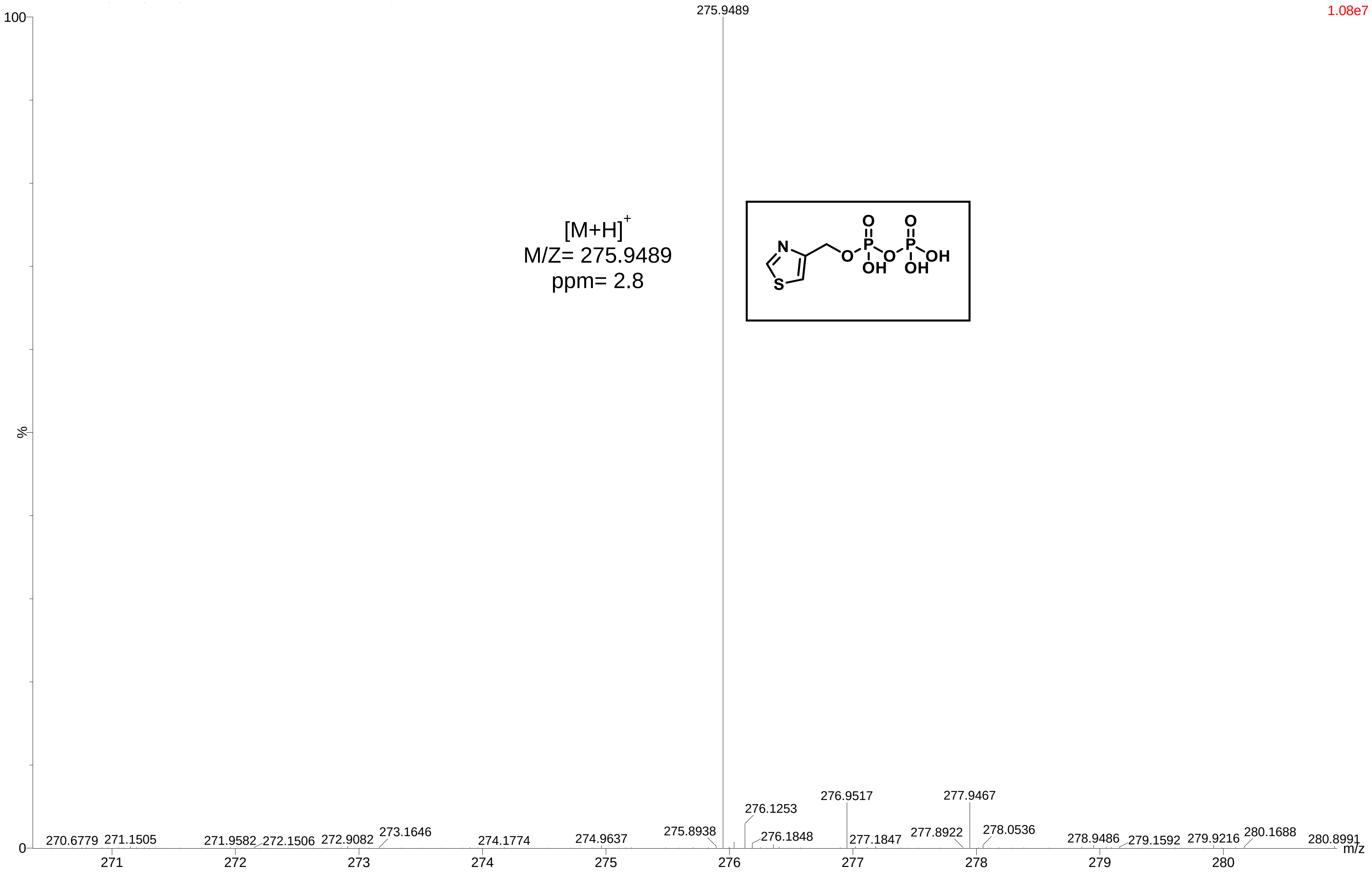

(+)-ESI-HRMS Spectrum of **63**

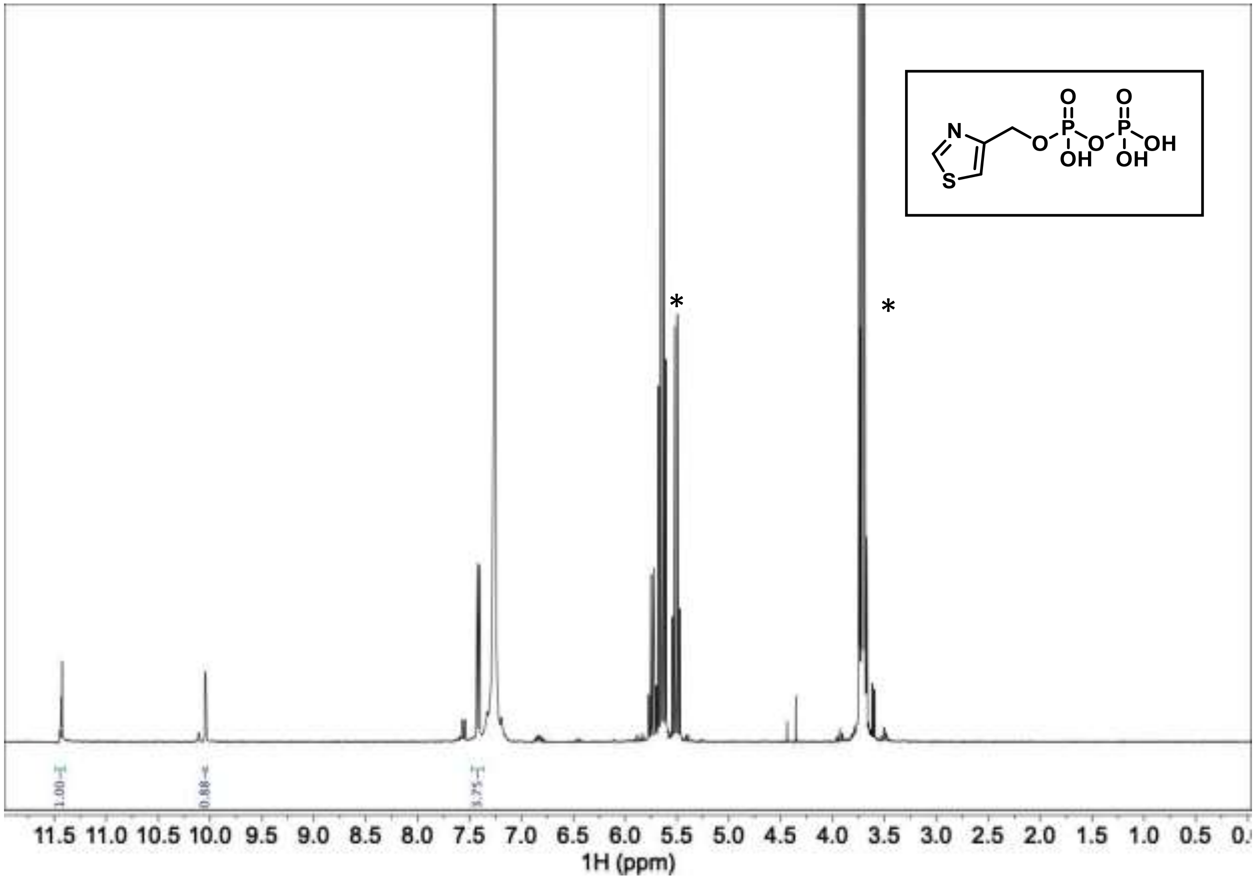

^{1}H NMR Spectrum of **63** (300 MHz, $CDCl_3$) *Denotes an impurity.

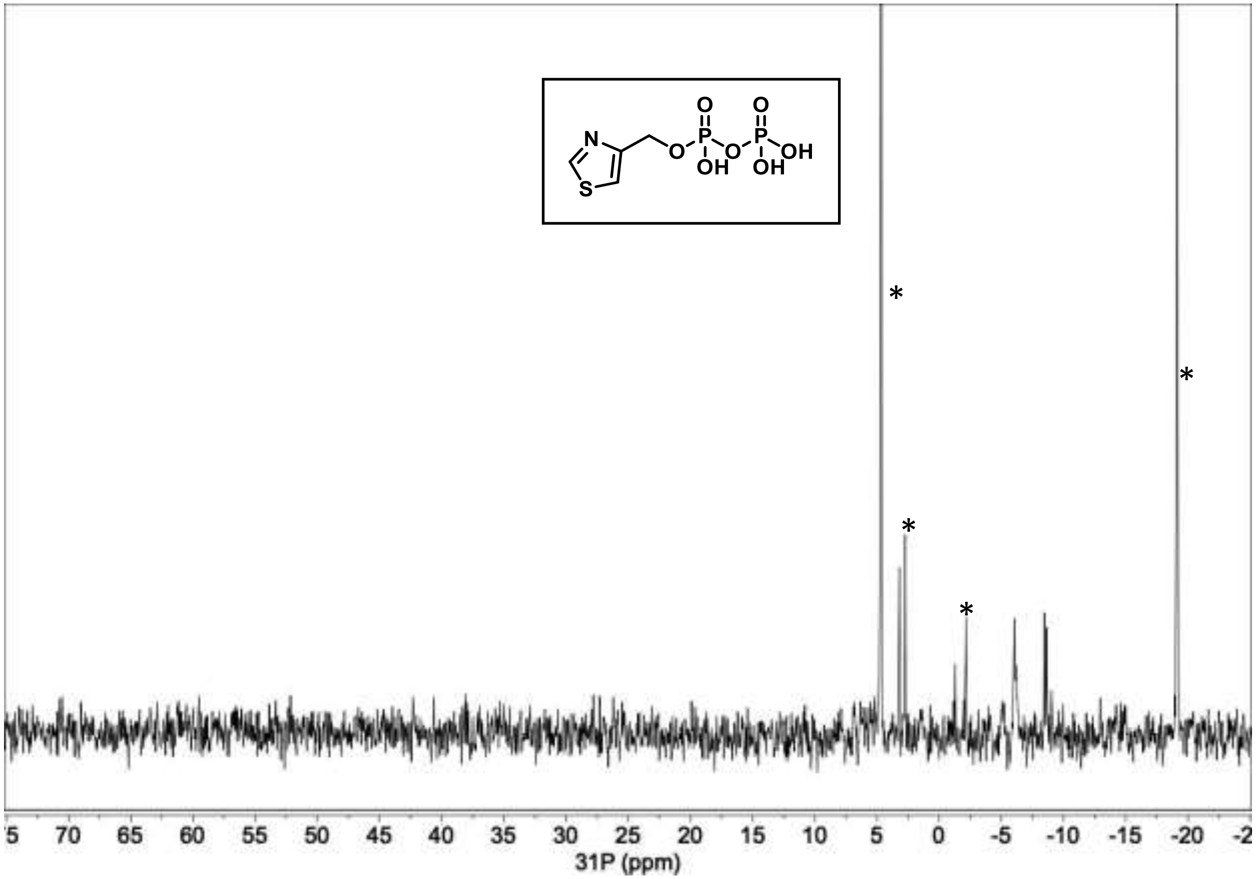

^{31}P NMR Spectrum of **63** (122 MHz, $CDCl_3$) *Denotes an impurity.

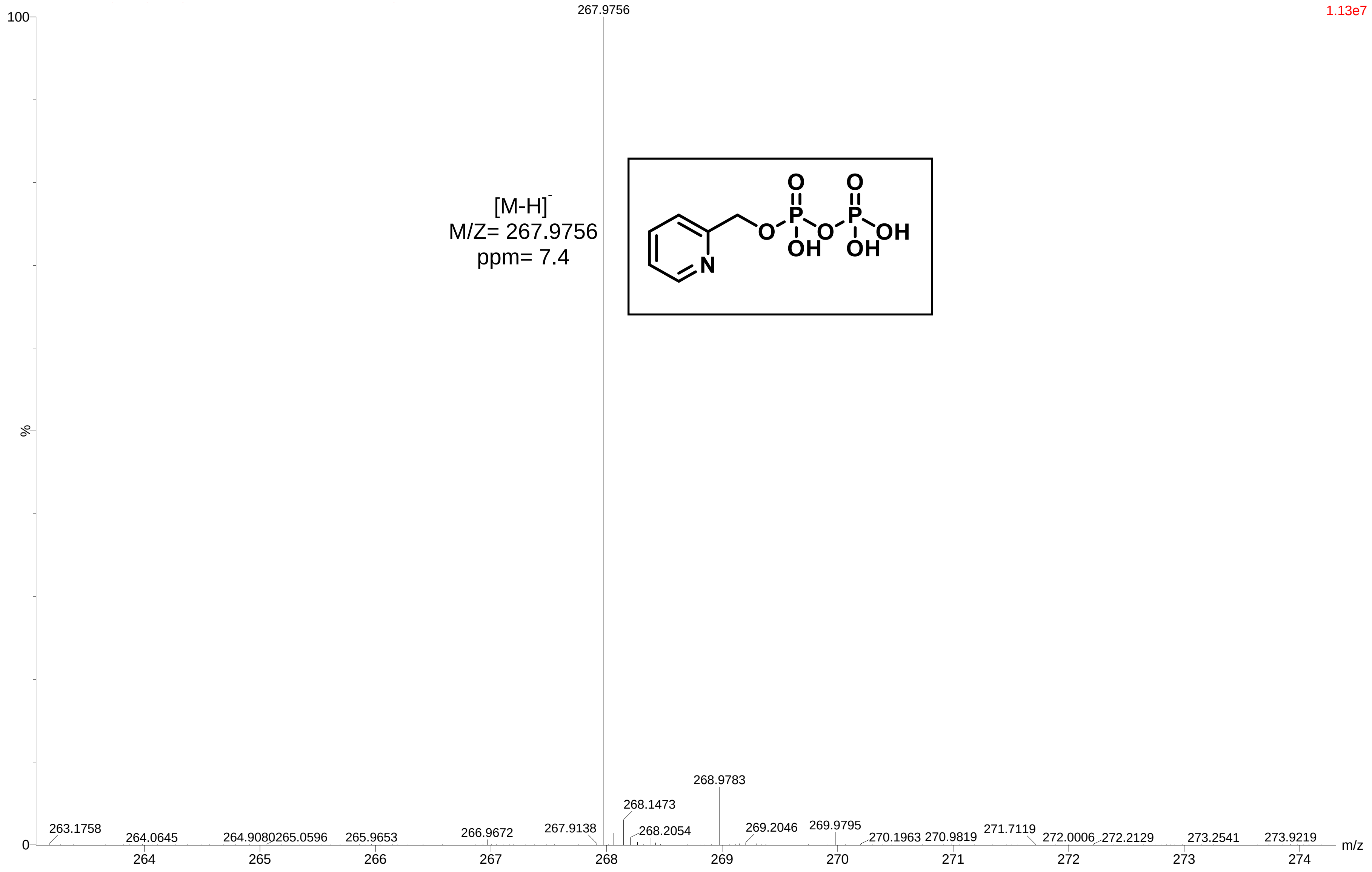

(-)-ESI-MS Spectrum of **64**

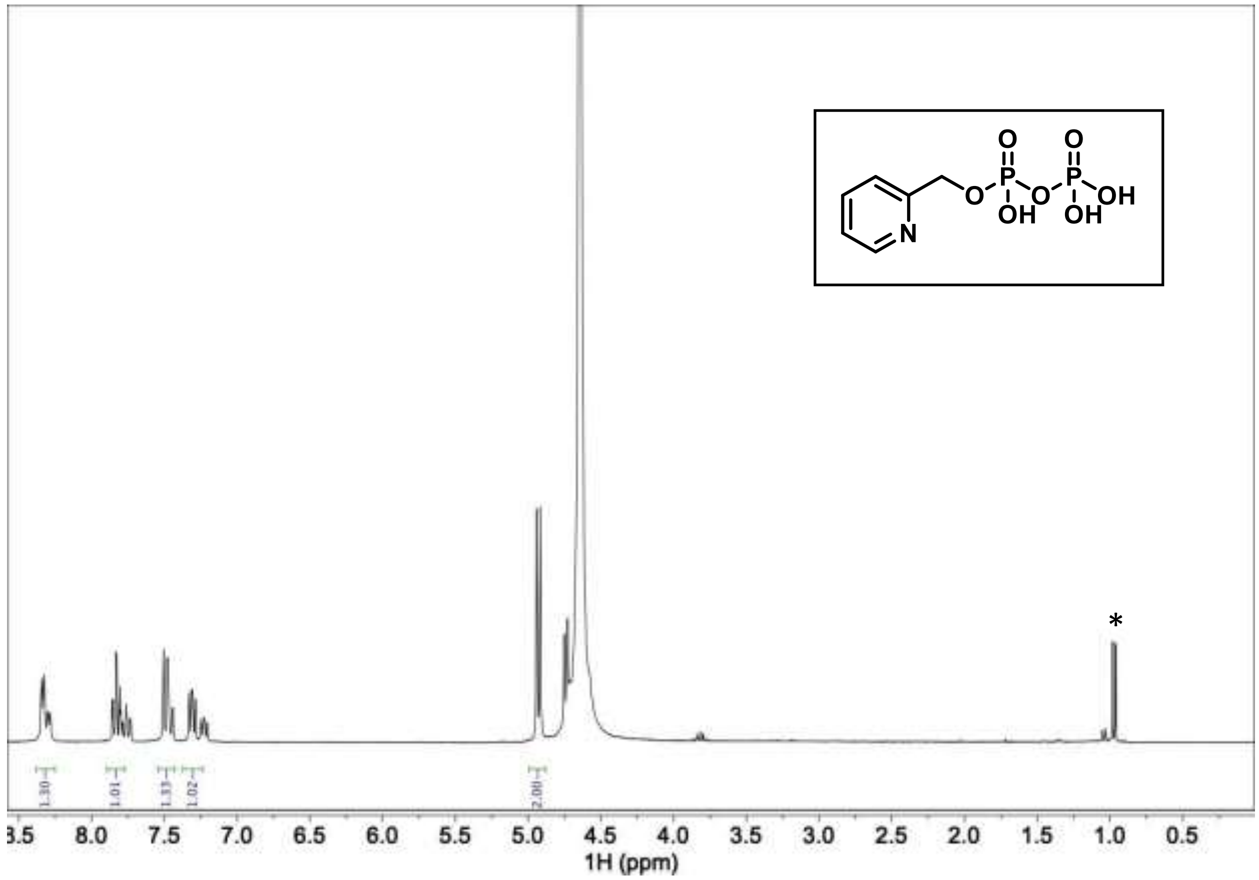

^{1}H NMR Spectrum of **64** (300 MHz, D_2O) *Denotes an impurity.

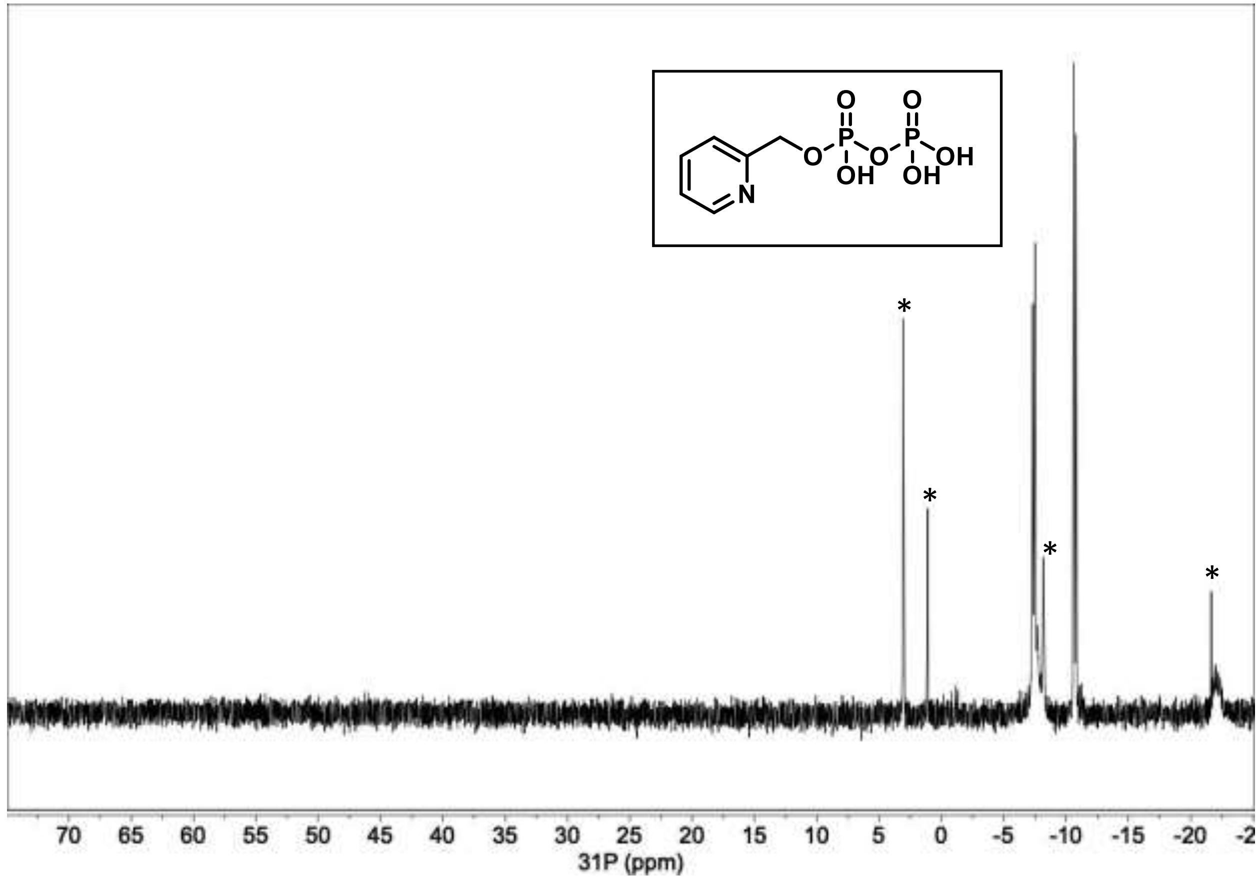

^{31}P NMR Spectrum of **64** (122 MHz, D_2O) *Denotes an impurity.

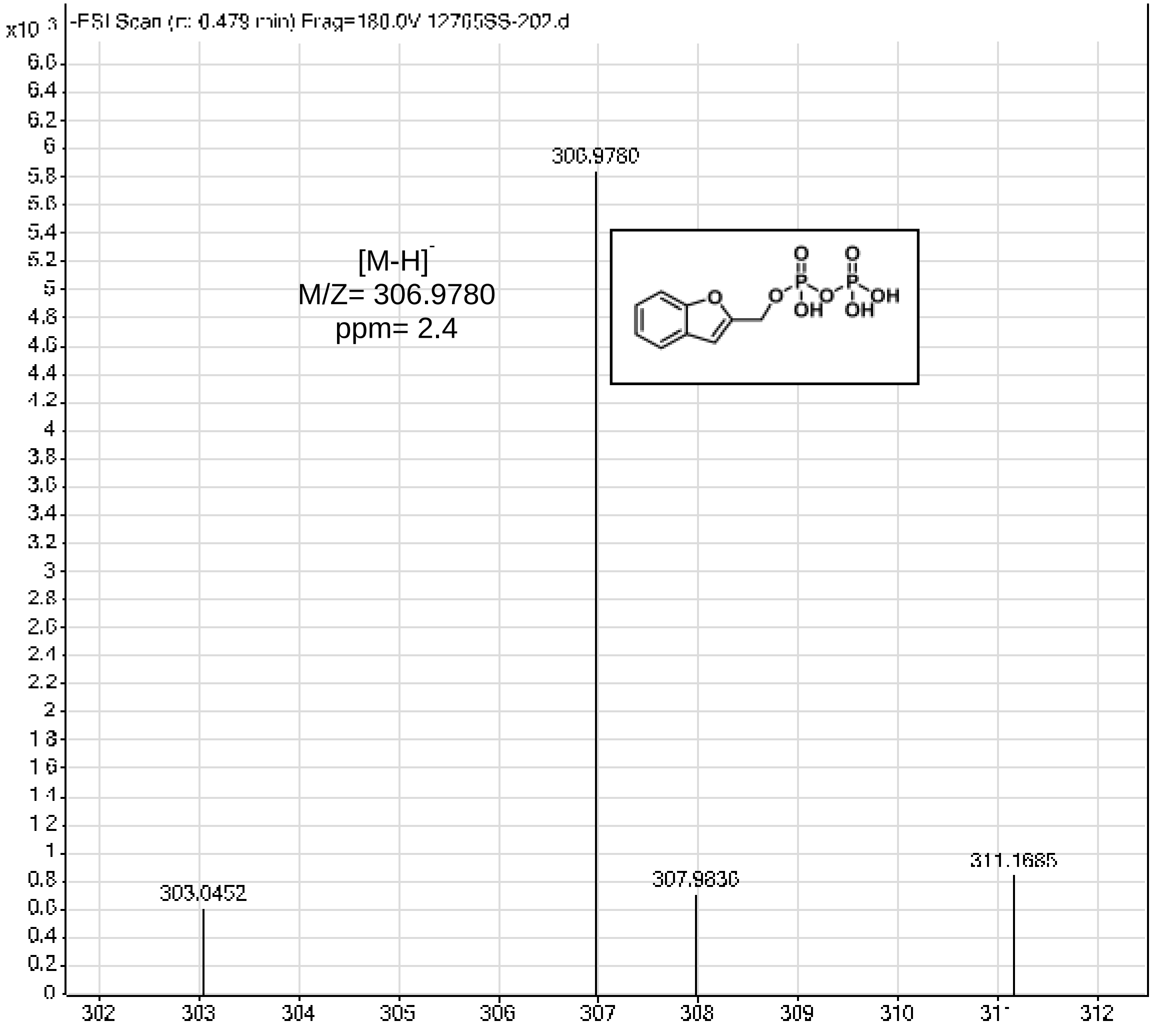

(-)-ESI-HRMS Spectrum of **65**

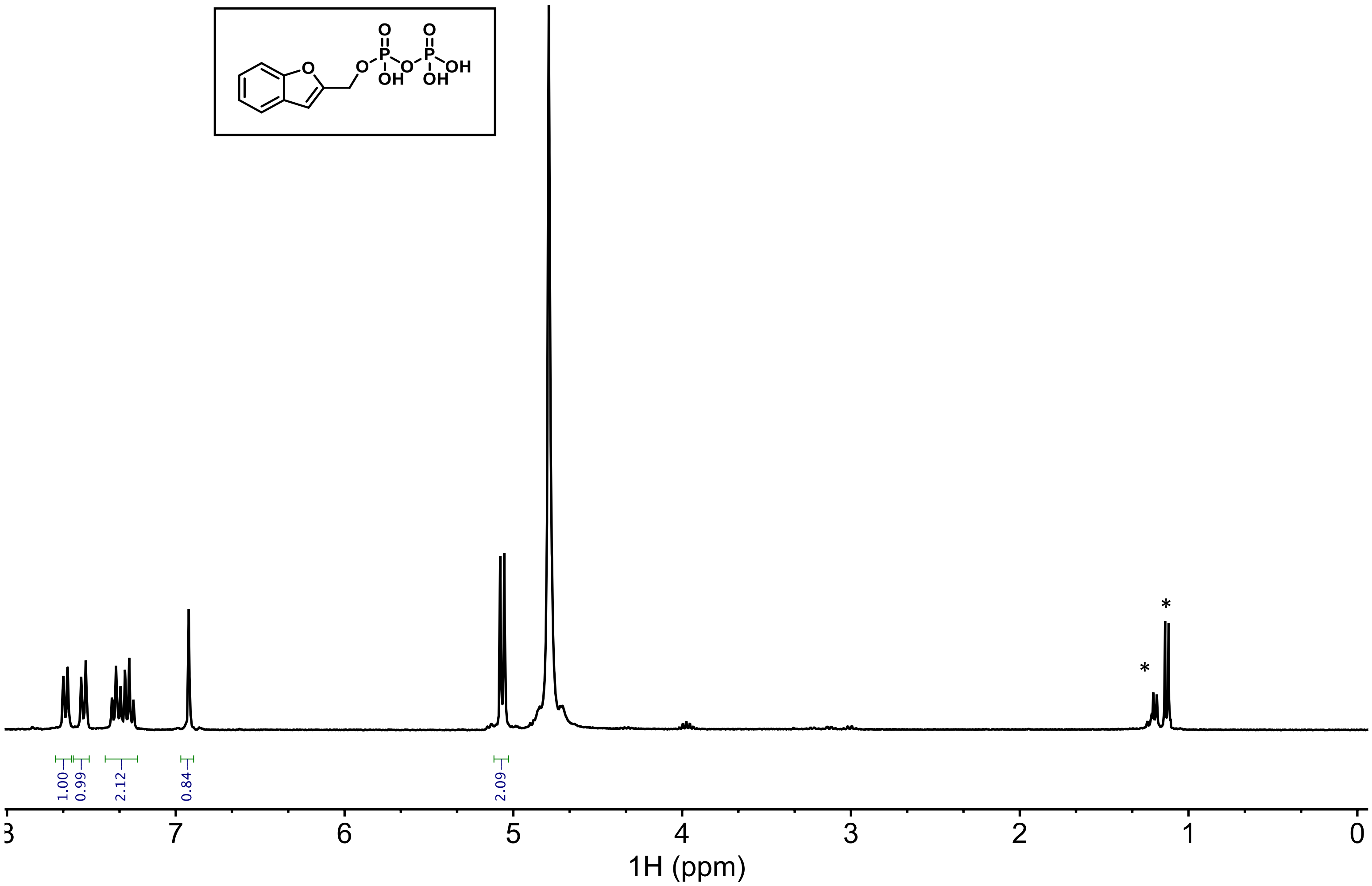

^{1}H NMR Spectrum of **65** (300 MHz, D_2O) *Denotes an impurity.

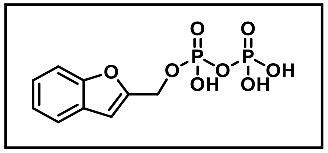

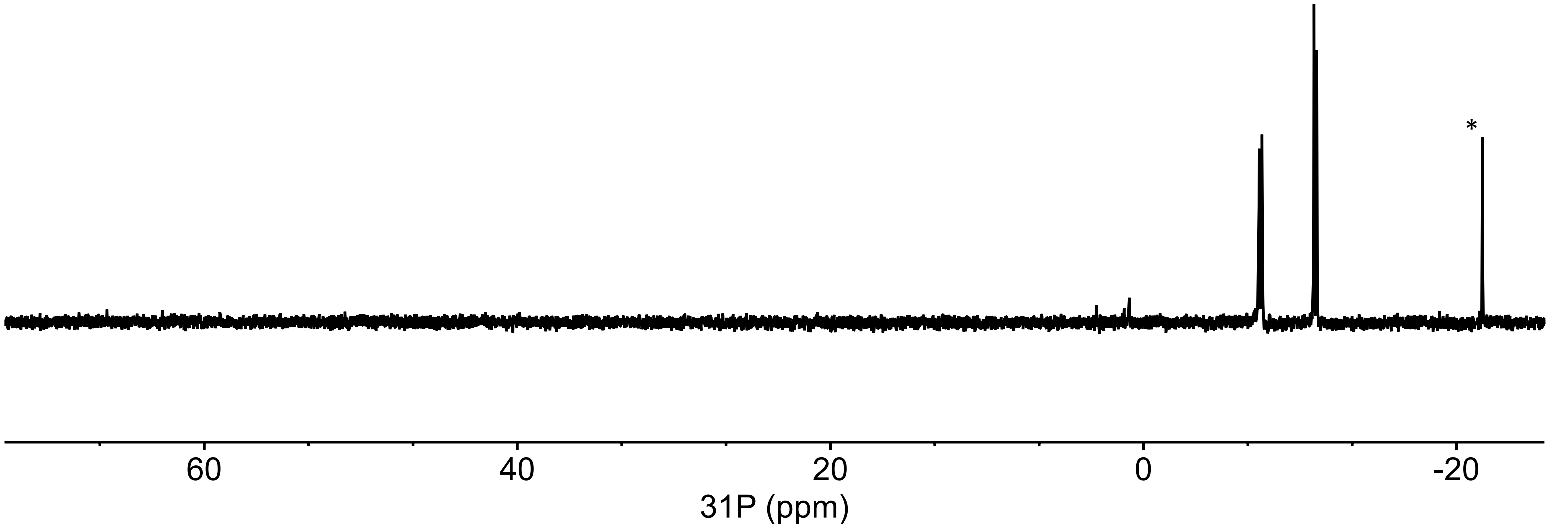

^{31}P NMR Spectrum of **65** (122 MHz, D_2O). *Denotes an impurity.

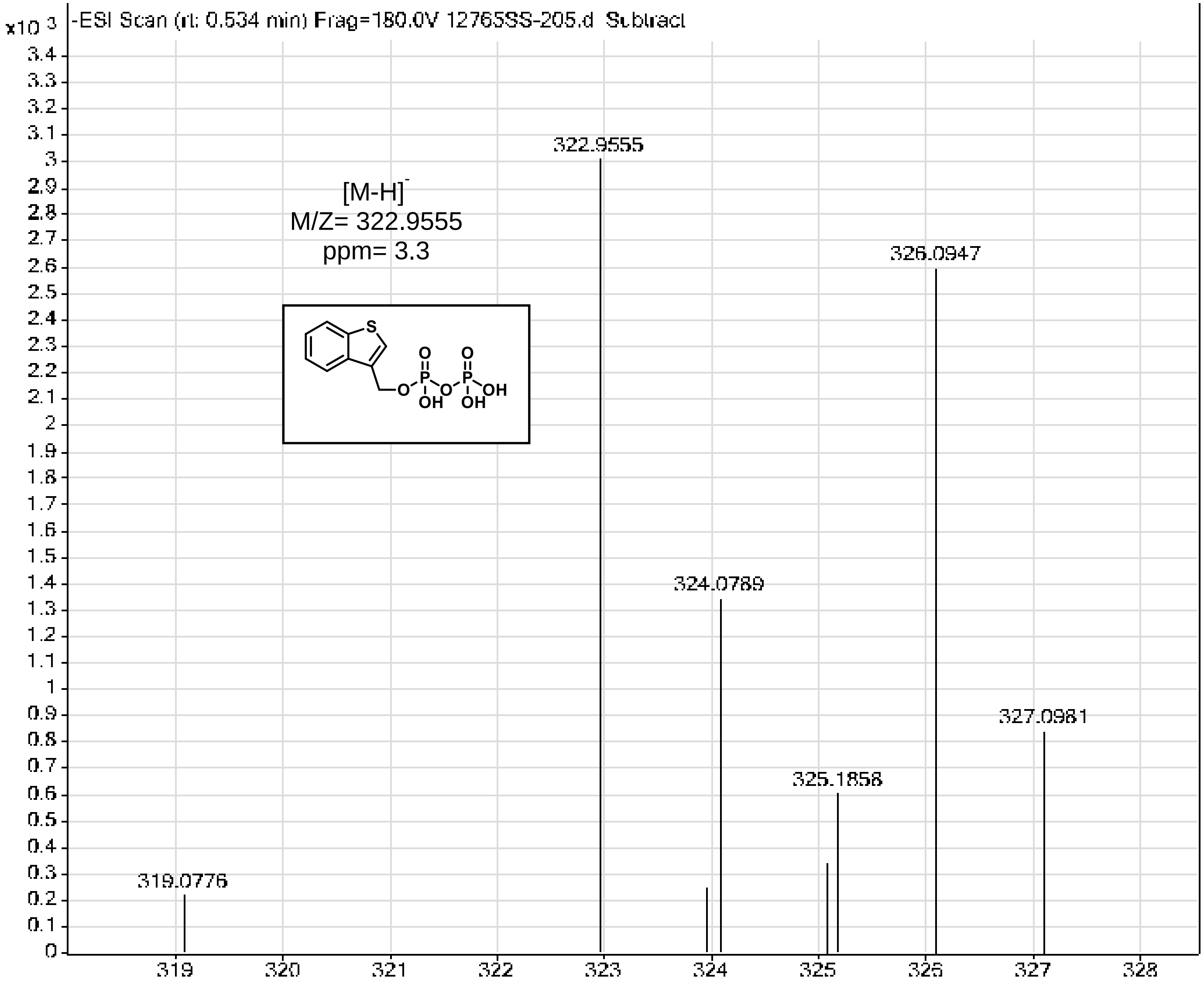

(-)-ESI-HRMS Spectrum of **66**

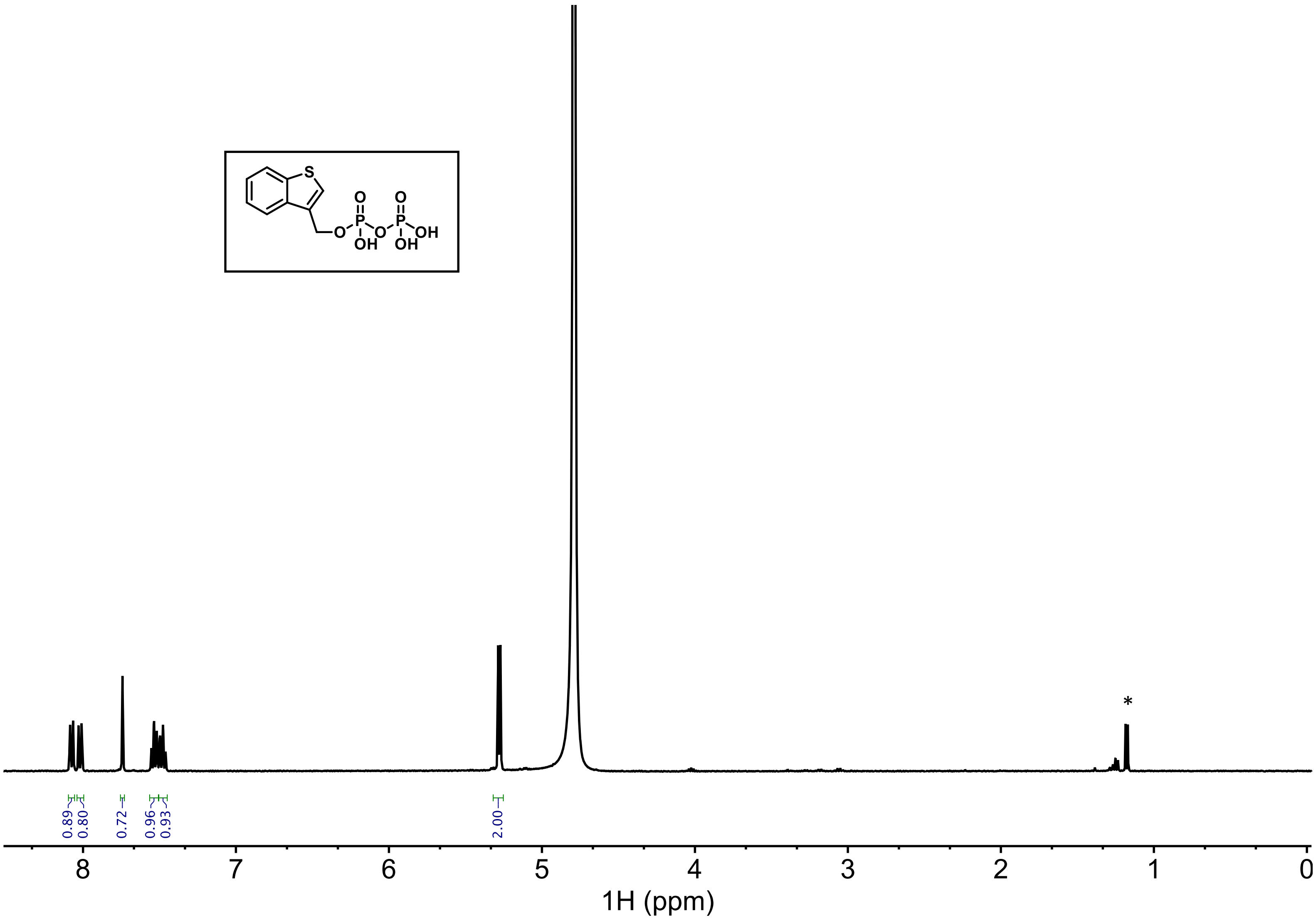

^{1}H NMR Spectrum of **66** (400 MHz, D_2O) *Denotes an impurity.

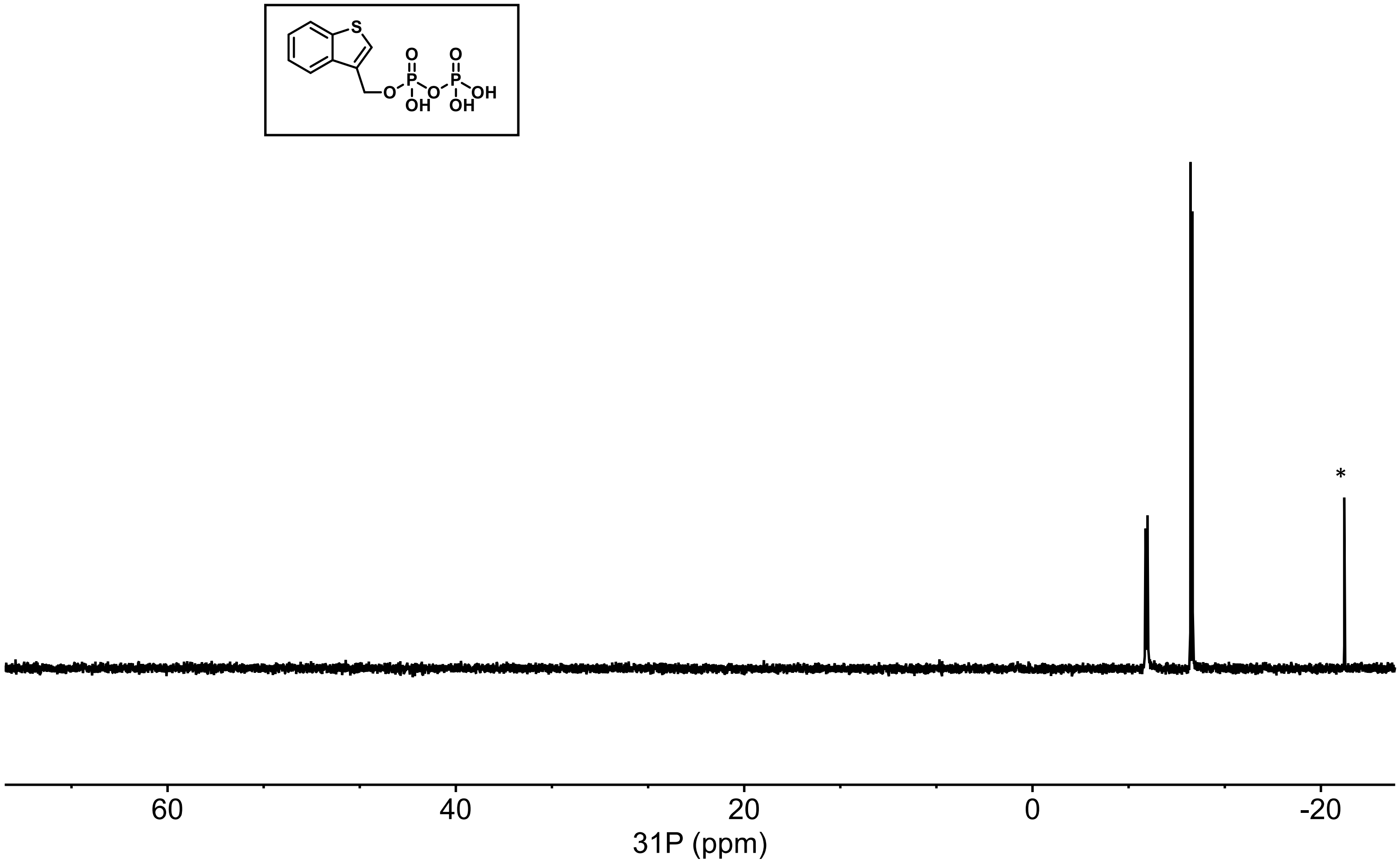

^{31}P NMR Spectrum of **66** (162 MHz, D_2O) *Denotes an impurity.

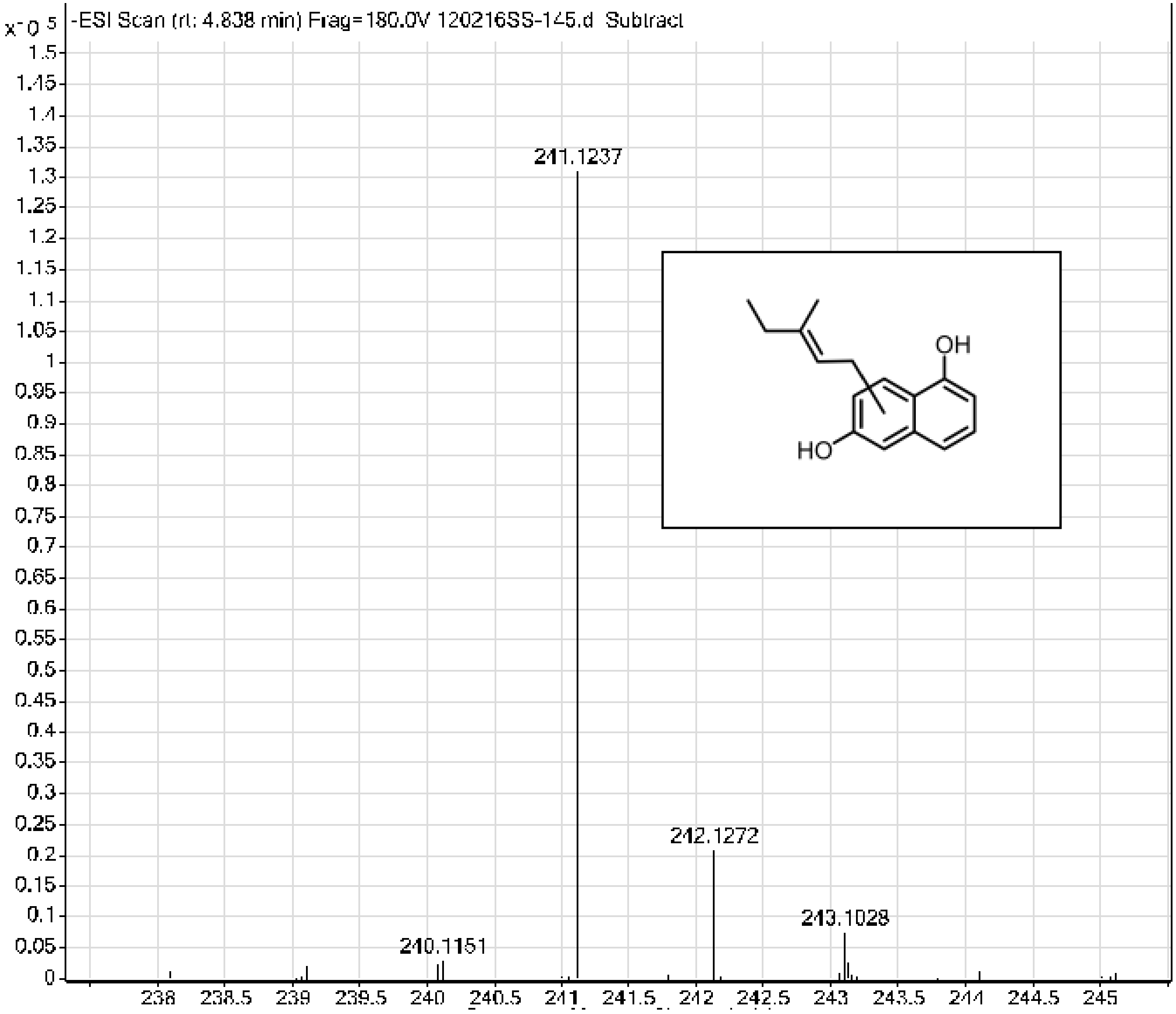

(-)-ESI-HRMS Spectrum of 1,6-DHN-**7**

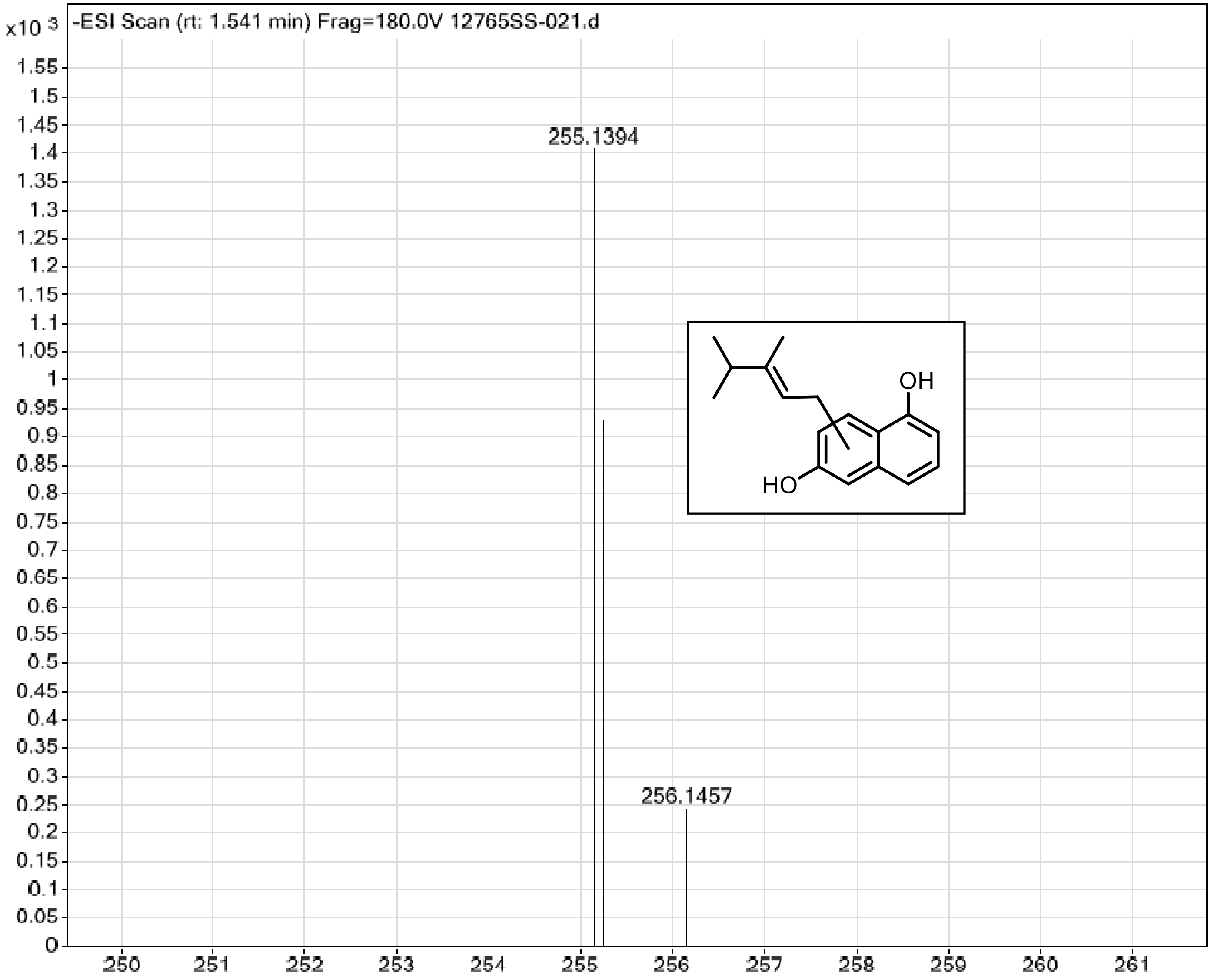

(-)-ESI-HRMS Spectrum of 1,6-DHN-**10**

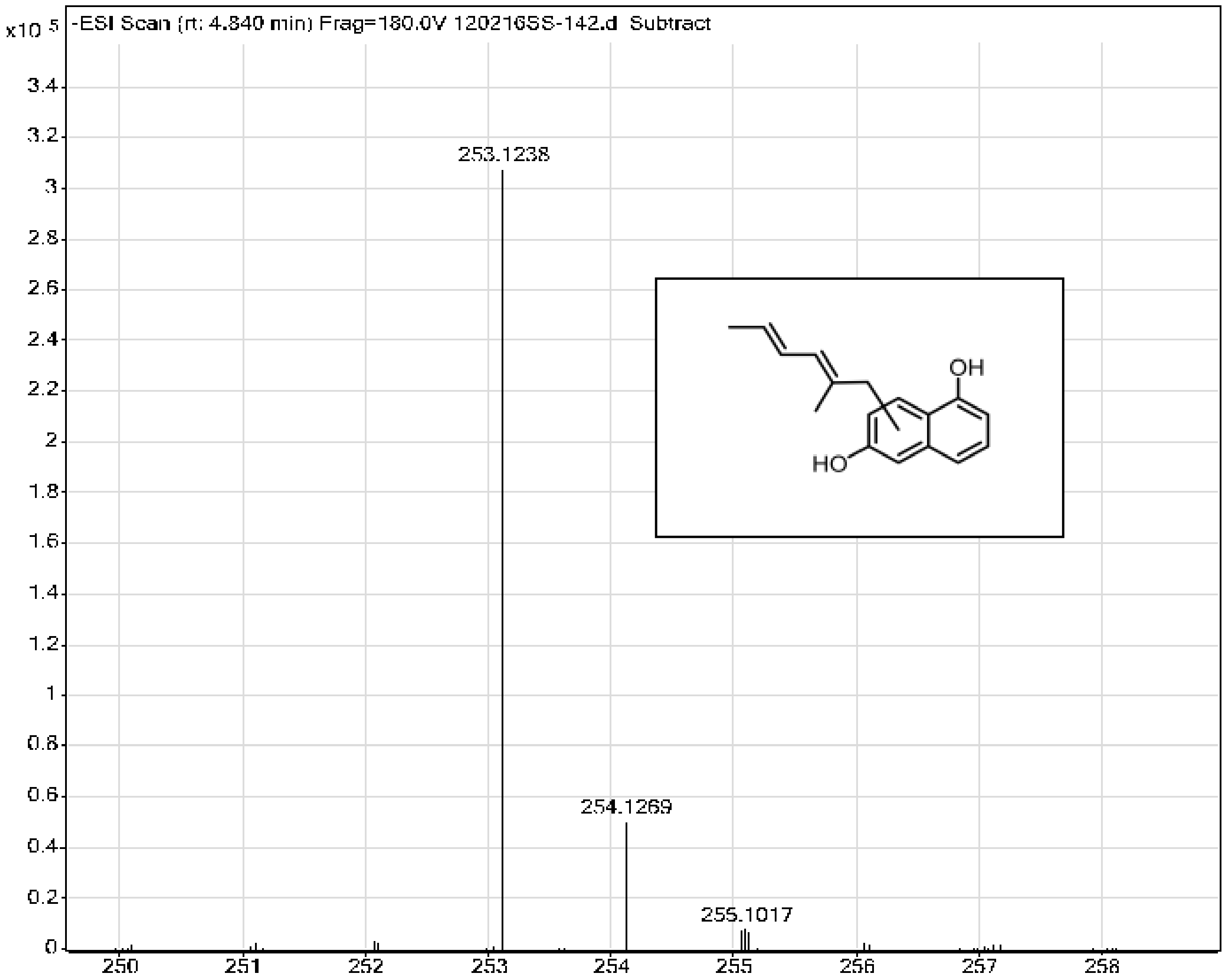

(-)-ESI-HRMS Spectrum of 1,6-DHN-**18**

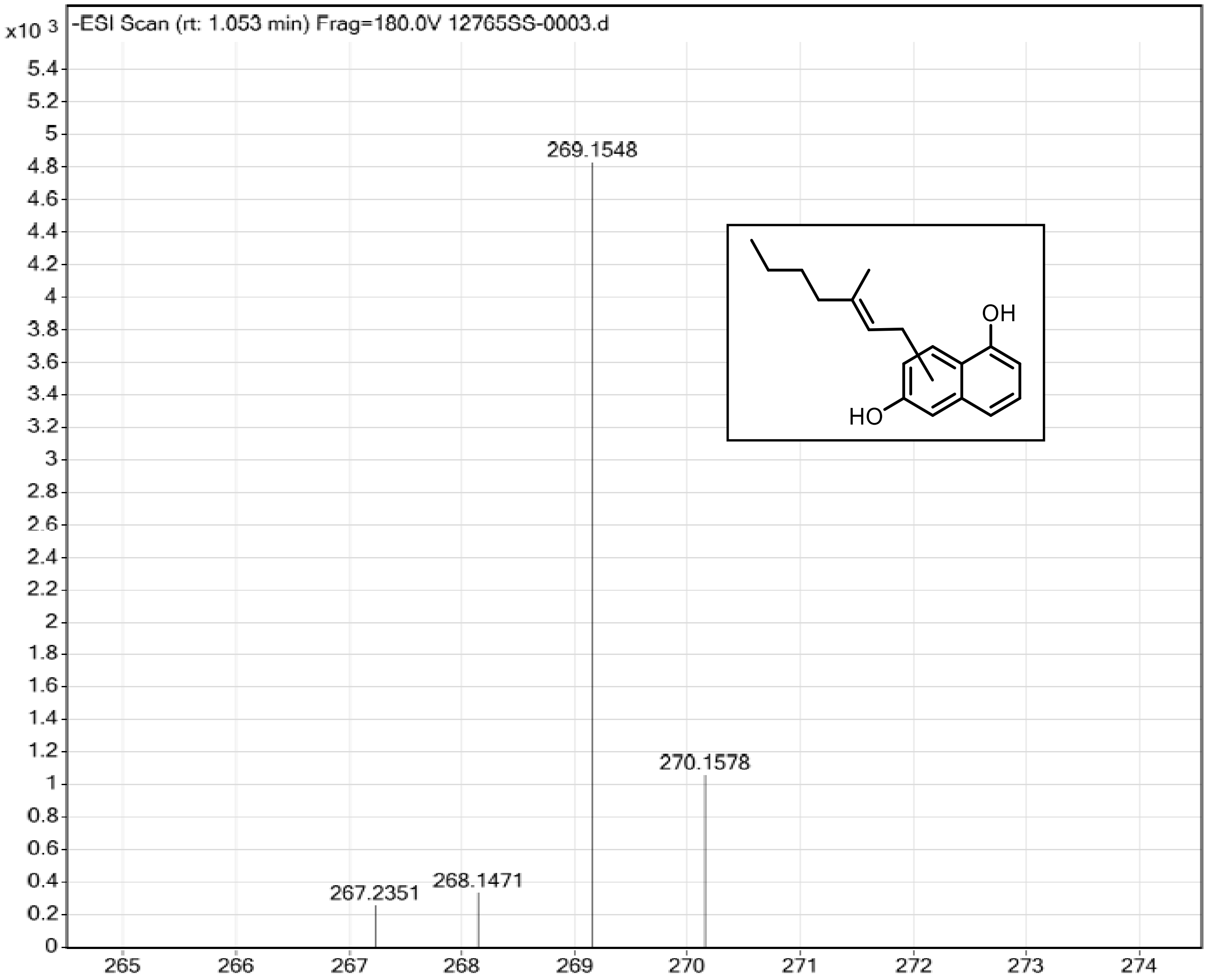

(-)-ESI-HRMS Spectrum of 1,6-DHN-**21**

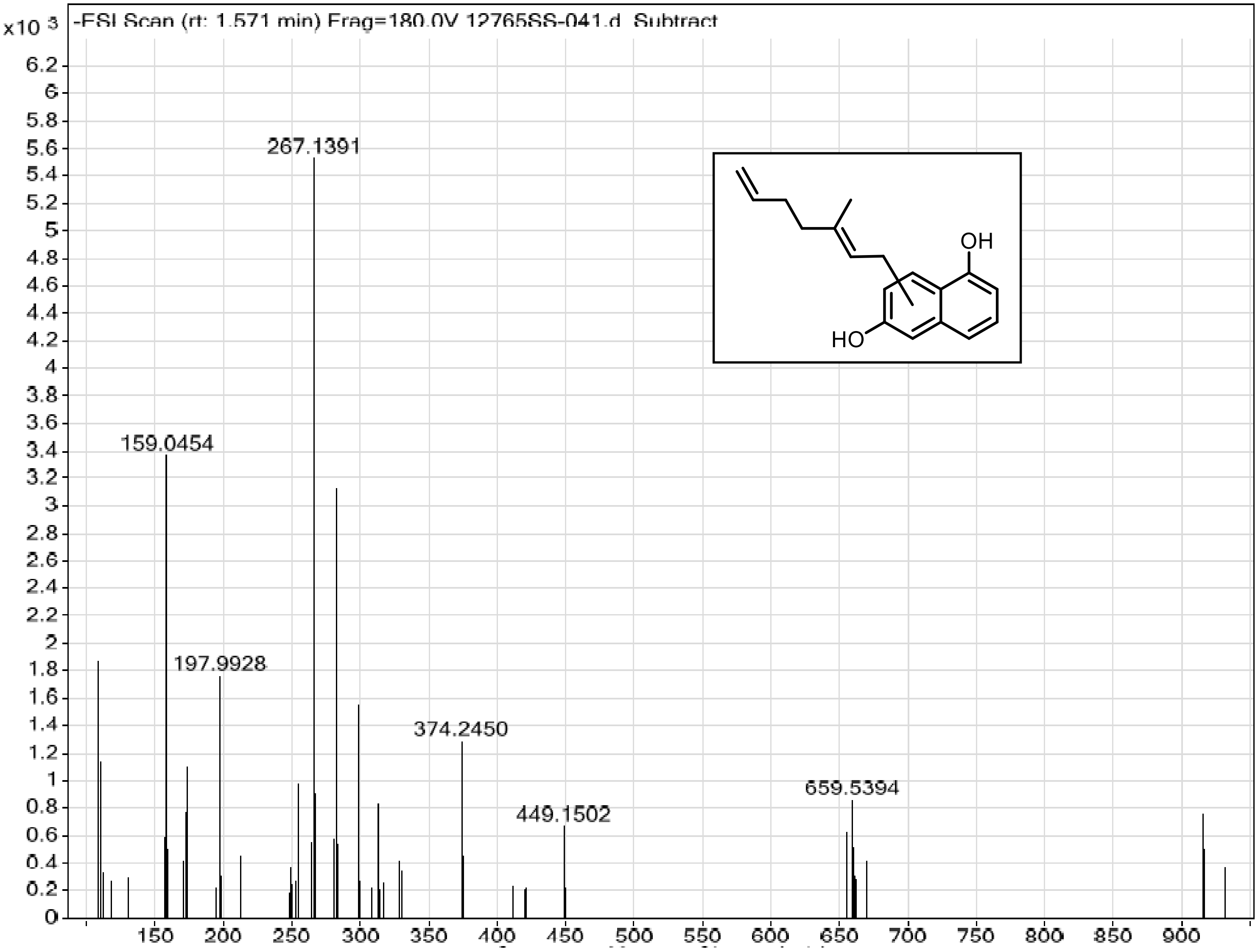

(-)-ESI-HRMS Spectrum of 1,6-DHN-**22**

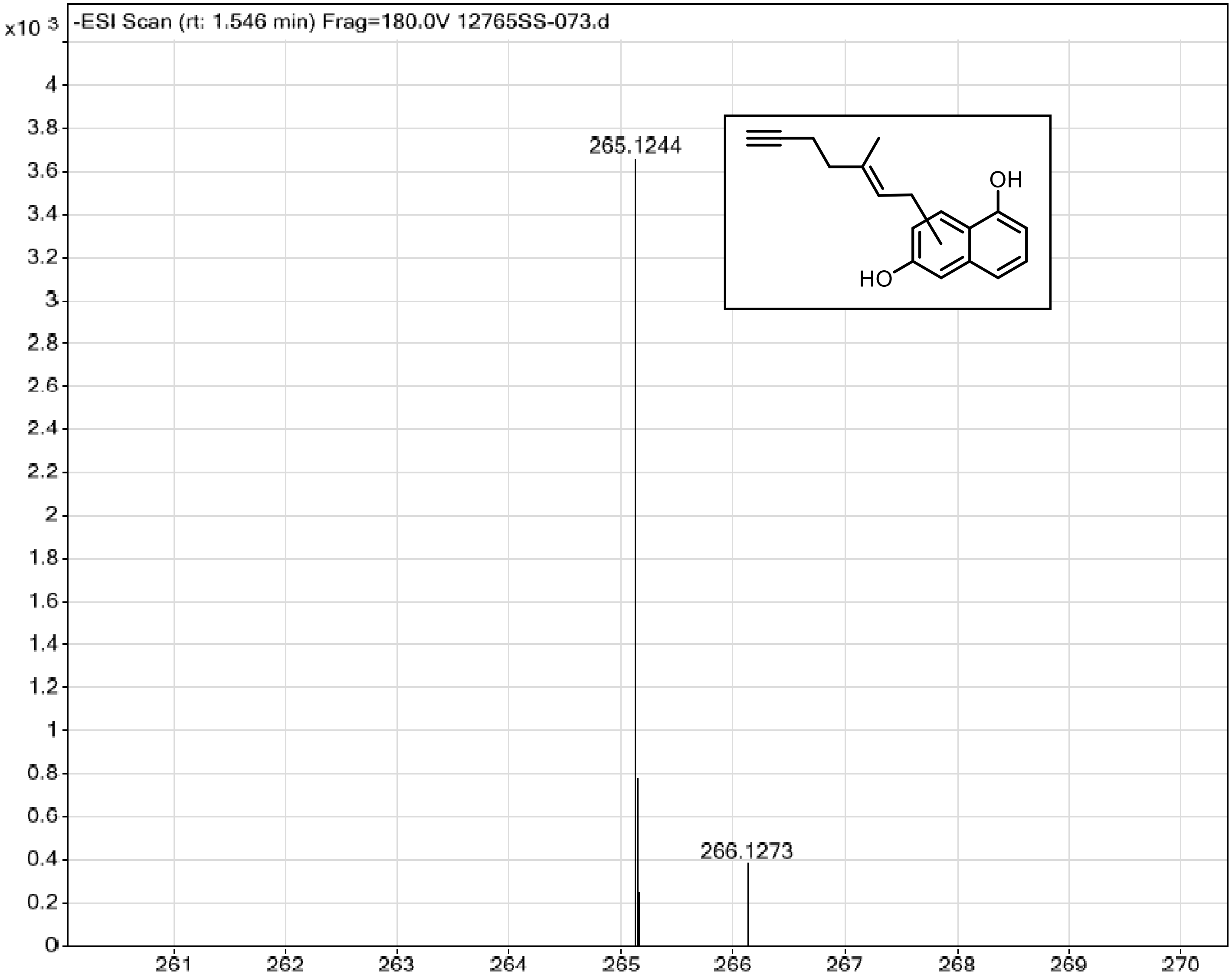

(-)-ESI-HRMS Spectrum of 1,6-DHN-**27**

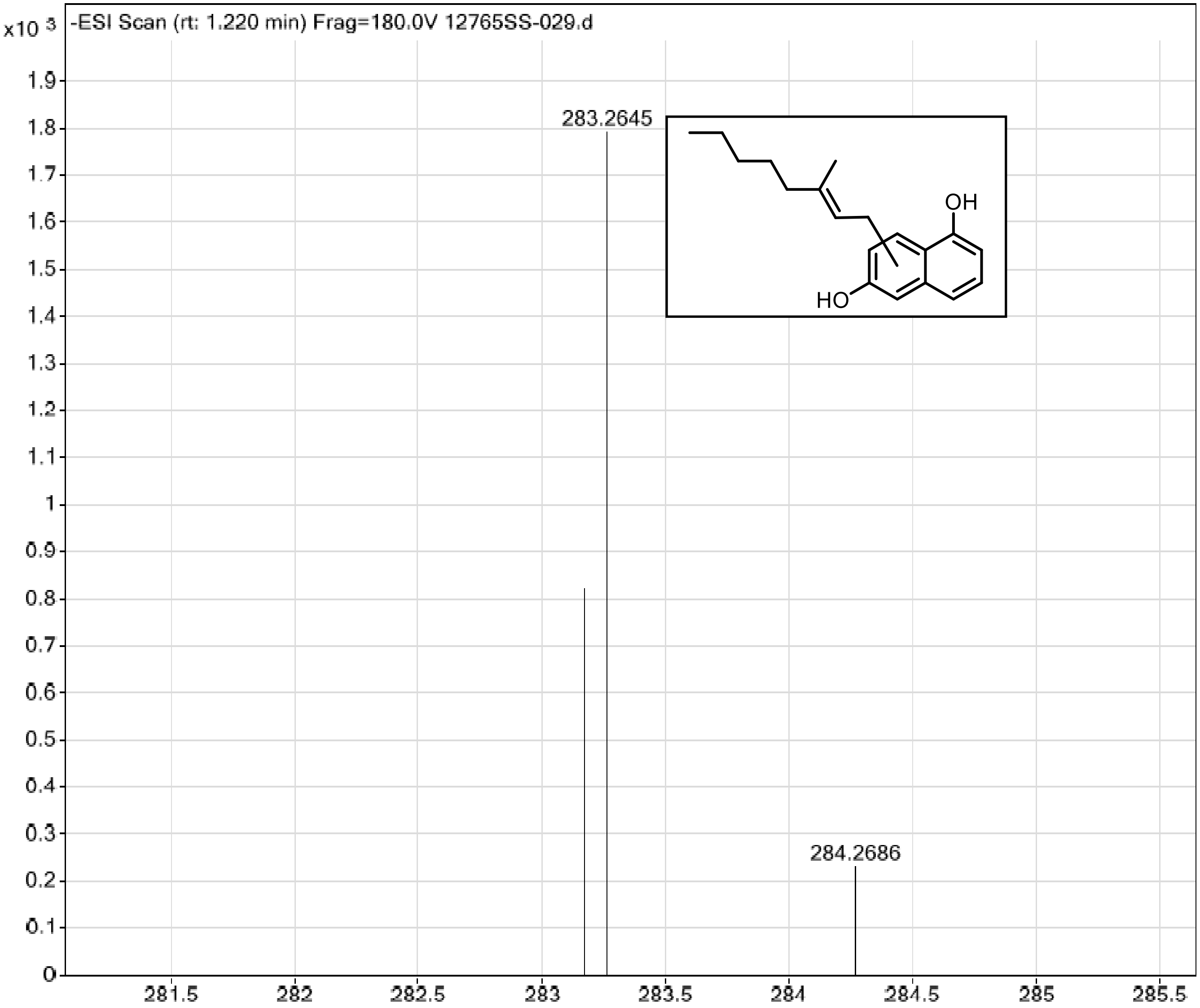

(-)-ESI-HRMS Spectrum of 1,6-DHN-**31**

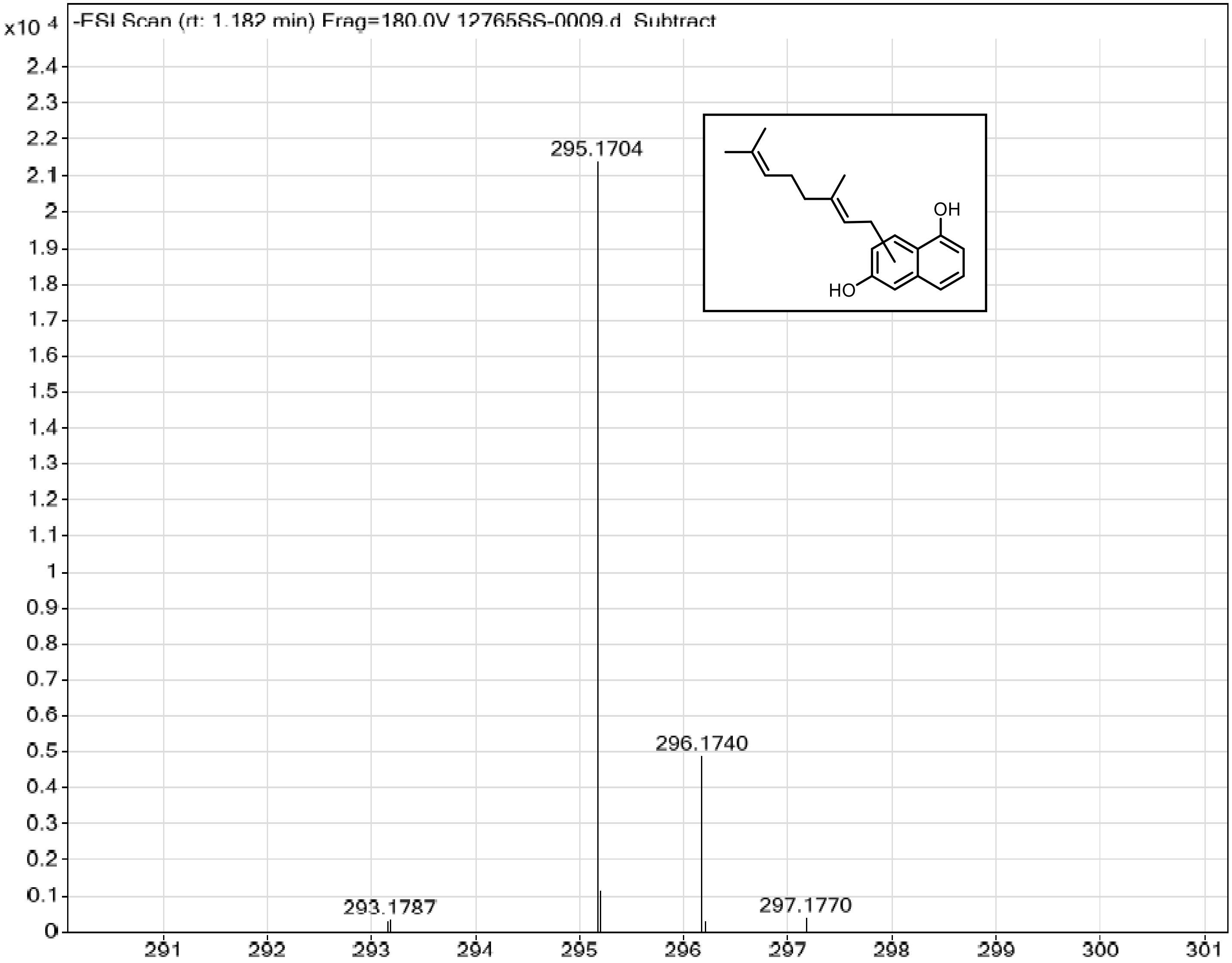

(-)-ESI-HRMS Spectrum of 1,6-DHN-**32**

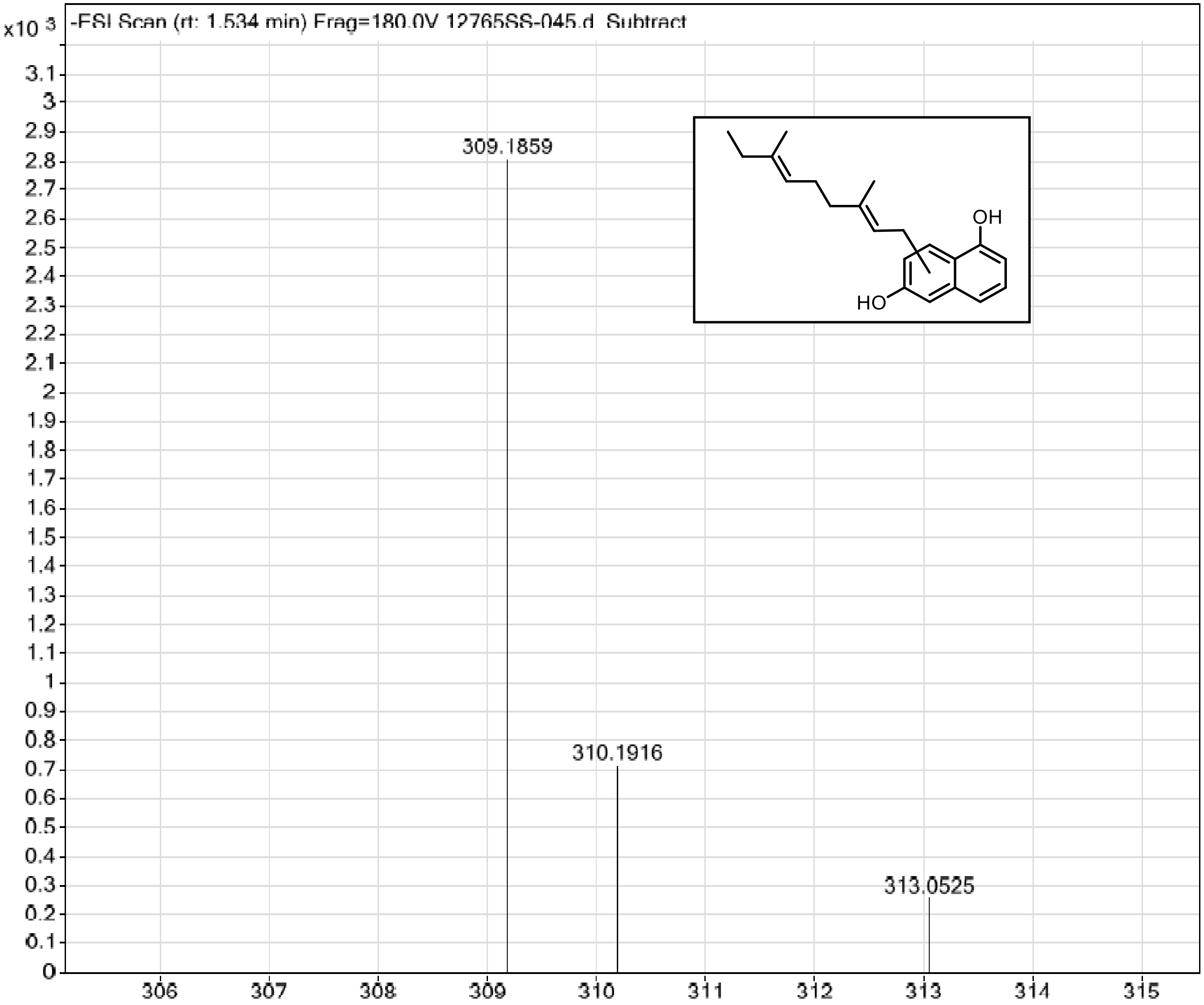

(-)-ESI-HRMS Spectrum of 1,6-DHN-**35**

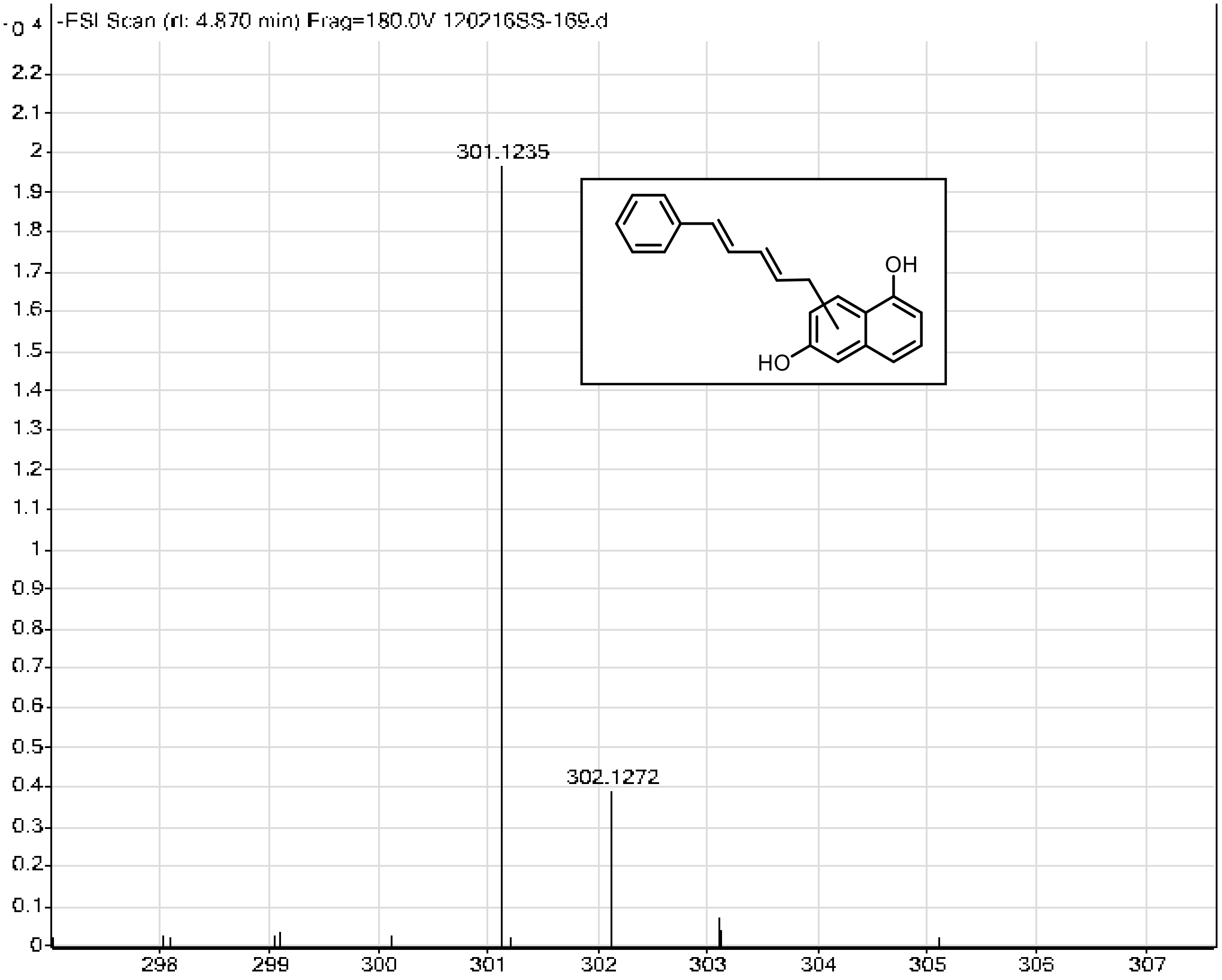

(-)-ESI-HRMS Spectrum of 1,6-DHN-**37**

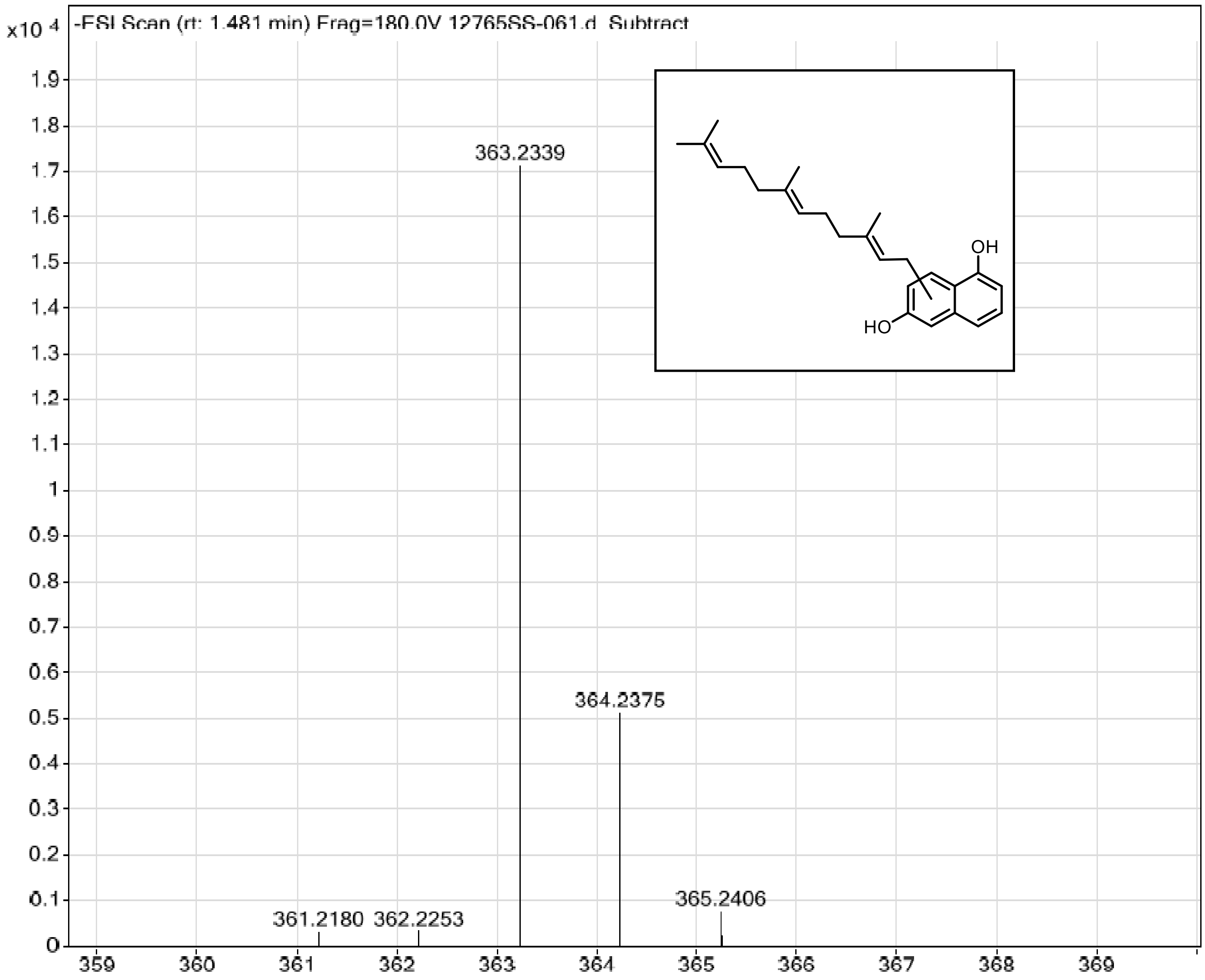

(-)-ESI-HRMS Spectrum of 1,6-DHN-**42**

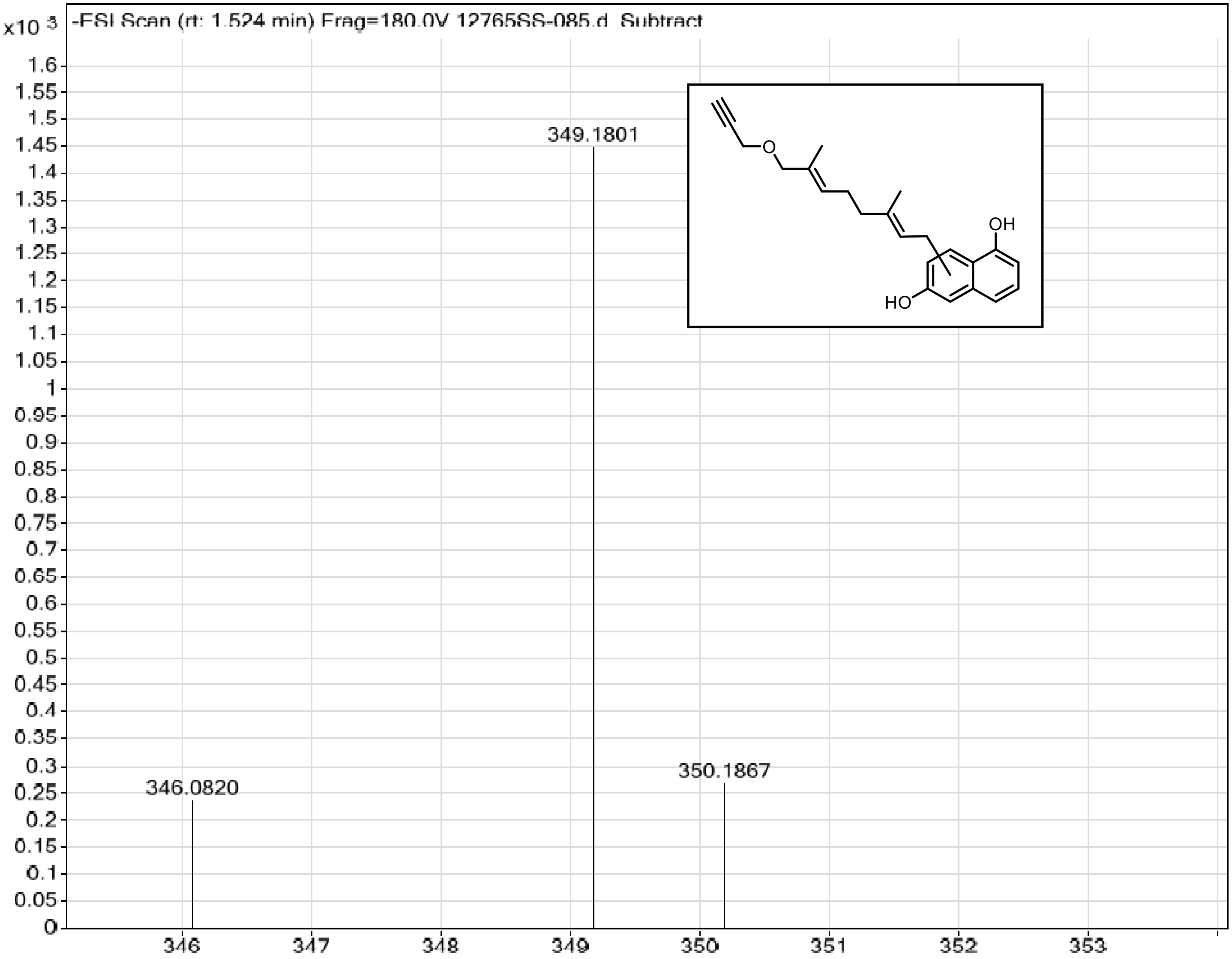

(-)-ESI-HRMS Spectrum of 1,6-DHN-**43**

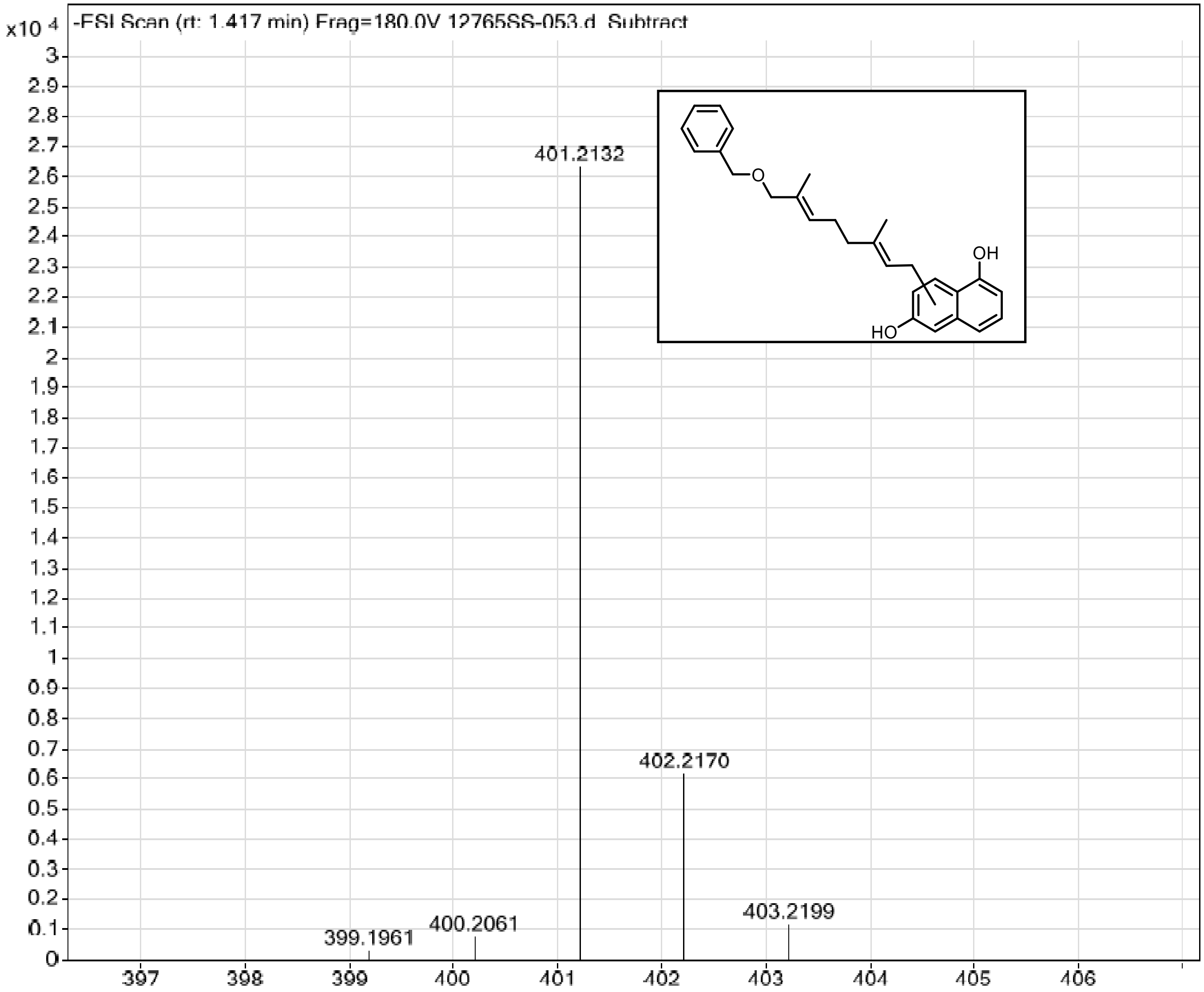

(-)-ESI-HRMS Spectrum of 1,6-DHN-**44**

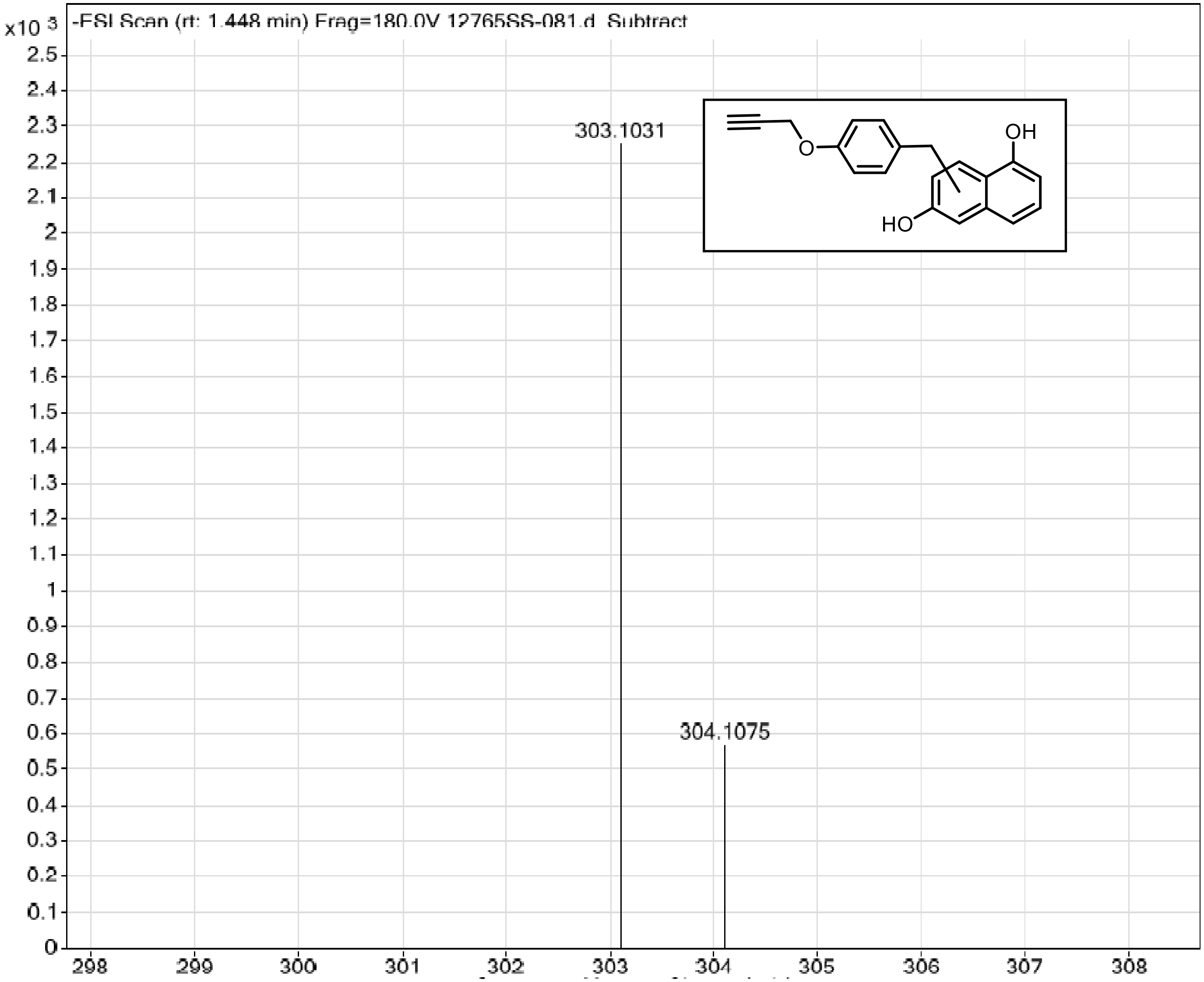

(-)-ESI-HRMS Spectrum of 1,6-DHN-**59**

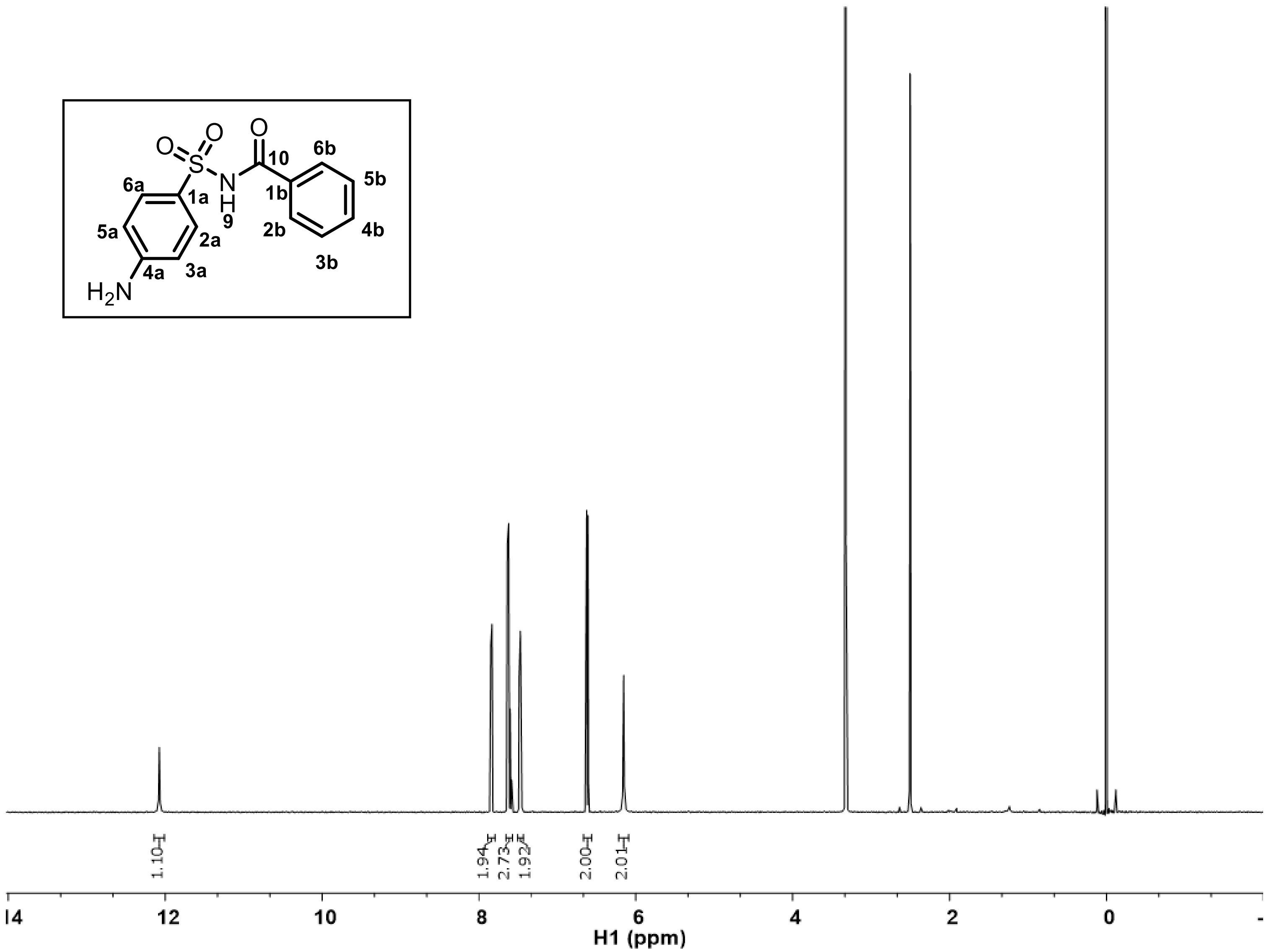

^{1}H NMR Spectrum of Sulfabenzamide (500 MHz, DMSO-d6)

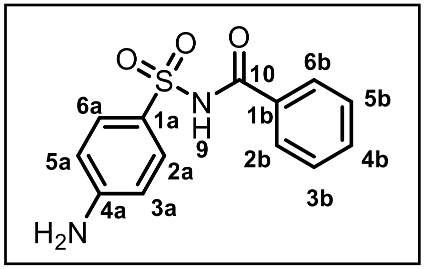

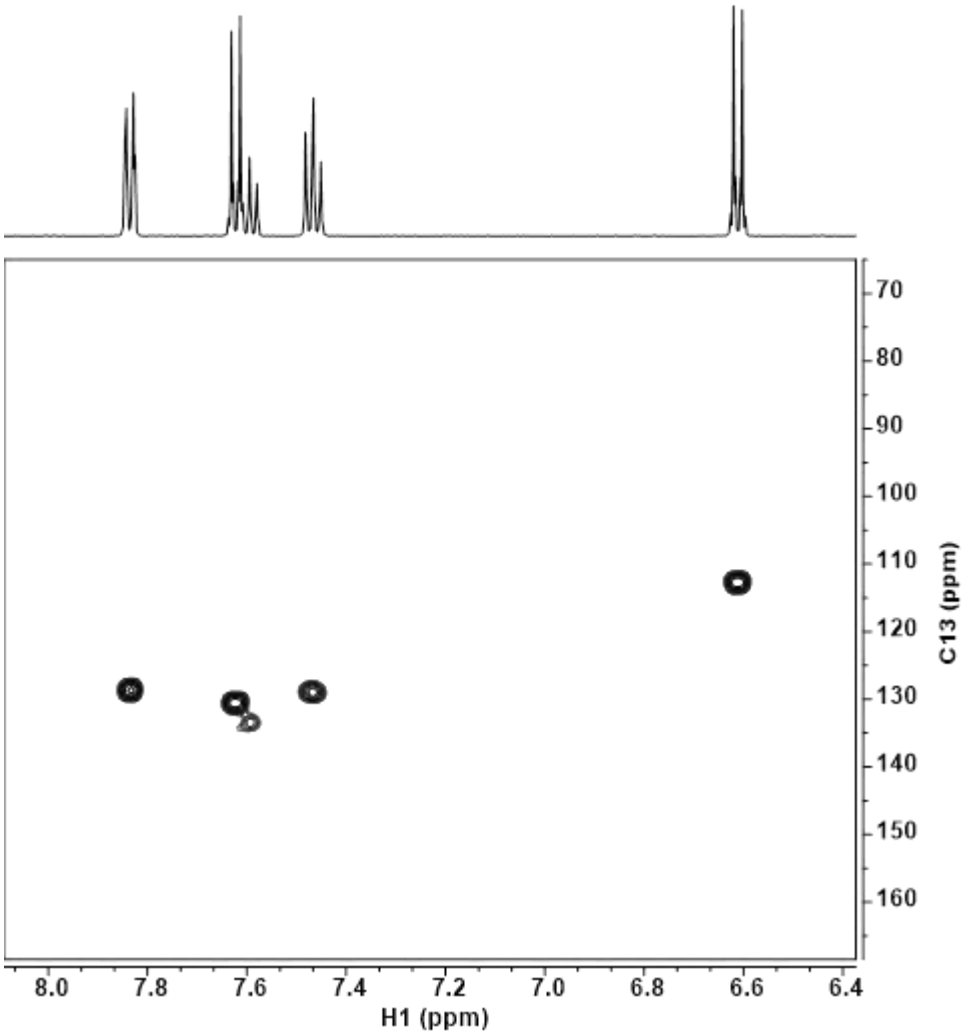

2D ^{1}H-^{13}C HSQC NMR Spectrum of Sulfabenzamide (500 MHz, DMSO-d6)

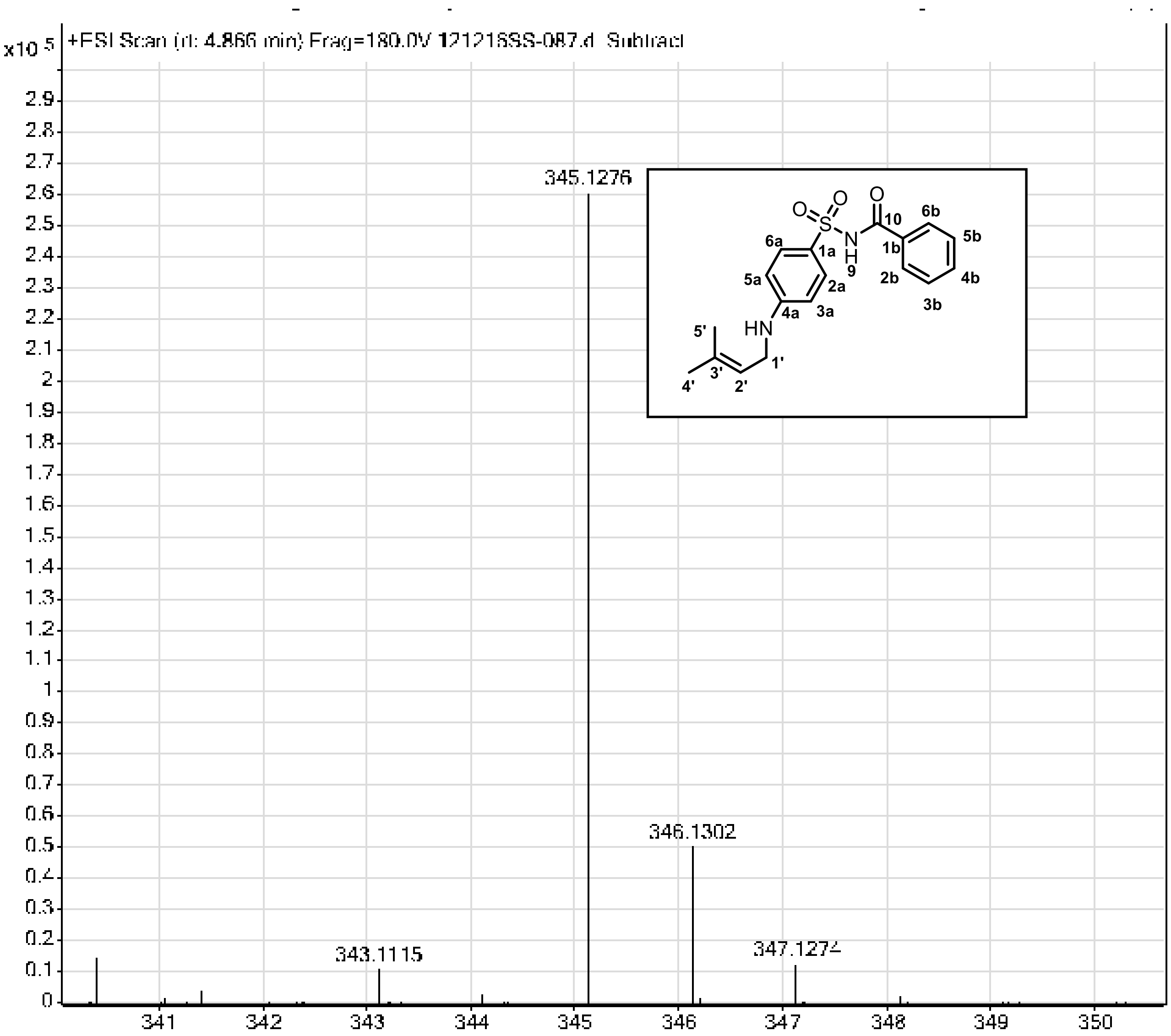

(+)-ESI-HRMS Spectrum of Sulfabenzamide-**2** (**67**)

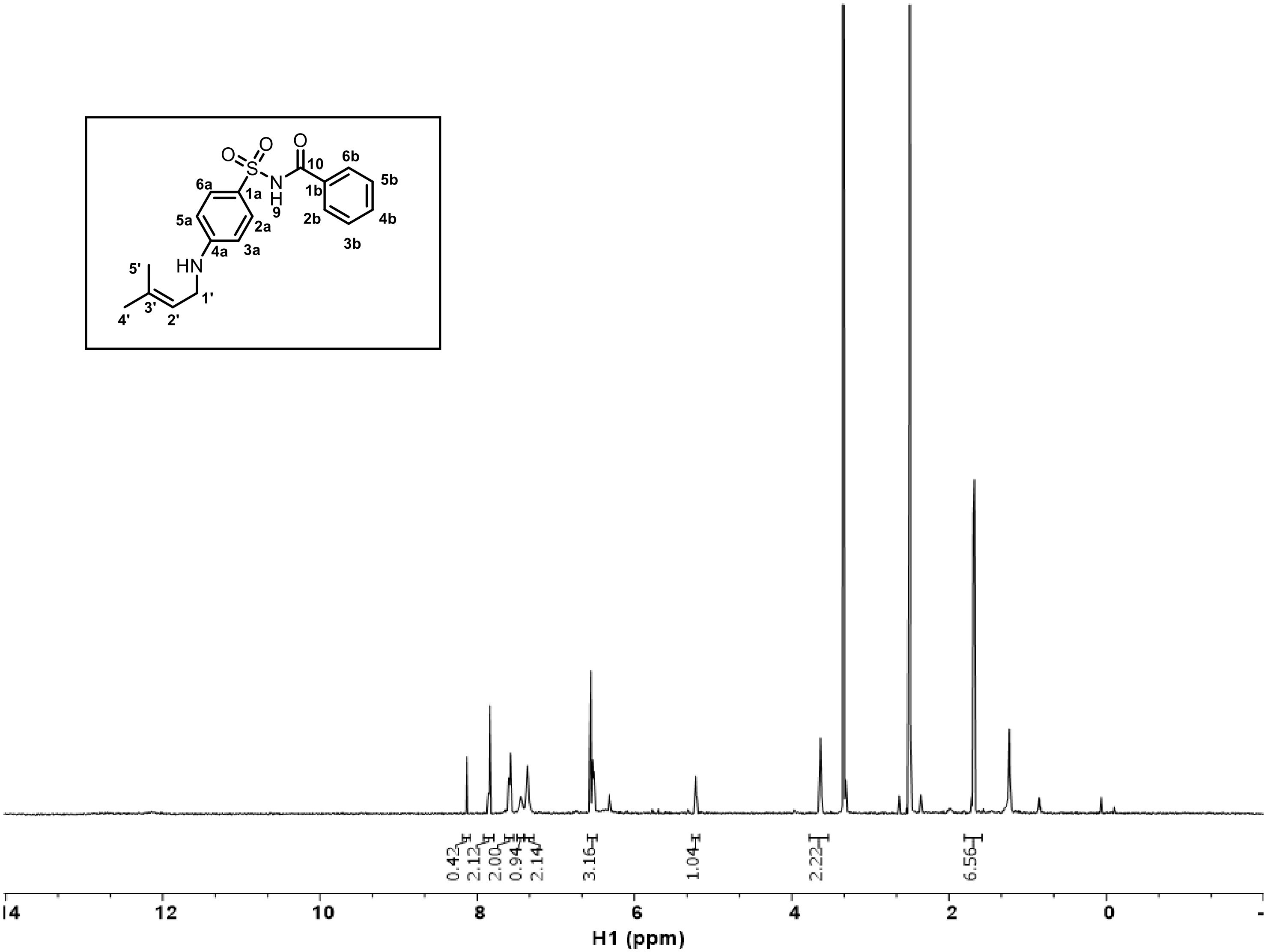

^{1}H NMR Spectrum of Sulfabenzamide-**2** (**67**) (500 MHz, DMSO-d6)

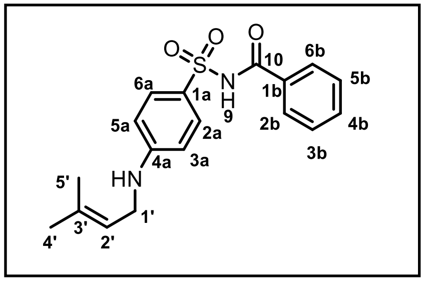

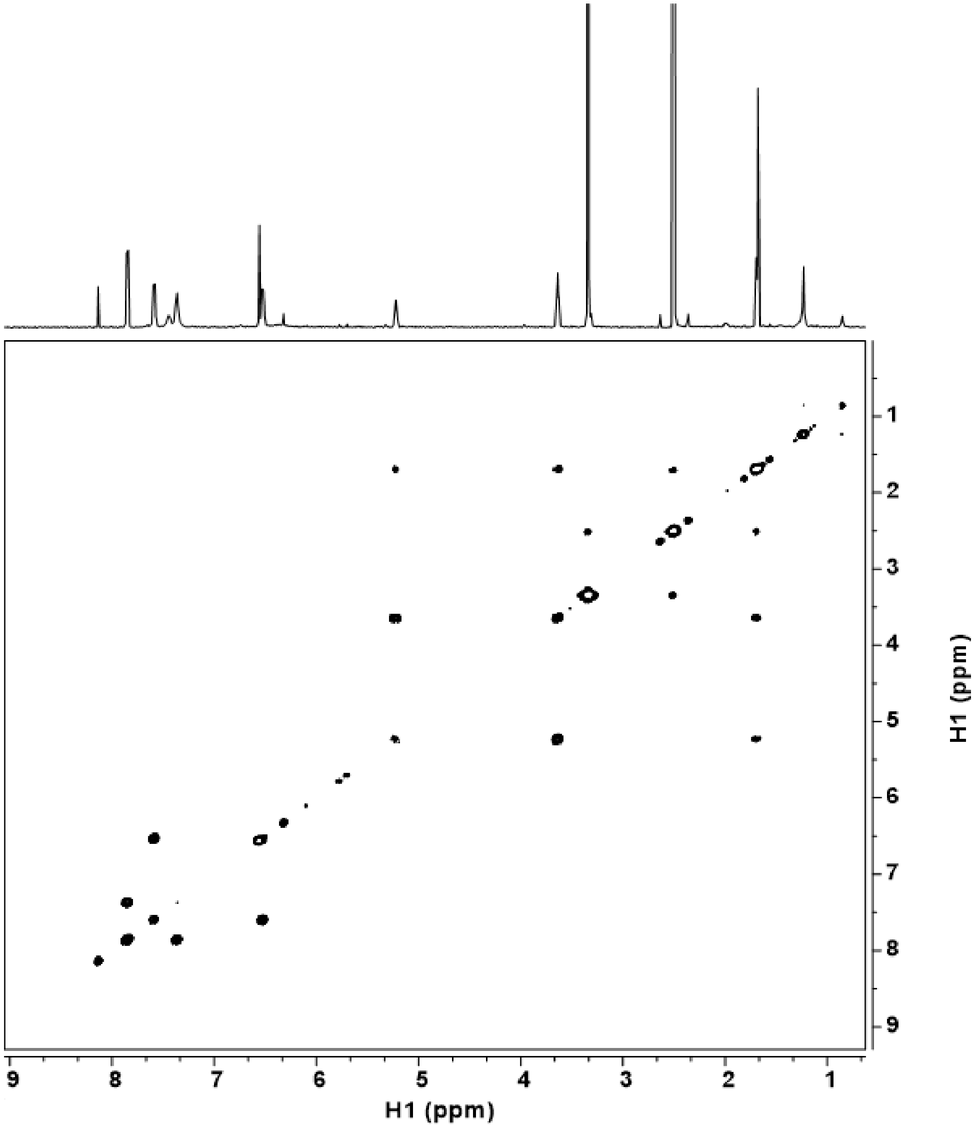

2D ^{1}H-^{1}H COSY NMR Spectrum of Sulfabenzamide-**2** (**67**) (500 MHz, DMSO-d6)

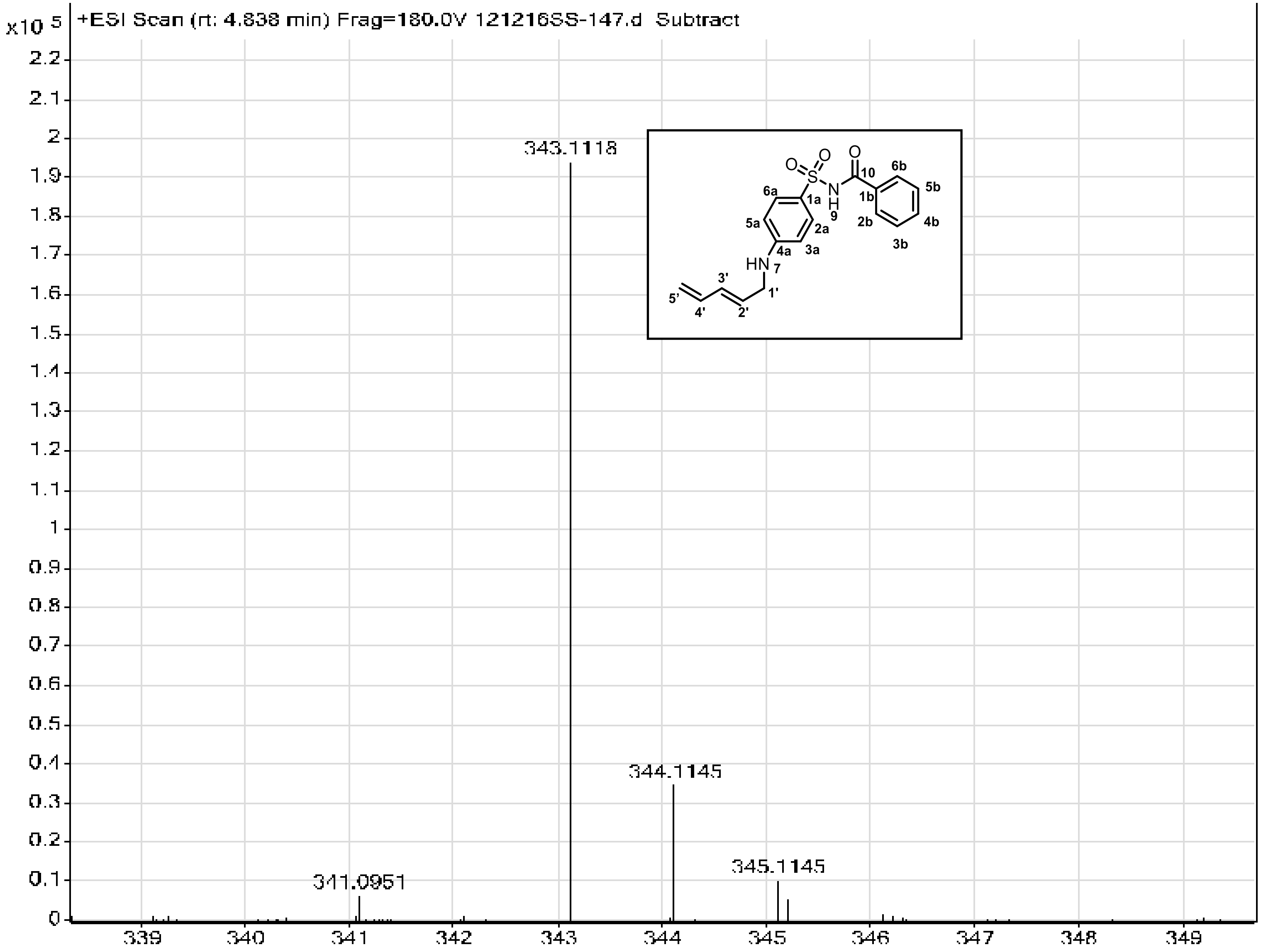

(+)-ESI-HRMS Spectrum of Sulfabenzamide-**6** (**68**)

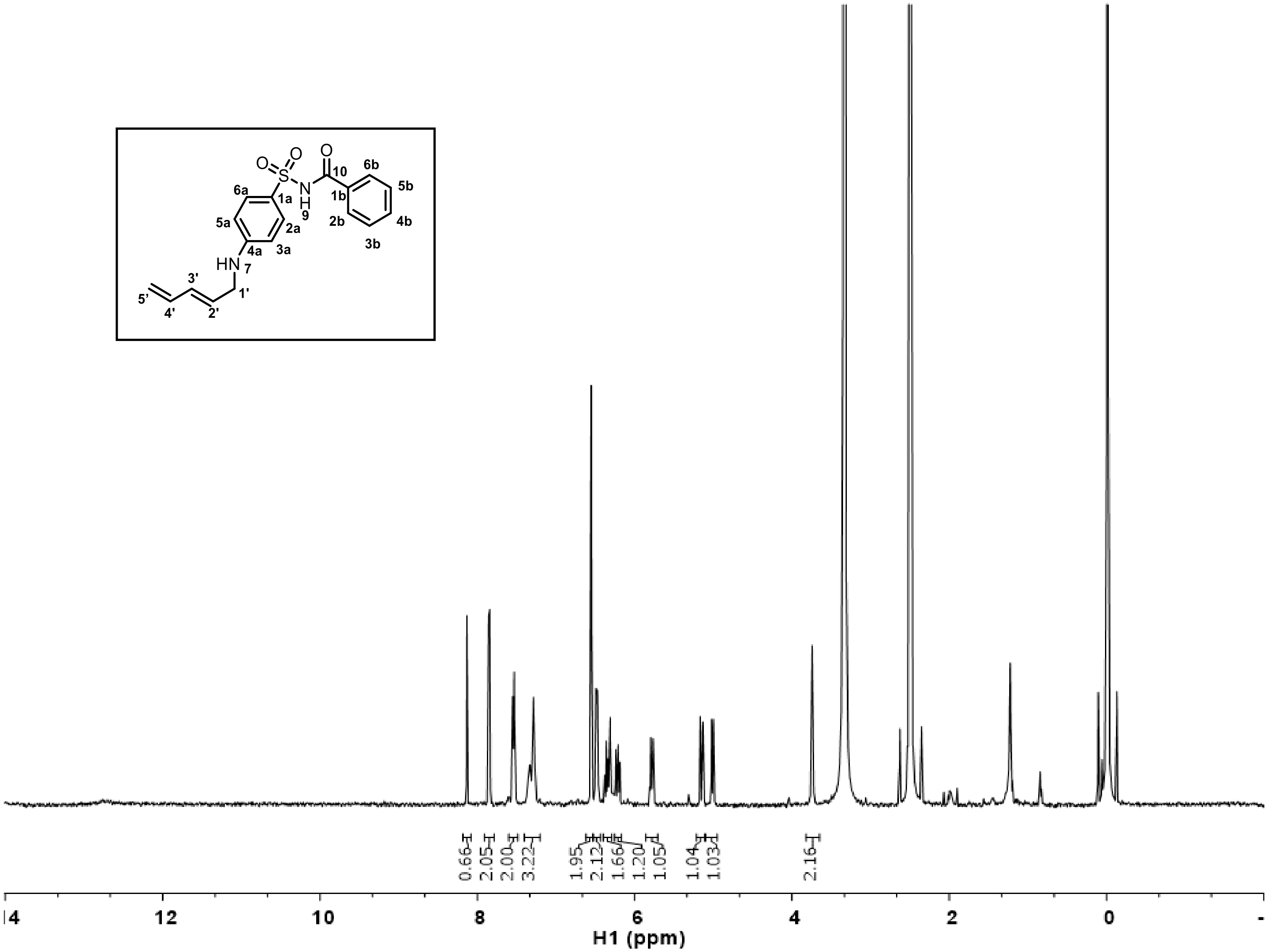

^{1}H NMR Spectrum of Sulfabenzamide-**6** (**68**) (500 MHz, DMSO-d6)

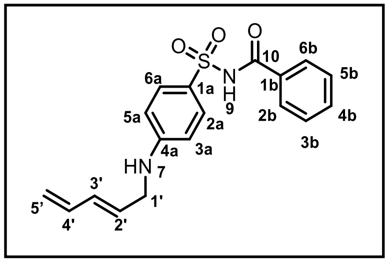

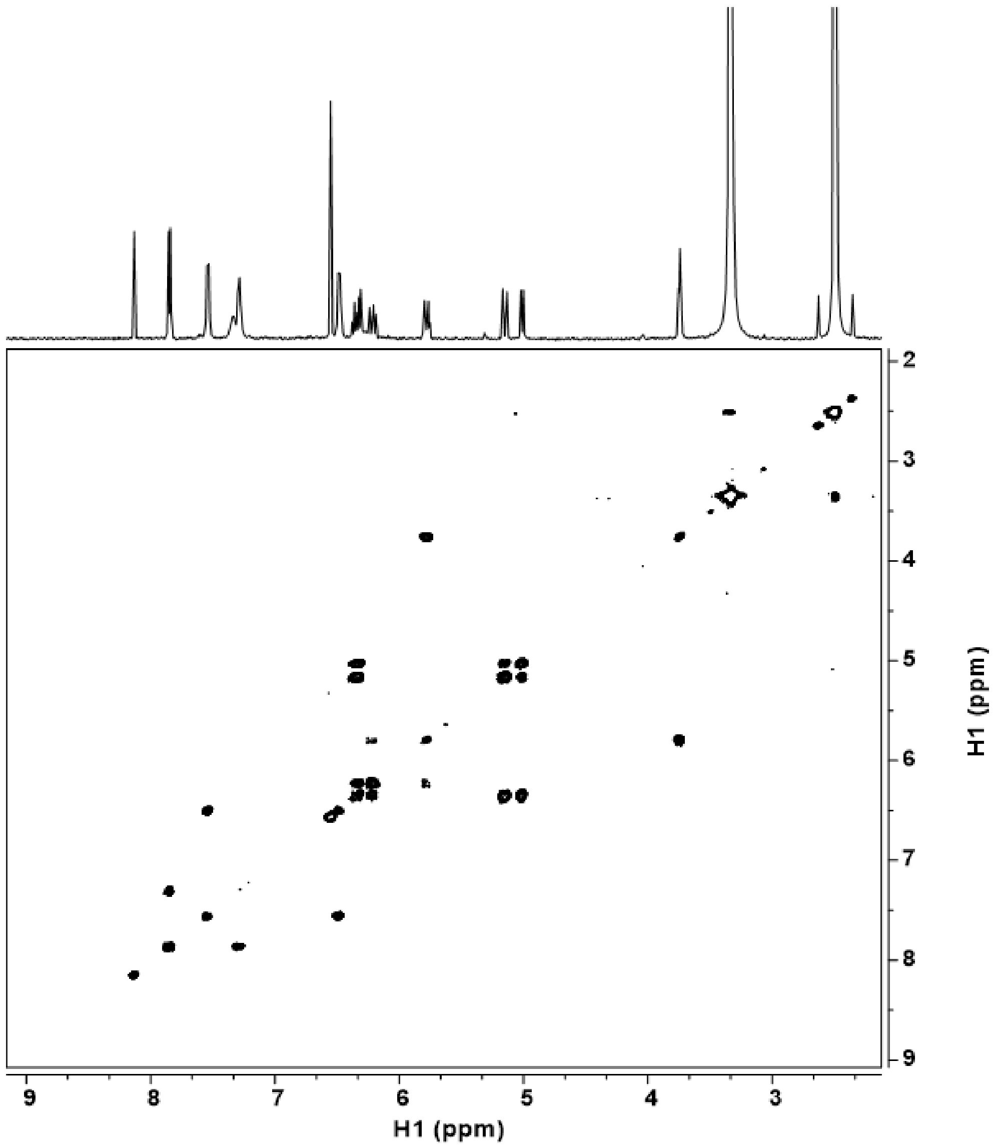

2D ^{1}H-^{1}H COSY NMR Spectrum of Sulfabenzamide-**6** (**68**) (500 MHz, DMSO-d6)

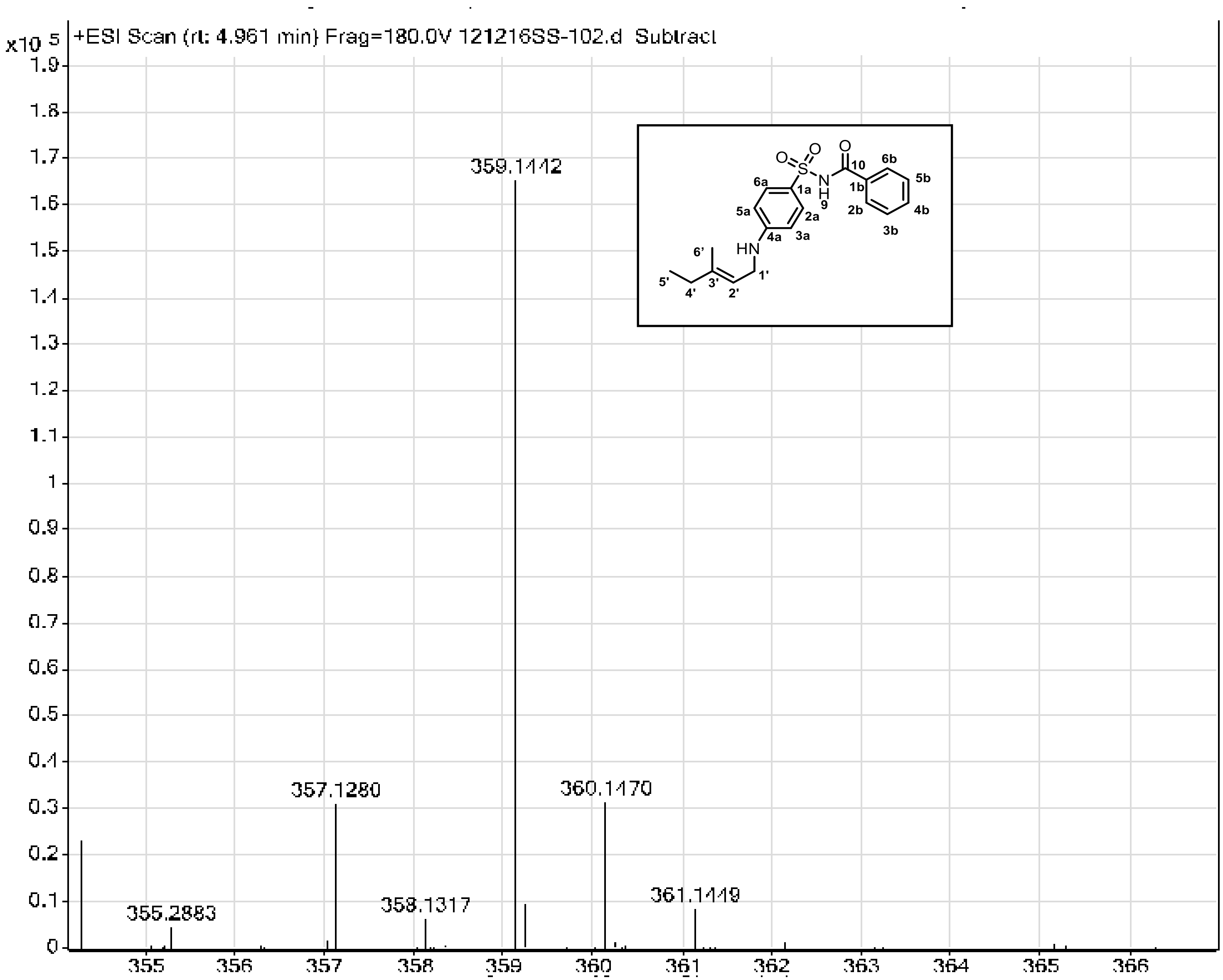

(+)-ESI-HRMS Spectrum of Sulfabenzamide-**7** (**69**)

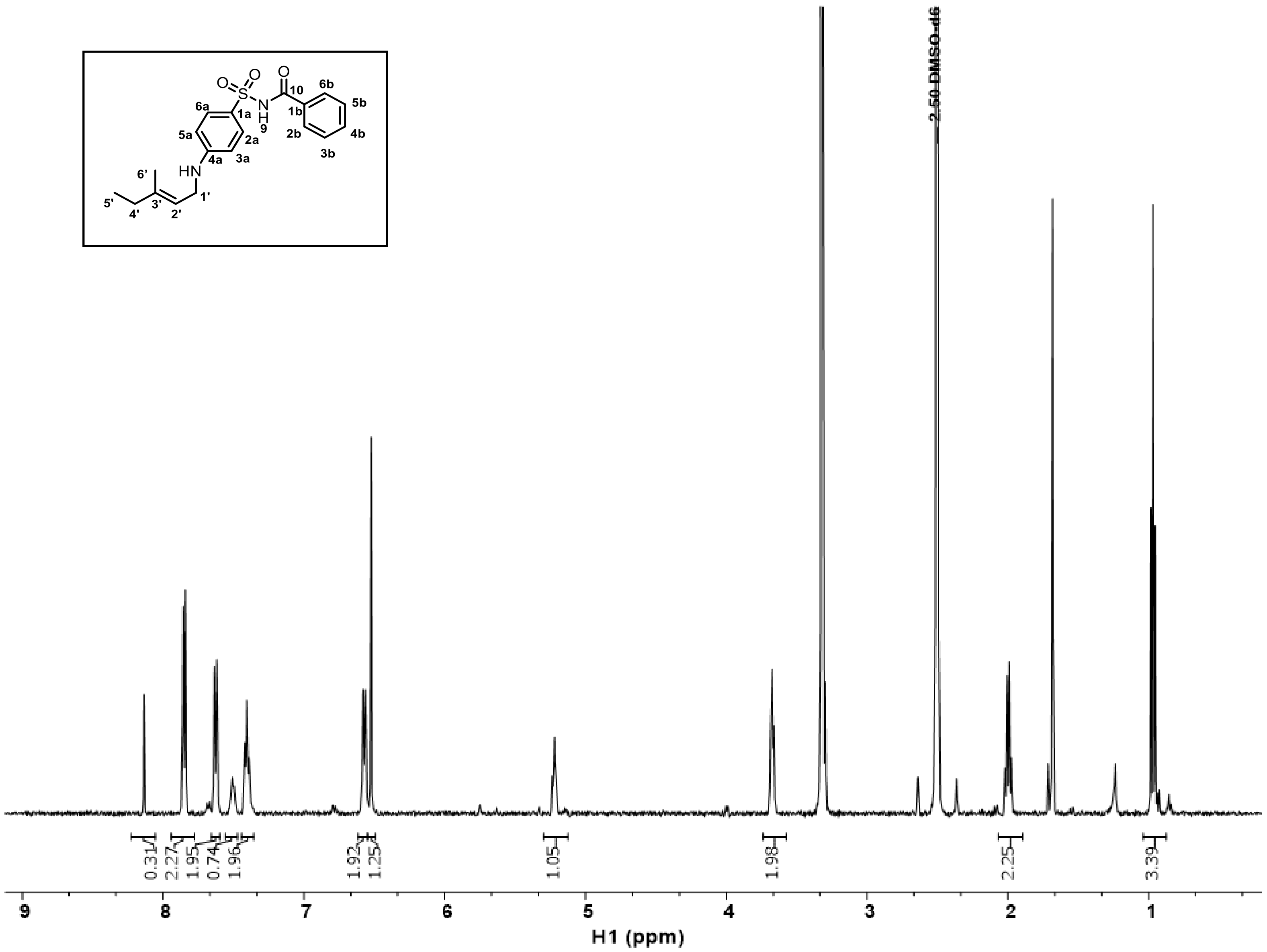

^{1}H NMR Spectrum of Sulfabenzamide-**7** (**69**) (500 MHz, DMSO-d6)

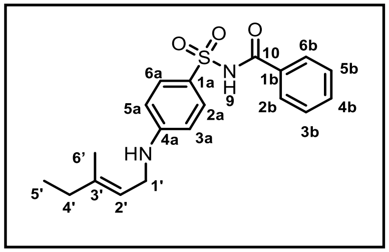

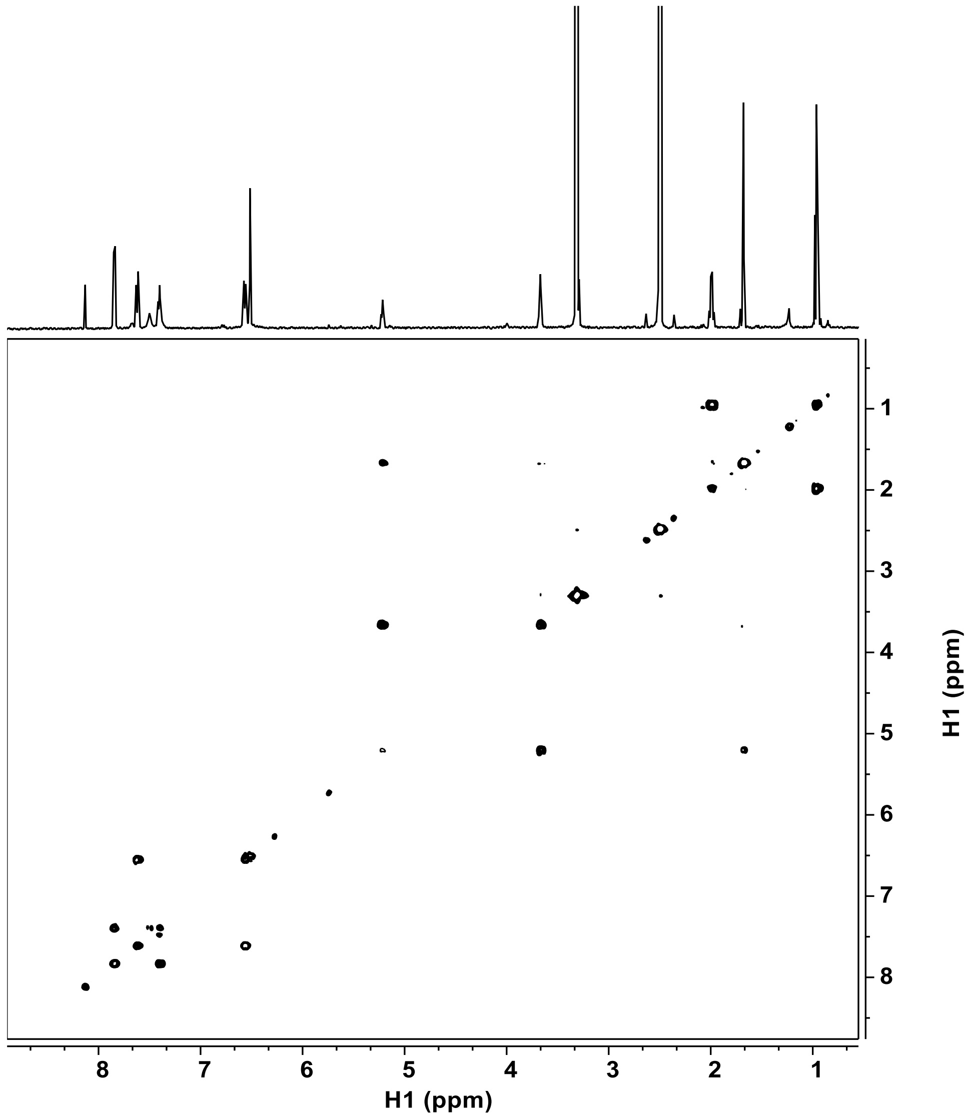

2D ^{1}H-^{1}H COSY NMR Spectrum of Sulfabenzamide-**7** (**69**) (500 MHz, DMSO-d6)

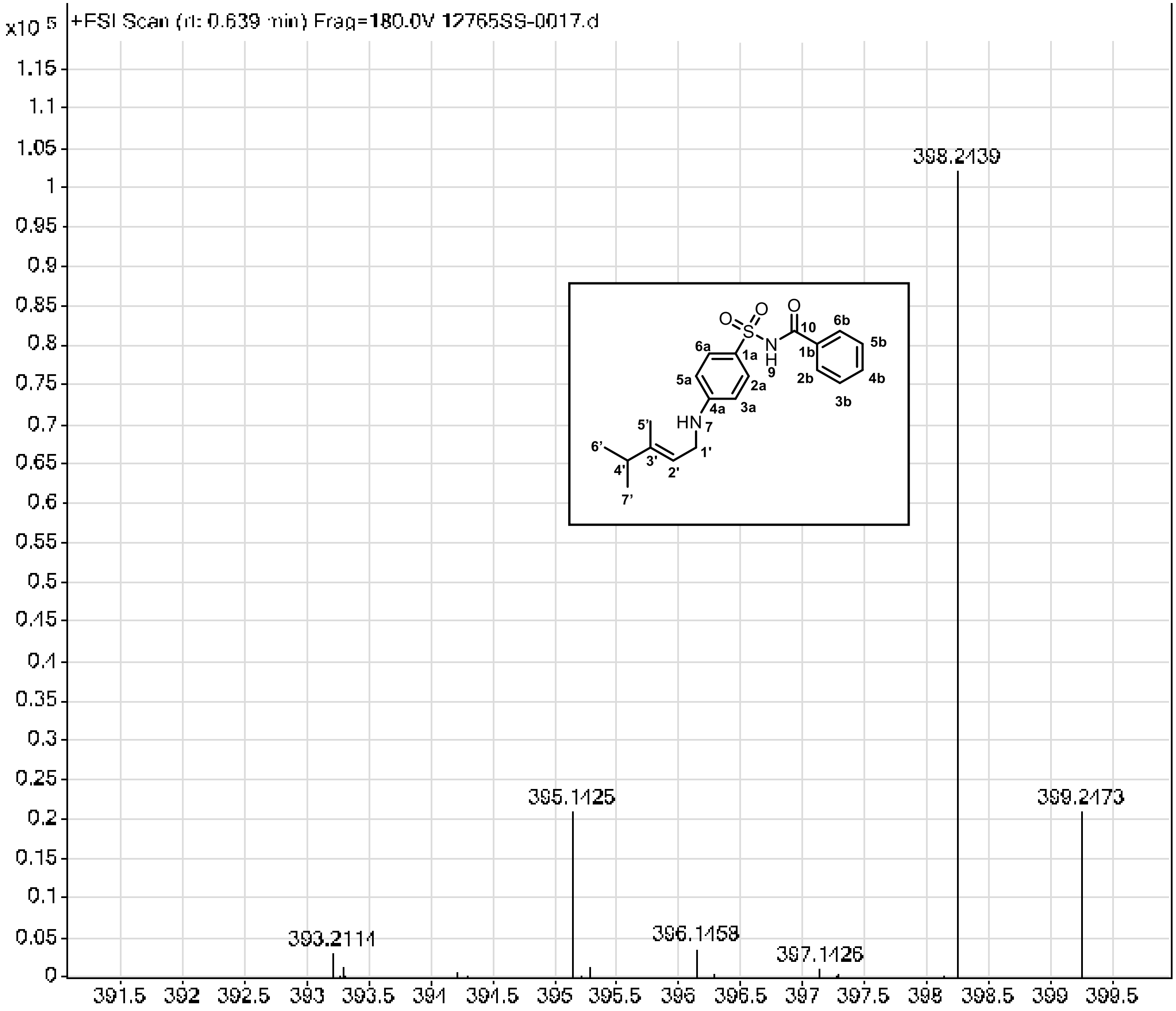

(+)-ESI-HRMS Spectrum of Sulfabenzamide-**10** (**70**)

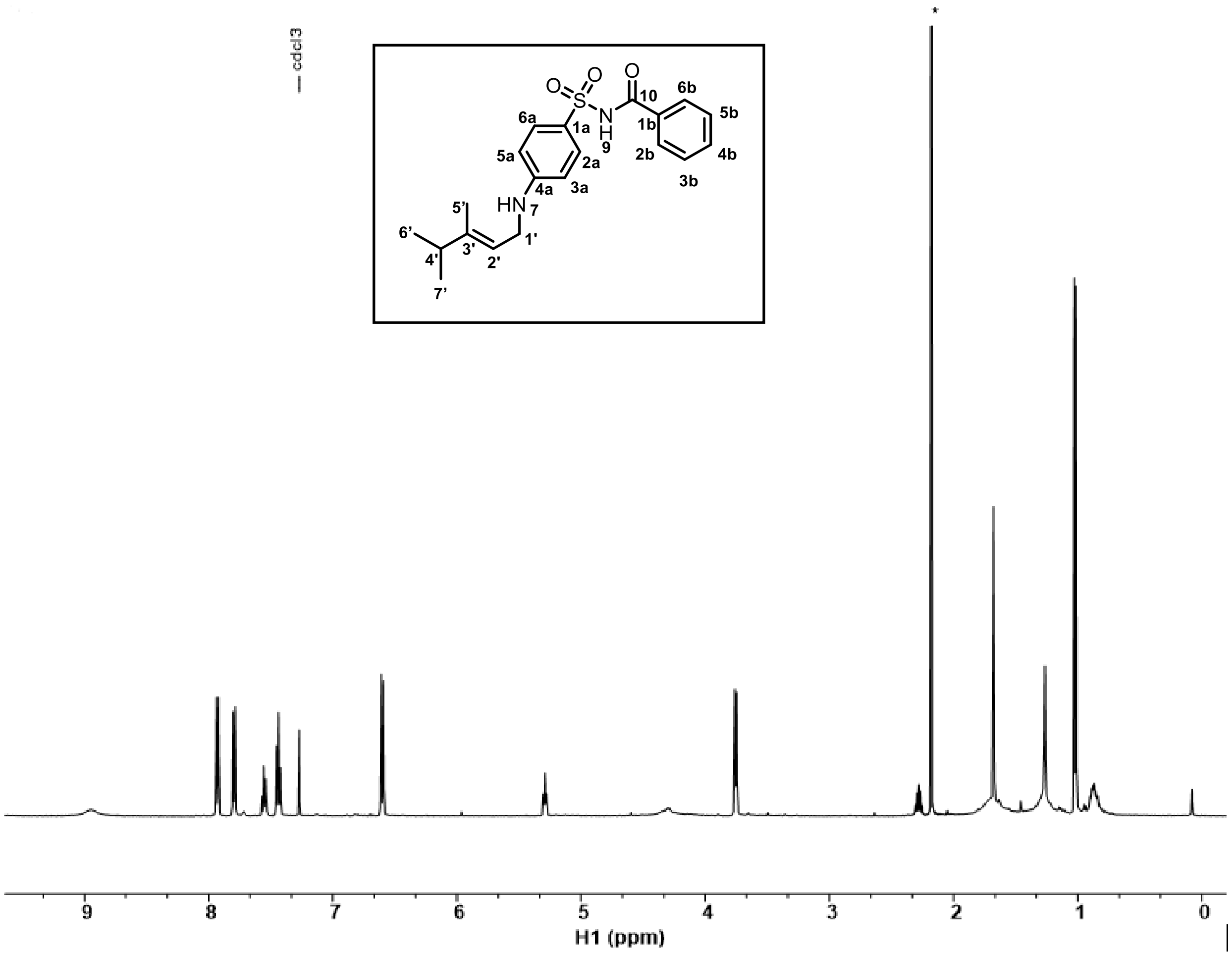

^{1}H NMR Spectrum of Sulfabenzamide-**10** (**70**) (500 MHz, $CDCl_3$) *Denotes an impurity.

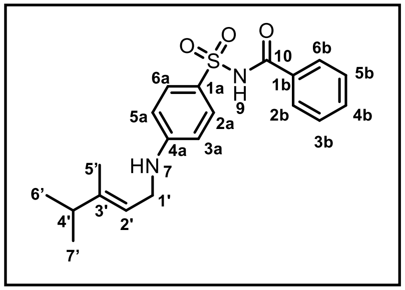

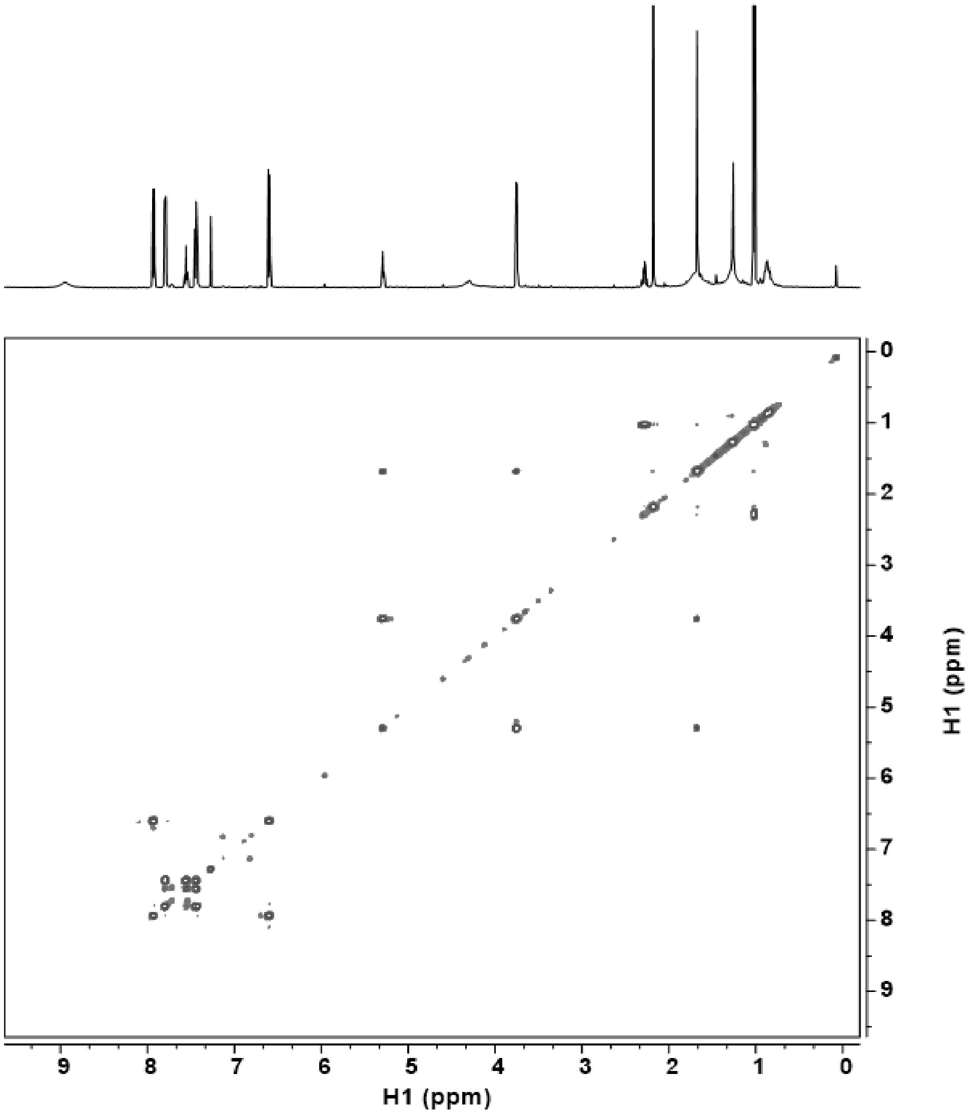

2D ^{1}H-^{1}H COSY NMR Spectrum of Sulfabenzamide-**10** (**70**) (500 MHz, $CDCl_3$)

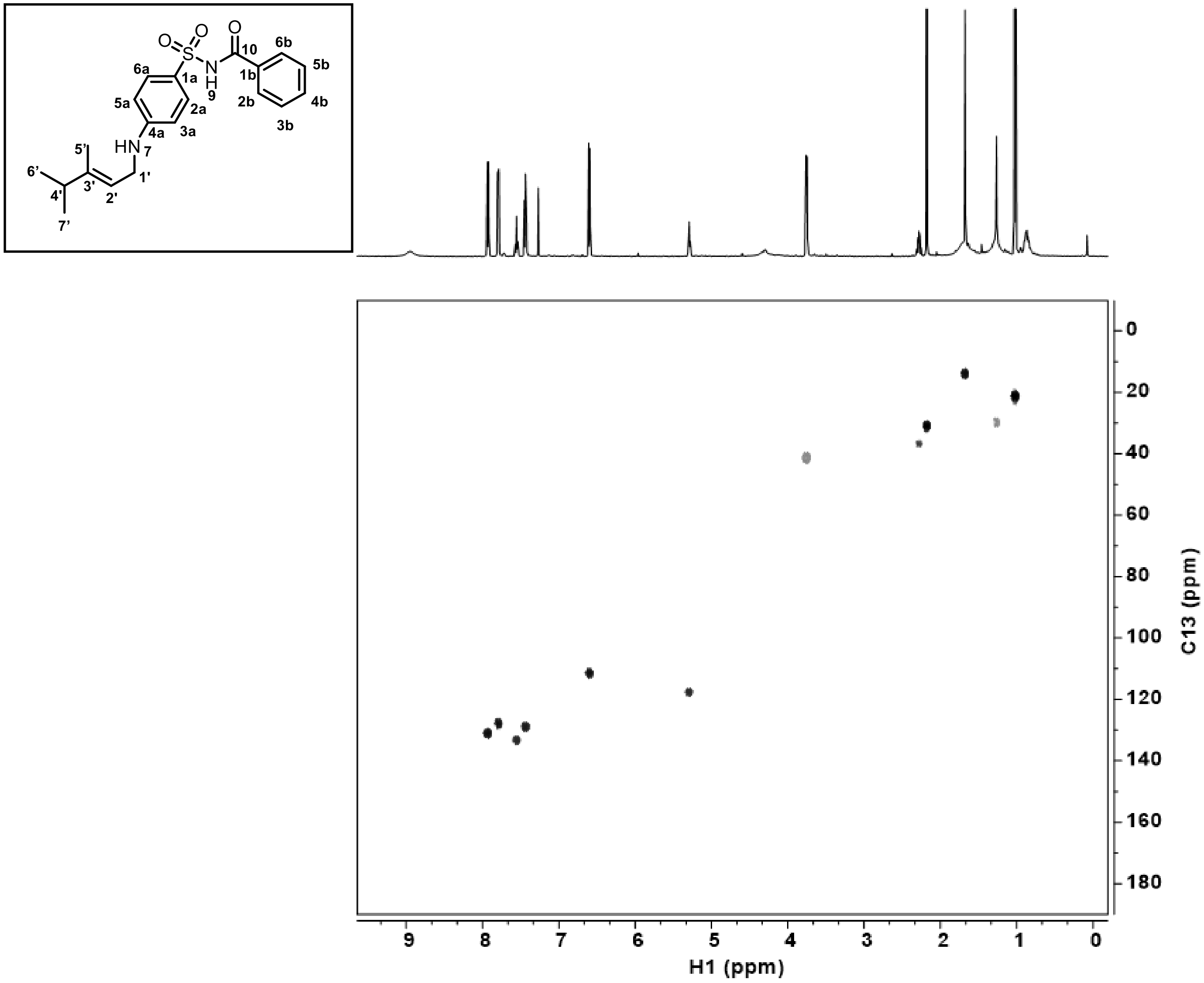

2D ^{1}H-^{13}C HSQC NMR Spectrum of Sulfabenzamide-**10** (**70**) (500 MHz, $CDCl_3$)

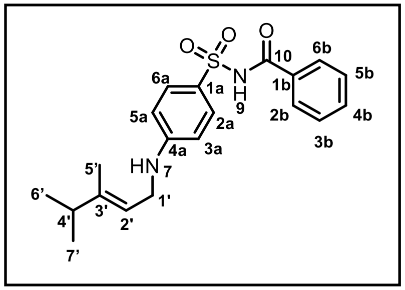

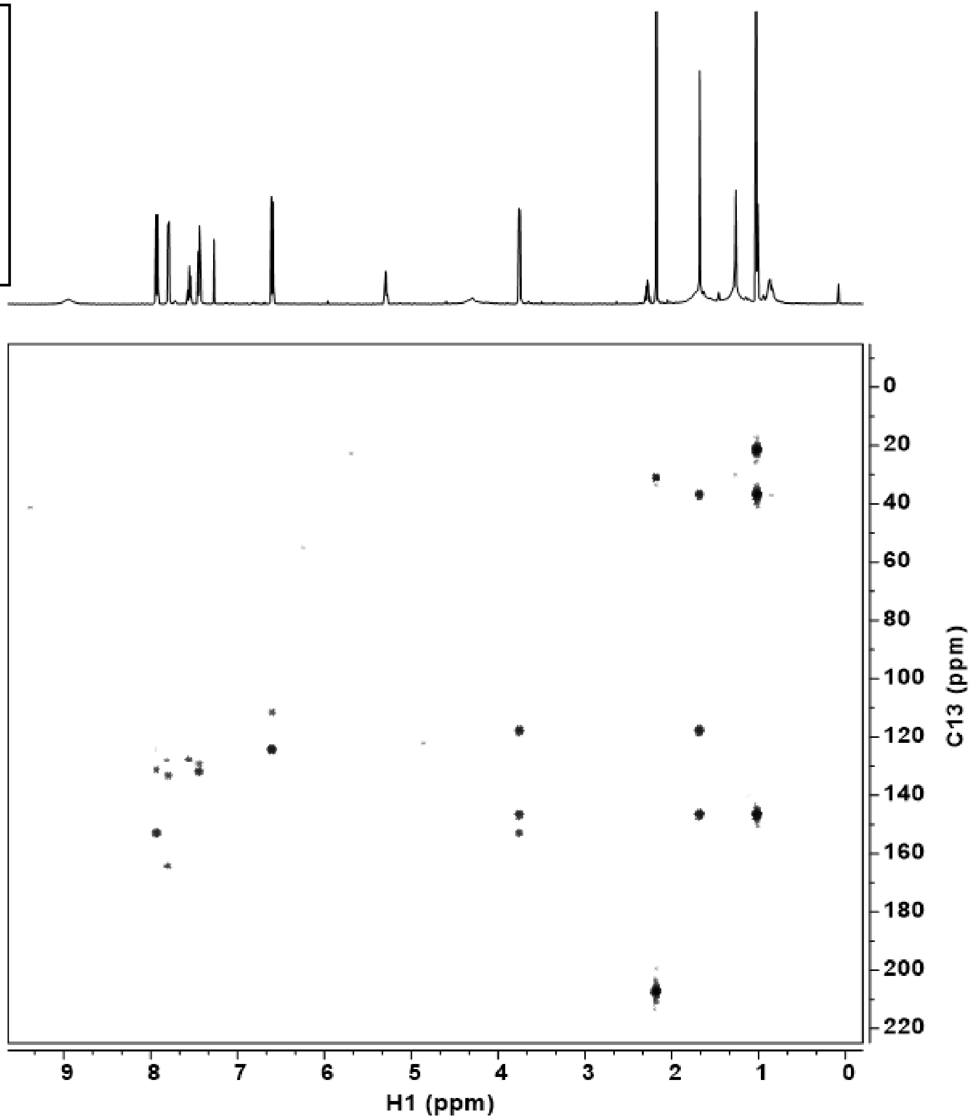

2D ^{1}H-^{13}C HMBC NMR Spectrum of Sulfabenzamide-**10** (**70**) (500 MHz, $CDCl_3$)

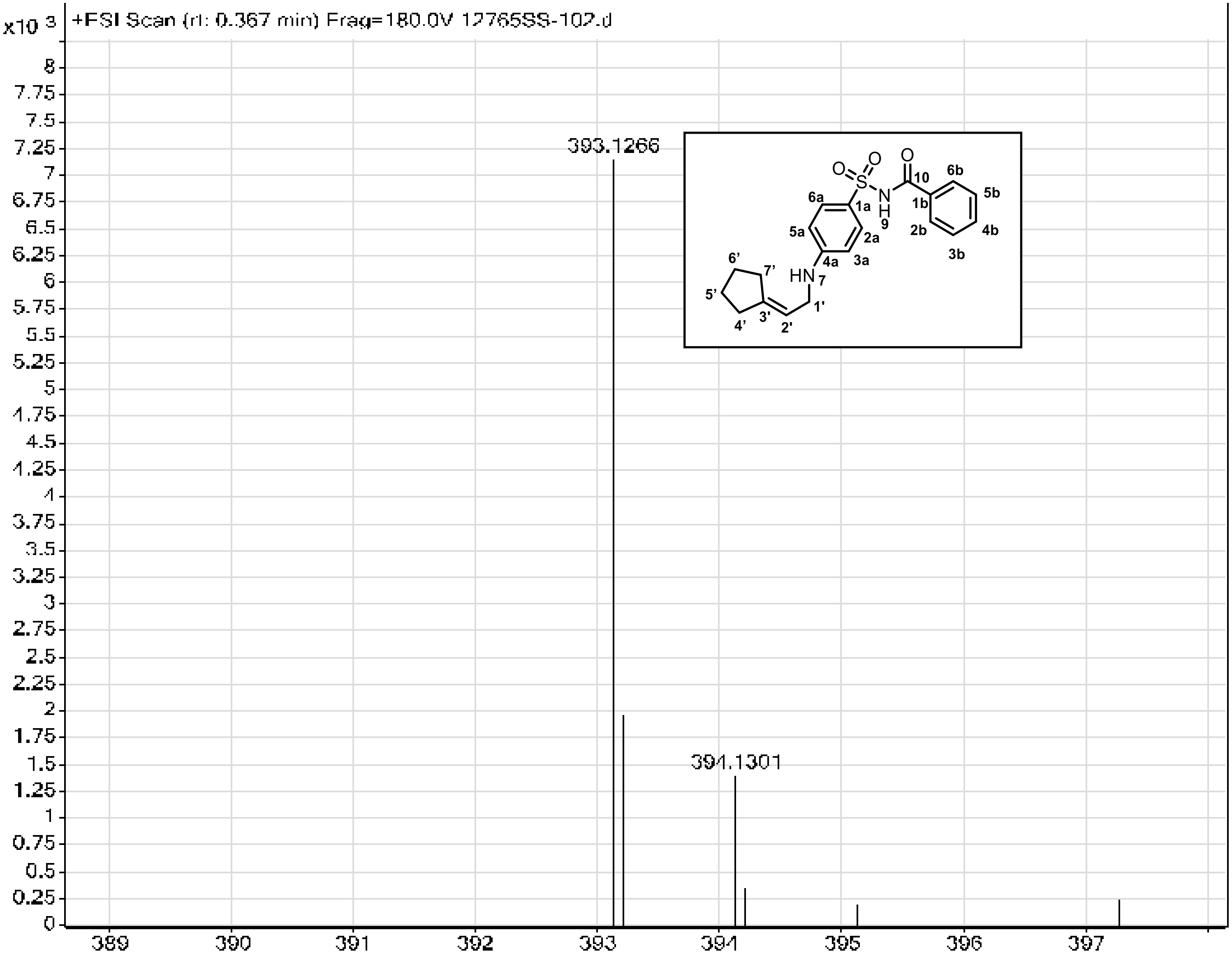

(+)-ESI-HRMS Spectrum of Sulfabenzamide-**12** (**71**)

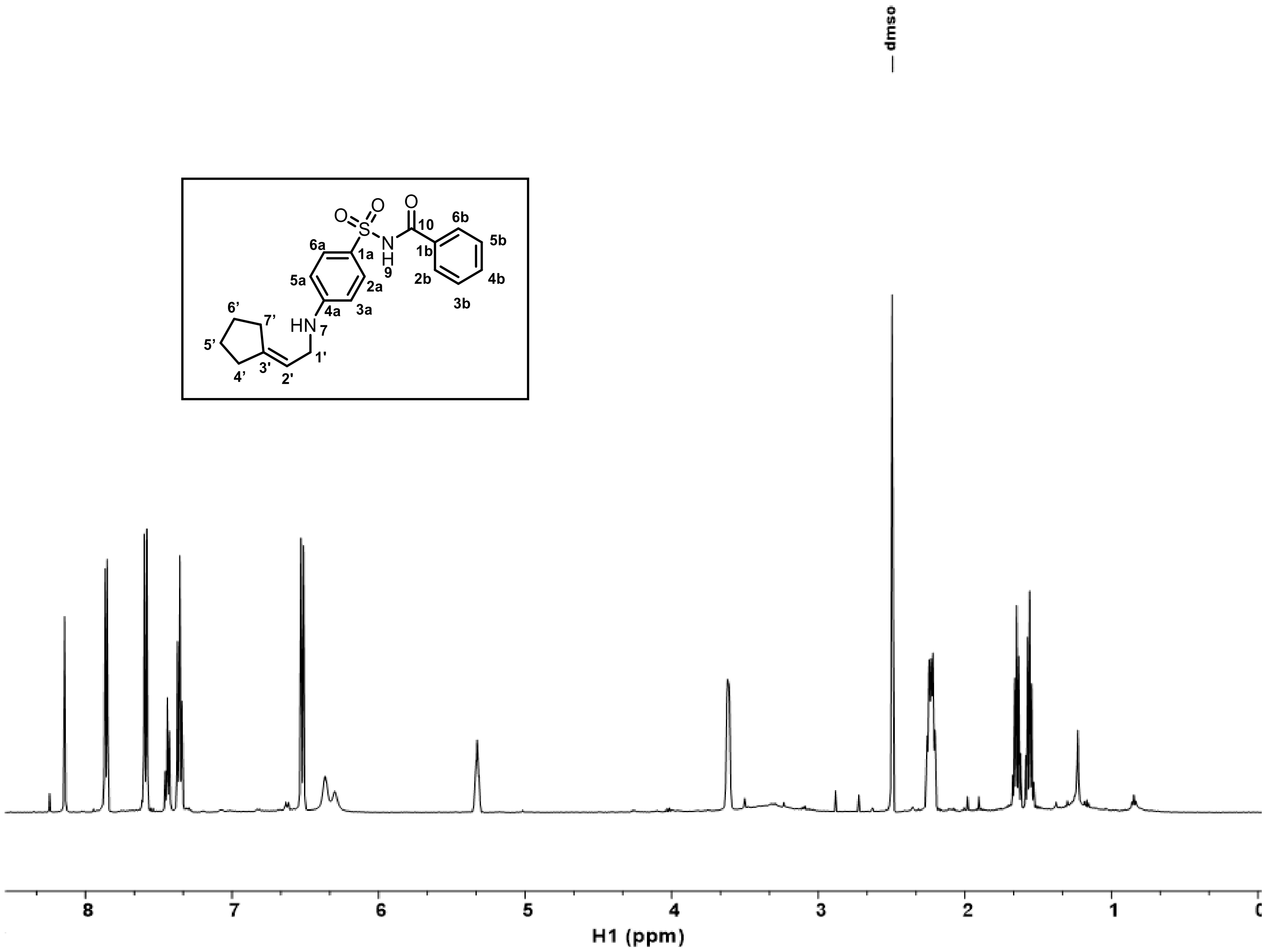

¹H NMR Spectrum of Sulfabenzamide-**12** (**71**) (500 MHz, DMSO-d6)

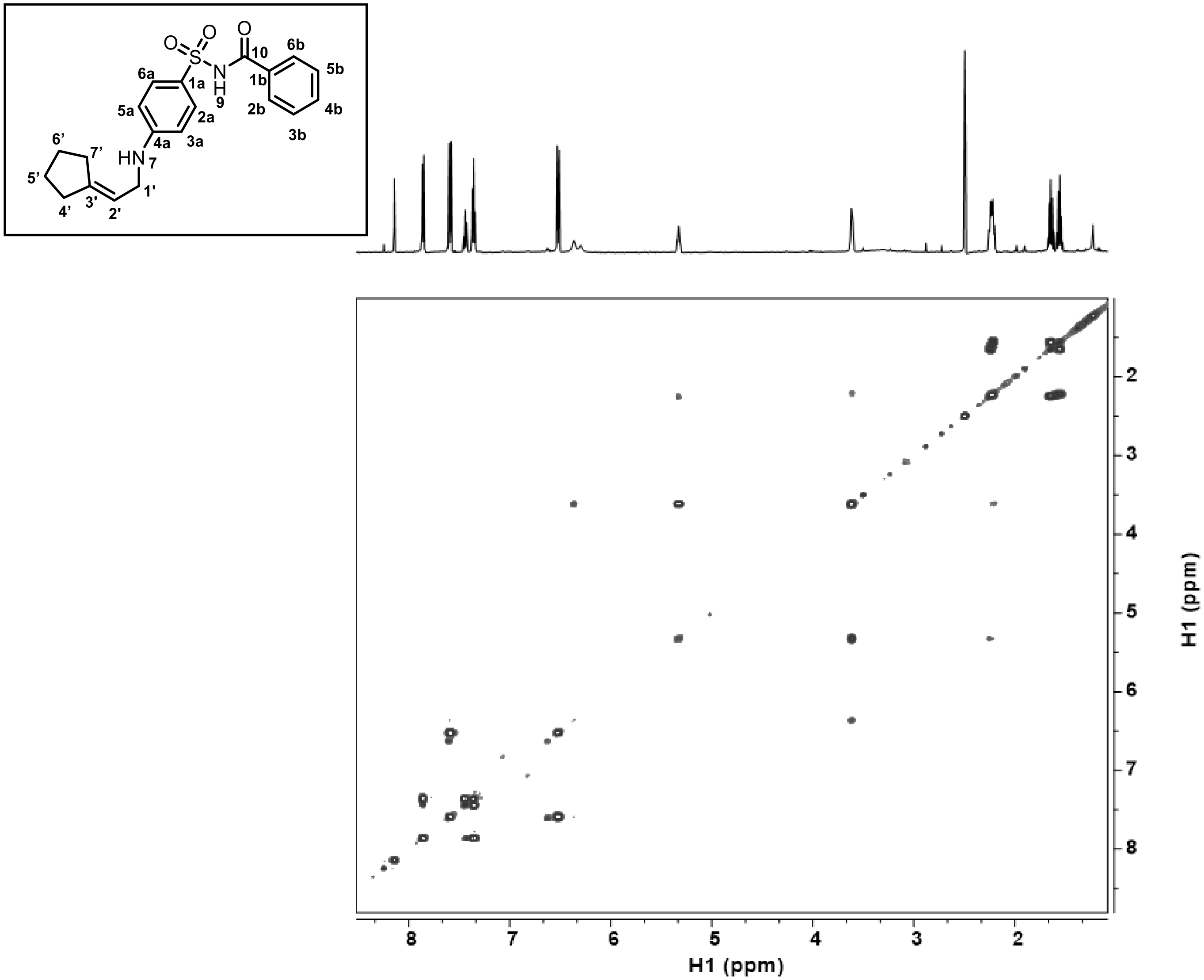

2D ^{1}H-^{1}H COSY NMR Spectrum of Sulfabenzamide-**12** (**71**) (500 MHz, DMSO-d6)

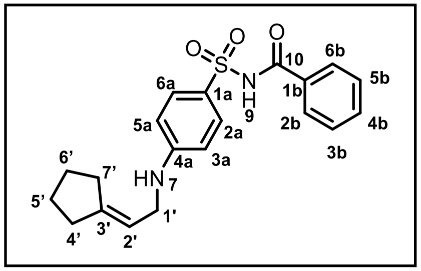

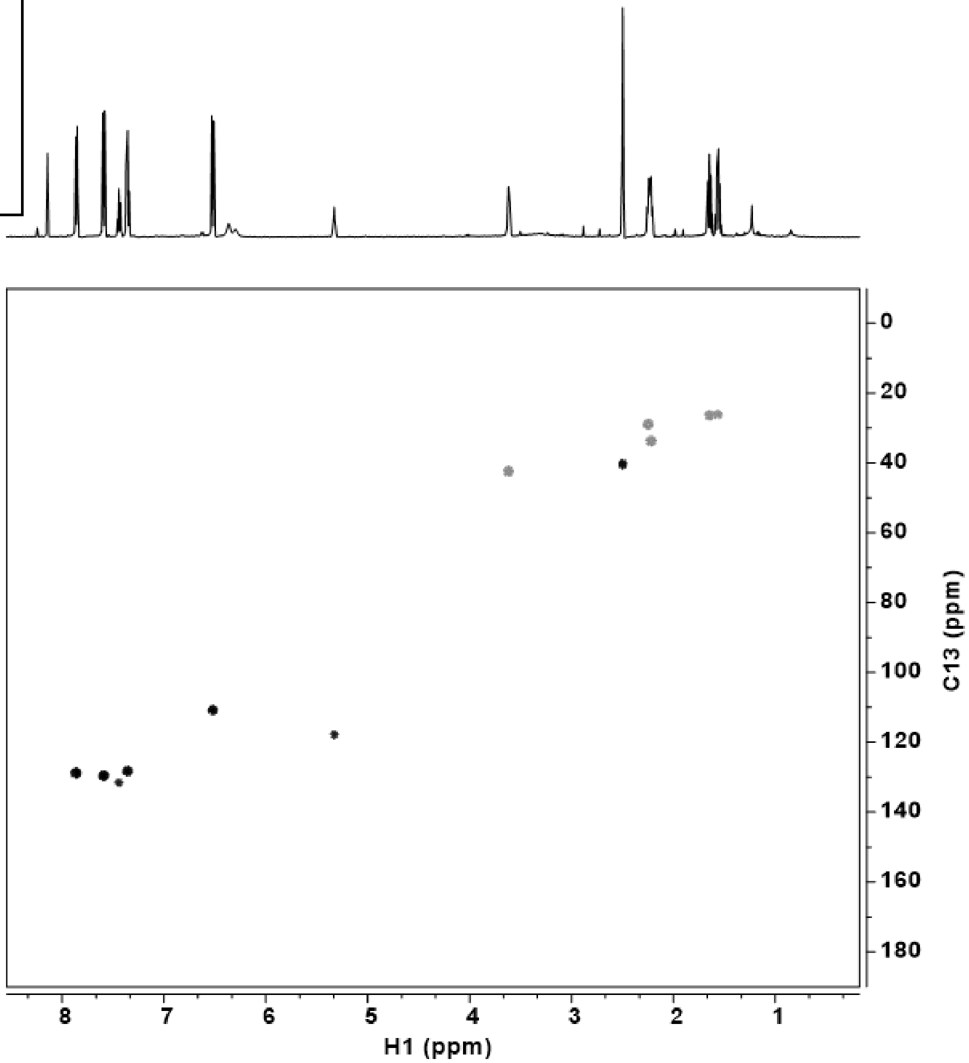

2D ^{1}H-^{13}C HSQC NMR Spectrum of Sulfabenzamide-**12** (**71**) (500 MHz, DMSO-d6)

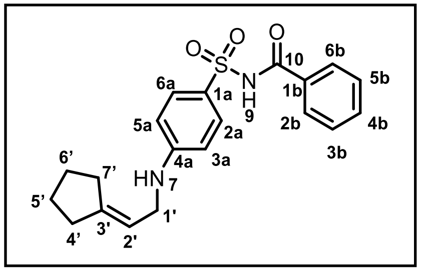

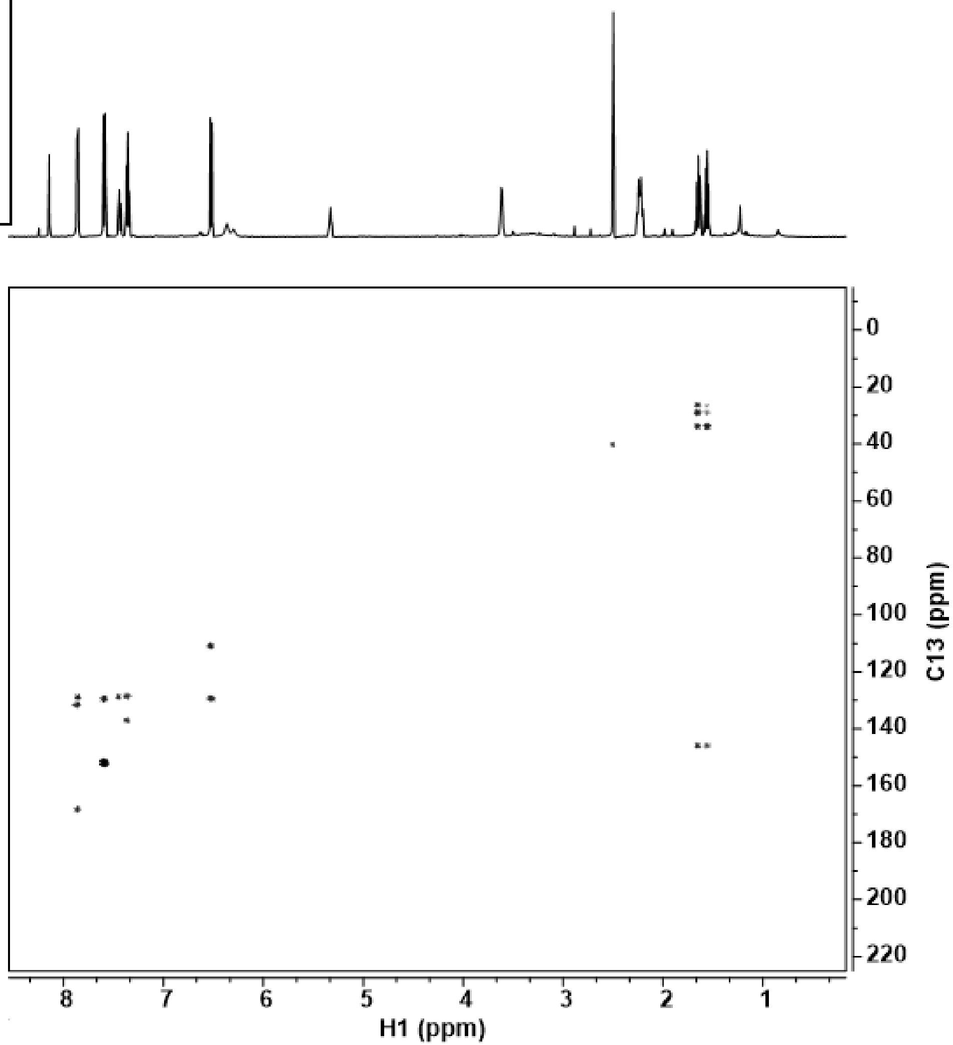

2D ^{1}H-^{13}C HMBC NMR Spectrum of Sulfabenzamide-**12** (**71**) (500 MHz, DMSO-d6)

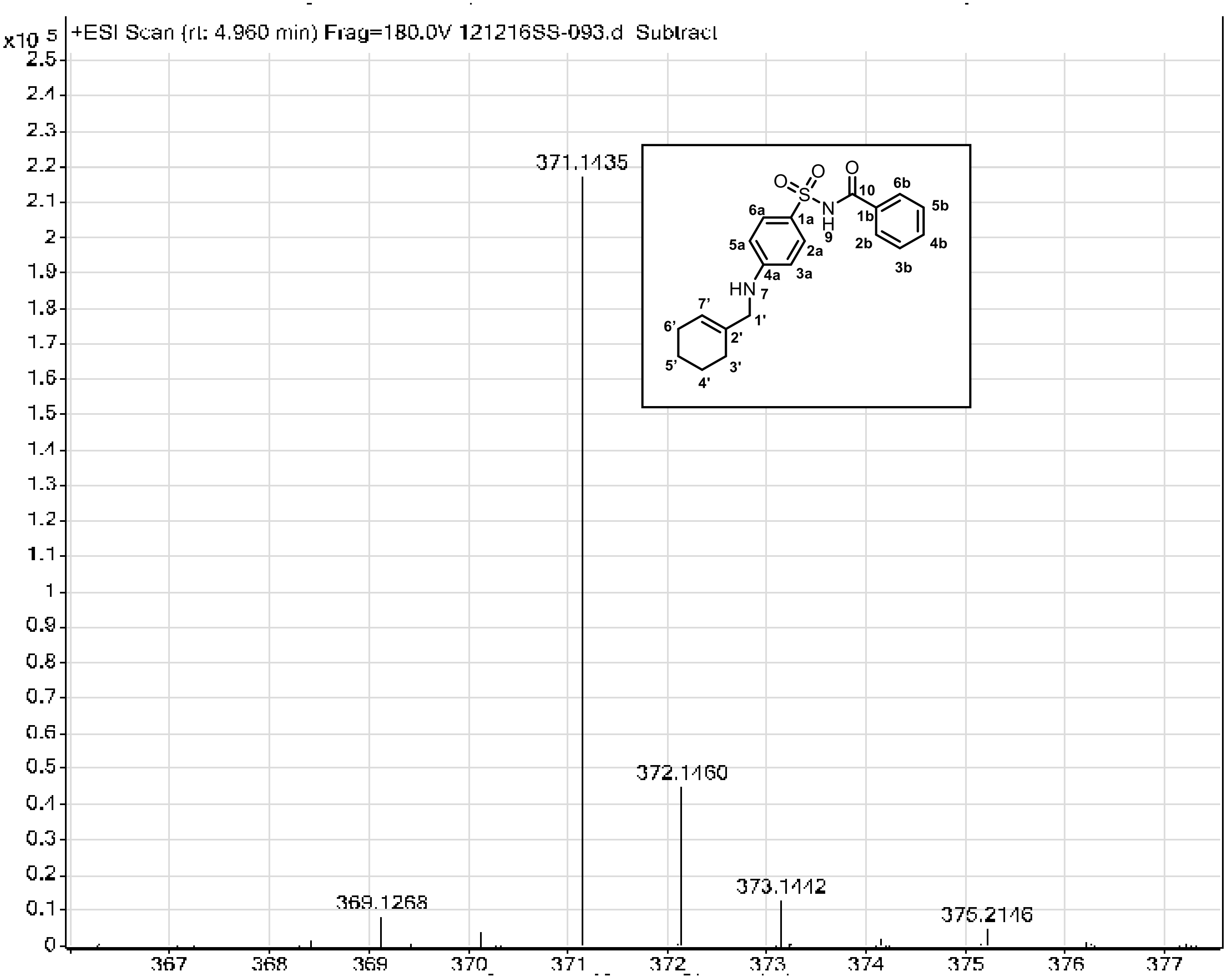

(+)-ESI-HRMS Spectrum of Sulfabenzamide-**13** (**72**)

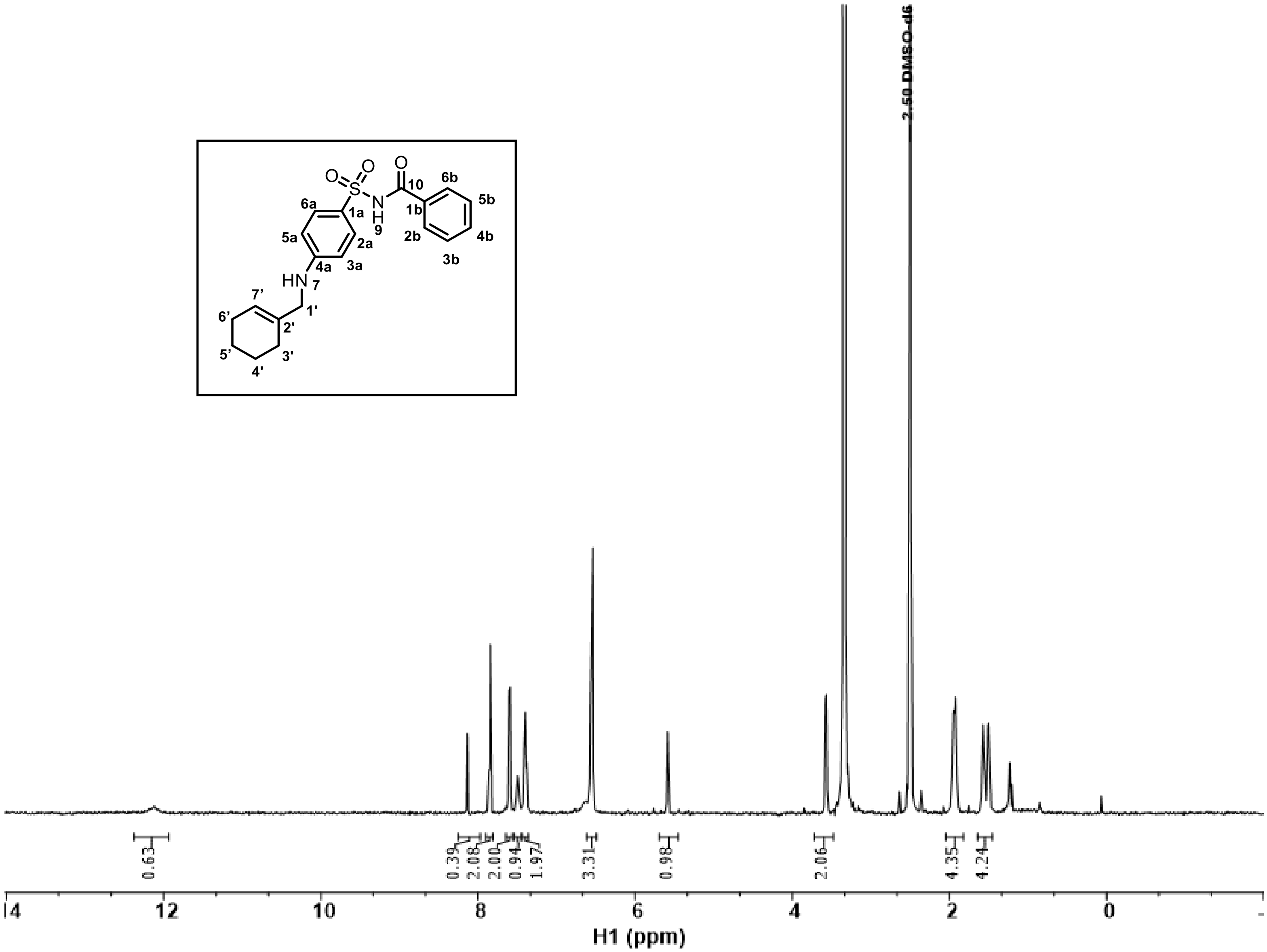

1H NMR Spectrum of Sulfabenzamide-**13** (**72**) (500 MHz, DMSO-d6)

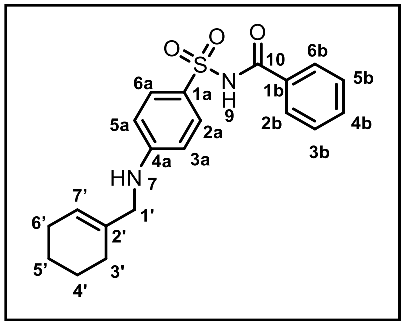

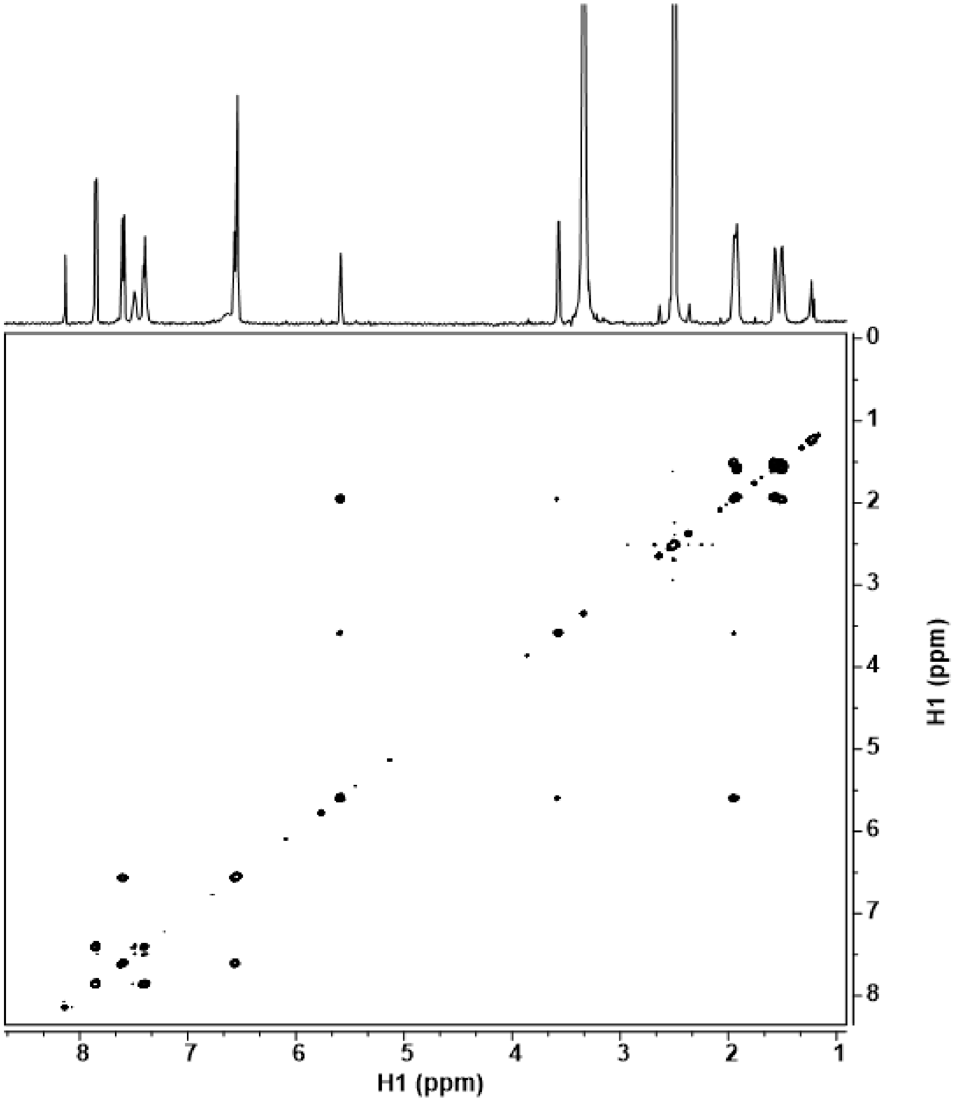

2D ^{1}H-^{1}H COSY NMR Spectrum of Sulfabenzamide-**13** (**72**) (500 MHz, DMSO-d6)

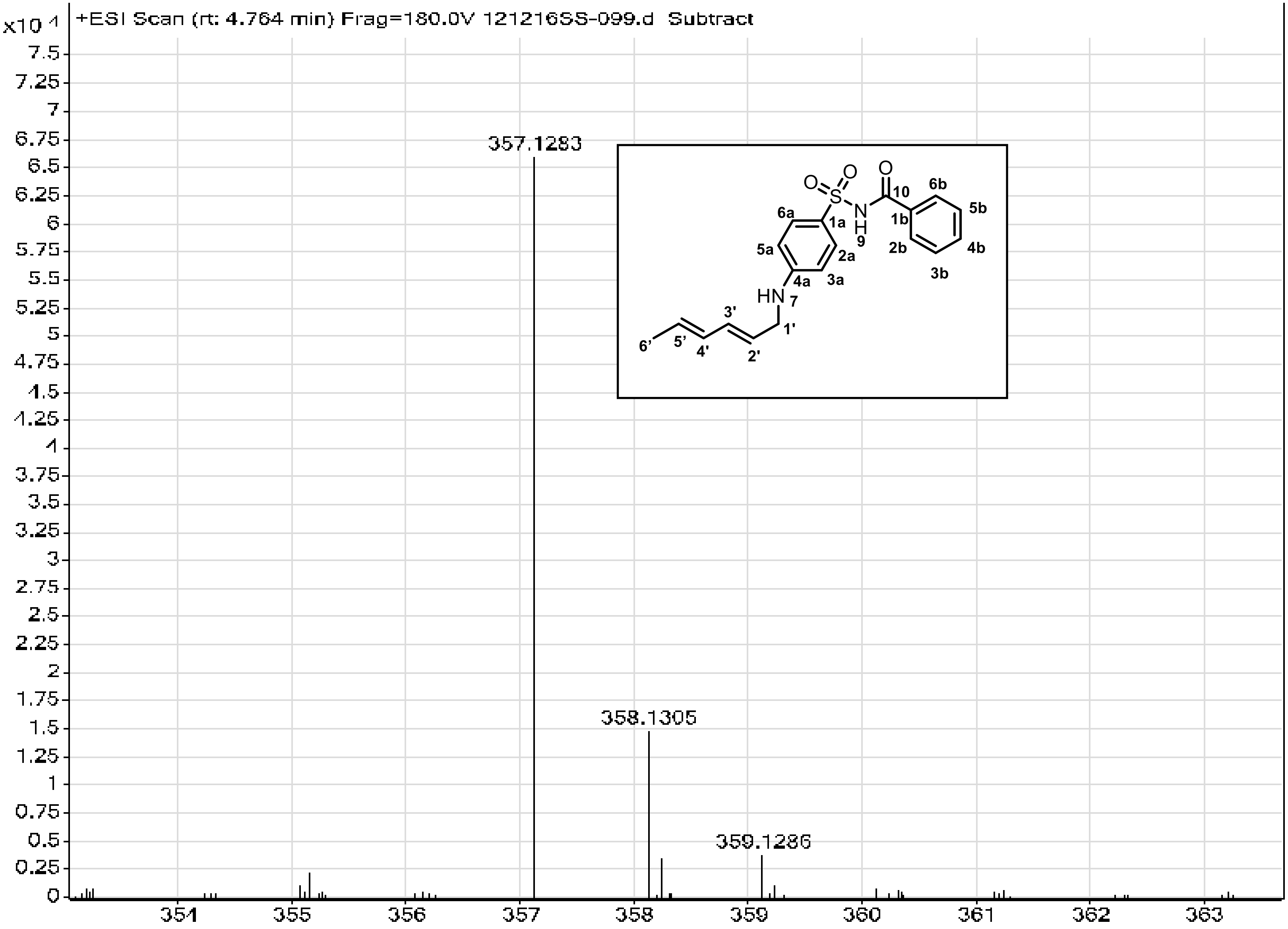

(+)-ESI-HRMS Spectrum of Sulfabenzamide-**16** (**73**)

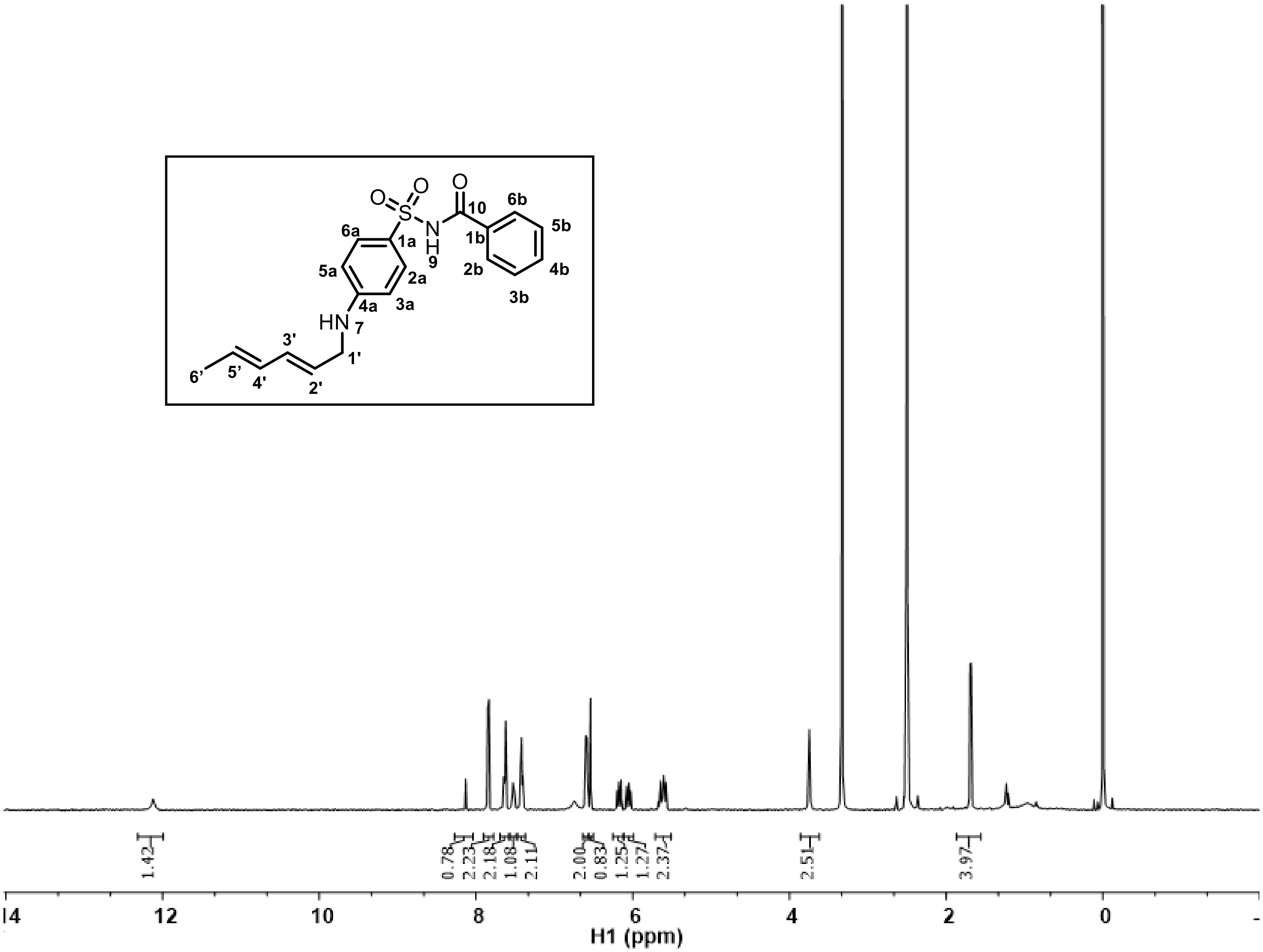

^{1}H NMR Spectrum of Sulfabenzamide-**16** (**73**) (500 MHz, DMSO-d6)

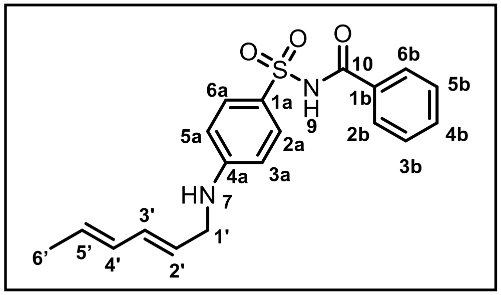

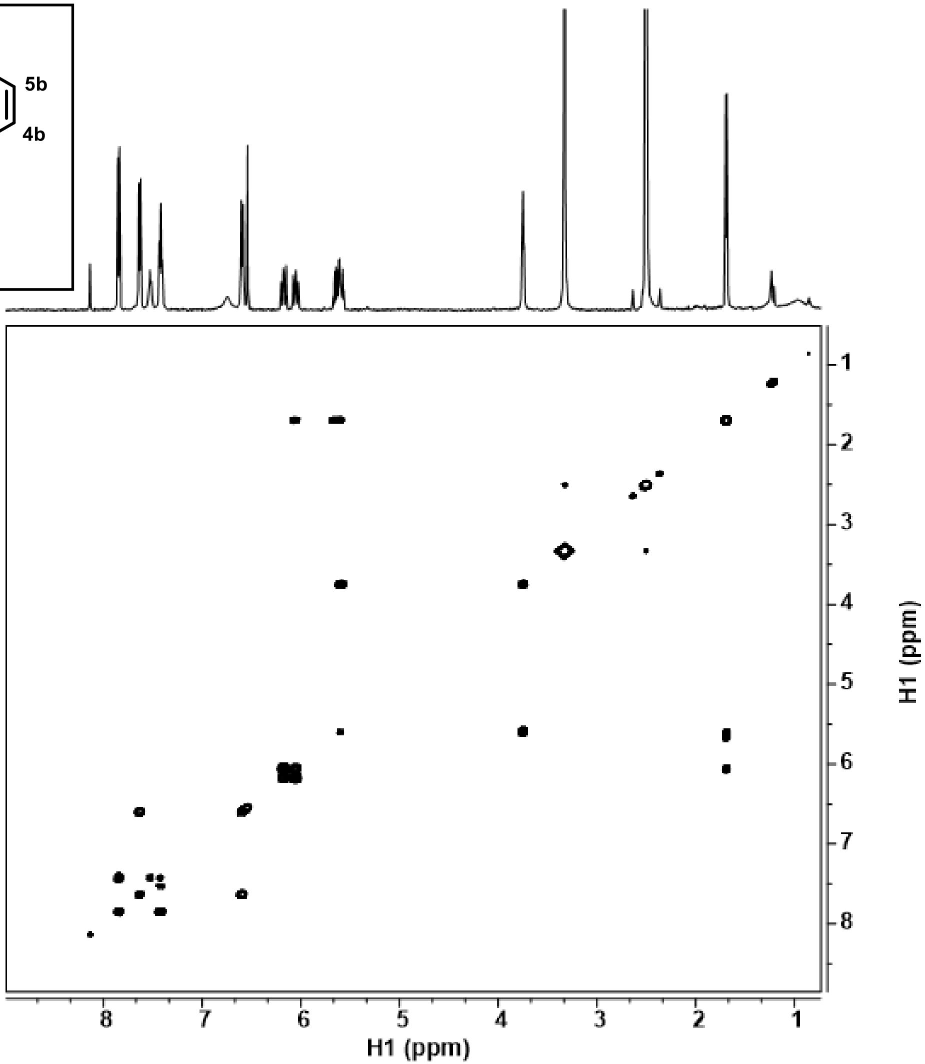

2D ^{1}H-^{1}H COSY NMR Spectrum of Sulfabenzamide-**16** (**73**) (500 MHz, DMSO-d6)

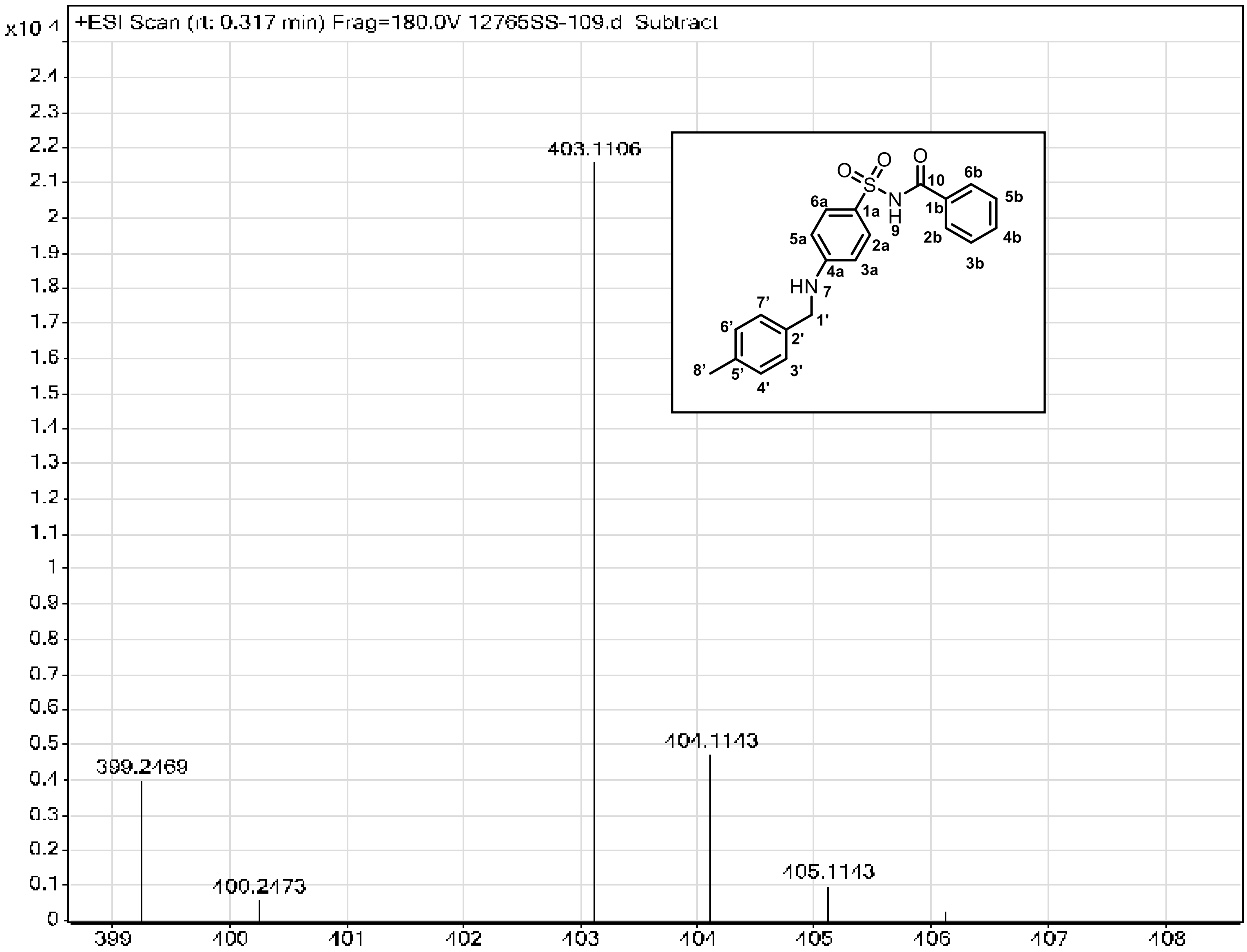

(+)-ESI-HRMS Spectrum of Sulfabenzamide-**46** (**74**)

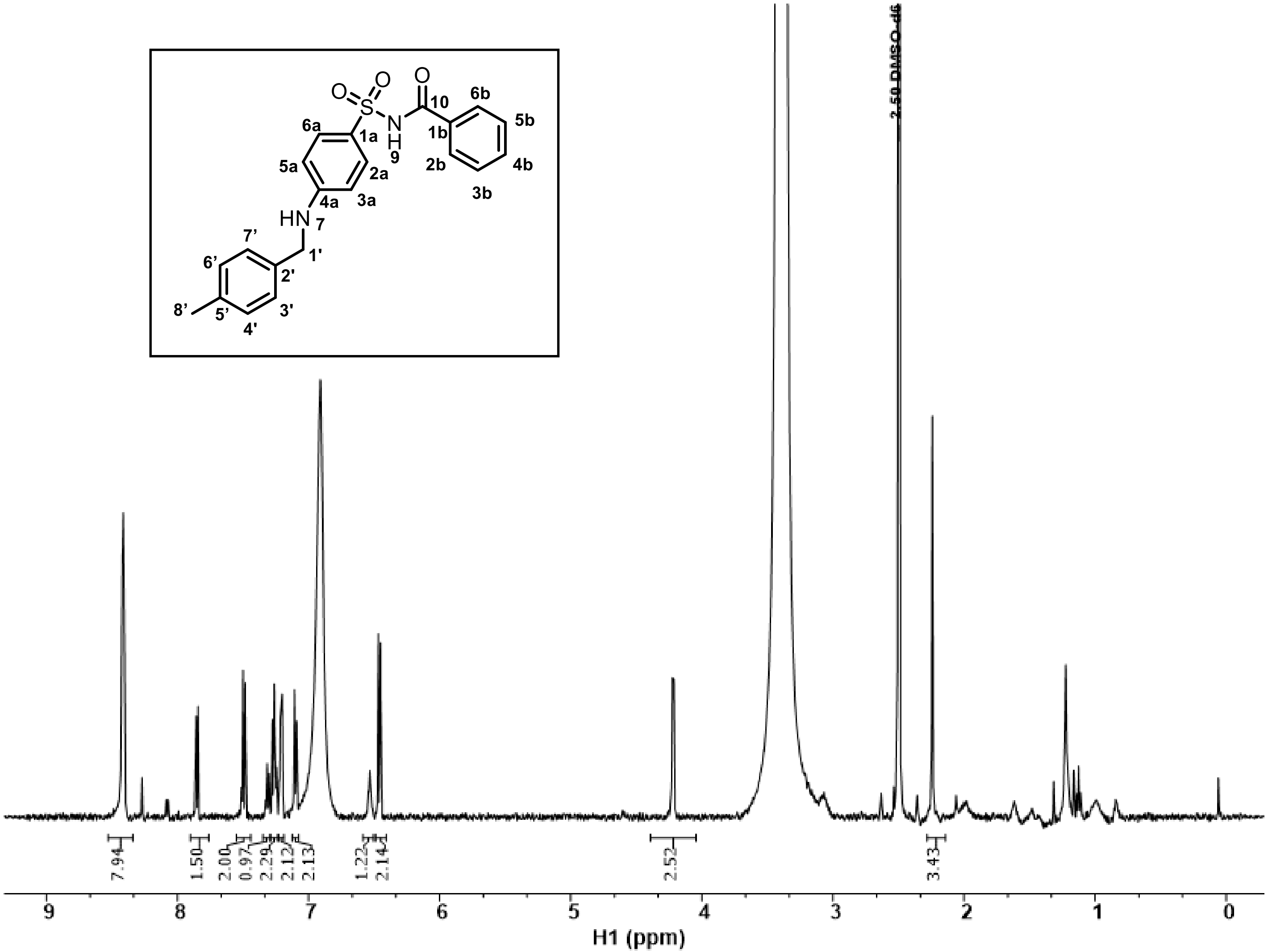

^{1}H NMR Spectrum of Sulfabenzamide-**46** (**74**) (500 MHz, DMSO-d6)

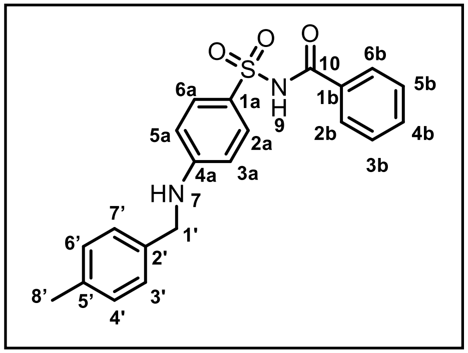

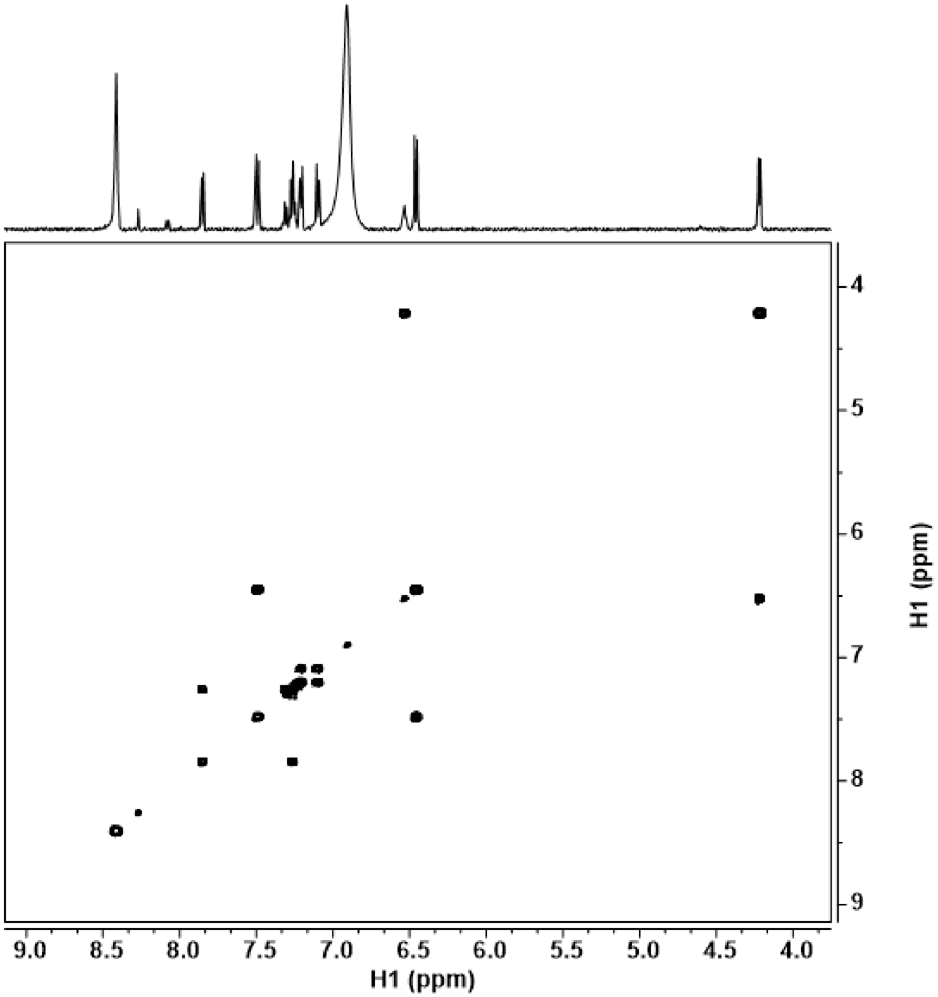

2D ^{1}H-^{1}H COSY NMR Spectrum of Sulfabenzamide-**46** (**74**) (500 MHz, DMSO-d6)

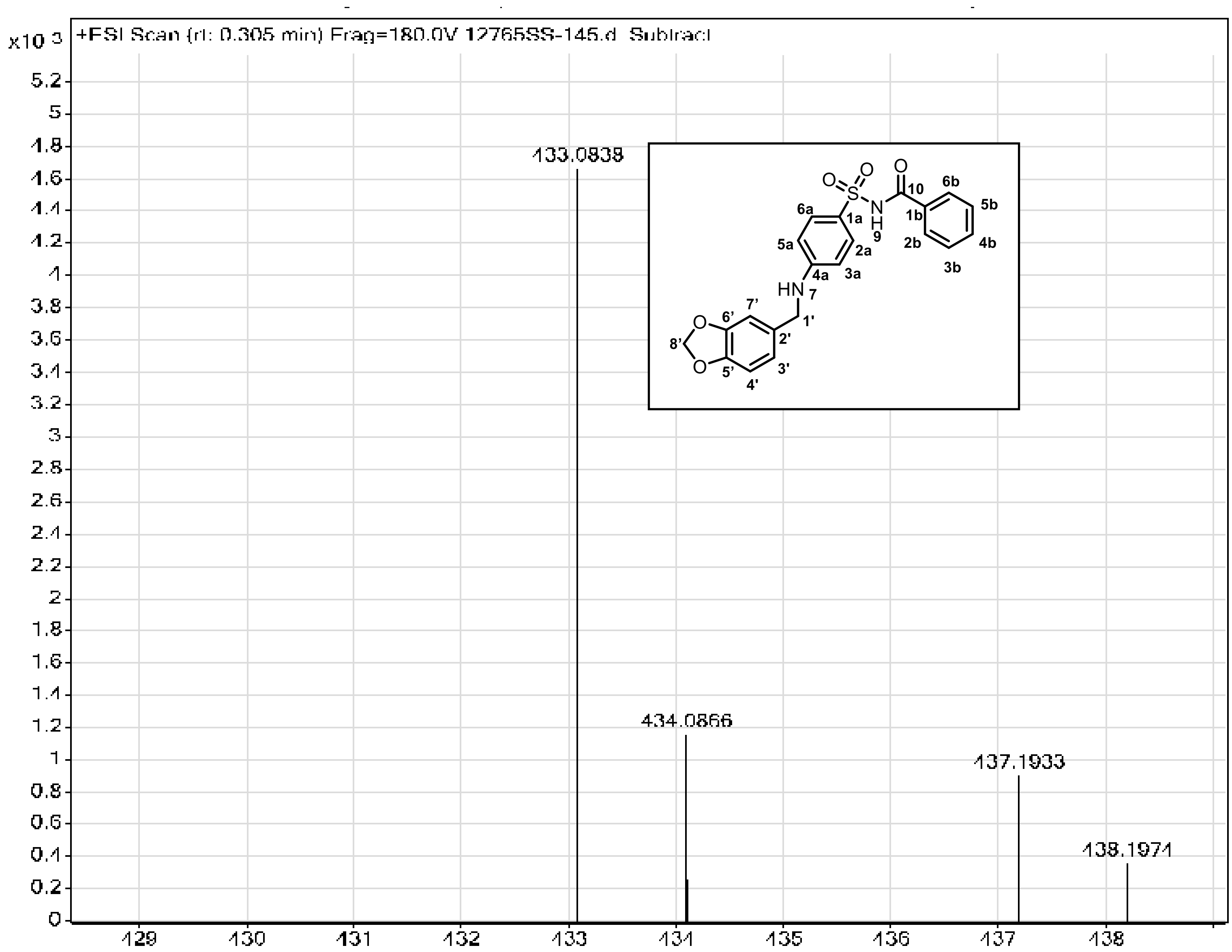

(+)-ESI-HRMS Spectrum of Sulfabenzamide-**58** (**75**)

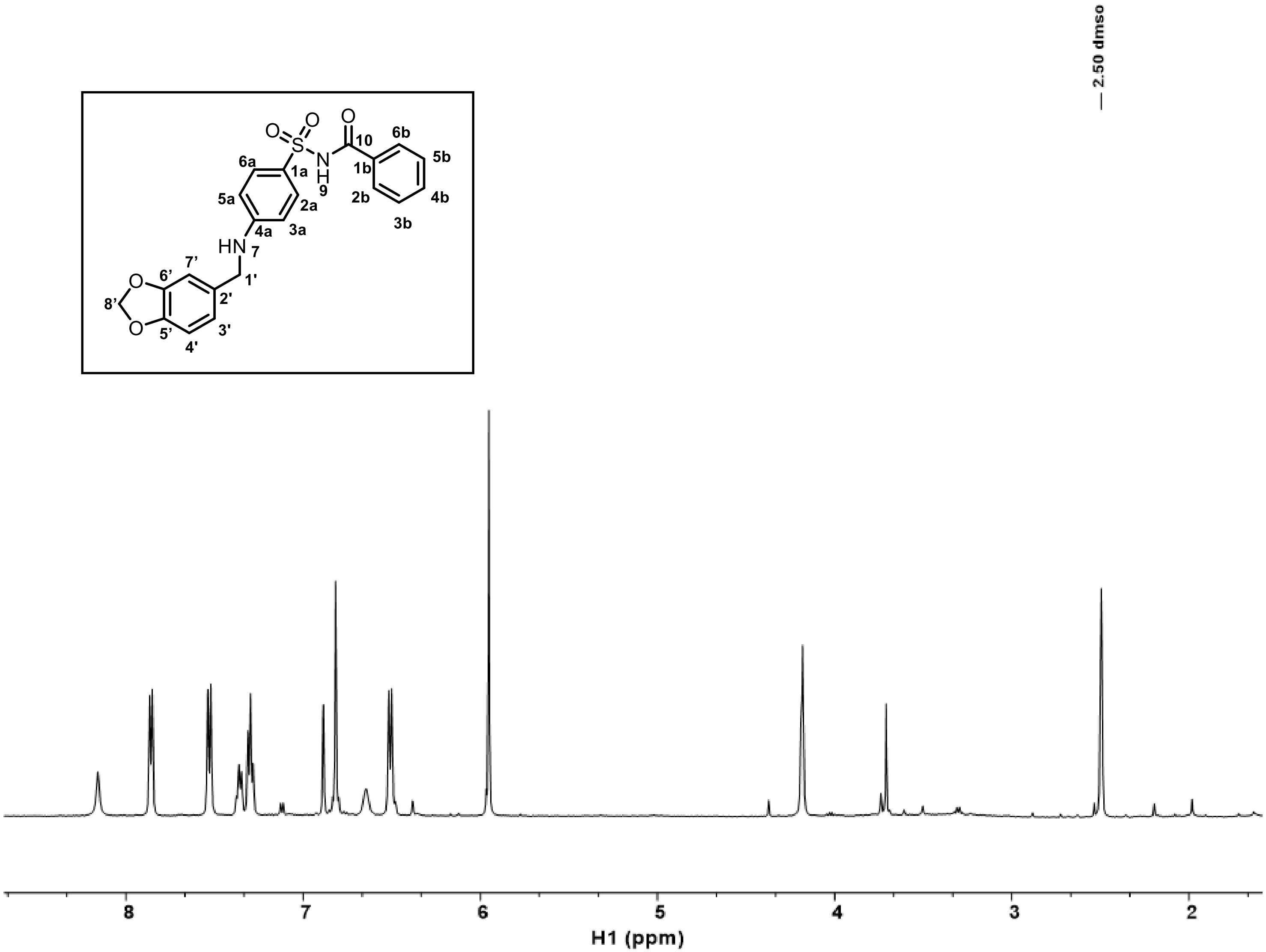

^{1}H NMR Spectrum of Sulfabenzamide-**58** (**75**) (500 MHz, DMSO-d6)

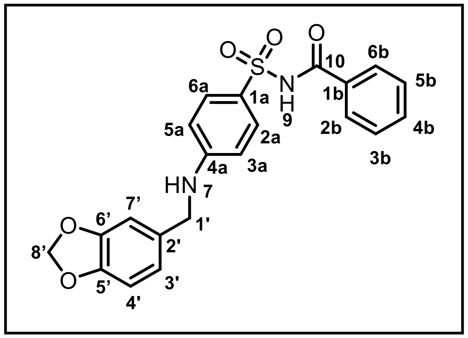

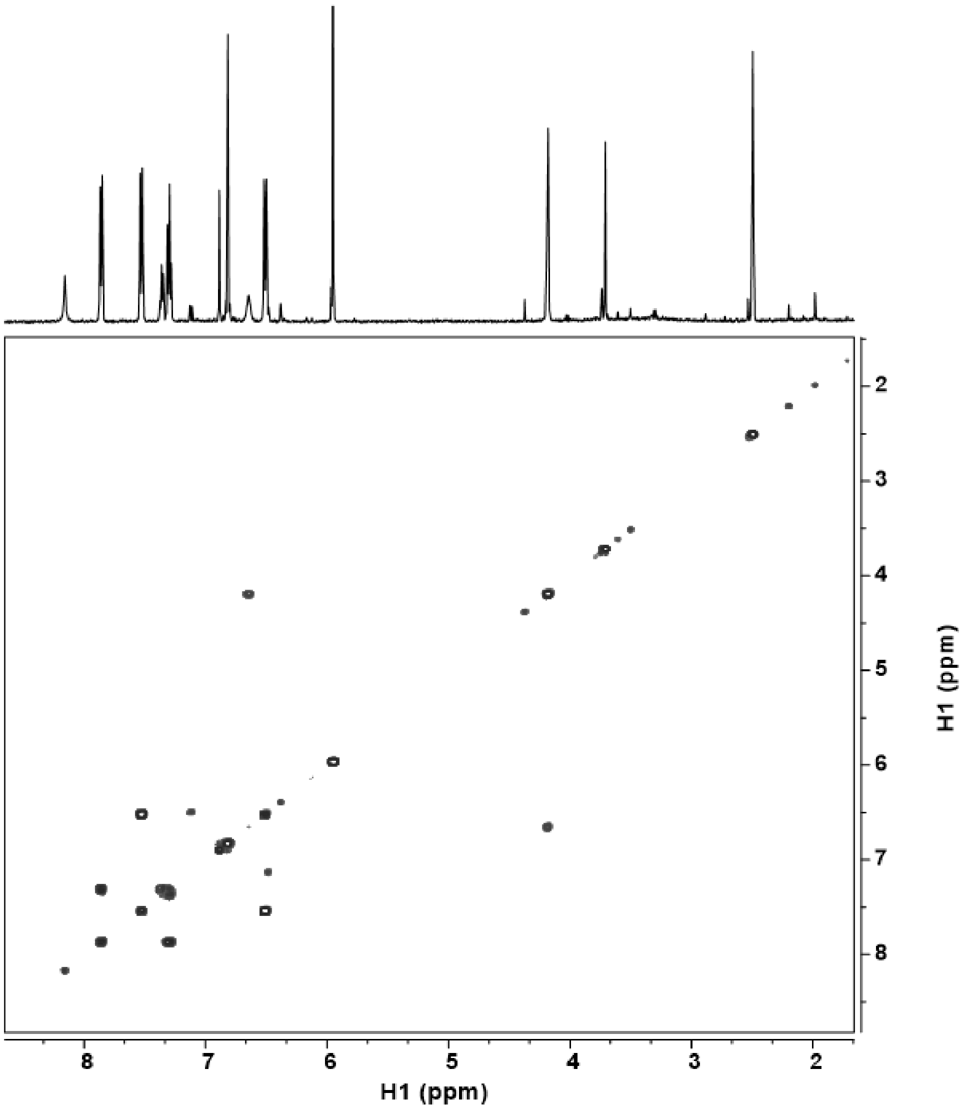

2D ^{1}H-^{1}H COSY NMR Spectrum of Sulfabenzamide-**58** (**75**) (500 MHz, DMSO-d6)

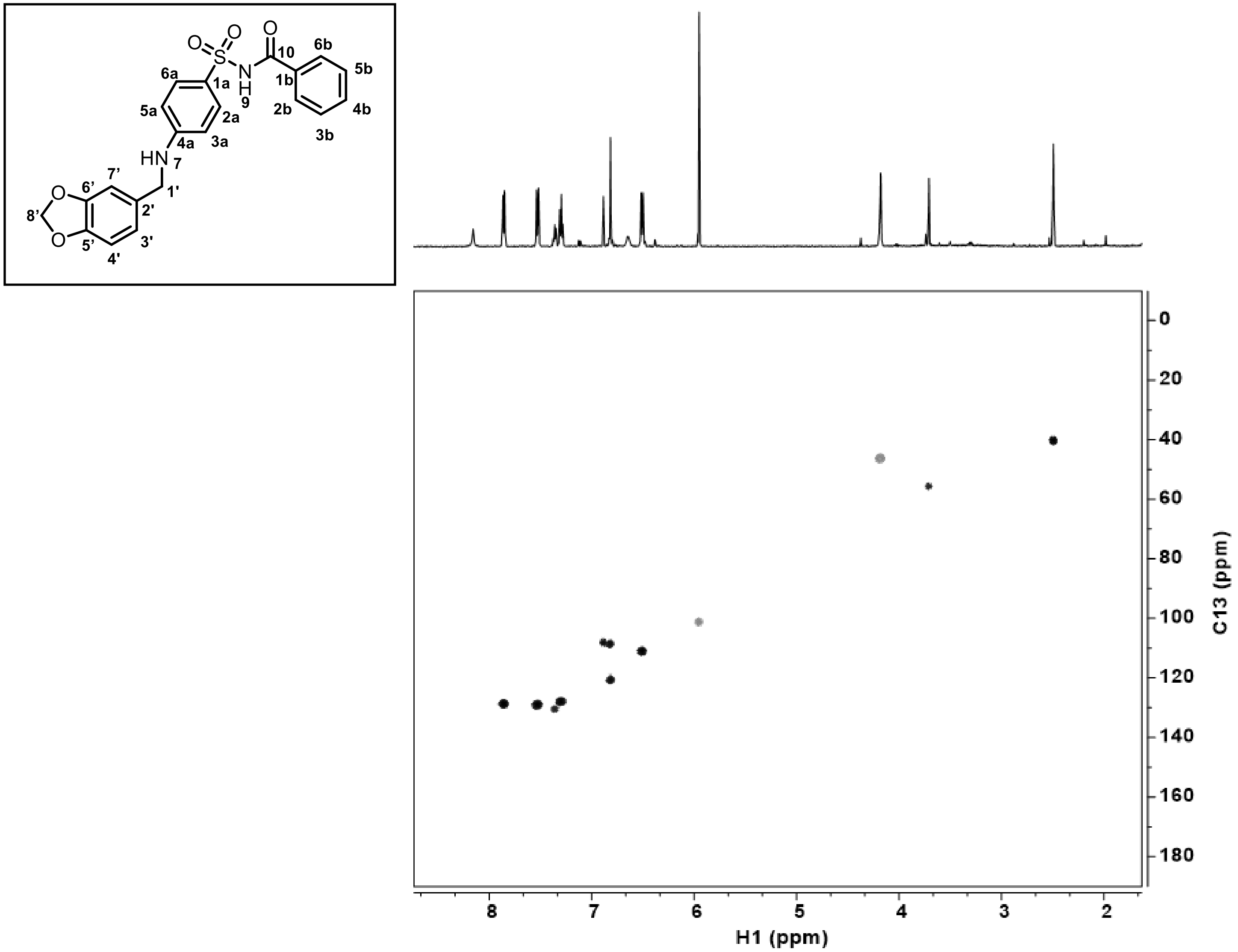

2D ^{1}H-^{13}C HSQC NMR Spectrum of Sulfabenzamide-**58** (**75**) (500 MHz, DMSO-d6)

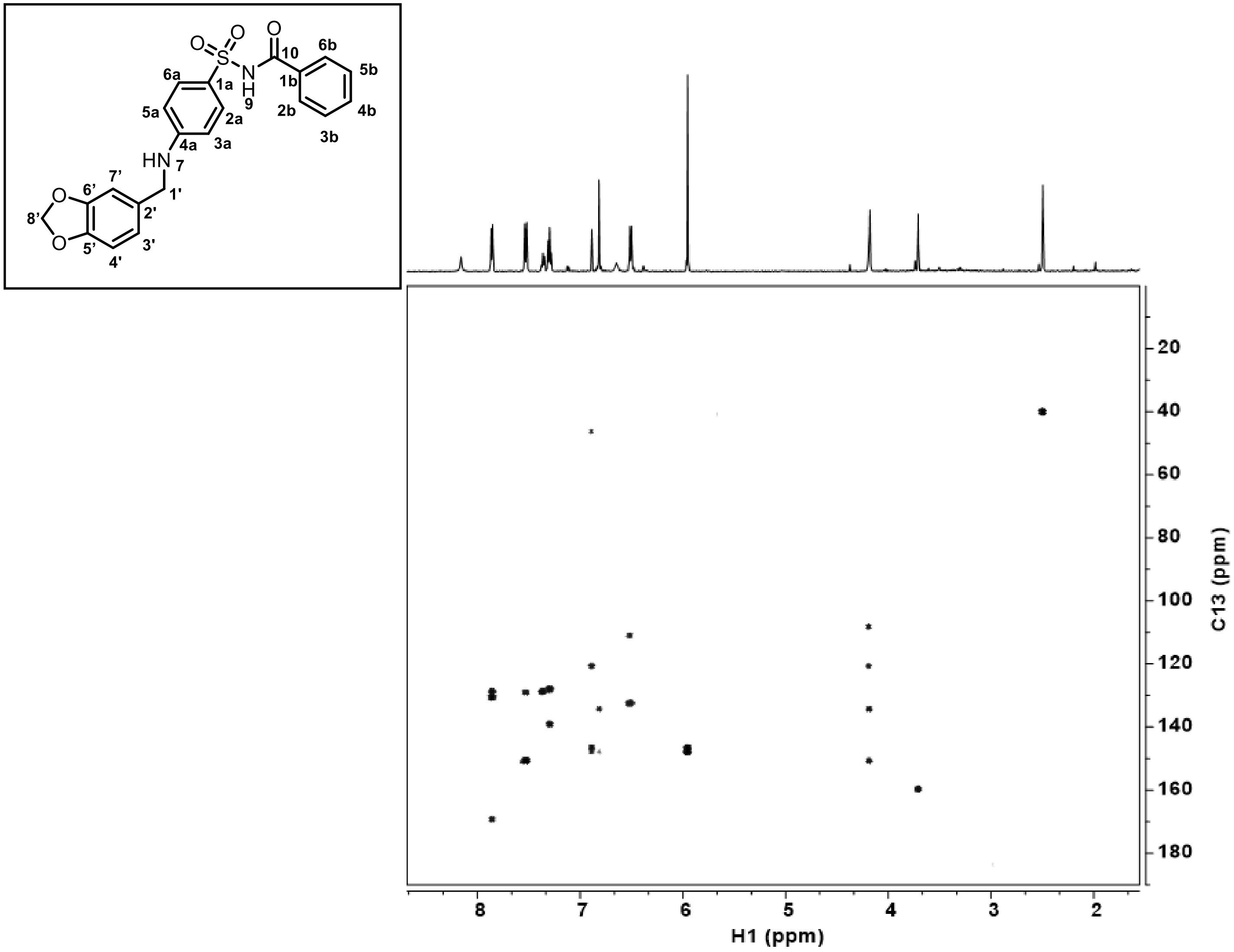

2D ^{1}H-^{13}C HMBC NMR Spectrum of Sulfabenzamide-**58** (**75**) (500 MHz, DMSO-d6)

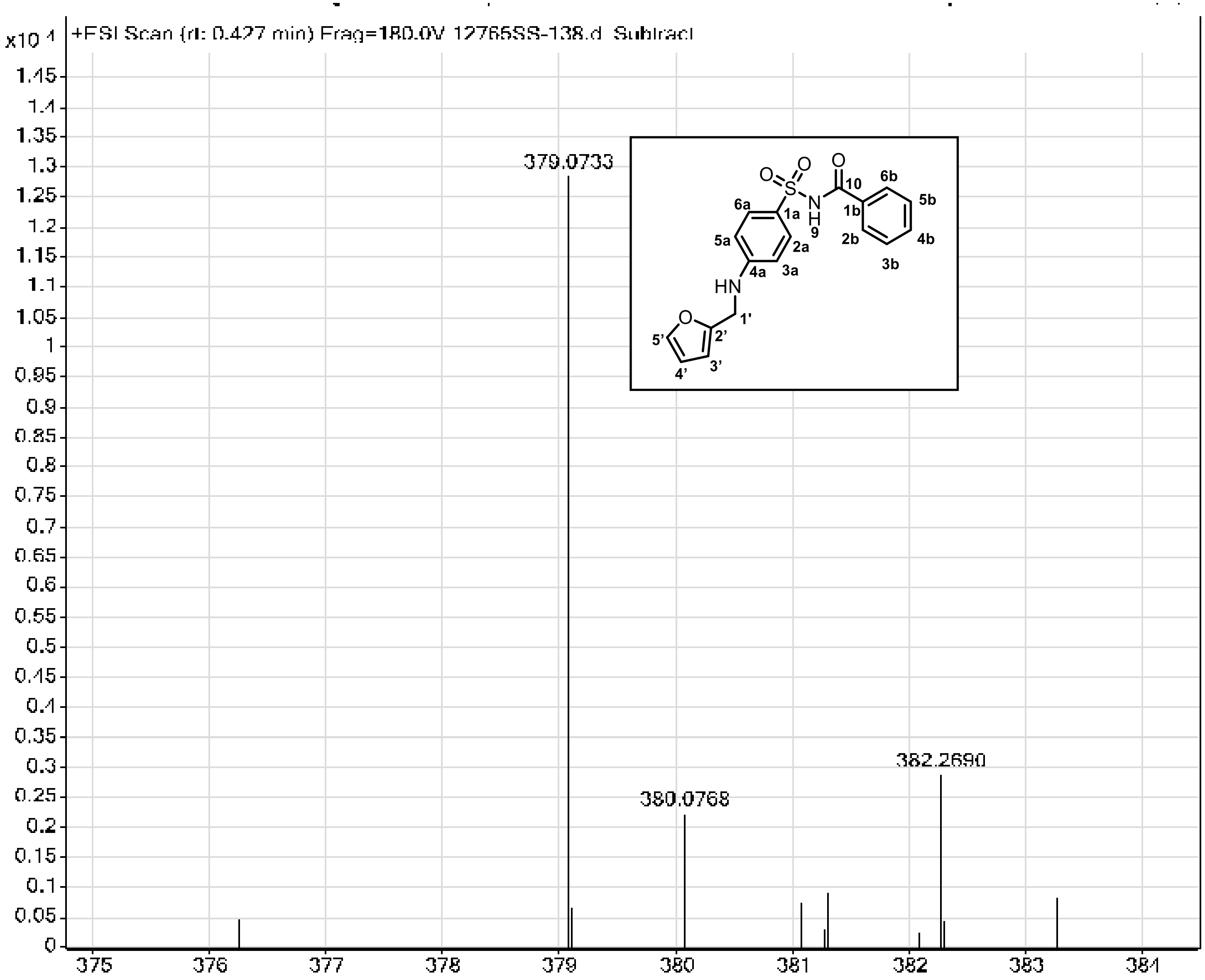

(+)-ESI-HRMS Spectrum of Sulfabenzamide-**61** (**76**)

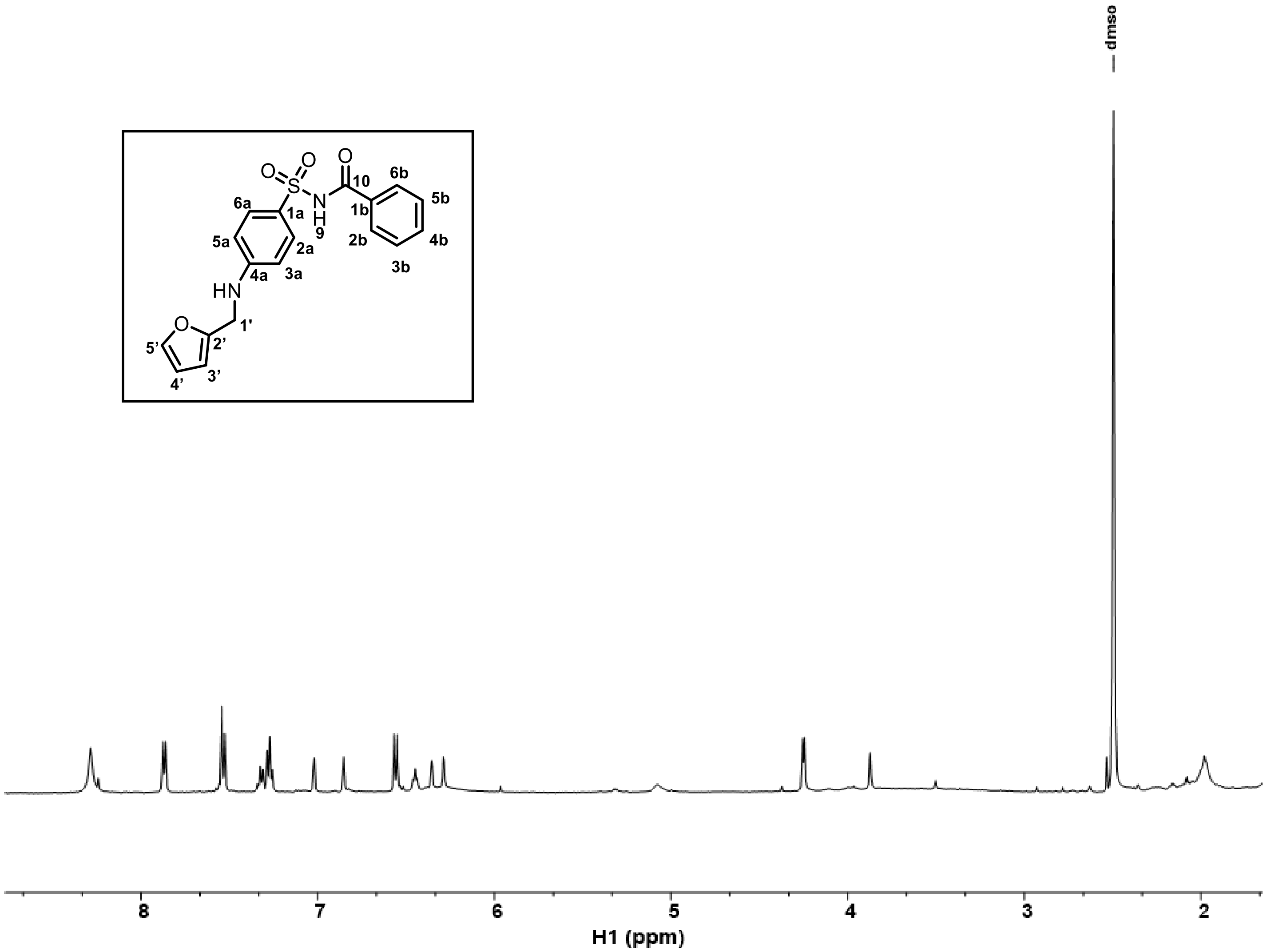

^{1}H NMR Spectrum of Sulfabenzamide-**61** (**76**) (500 MHz, DMSO-d6)

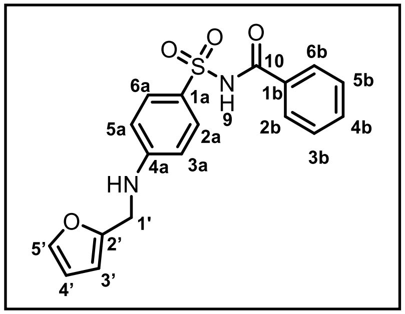

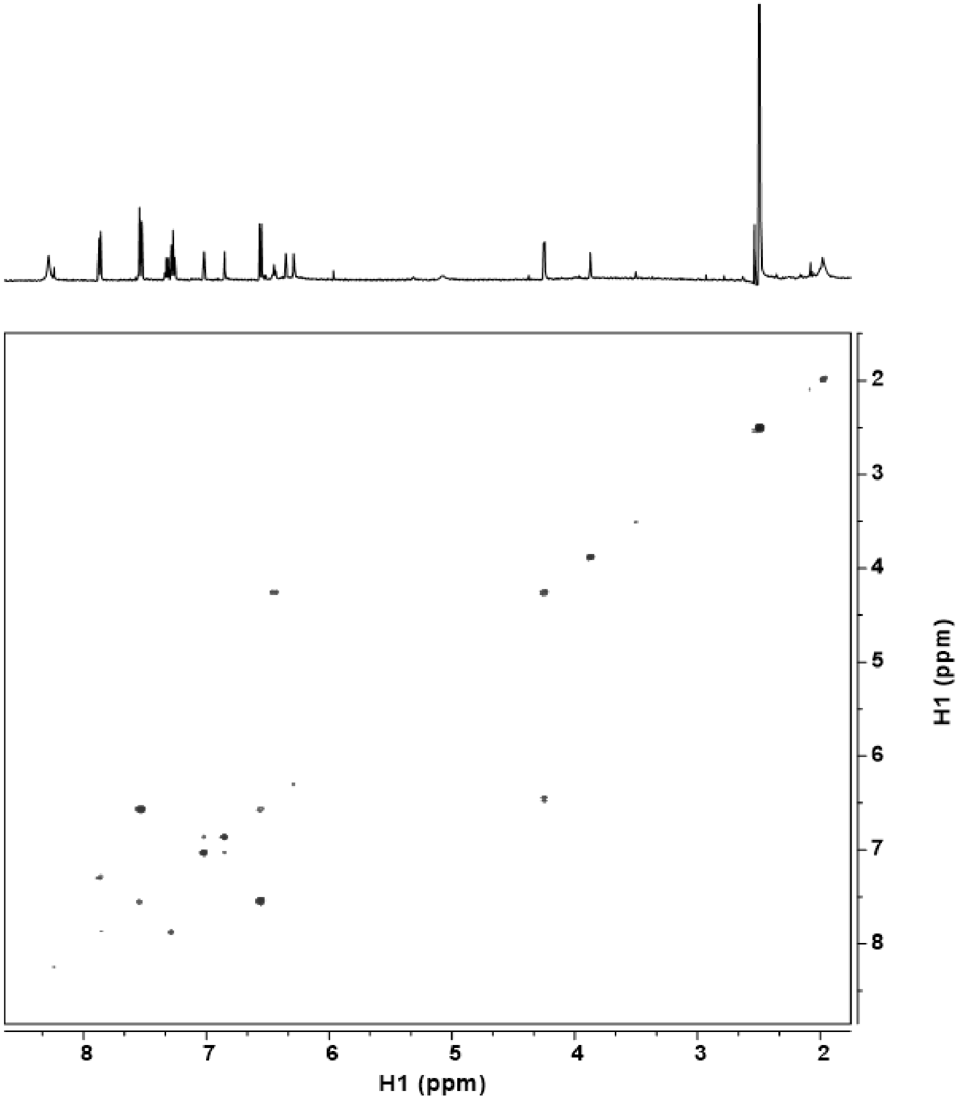

2D ^{1}H-^{1}H COSY NMR Spectrum of Sulfabenzamide-**61** (**76**) (500 MHz, DMSO-d6)

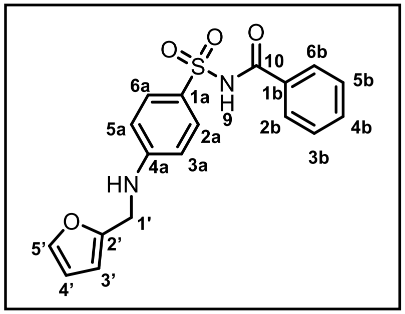

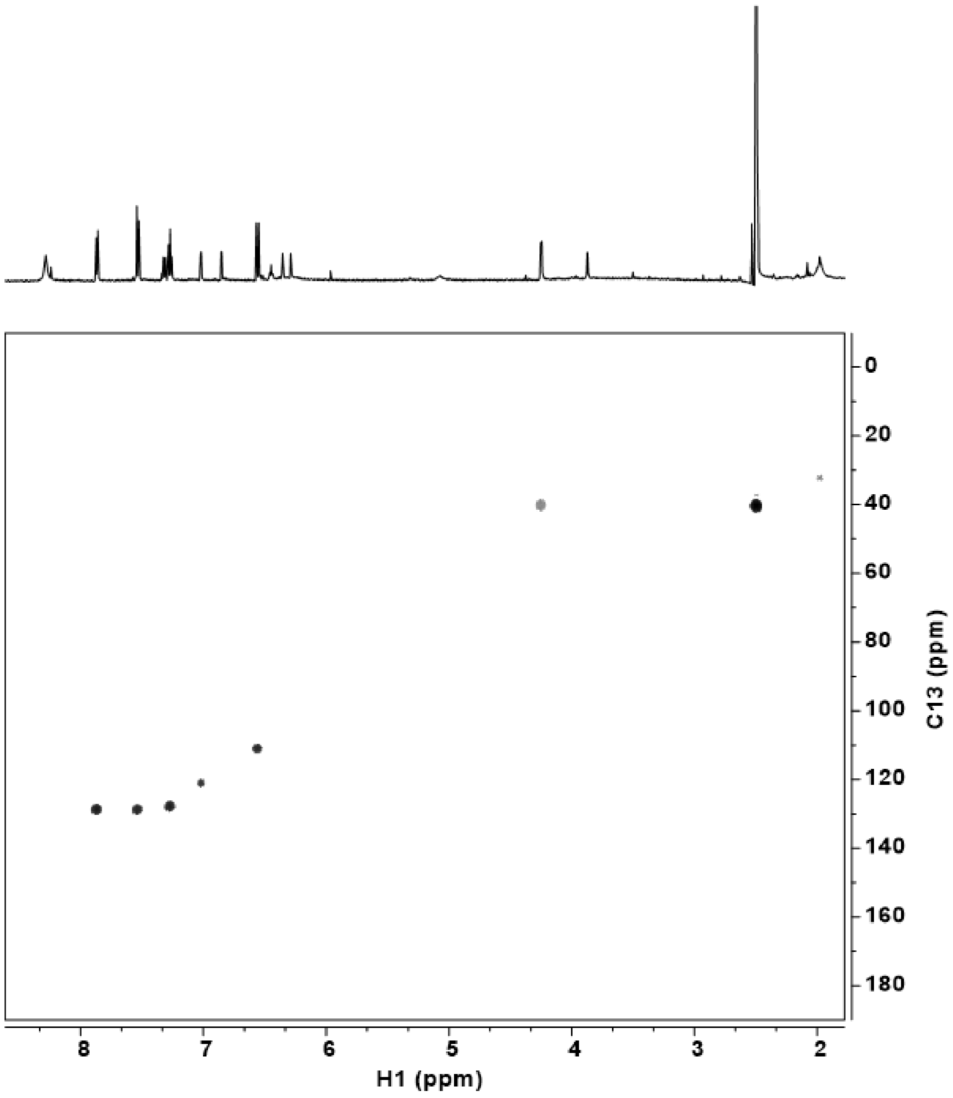

2D ^{1}H-^{13}C HSQC NMR Spectrum of Sulfabenzamide-**61** (**76**) (500 MHz, DMSO-d6)

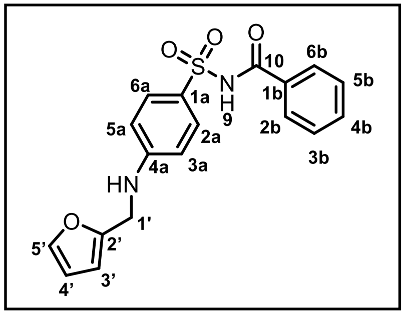

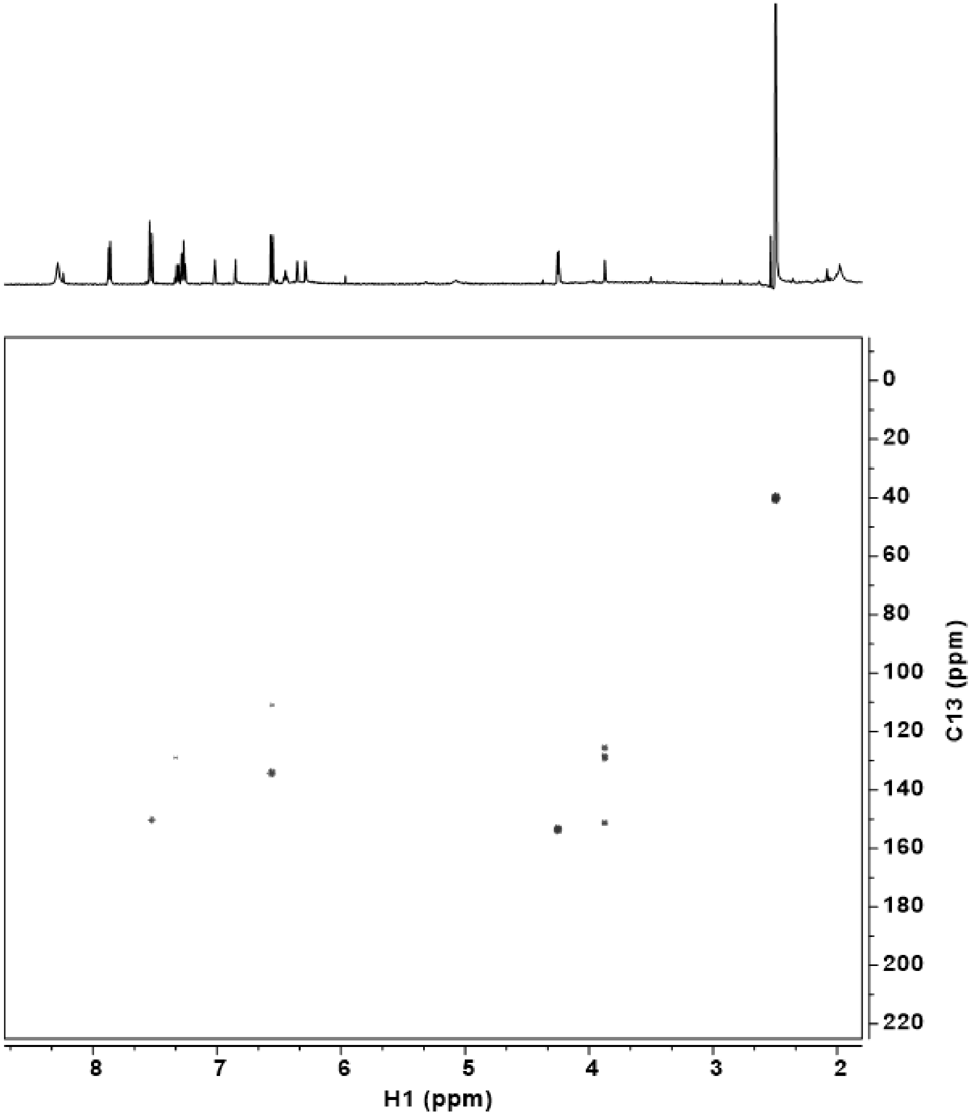

2D ^{1}H-^{13}C HMBC NMR of Sulfabenzamide-**61** (**76**) (500 MHz, DMSO-d6)

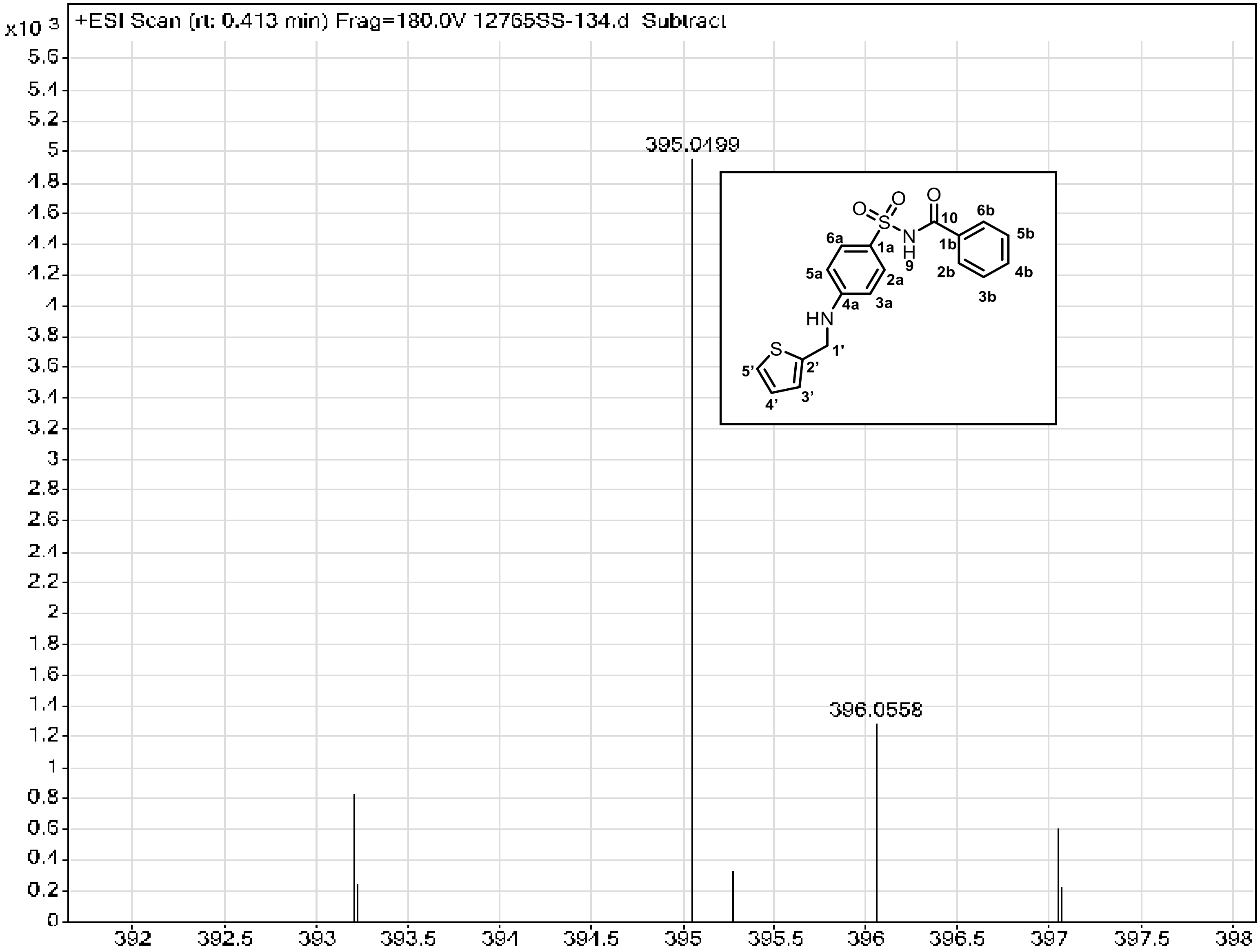

(+)-ESI-HRMS Spectrum of Sulfabenzamide-**62** (**77**)

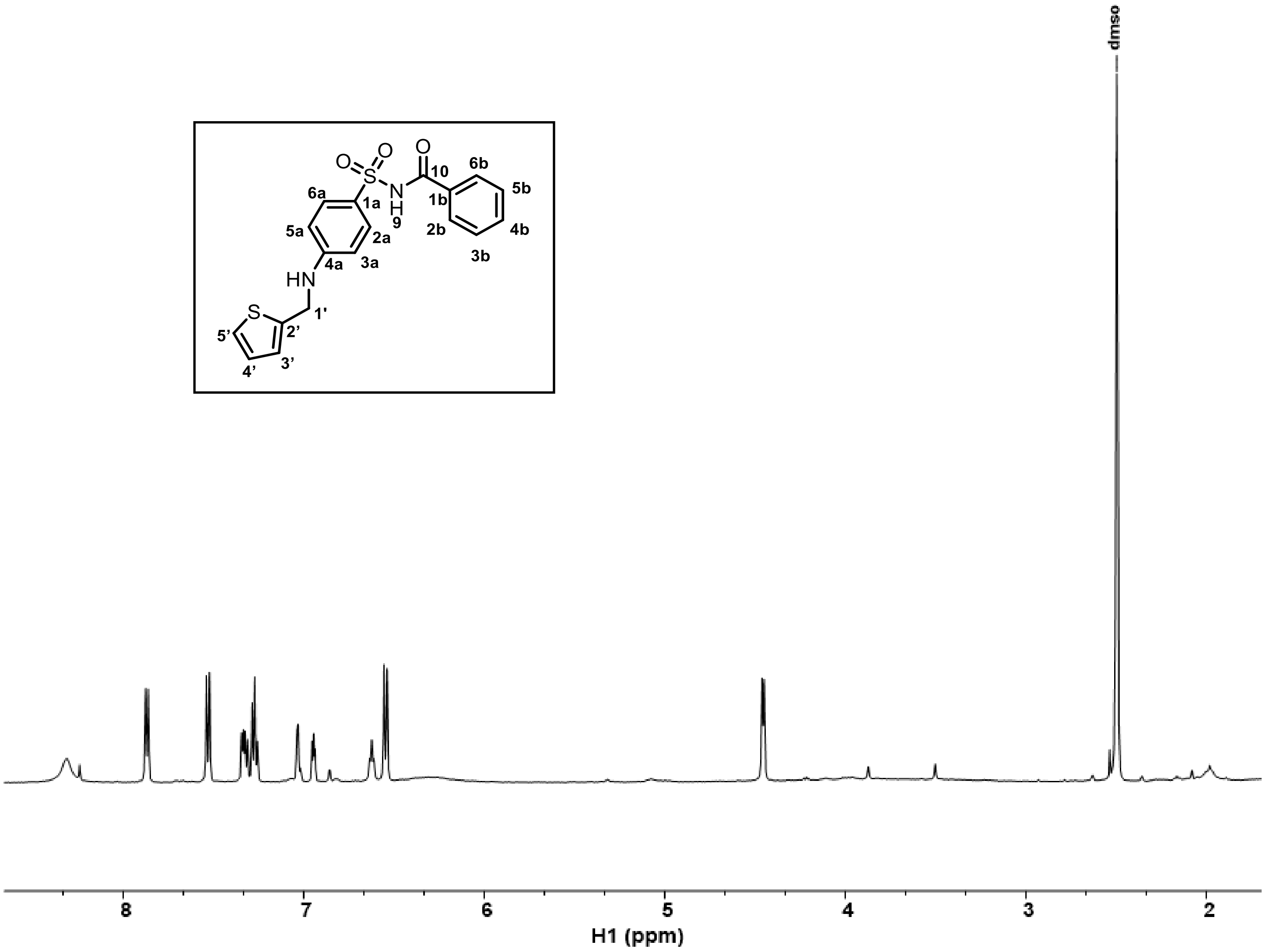

^{1}H NMR Spectrum of Sulfabenzamide-**62** (**77**) (500 MHz, DMSO-d6)

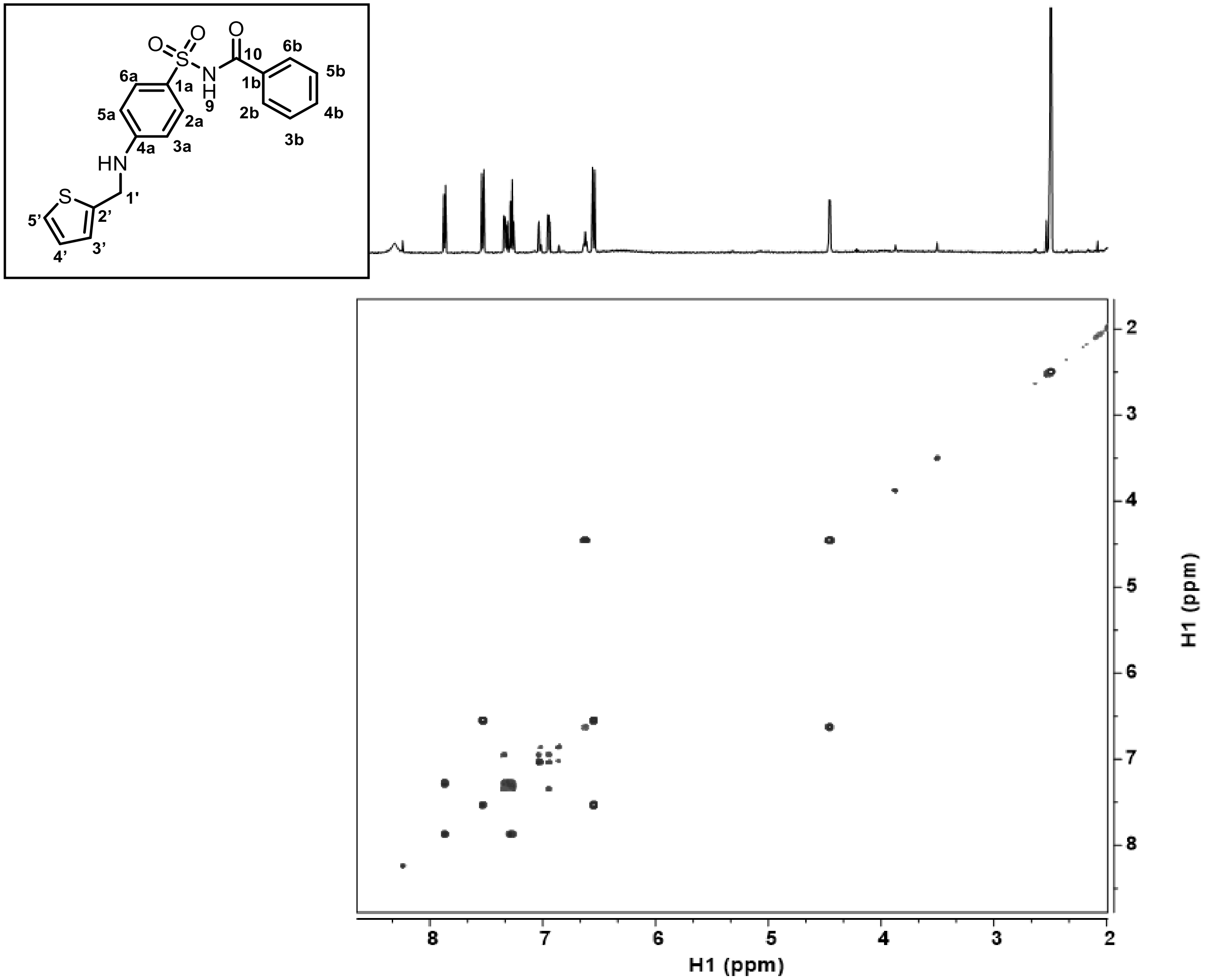

2D ^{1}H-^{1}H COSY NMR Spectrum of Sulfabenzamide-**62** (**77**) (500 MHz, DMSO-d6)

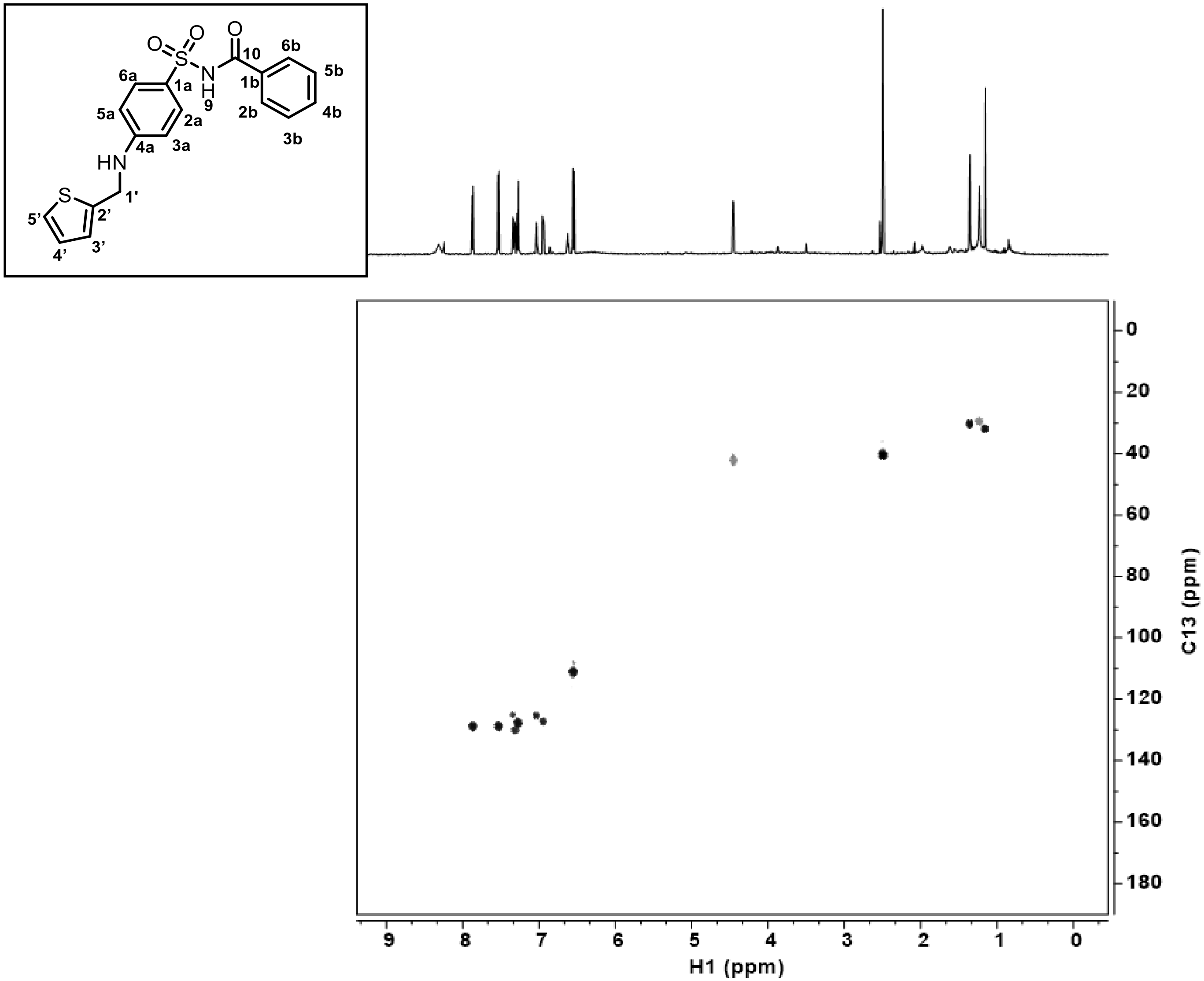

2D ^{1}H-^{13}C HSQC NMR Spectrum of Sulfabenzamide-**62** (**77**) (500 MHz, DMSO-d6)

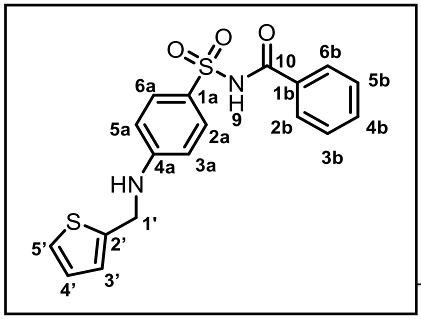

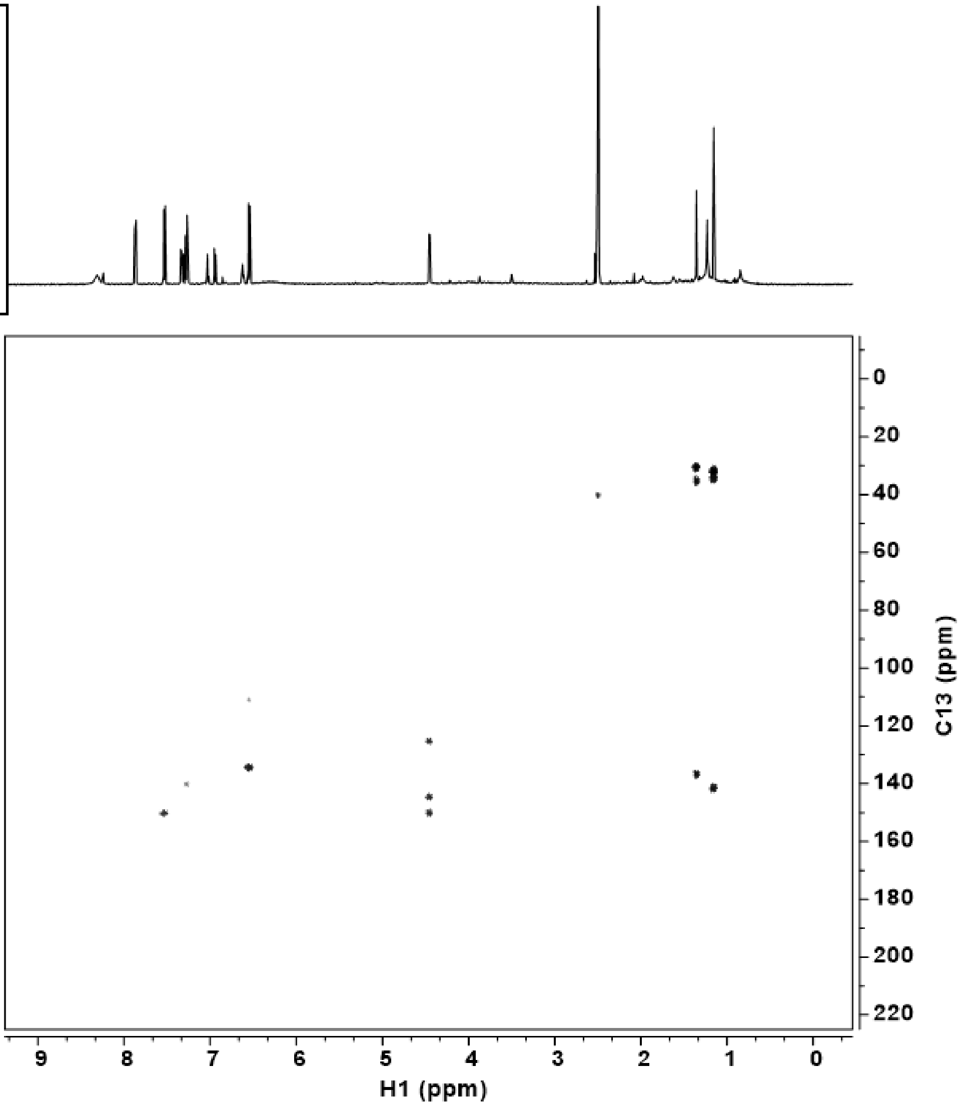

2D ^{1}H-^{13}C HMBC NMR of Sulfabenzamide-**62** (**77**) (500 MHz, DMSO-d6)

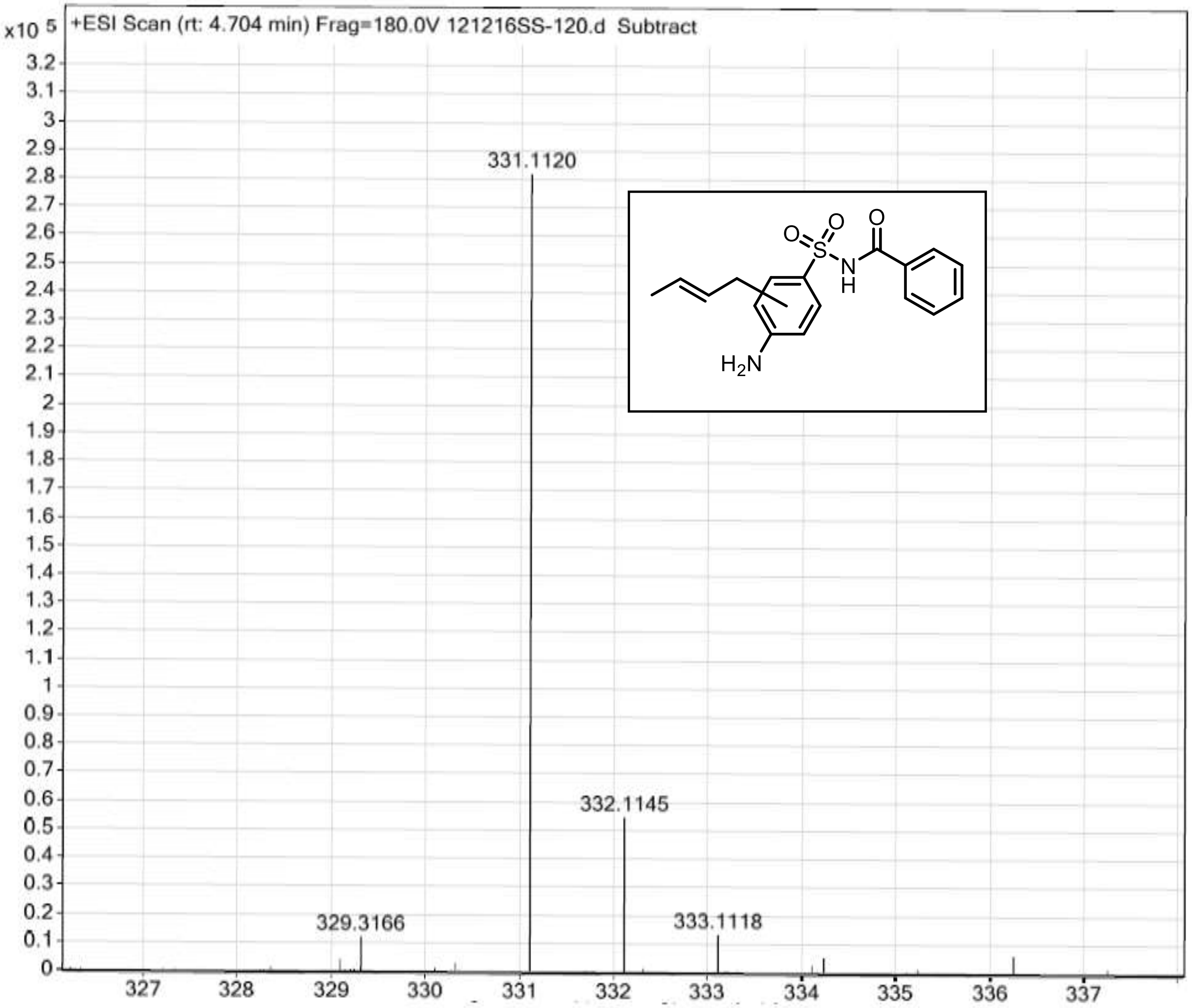

(+)-ESI-HRMS Spectrum of Sulfabenzamide-**1**

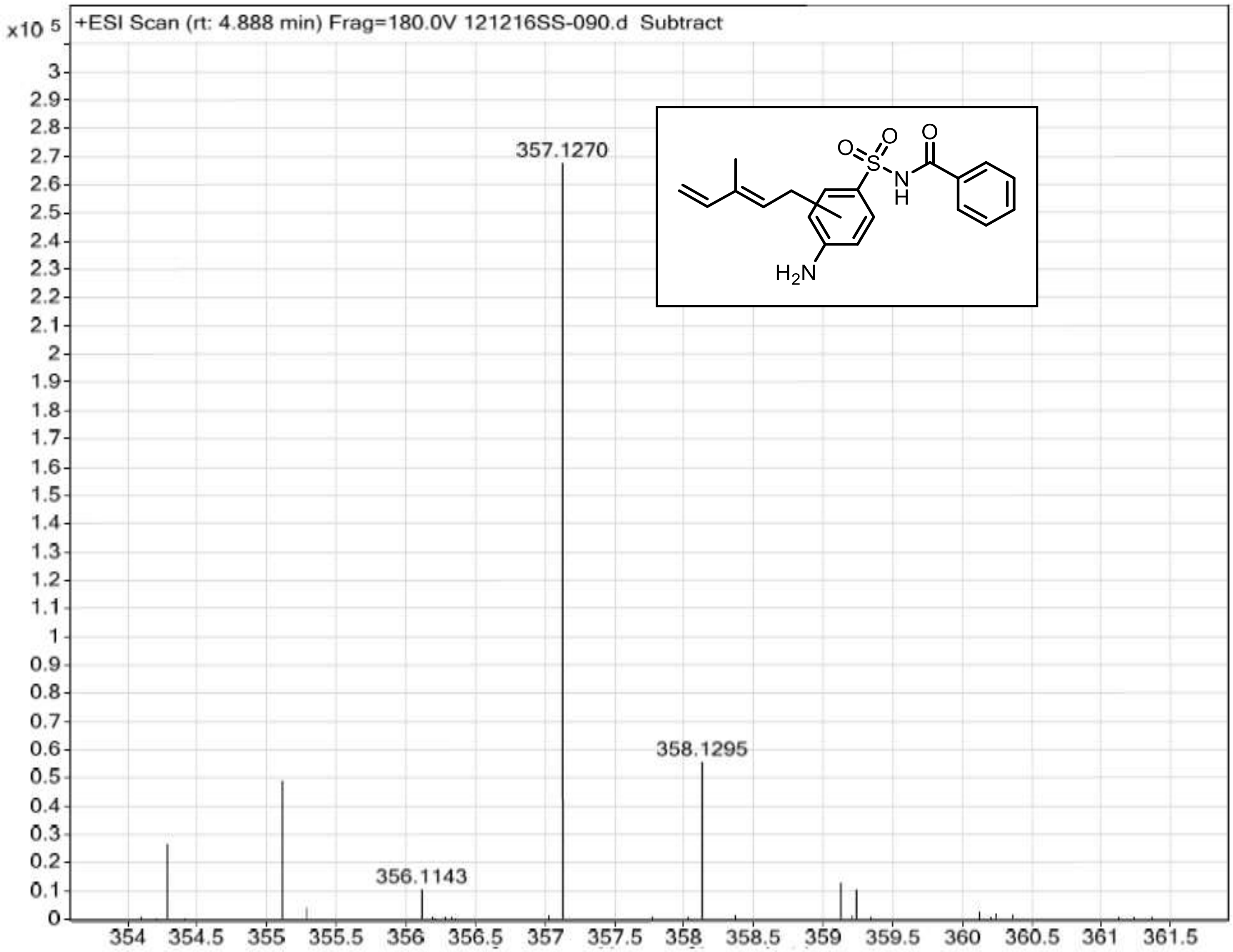

(+)-ESI-HRMS Spectrum of Sulfabenzamide-**8**

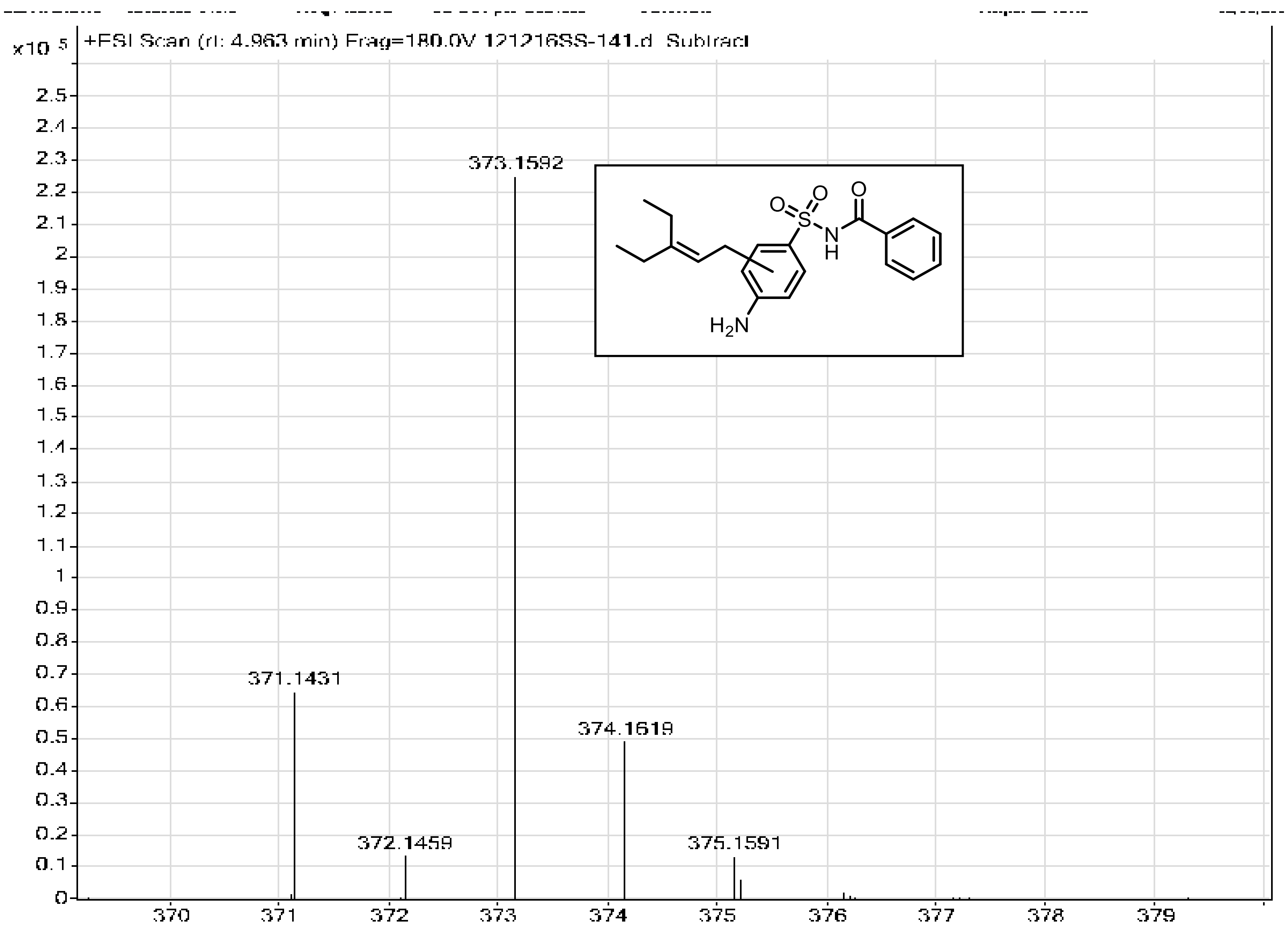

(+)-ESI-HRMS Spectrum of Sulfabenzamide-**11**

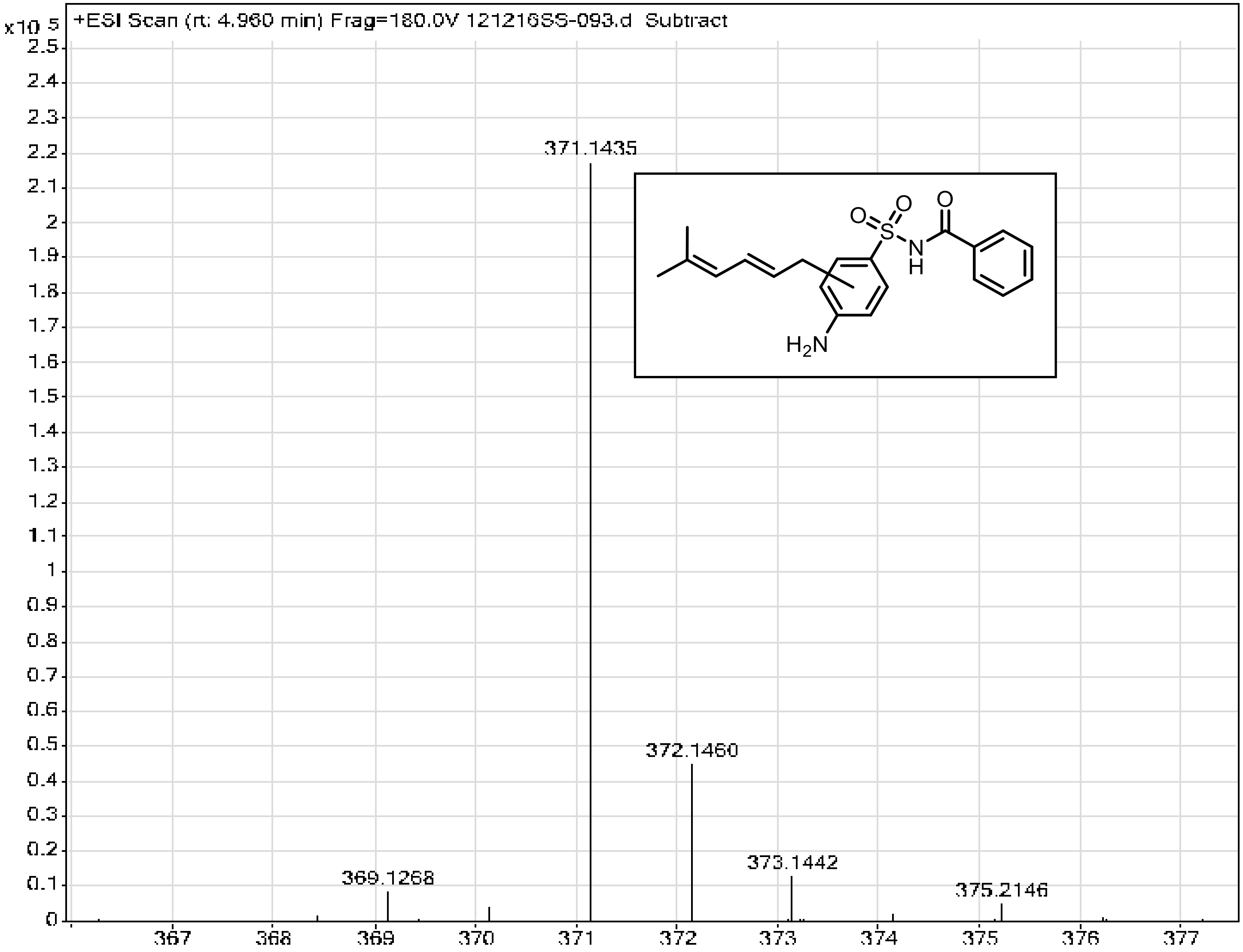

(+)-ESI-HRMS Spectrum of Sulfabenzamide-**17**

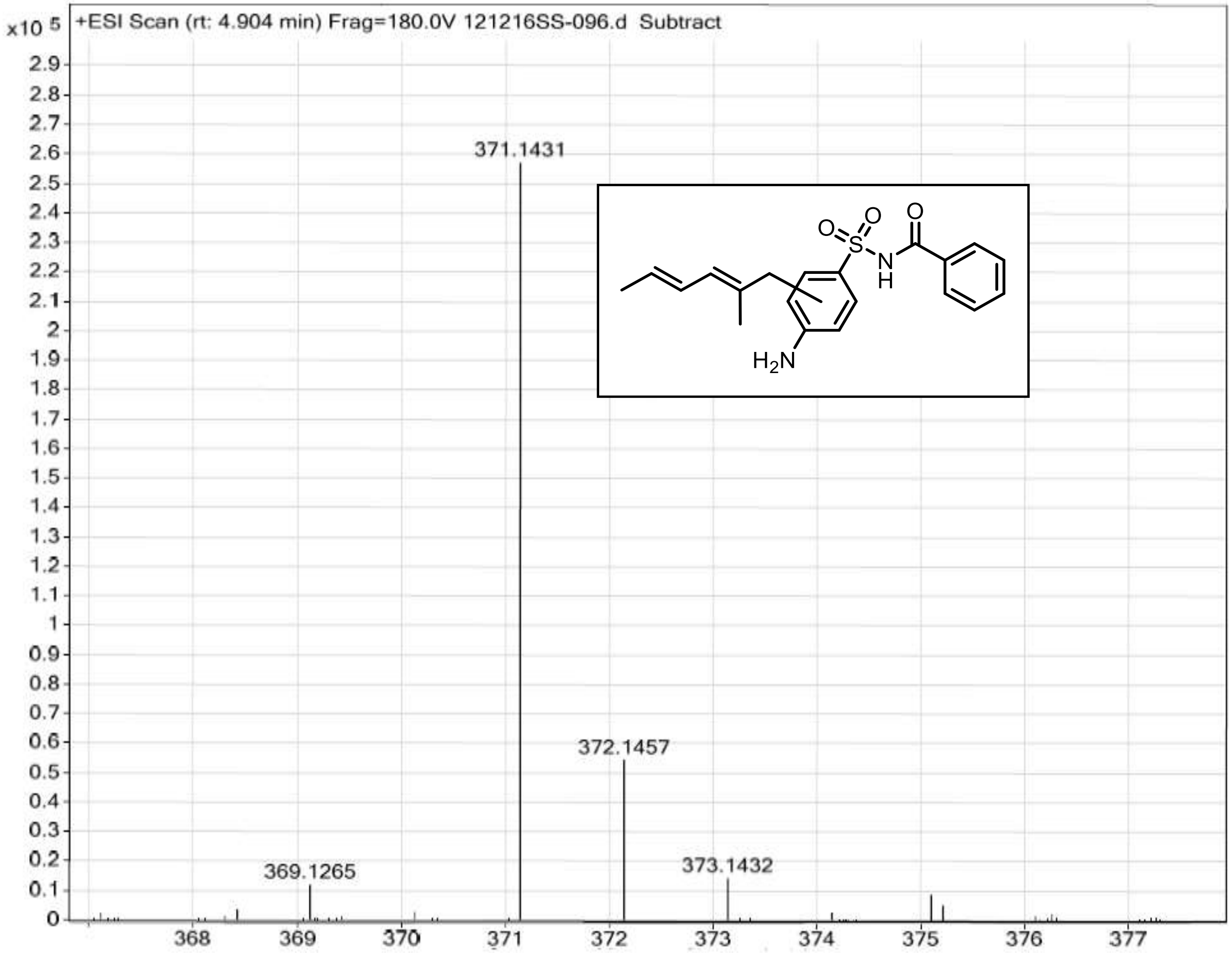

(+)-ESI-HRMS Spectrum of Sulfabenzamide-**18**

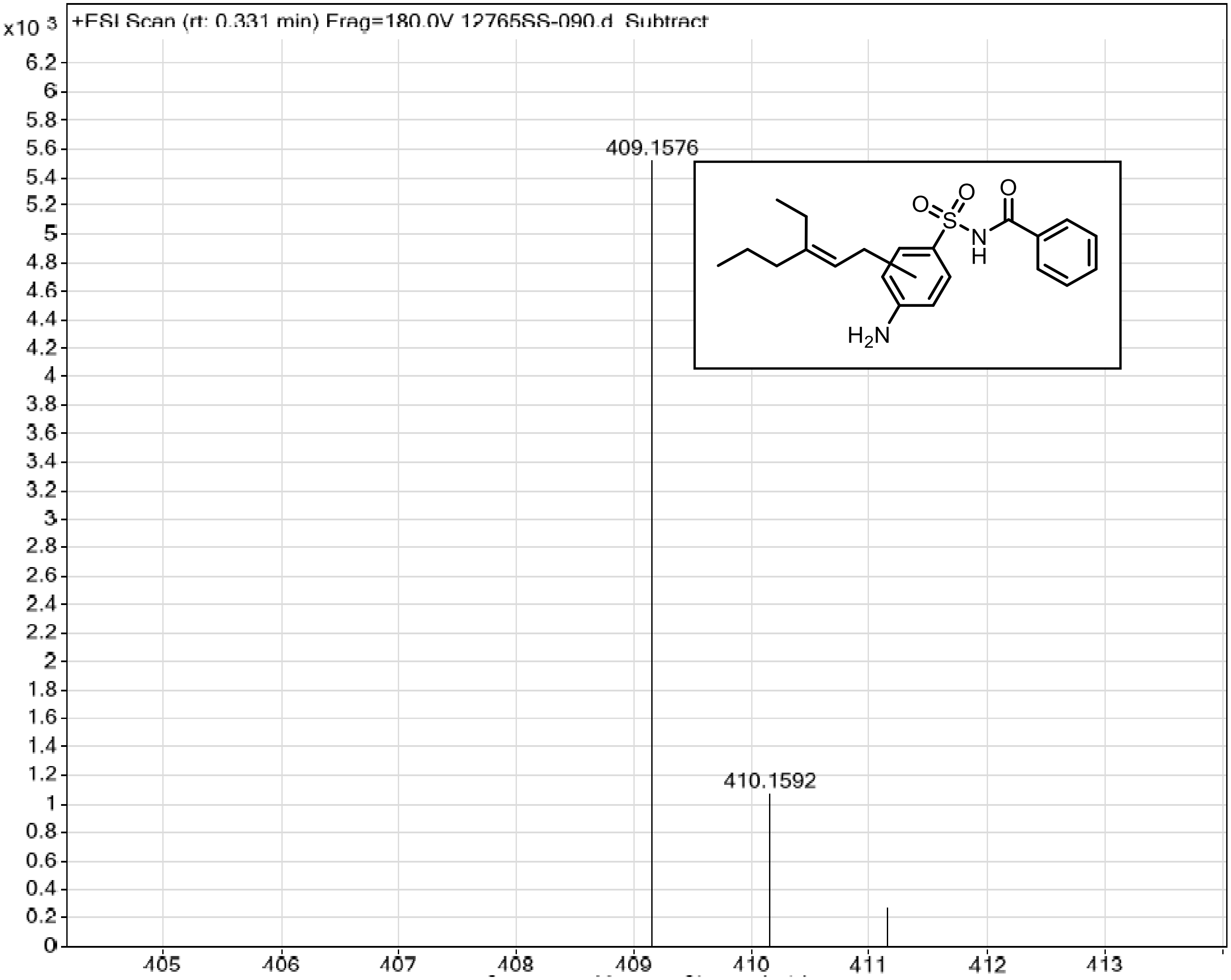

(+)-ESI-HRMS Spectrum of Sulfabenzamide-**19**

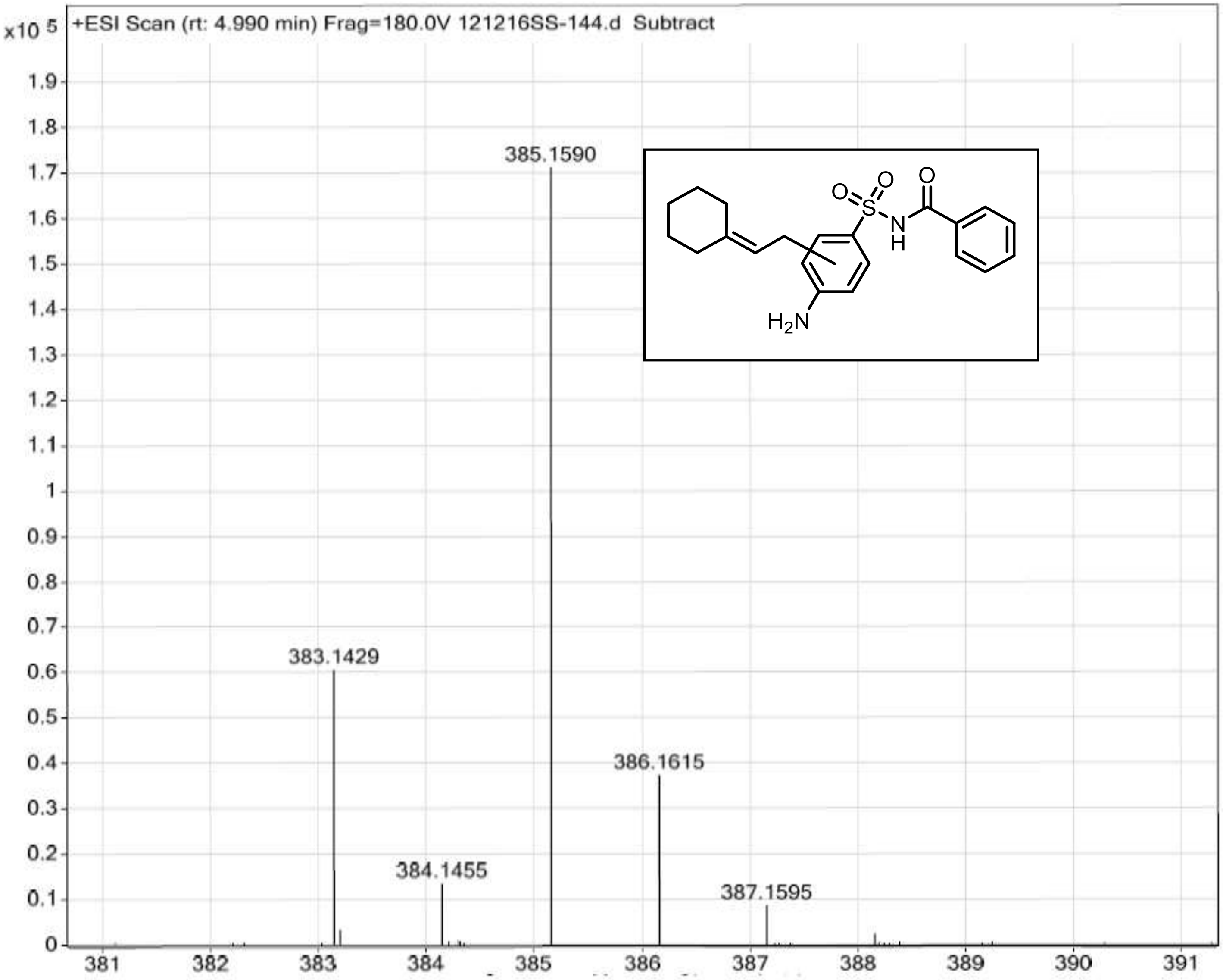

(+)-ESI-HRMS Spectrum of Sulfabenzamide-**20**

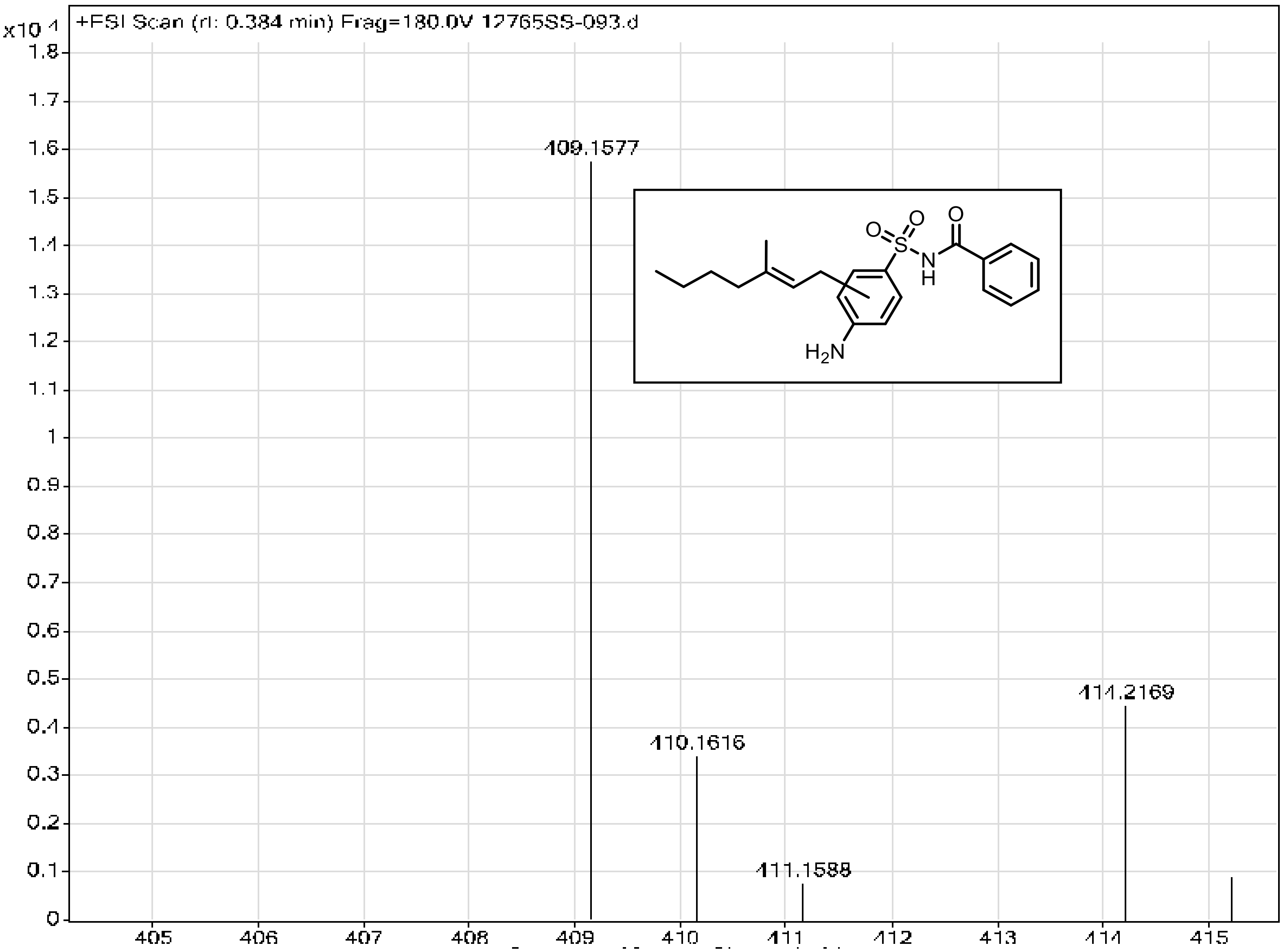

(+)-ESI-HRMS Spectrum of Sulfabenzamide-**21**

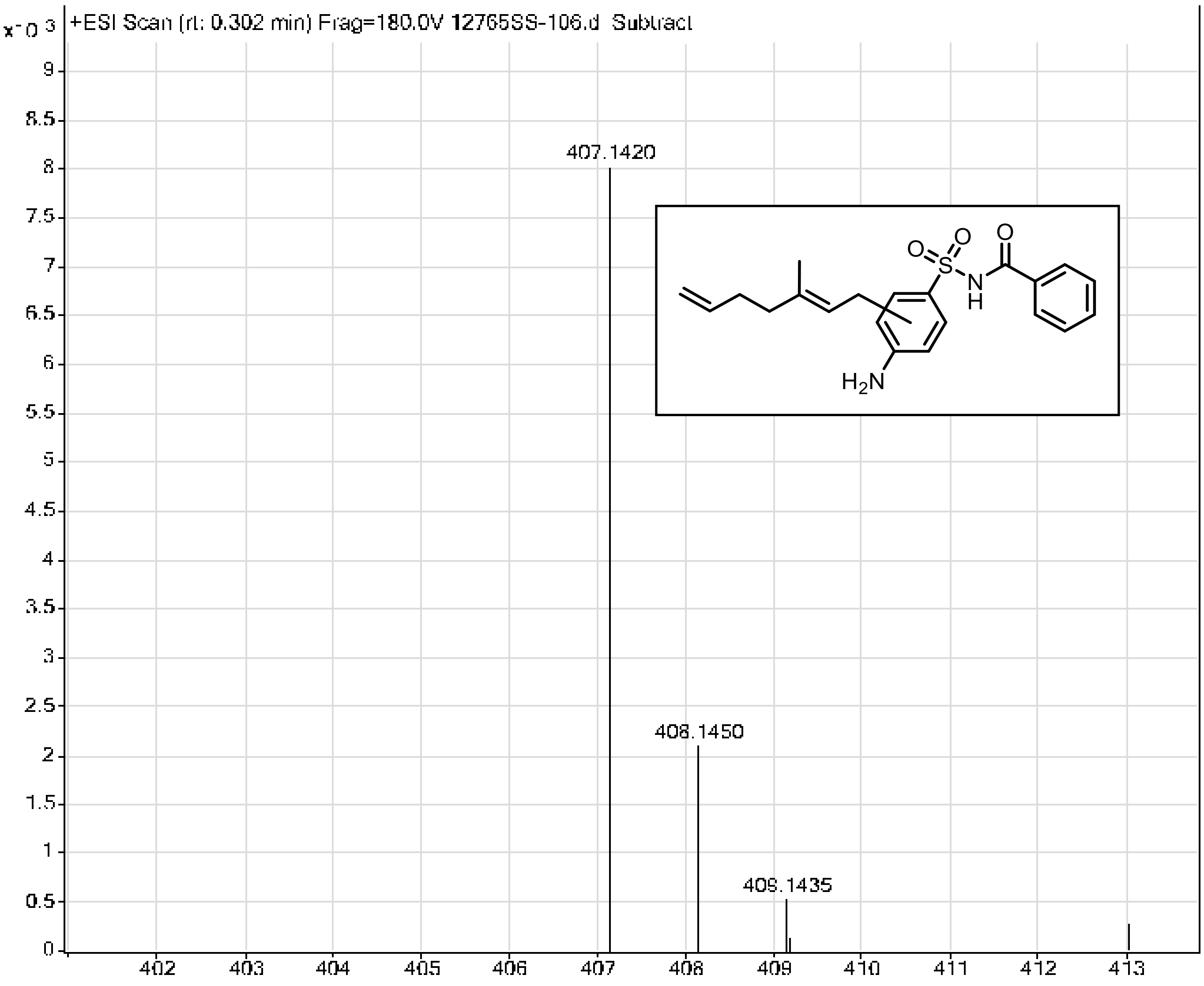

(+)-ESI-HRMS Spectrum of Sulfabenzamide-**22**

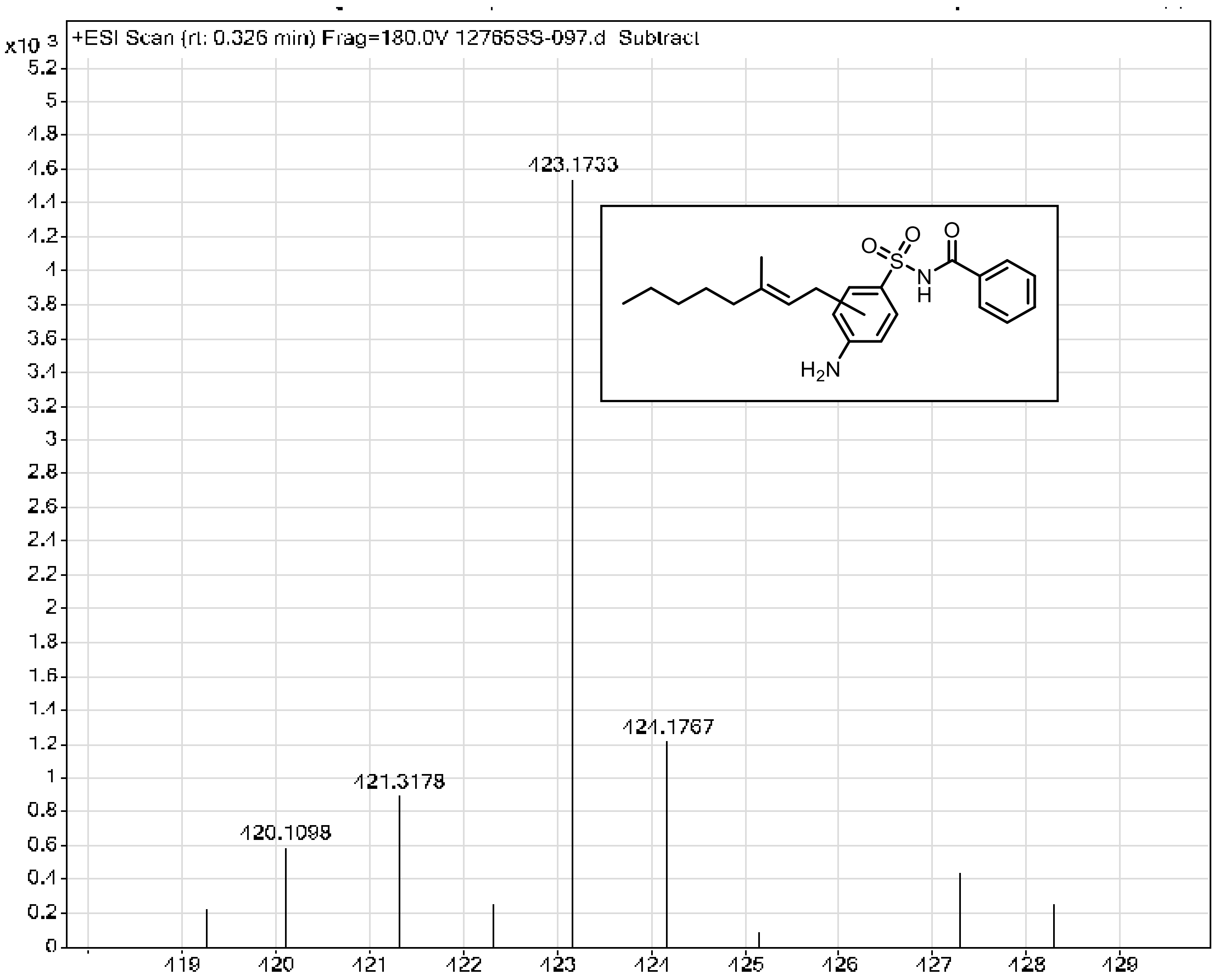

(+)-ESI-HRMS Spectrum of Sulfabenzamide-**31**

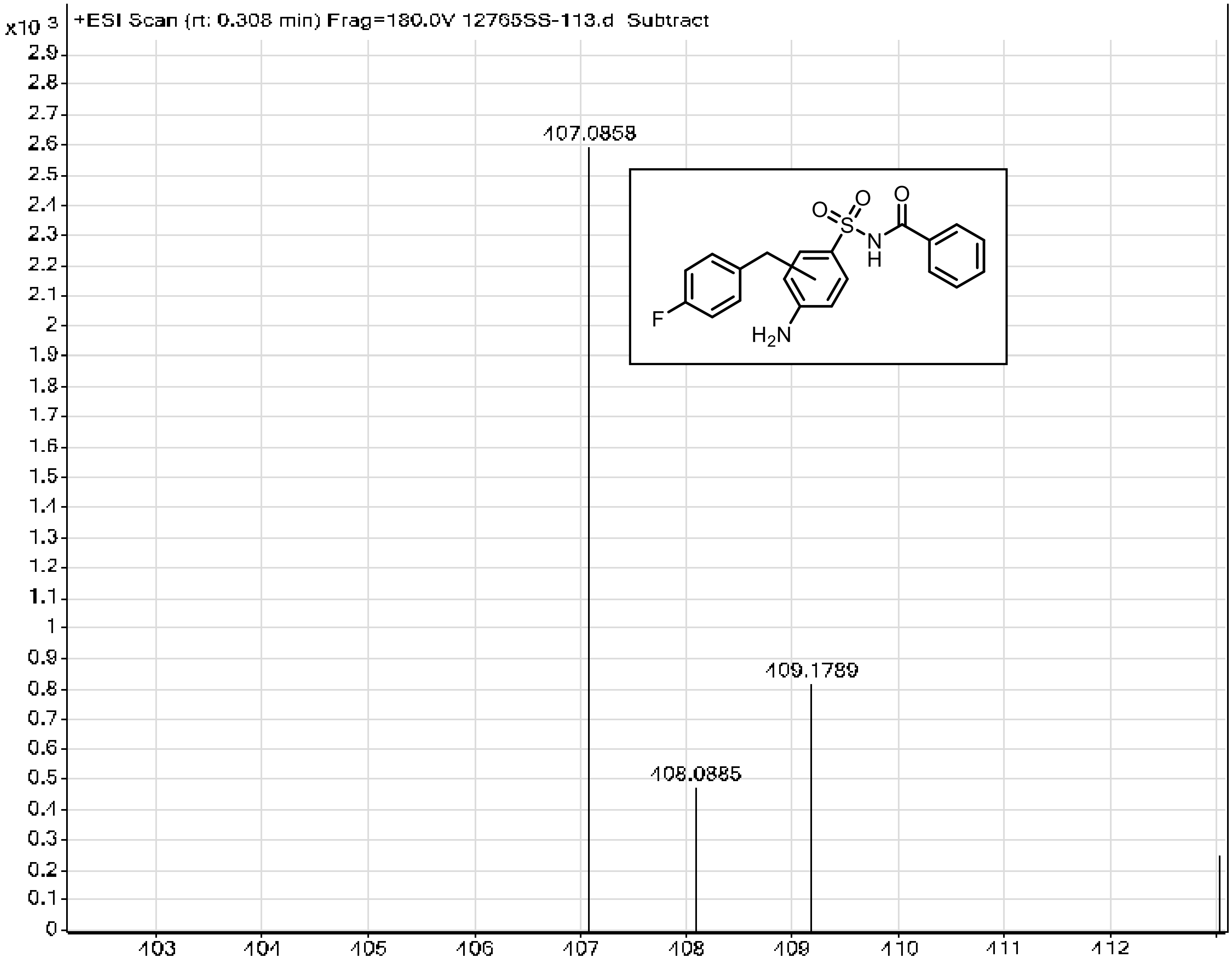

(+)-ESI-HRMS Spectrum of Sulfabenzamide-**47**

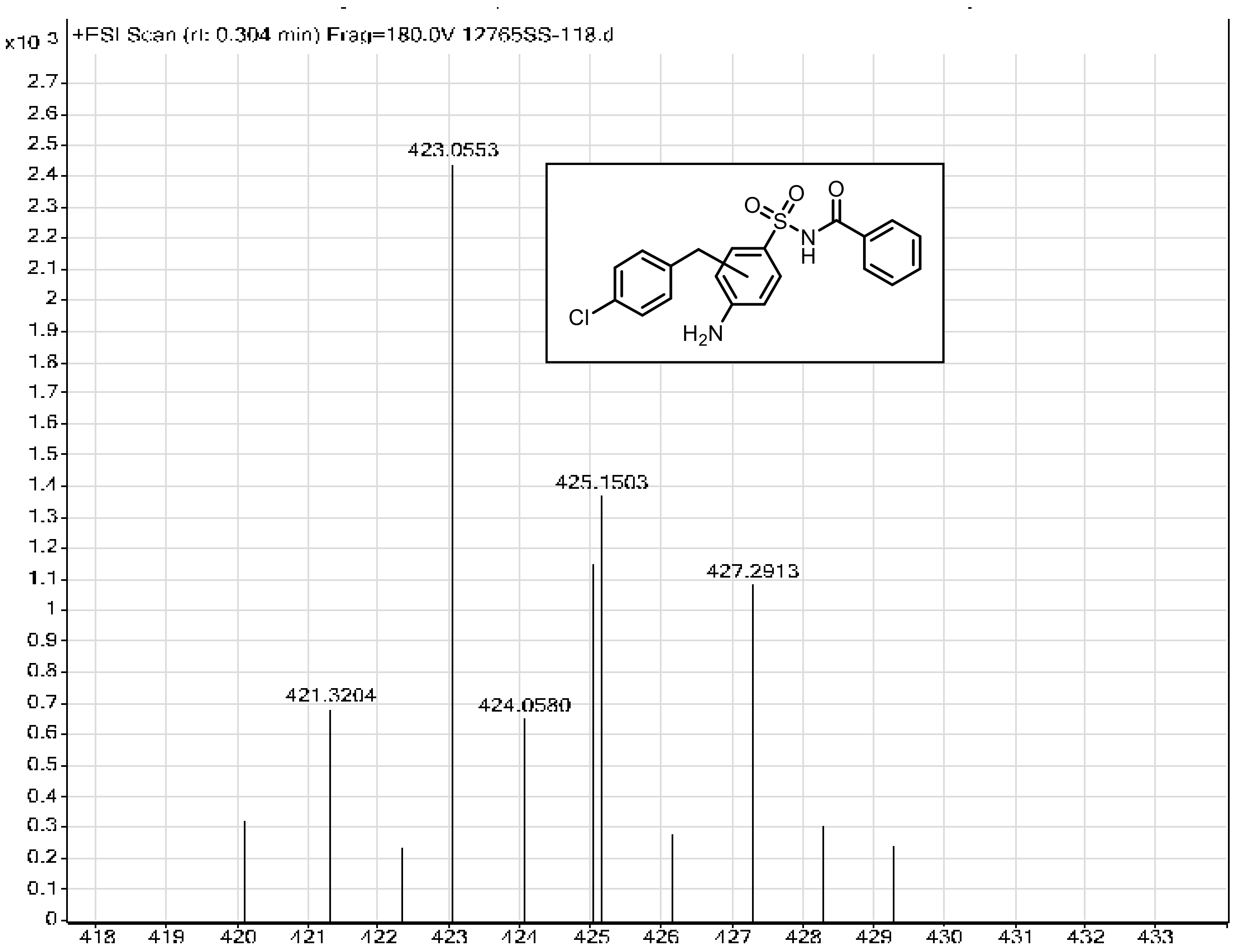

(+)-ESI-HRMS Spectrum of Sulfabenzamide-**49**

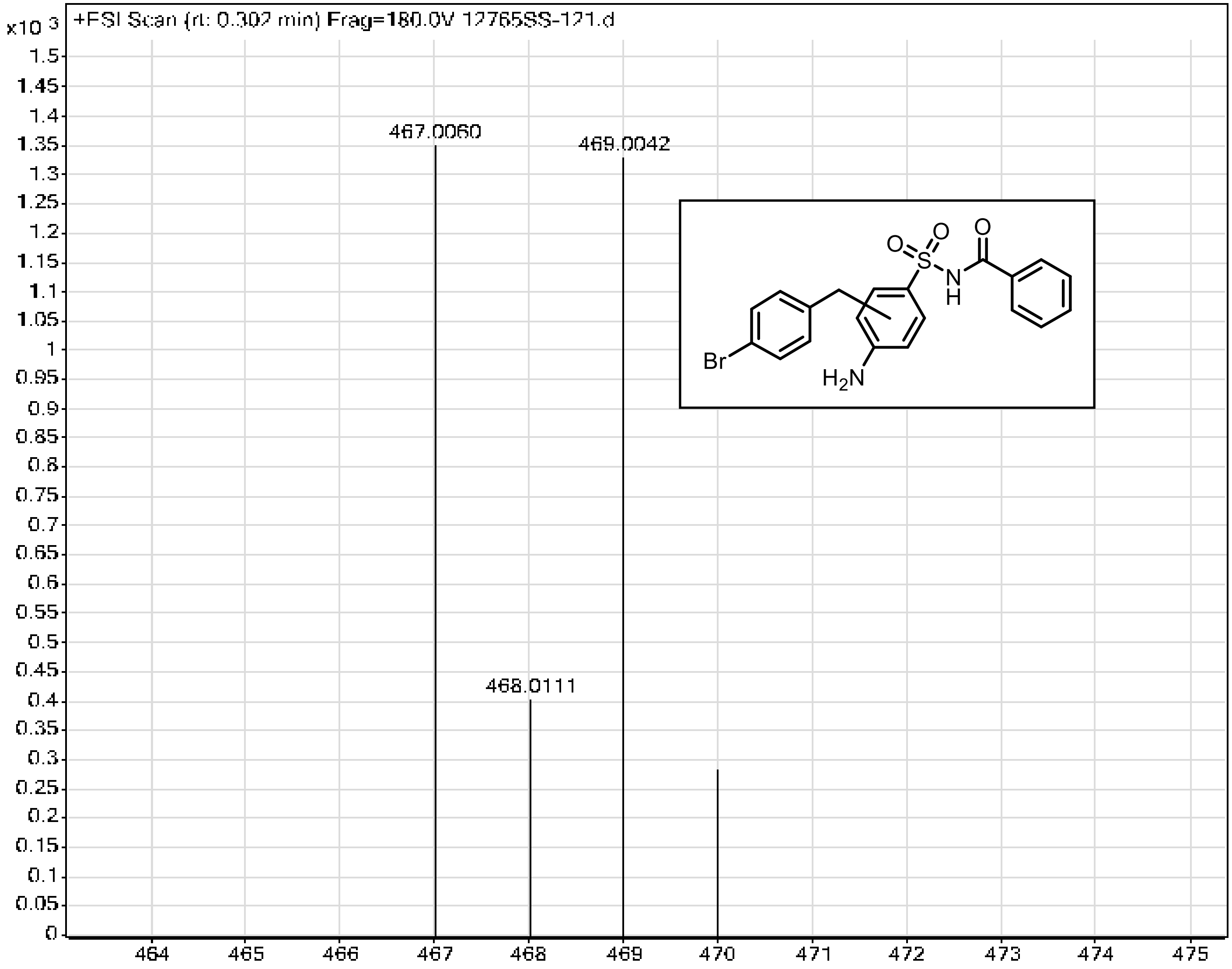

(+)-ESI-HRMS Spectrum of Sulfabenzamide-**50**

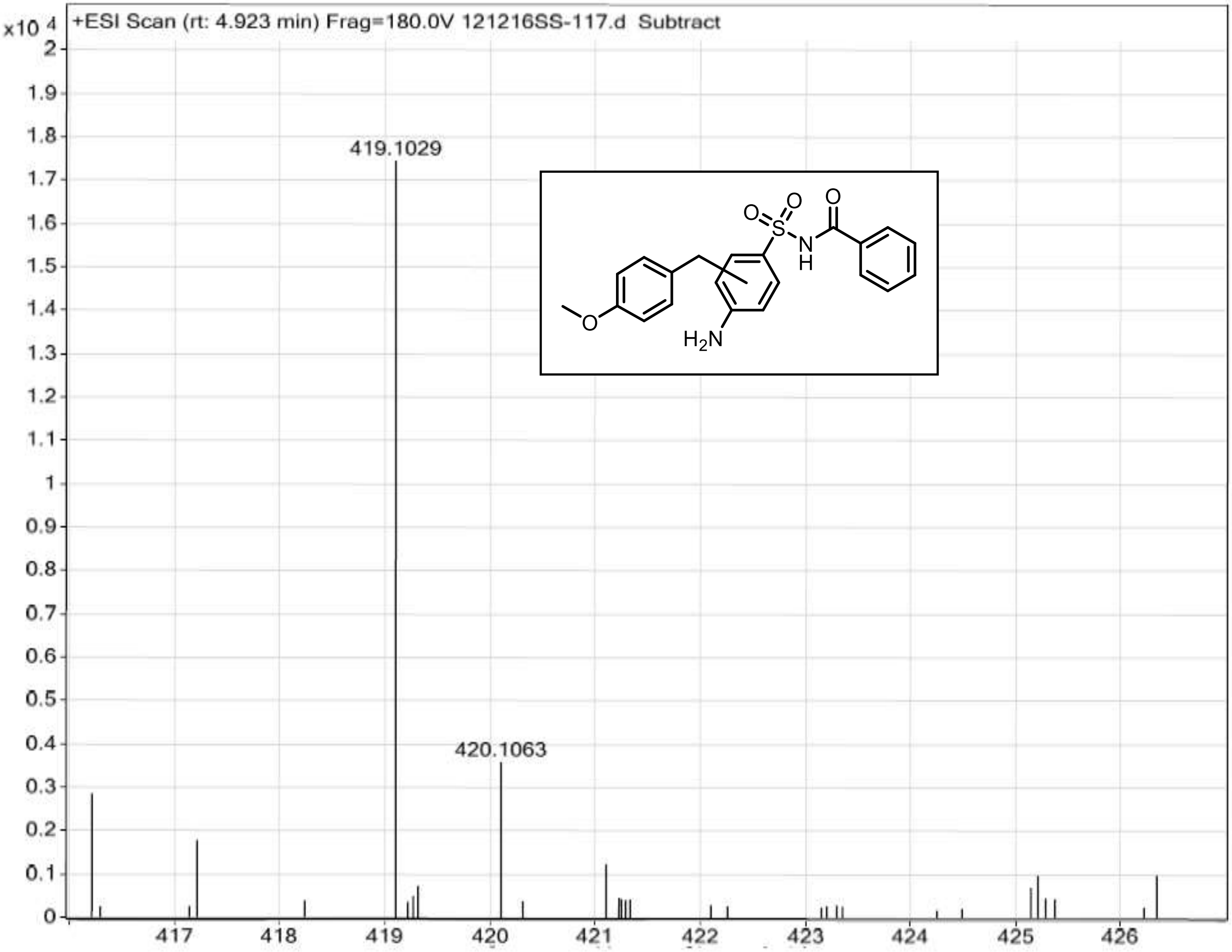

(+)-ESI-HRMS Spectrum of Sulfabenzamide-**52**

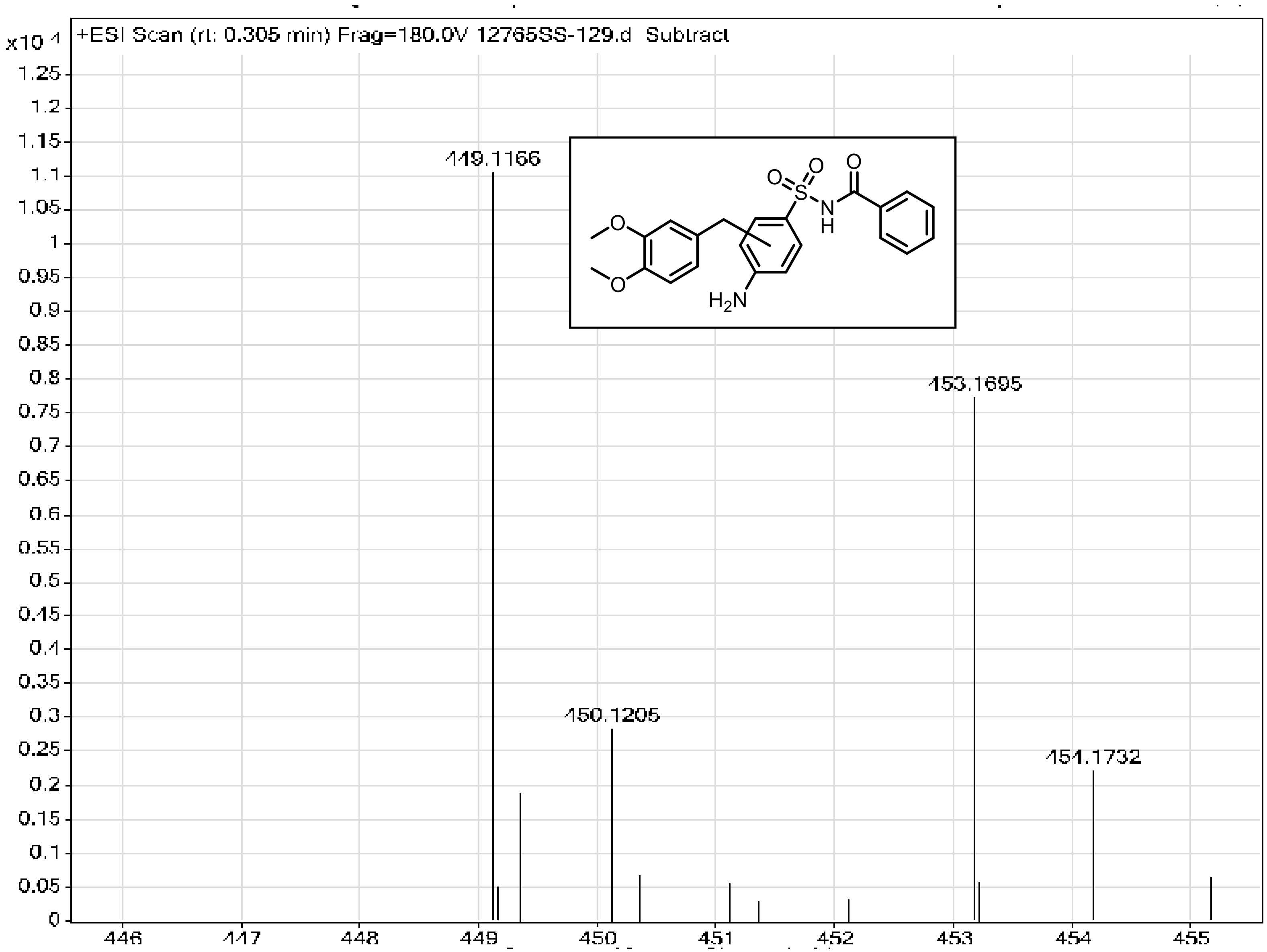

(+)-ESI-HRMS Spectrum of Sulfabenzamide-**56**

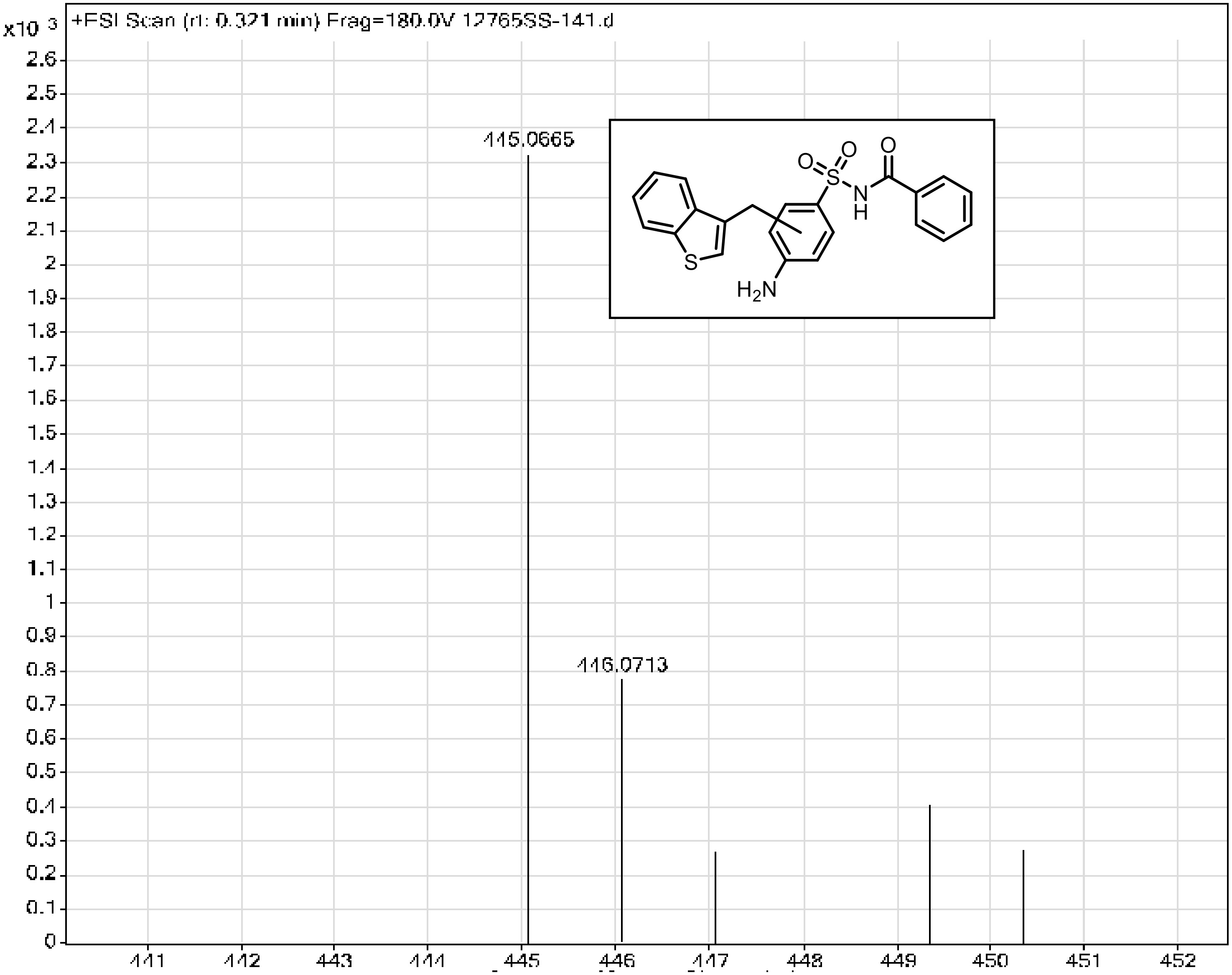

(+)-ESI-HRMS Spectrum of Sulfabenzamide-**66**
